# Analyses of non-coding somatic drivers in 2,658 cancer whole genomes

**DOI:** 10.1038/s41586-020-1965-x

**Published:** 2020-02-05

**Authors:** Esther Rheinbay, Morten Muhlig Nielsen, Federico Abascal, Jeremiah A. Wala, Ofer Shapira, Grace Tiao, Henrik Hornshøj, Julian M. Hess, Randi Istrup Juul, Ziao Lin, Lars Feuerbach, Radhakrishnan Sabarinathan, Tobias Madsen, Jaegil Kim, Loris Mularoni, Shimin Shuai, Andrés Lanzós, Carl Herrmann, Yosef E. Maruvka, Ciyue Shen, Samirkumar B. Amin, Pratiti Bandopadhayay, Johanna Bertl, Keith A. Boroevich, John Busanovich, Joana Carlevaro-Fita, Dimple Chakravarty, Calvin Wing Yiu Chan, David Craft, Priyanka Dhingra, Klev Diamanti, Nuno A. Fonseca, Abel Gonzalez-Perez, Qianyun Guo, Mark P. Hamilton, Nicholas J. Haradhvala, Chen Hong, Keren Isaev, Todd A. Johnson, Malene Juul, Andre Kahles, Abdullah Kahraman, Youngwook Kim, Jan Komorowski, Kiran Kumar, Sushant Kumar, Donghoon Lee, Kjong-Van Lehmann, Yilong Li, Eric Minwei Liu, Lucas Lochovsky, Keunchil Park, Oriol Pich, Nicola D. Roberts, Gordon Saksena, Steven E. Schumacher, Nikos Sidiropoulos, Lina Sieverling, Nasa Sinnott-Armstrong, Chip Stewart, David Tamborero, Jose M. C. Tubio, Husen M. Umer, Liis Uusküla-Reimand, Claes Wadelius, Lina Wadi, Xiaotong Yao, Cheng-Zhong Zhang, Jing Zhang, James E. Haber, Asger Hobolth, Marcin Imielinski, Manolis Kellis, Michael S. Lawrence, Christian von Mering, Hidewaki Nakagawa, Benjamin J. Raphael, Mark A. Rubin, Chris Sander, Lincoln D. Stein, Joshua M. Stuart, Tatsuhiko Tsunoda, David A. Wheeler, Rory Johnson, Jüri Reimand, Mark Gerstein, Ekta Khurana, Peter J. Campbell, Núria López-Bigas, PCAWG Drivers and Functional Interpretation Working Group, Federico Abascal, Samirkumar B. Amin, Gary D. Bader, Pratiti Bandopadhayay, Jonathan Barenboim, Rameen Beroukhim, Johanna Bertl, Keith A. Boroevich, Søren Brunak, Peter J. Campbell, Joana Carlevaro-Fita, Dimple Chakravarty, Calvin Wing Yiu Chan, Ken Chen, Jung Kyoon Choi, Jordi Deu-Pons, Priyanka Dhingra, Klev Diamanti, Lars Feuerbach, J. Lynn Fink, Nuno A. Fonseca, Joan Frigola, Carlo Gambacorti-Passerini, Dale W. Garsed, Mark Gerstein, Gad Getz, Qianyun Guo, Ivo G. Gut, David Haan, Mark P. Hamilton, Nicholas J. Haradhvala, Arif O. Harmanci, Mohamed Helmy, Carl Herrmann, Julian M. Hess, Asger Hobolth, Ermin Hodzic, Chen Hong, Henrik Hornshøj, Keren Isaev, Jose M. G. Izarzugaza, Rory Johnson, Todd A. Johnson, Malene Juul, Randi Istrup Juul, Andre Kahles, Abdullah Kahraman, Manolis Kellis, Ekta Khurana, Jaegil Kim, Jong K. Kim, Youngwook Kim, Jan Komorowski, Jan O. Korbel, Sushant Kumar, Andrés Lanzós, Erik Larsson, Michael S. Lawrence, Donghoon Lee, Kjong-Van Lehmann, Shantao Li, Xiaotong Li, Ziao Lin, Eric Minwei Liu, Lucas Lochovsky, Shaoke Lou, Tobias Madsen, Kathleen Marchal, Iñigo Martincorena, Alexander Martinez-Fundichely, Yosef E. Maruvka, Patrick D. McGillivray, William Meyerson, Ferran Muiños, Loris Mularoni, Hidewaki Nakagawa, Morten Muhlig Nielsen, Marta Paczkowska, Keunchil Park, Kiejung Park, Jakob Skou Pedersen, Tirso Pons, Sergio Pulido-Tamayo, Benjamin J. Raphael, Jüri Reimand, Iker Reyes-Salazar, Matthew A. Reyna, Esther Rheinbay, Mark A. Rubin, Carlota Rubio-Perez, S. Cenk Sahinalp, Gordon Saksena, Leonidas Salichos, Chris Sander, Steven E. Schumacher, Mark Shackleton, Ofer Shapira, Ciyue Shen, Raunak Shrestha, Shimin Shuai, Nikos Sidiropoulos, Lina Sieverling, Nasa Sinnott-Armstrong, Lincoln D. Stein, Joshua M. Stuart, David Tamborero, Grace Tiao, Tatsuhiko Tsunoda, Husen M. Umer, Liis Uusküla-Reimand, Alfonso Valencia, Miguel Vazquez, Lieven P. C. Verbeke, Claes Wadelius, Lina Wadi, Jiayin Wang, Jonathan Warrell, Sebastian M. Waszak, Joachim Weischenfeldt, David A. Wheeler, Guanming Wu, Jun Yu, Jing Zhang, Xuanping Zhang, Yan Zhang, Zhongming Zhao, Lihua Zou, Christian von Mering, PCAWG Structural Variation Working Group, Kadir C. Akdemir, Eva G. Alvarez, Adrian Baez-Ortega, Rameen Beroukhim, Paul C. Boutros, David D. L. Bowtell, Benedikt Brors, Kathleen H. Burns, John Busanovich, Peter J. Campbell, Kin Chan, Ken Chen, Isidro Cortés-Ciriano, Ana Dueso-Barroso, Andrew J. Dunford, Paul A. Edwards, Xavier Estivill, Dariush Etemadmoghadam, Lars Feuerbach, J. Lynn Fink, Milana Frenkel-Morgenstern, Dale W. Garsed, Mark Gerstein, Dmitry A. Gordenin, David Haan, James E. Haber, Julian M. Hess, Barbara Hutter, Marcin Imielinski, David T. W. Jones, Young Seok Ju, Marat D. Kazanov, Leszek J. Klimczak, Youngil Koh, Jan O. Korbel, Kiran Kumar, Eunjung Alice Lee, Jake June-Koo Lee, Yilong Li, Andy G. Lynch, Geoff Macintyre, Florian Markowetz, Iñigo Martincorena, Alexander Martinez-Fundichely, Matthew Meyerson, Satoru Miyano, Hidewaki Nakagawa, Fabio C. P. Navarro, Stephan Ossowski, Peter J. Park, John V. Pearson, Montserrat Puiggròs, Karsten Rippe, Nicola D. Roberts, Steven A. Roberts, Bernardo Rodriguez-Martin, Steven E. Schumacher, Ralph Scully, Mark Shackleton, Nikos Sidiropoulos, Lina Sieverling, Chip Stewart, David Torrents, Jose M. C. Tubio, Izar Villasante, Nicola Waddell, Jeremiah A. Wala, Joachim Weischenfeldt, Lixing Yang, Xiaotong Yao, Sung-Soo Yoon, Jorge Zamora, Cheng-Zhong Zhang, Joachim Weischenfeldt, Rameen Beroukhim, Iñigo Martincorena, Jakob Skou Pedersen, Gad Getz, PCAWG Consortium, Lauri A. Aaltonen, Federico Abascal, Adam Abeshouse, Hiroyuki Aburatani, David J. Adams, Nishant Agrawal, Keun Soo Ahn, Sung-Min Ahn, Hiroshi Aikata, Rehan Akbani, Kadir C. Akdemir, Hikmat Al-Ahmadie, Sultan T. Al-Sedairy, Fatima Al-Shahrour, Malik Alawi, Monique Albert, Kenneth Aldape, Ludmil B. Alexandrov, Adrian Ally, Kathryn Alsop, Eva G. Alvarez, Fernanda Amary, Samirkumar B. Amin, Brice Aminou, Ole Ammerpohl, Matthew J. Anderson, Yeng Ang, Davide Antonello, Pavana Anur, Samuel Aparicio, Elizabeth L. Appelbaum, Yasuhito Arai, Axel Aretz, Koji Arihiro, Shun-ichi Ariizumi, Joshua Armenia, Laurent Arnould, Sylvia Asa, Yassen Assenov, Gurnit Atwal, Sietse Aukema, J. Todd Auman, Miriam R. R. Aure, Philip Awadalla, Marta Aymerich, Gary D. Bader, Adrian Baez-Ortega, Matthew H. Bailey, Peter J. Bailey, Miruna Balasundaram, Saianand Balu, Pratiti Bandopadhayay, Rosamonde E. Banks, Stefano Barbi, Andrew P. Barbour, Jonathan Barenboim, Jill Barnholtz-Sloan, Hugh Barr, Elisabet Barrera, John Bartlett, Javier Bartolome, Claudio Bassi, Oliver F. Bathe, Daniel Baumhoer, Prashant Bavi, Stephen B. Baylin, Wojciech Bazant, Duncan Beardsmore, Timothy A. Beck, Sam Behjati, Andreas Behren, Beifang Niu, Cindy Bell, Sergi Beltran, Christopher Benz, Andrew Berchuck, Anke K. Bergmann, Erik N. Bergstrom, Benjamin P. Berman, Daniel M. Berney, Stephan H. Bernhart, Rameen Beroukhim, Mario Berrios, Samantha Bersani, Johanna Bertl, Miguel Betancourt, Vinayak Bhandari, Shriram G. Bhosle, Andrew V. Biankin, Matthias Bieg, Darell Bigner, Hans Binder, Ewan Birney, Michael Birrer, Nidhan K. Biswas, Bodil Bjerkehagen, Tom Bodenheimer, Lori Boice, Giada Bonizzato, Johann S. De Bono, Arnoud Boot, Moiz S. Bootwalla, Ake Borg, Arndt Borkhardt, Keith A. Boroevich, Ivan Borozan, Christoph Borst, Marcus Bosenberg, Mattia Bosio, Jacqueline Boultwood, Guillaume Bourque, Paul C. Boutros, G. Steven Bova, David T. Bowen, Reanne Bowlby, David D. L. Bowtell, Sandrine Boyault, Rich Boyce, Jeffrey Boyd, Alvis Brazma, Paul Brennan, Daniel S. Brewer, Arie B. Brinkman, Robert G. Bristow, Russell R. Broaddus, Jane E. Brock, Malcolm Brock, Annegien Broeks, Angela N. Brooks, Denise Brooks, Benedikt Brors, Søren Brunak, Timothy J. C. Bruxner, Alicia L. Bruzos, Alex Buchanan, Ivo Buchhalter, Christiane Buchholz, Susan Bullman, Hazel Burke, Birgit Burkhardt, Kathleen H. Burns, John Busanovich, Carlos D. Bustamante, Adam P. Butler, Atul J. Butte, Niall J. Byrne, Anne-Lise Børresen-Dale, Samantha J. Caesar-Johnson, Andy Cafferkey, Declan Cahill, Claudia Calabrese, Carlos Caldas, Fabien Calvo, Niedzica Camacho, Peter J. Campbell, Elias Campo, Cinzia Cantù, Shaolong Cao, Thomas E. Carey, Joana Carlevaro-Fita, Rebecca Carlsen, Ivana Cataldo, Mario Cazzola, Jonathan Cebon, Robert Cerfolio, Dianne E. Chadwick, Dimple Chakravarty, Don Chalmers, Calvin Wing Yiu Chan, Kin Chan, Michelle Chan-Seng-Yue, Vishal S. Chandan, David K. Chang, Stephen J. Chanock, Lorraine A. Chantrill, Aurélien Chateigner, Nilanjan Chatterjee, Kazuaki Chayama, Hsiao-Wei Chen, Jieming Chen, Ken Chen, Yiwen Chen, Zhaohong Chen, Andrew D. Cherniack, Jeremy Chien, Yoke-Eng Chiew, Suet-Feung Chin, Juok Cho, Sunghoon Cho, Jung Kyoon Choi, Wan Choi, Christine Chomienne, Zechen Chong, Su Pin Choo, Angela Chou, Angelika N. Christ, Elizabeth L. Christie, Eric Chuah, Carrie Cibulskis, Kristian Cibulskis, Sara Cingarlini, Peter Clapham, Alexander Claviez, Sean Cleary, Nicole Cloonan, Marek Cmero, Colin C. Collins, Ashton A. Connor, Susanna L. Cooke, Colin S. Cooper, Leslie Cope, Vincenzo Corbo, Matthew G. Cordes, Stephen M. Cordner, Isidro Cortés-Ciriano, Kyle Covington, Prue A. Cowin, Brian Craft, David Craft, Chad J. Creighton, Yupeng Cun, Erin Curley, Ioana Cutcutache, Karolina Czajka, Bogdan Czerniak, Rebecca A. Dagg, Ludmila Danilova, Maria Vittoria Davi, Natalie R. Davidson, Helen Davies, Ian J. Davis, Brandi N. Davis-Dusenbery, Kevin J. Dawson, Francisco M. De La Vega, Ricardo De Paoli-Iseppi, Timothy Defreitas, Angelo P. Dei Tos, Olivier Delaneau, John A. Demchok, Jonas Demeulemeester, German M. Demidov, Deniz Demircioğlu, Nening M. Dennis, Robert E. Denroche, Stefan C. Dentro, Nikita Desai, Vikram Deshpande, Amit G. Deshwar, Christine Desmedt, Jordi Deu-Pons, Noreen Dhalla, Neesha C. Dhani, Priyanka Dhingra, Rajiv Dhir, Anthony DiBiase, Klev Diamanti, Li Ding, Shuai Ding, Huy Q. Dinh, Luc Dirix, HarshaVardhan Doddapaneni, Nilgun Donmez, Michelle T. Dow, Ronny Drapkin, Oliver Drechsel, Ruben M. Drews, Serge Serge, Tim Dudderidge, Ana Dueso-Barroso, Andrew J. Dunford, Michael Dunn, Lewis Jonathan Dursi, Fraser R. Duthie, Ken Dutton-Regester, Jenna Eagles, Douglas F. Easton, Stuart Edmonds, Paul A. Edwards, Sandra E. Edwards, Rosalind A. Eeles, Anna Ehinger, Juergen Eils, Roland Eils, Adel El-Naggar, Matthew Eldridge, Kyle Ellrott, Serap Erkek, Georgia Escaramis, Shadrielle M. G. Espiritu, Xavier Estivill, Dariush Etemadmoghadam, Jorunn E. Eyfjord, Bishoy M. Faltas, Daiming Fan, Yu Fan, William C. Faquin, Claudiu Farcas, Matteo Fassan, Aquila Fatima, Francesco Favero, Nodirjon Fayzullaev, Ina Felau, Sian Fereday, Martin L. Ferguson, Vincent Ferretti, Lars Feuerbach, Matthew A. Field, J. Lynn Fink, Gaetano Finocchiaro, Cyril Fisher, Matthew W. Fittall, Anna Fitzgerald, Rebecca C. Fitzgerald, Adrienne M. Flanagan, Neil E. Fleshner, Paul Flicek, John A. Foekens, Kwun M. Fong, Nuno A. Fonseca, Christopher S. Foster, Natalie S. Fox, Michael Fraser, Scott Frazer, Milana Frenkel-Morgenstern, William Friedman, Joan Frigola, Catrina C. Fronick, Akihiro Fujimoto, Masashi Fujita, Masashi Fukayama, Lucinda A. Fulton, Robert S. Fulton, Mayuko Furuta, P. Andrew Futreal, Anja Füllgrabe, Stacey B. Gabriel, Steven Gallinger, Carlo Gambacorti-Passerini, Jianjiong Gao, Shengjie Gao, Levi Garraway, Øystein Garred, Erik Garrison, Dale W. Garsed, Nils Gehlenborg, Josep L. L. Gelpi, Joshy George, Daniela S. Gerhard, Clarissa Gerhauser, Jeffrey E. Gershenwald, Mark Gerstein, Moritz Gerstung, Gad Getz, Mohammed Ghori, Ronald Ghossein, Nasra H. Giama, Richard A. Gibbs, Bob Gibson, Anthony J. Gill, Pelvender Gill, Dilip D. Giri, Dominik Glodzik, Vincent J. Gnanapragasam, Maria Elisabeth Goebler, Mary J. Goldman, Carmen Gomez, Santiago Gonzalez, Abel Gonzalez-Perez, Dmitry A. Gordenin, James Gossage, Kunihito Gotoh, Ramaswamy Govindan, Dorthe Grabau, Janet S. Graham, Robert C. Grant, Anthony R. Green, Eric Green, Liliana Greger, Nicola Grehan, Sonia Grimaldi, Sean M. Grimmond, Robert L. Grossman, Adam Grundhoff, Gunes Gundem, Qianyun Guo, Manaswi Gupta, Shailja Gupta, Ivo G. Gut, Marta Gut, Jonathan Göke, Gavin Ha, Andrea Haake, David Haan, Siegfried Haas, Kerstin Haase, James E. Haber, Nina Habermann, Faraz Hach, Syed Haider, Natsuko Hama, Freddie C. Hamdy, Anne Hamilton, Mark P. Hamilton, Leng Han, George B. Hanna, Martin Hansmann, Nicholas J. Haradhvala, Olivier Harismendy, Ivon Harliwong, Arif O. Harmanci, Eoghan Harrington, Takanori Hasegawa, David Haussler, Steve Hawkins, Shinya Hayami, Shuto Hayashi, D. Neil Hayes, Stephen J. Hayes, Nicholas K. Hayward, Steven Hazell, Yao He, Allison P. Heath, Simon C. Heath, David Hedley, Apurva M. Hegde, David I. Heiman, Michael C. Heinold, Zachary Heins, Lawrence E. Heisler, Eva Hellstrom-Lindberg, Mohamed Helmy, Seong Gu Heo, Austin J. Hepperla, José María Heredia-Genestar, Carl Herrmann, Peter Hersey, Julian M. Hess, Holmfridur Hilmarsdottir, Jonathan Hinton, Satoshi Hirano, Nobuyoshi Hiraoka, Katherine A. Hoadley, Asger Hobolth, Ermin Hodzic, Jessica I. Hoell, Steve Hoffmann, Oliver Hofmann, Andrea Holbrook, Aliaksei Z. Holik, Michael A. Hollingsworth, Oliver Holmes, Robert A. Holt, Chen Hong, Eun Pyo Hong, Jongwhi H. Hong, Gerrit K. Hooijer, Henrik Hornshøj, Fumie Hosoda, Yong Hou, Volker Hovestadt, William Howat, Alan P. Hoyle, Ralph H. Hruban, Jianhong Hu, Taobo Hu, Xing Hua, Kuan-lin Huang, Mei Huang, Mi Ni Huang, Vincent Huang, Yi Huang, Wolfgang Huber, Thomas J. Hudson, Michael Hummel, Jillian A. Hung, David Huntsman, Ted R. Hupp, Jason Huse, Matthew R. Huska, Barbara Hutter, Carolyn M. Hutter, Daniel Hübschmann, Christine A. Iacobuzio-Donahue, Charles David Imbusch, Marcin Imielinski, Seiya Imoto, William B. Isaacs, Keren Isaev, Shumpei Ishikawa, Murat Iskar, S. M. Ashiqul Islam, Michael Ittmann, Sinisa Ivkovic, Jose M. G. Izarzugaza, Jocelyne Jacquemier, Valerie Jakrot, Nigel B. Jamieson, Gun Ho Jang, Se Jin Jang, Joy C. Jayaseelan, Reyka Jayasinghe, Stuart R. Jefferys, Karine Jegalian, Jennifer L. Jennings, Seung-Hyup Jeon, Lara Jerman, Yuan Ji, Wei Jiao, Peter A. Johansson, Amber L. Johns, Jeremy Johns, Rory Johnson, Todd A. Johnson, Clemency Jolly, Yann Joly, Jon G. Jonasson, Corbin D. Jones, David R. Jones, David T. W. Jones, Nic Jones, Steven J. M. Jones, Jos Jonkers, Young Seok Ju, Hartmut Juhl, Jongsun Jung, Malene Juul, Randi Istrup Juul, Sissel Juul, Natalie Jäger, Rolf Kabbe, Andre Kahles, Abdullah Kahraman, Vera B. Kaiser, Hojabr Kakavand, Sangeetha Kalimuthu, Christof von Kalle, Koo Jeong Kang, Katalin Karaszi, Beth Karlan, Rosa Karlić, Dennis Karsch, Katayoon Kasaian, Karin S. Kassahn, Hitoshi Katai, Mamoru Kato, Hiroto Katoh, Yoshiiku Kawakami, Jonathan D. Kay, Stephen H. Kazakoff, Marat D. Kazanov, Maria Keays, Electron Kebebew, Richard F. Kefford, Manolis Kellis, James G. Kench, Catherine J. Kennedy, Jules N. A. Kerssemakers, David Khoo, Vincent Khoo, Narong Khuntikeo, Ekta Khurana, Helena Kilpinen, Hark Kyun Kim, Hyung-Lae Kim, Hyung-Yong Kim, Hyunghwan Kim, Jaegil Kim, Jihoon Kim, Jong K. Kim, Youngwook Kim, Tari A. King, Wolfram Klapper, Kortine Kleinheinz, Leszek J. Klimczak, Stian Knappskog, Michael Kneba, Bartha M. Knoppers, Youngil Koh, Jan Komorowski, Daisuke Komura, Mitsuhiro Komura, Gu Kong, Marcel Kool, Jan O. Korbel, Viktoriya Korchina, Andrey Korshunov, Michael Koscher, Roelof Koster, Zsofia Kote-Jarai, Antonios Koures, Milena Kovacevic, Barbara Kremeyer, Helene Kretzmer, Markus Kreuz, Savitri Krishnamurthy, Dieter Kube, Kiran Kumar, Pardeep Kumar, Sushant Kumar, Yogesh Kumar, Ritika Kundra, Kirsten Kübler, Ralf Küppers, Jesper Lagergren, Phillip H. Lai, Peter W. Laird, Sunil R. Lakhani, Christopher M. Lalansingh, Emilie Lalonde, Fabien C. Lamaze, Adam Lambert, Eric Lander, Pablo Landgraf, Luca Landoni, Anita Langerød, Andrés Lanzós, Denis Larsimont, Erik Larsson, Mark Lathrop, Loretta M. S. Lau, Chris Lawerenz, Rita T. Lawlor, Michael S. Lawrence, Alexander J. Lazar, Ana Mijalkovic Lazic, Xuan Le, Darlene Lee, Donghoon Lee, Eunjung Alice Lee, Hee Jin Lee, Jake June-Koo Lee, Jeong-Yeon Lee, Juhee Lee, Ming Ta Michael Lee, Henry Lee-Six, Kjong-Van Lehmann, Hans Lehrach, Dido Lenze, Conrad R. Leonard, Daniel A. Leongamornlert, Ignaty Leshchiner, Louis Letourneau, Ivica Letunic, Douglas A. Levine, Lora Lewis, Tim Ley, Chang Li, Constance H. Li, Haiyan Irene Li, Jun Li, Lin Li, Shantao Li, Siliang Li, Xiaobo Li, Xiaotong Li, Xinyue Li, Yilong Li, Han Liang, Sheng-Ben Liang, Peter Lichter, Pei Lin, Ziao Lin, W. M. Linehan, Ole Christian Lingjærde, Dongbing Liu, Eric Minwei Liu, Fei-Fei Fei Liu, Fenglin Liu, Jia Liu, Xingmin Liu, Julie Livingstone, Dimitri Livitz, Naomi Livni, Lucas Lochovsky, Markus Loeffler, Georgina V. Long, Armando Lopez-Guillermo, Shaoke Lou, David N. Louis, Laurence B. Lovat, Yiling Lu, Yong-Jie Lu, Youyong Lu, Claudio Luchini, Ilinca Lungu, Xuemei Luo, Hayley J. Luxton, Andy G. Lynch, Lisa Lype, Cristina López, Carlos López-Otín, Eric Z. Ma, Yussanne Ma, Gaetan MacGrogan, Shona MacRae, Geoff Macintyre, Tobias Madsen, Kazuhiro Maejima, Andrea Mafficini, Dennis T. Maglinte, Arindam Maitra, Partha P. Majumder, Luca Malcovati, Salem Malikic, Giuseppe Malleo, Graham J. Mann, Luisa Mantovani-Löffler, Kathleen Marchal, Giovanni Marchegiani, Elaine R. Mardis, Adam A. Margolin, Maximillian G. Marin, Florian Markowetz, Julia Markowski, Jeffrey Marks, Tomas Marques-Bonet, Marco A. Marra, Luke Marsden, John W. M. Martens, Sancha Martin, Jose I. Martin-Subero, Iñigo Martincorena, Alexander Martinez-Fundichely, Yosef E. Maruvka, R. Jay Mashl, Charlie E. Massie, Thomas J. Matthew, Lucy Matthews, Erik Mayer, Simon Mayes, Michael Mayo, Faridah Mbabaali, Karen McCune, Ultan McDermott, Patrick D. McGillivray, Michael D. McLellan, John D. McPherson, John R. McPherson, Treasa A. McPherson, Samuel R. Meier, Alice Meng, Shaowu Meng, Andrew Menzies, Neil D. Merrett, Sue Merson, Matthew Meyerson, William Meyerson, Piotr A. Mieczkowski, George L. Mihaiescu, Sanja Mijalkovic, Tom Mikkelsen, Michele Milella, Linda Mileshkin, Christopher A. Miller, David K. Miller, Jessica K. Miller, Gordon B. Mills, Ana Milovanovic, Sarah Minner, Marco Miotto, Gisela Mir Arnau, Lisa Mirabello, Chris Mitchell, Thomas J. Mitchell, Satoru Miyano, Naoki Miyoshi, Shinichi Mizuno, Fruzsina Molnár-Gábor, Malcolm J. Moore, Richard A. Moore, Sandro Morganella, Quaid D. Morris, Carl Morrison, Lisle E. Mose, Catherine D. Moser, Ferran Muiños, Loris Mularoni, Andrew J. Mungall, Karen Mungall, Elizabeth A. Musgrove, Ville Mustonen, David Mutch, Francesc Muyas, Donna M. Muzny, Alfonso Muñoz, Jerome Myers, Ola Myklebost, Peter Möller, Genta Nagae, Adnan M. Nagrial, Hardeep K. Nahal-Bose, Hitoshi Nakagama, Hidewaki Nakagawa, Hiromi Nakamura, Toru Nakamura, Kaoru Nakano, Tannistha Nandi, Jyoti Nangalia, Mia Nastic, Arcadi Navarro, Fabio C. P. Navarro, David E. Neal, Gerd Nettekoven, Felicity Newell, Steven J. Newhouse, Yulia Newton, Alvin Wei Tian Ng, Anthony Ng, Jonathan Nicholson, David Nicol, Yongzhan Nie, G. Petur Nielsen, Morten Muhlig Nielsen, Serena Nik-Zainal, Michael S. Noble, Katia Nones, Paul A. Northcott, Faiyaz Notta, Brian D. O’Connor, Peter O’Donnell, Maria O’Donovan, Sarah O’Meara, Brian Patrick O’Neill, J. Robert O’Neill, David Ocana, Angelica Ochoa, Layla Oesper, Christopher Ogden, Hideki Ohdan, Kazuhiro Ohi, Lucila Ohno-Machado, Karin A. Oien, Akinyemi I. Ojesina, Hidenori Ojima, Takuji Okusaka, Larsson Omberg, Choon Kiat Ong, Stephan Ossowski, German Ott, B. F. Francis Ouellette, Christine P’ng, Marta Paczkowska, Salvatore Paiella, Chawalit Pairojkul, Marina Pajic, Qiang Pan-Hammarström, Elli Papaemmanuil, Irene Papatheodorou, Nagarajan Paramasivam, Ji Wan Park, Joong-Won Park, Keunchil Park, Kiejung Park, Peter J. Park, Joel S. Parker, Simon L. Parsons, Harvey Pass, Danielle Pasternack, Alessandro Pastore, Ann-Marie Patch, Iris Pauporté, Antonio Pea, John V. Pearson, Chandra Sekhar Pedamallu, Jakob Skou Pedersen, Paolo Pederzoli, Martin Peifer, Nathan A. Pennell, Charles M. Perou, Marc D. Perry, Gloria M. Petersen, Myron Peto, Nicholas Petrelli, Robert Petryszak, Stefan M. Pfister, Mark Phillips, Oriol Pich, Hilda A. Pickett, Todd D. Pihl, Nischalan Pillay, Sarah Pinder, Mark Pinese, Andreia V. Pinho, Esa Pitkänen, Xavier Pivot, Elena Piñeiro-Yáñez, Laura Planko, Christoph Plass, Paz Polak, Tirso Pons, Irinel Popescu, Olga Potapova, Aparna Prasad, Shaun R. Preston, Manuel Prinz, Antonia L. Pritchard, Stephenie D. Prokopec, Elena Provenzano, Xose S. Puente, Sonia Puig, Montserrat Puiggròs, Sergio Pulido-Tamayo, Gulietta M. Pupo, Colin A. Purdie, Michael C. Quinn, Raquel Rabionet, Janet S. Rader, Bernhard Radlwimmer, Petar Radovic, Benjamin Raeder, Keiran M. Raine, Manasa Ramakrishna, Kamna Ramakrishnan, Suresh Ramalingam, Benjamin J. Raphael, W. Kimryn Rathmell, Tobias Rausch, Guido Reifenberger, Jüri Reimand, Jorge Reis-Filho, Victor Reuter, Iker Reyes-Salazar, Matthew A. Reyna, Sheila M. Reynolds, Esther Rheinbay, Yasser Riazalhosseini, Andrea L. Richardson, Julia Richter, Matthew Ringel, Markus Ringnér, Yasushi Rino, Karsten Rippe, Jeffrey Roach, Lewis R. Roberts, Nicola D. Roberts, Steven A. Roberts, A. Gordon Robertson, Alan J. Robertson, Javier Bartolomé Rodriguez, Bernardo Rodriguez-Martin, F. Germán Rodríguez-González, Michael H. A. Roehrl, Marius Rohde, Hirofumi Rokutan, Gilles Romieu, Ilse Rooman, Tom Roques, Daniel Rosebrock, Mara Rosenberg, Philip C. Rosenstiel, Andreas Rosenwald, Edward W. Rowe, Romina Royo, Steven G. Rozen, Yulia Rubanova, Mark A. Rubin, Carlota Rubio-Perez, Vasilisa A. Rudneva, Borislav C. Rusev, Andrea Ruzzenente, Gunnar Rätsch, Radhakrishnan Sabarinathan, Veronica Y. Sabelnykova, Sara Sadeghi, S. Cenk Sahinalp, Natalie Saini, Mihoko Saito-Adachi, Gordon Saksena, Adriana Salcedo, Roberto Salgado, Leonidas Salichos, Richard Sallari, Charles Saller, Roberto Salvia, Michelle Sam, Jaswinder S. Samra, Francisco Sanchez-Vega, Chris Sander, Grant Sanders, Rajiv Sarin, Iman Sarrafi, Aya Sasaki-Oku, Torill Sauer, Guido Sauter, Robyn P. M. Saw, Maria Scardoni, Christopher J. Scarlett, Aldo Scarpa, Ghislaine Scelo, Dirk Schadendorf, Jacqueline E. Schein, Markus B. Schilhabel, Matthias Schlesner, Thorsten Schlomm, Heather K. Schmidt, Sarah-Jane Schramm, Stefan Schreiber, Nikolaus Schultz, Steven E. Schumacher, Roland F. Schwarz, Richard A. Scolyer, David Scott, Ralph Scully, Raja Seethala, Ayellet V. Segre, Iris Selander, Colin A. Semple, Yasin Senbabaoglu, Subhajit Sengupta, Elisabetta Sereni, Stefano Serra, Dennis C. Sgroi, Mark Shackleton, Nimish C. Shah, Sagedeh Shahabi, Catherine A. Shang, Ping Shang, Ofer Shapira, Troy Shelton, Ciyue Shen, Hui Shen, Rebecca Shepherd, Ruian Shi, Yan Shi, Yu-Jia Shiah, Tatsuhiro Shibata, Juliann Shih, Eigo Shimizu, Kiyo Shimizu, Seung Jun Shin, Yuichi Shiraishi, Tal Shmaya, Ilya Shmulevich, Solomon I. Shorser, Charles Short, Raunak Shrestha, Suyash S. Shringarpure, Craig Shriver, Shimin Shuai, Nikos Sidiropoulos, Reiner Siebert, Anieta M. Sieuwerts, Lina Sieverling, Sabina Signoretti, Katarzyna O. Sikora, Michele Simbolo, Ronald Simon, Janae V. Simons, Jared T. Simpson, Peter T. Simpson, Samuel Singer, Nasa Sinnott-Armstrong, Payal Sipahimalani, Tara J. Skelly, Marcel Smid, Jaclyn Smith, Karen Smith-McCune, Nicholas D. Socci, Heidi J. Sofia, Matthew G. Soloway, Lei Song, Anil K. Sood, Sharmila Sothi, Christos Sotiriou, Cameron M. Soulette, Paul N. Span, Paul T. Spellman, Nicola Sperandio, Andrew J. Spillane, Oliver Spiro, Jonathan Spring, Johan Staaf, Peter F. Stadler, Peter Staib, Stefan G. Stark, Lucy Stebbings, Ólafur Andri Stefánsson, Oliver Stegle, Lincoln D. Stein, Alasdair Stenhouse, Chip Stewart, Stephan Stilgenbauer, Miranda D. Stobbe, Michael R. Stratton, Jonathan R. Stretch, Adam J. Struck, Joshua M. Stuart, Henk G. Stunnenberg, Hong Su, Xiaoping Su, Ren X. Sun, Stephanie Sungalee, Hana Susak, Akihiro Suzuki, Fred Sweep, Monika Szczepanowski, Holger Sültmann, Takashi Yugawa, Angela Tam, David Tamborero, Benita Kiat Tee Tan, Donghui Tan, Patrick Tan, Hiroko Tanaka, Hirokazu Taniguchi, Tomas J. Tanskanen, Maxime Tarabichi, Roy Tarnuzzer, Patrick Tarpey, Morgan L. Taschuk, Kenji Tatsuno, Simon Tavaré, Darrin F. Taylor, Amaro Taylor-Weiner, Jon W. Teague, Bin Tean Teh, Varsha Tembe, Javier Temes, Kevin Thai, Sarah P. Thayer, Nina Thiessen, Gilles Thomas, Sarah Thomas, Alan Thompson, Alastair M. Thompson, John F. F. Thompson, R. Houston Thompson, Heather Thorne, Leigh B. Thorne, Adrian Thorogood, Grace Tiao, Nebojsa Tijanic, Lee E. Timms, Roberto Tirabosco, Marta Tojo, Stefania Tommasi, Christopher W. Toon, Umut H. Toprak, David Torrents, Giampaolo Tortora, Jörg Tost, Yasushi Totoki, David Townend, Nadia Traficante, Isabelle Treilleux, Jean-Rémi Trotta, Lorenz H. P. Trümper, Ming Tsao, Tatsuhiko Tsunoda, Jose M. C. Tubio, Olga Tucker, Richard Turkington, Daniel J. Turner, Andrew Tutt, Masaki Ueno, Naoto T. Ueno, Christopher Umbricht, Husen M. Umer, Timothy J. Underwood, Lara Urban, Tomoko Urushidate, Tetsuo Ushiku, Liis Uusküla-Reimand, Alfonso Valencia, David J. Van Den Berg, Steven Van Laere, Peter Van Loo, Erwin G. Van Meir, Gert G. Van den Eynden, Theodorus Van der Kwast, Naveen Vasudev, Miguel Vazquez, Ravikiran Vedururu, Umadevi Veluvolu, Shankar Vembu, Lieven P. C. Verbeke, Peter Vermeulen, Clare Verrill, Alain Viari, David Vicente, Caterina Vicentini, K. VijayRaghavan, Juris Viksna, Ricardo E. Vilain, Izar Villasante, Anne Vincent-Salomon, Tapio Visakorpi, Douglas Voet, Paresh Vyas, Ignacio Vázquez-García, Nick M. Waddell, Nicola Waddell, Claes Wadelius, Lina Wadi, Rabea Wagener, Jeremiah A. Wala, Jian Wang, Jiayin Wang, Linghua Wang, Qi Wang, Wenyi Wang, Yumeng Wang, Zhining Wang, Paul M. Waring, Hans-Jörg Warnatz, Jonathan Warrell, Anne Y. Warren, Sebastian M. Waszak, David C. Wedge, Dieter Weichenhan, Paul Weinberger, John N. Weinstein, Joachim Weischenfeldt, Daniel J. Weisenberger, Ian Welch, Michael C. Wendl, Johannes Werner, Justin P. Whalley, David A. Wheeler, Hayley C. Whitaker, Dennis Wigle, Matthew D. Wilkerson, Ashley Williams, James S. Wilmott, Gavin W. Wilson, Julie M. Wilson, Richard K. Wilson, Boris Winterhoff, Jeffrey A. Wintersinger, Maciej Wiznerowicz, Stephan Wolf, Bernice H. Wong, Tina Wong, Winghing Wong, Youngchoon Woo, Scott Wood, Bradly G. Wouters, Adam J. Wright, Derek W. Wright, Mark H. Wright, Chin-Lee Wu, Dai-Ying Wu, Guanming Wu, Jianmin Wu, Kui Wu, Yang Wu, Zhenggang Wu, Liu Xi, Tian Xia, Qian Xiang, Xiao Xiao, Rui Xing, Heng Xiong, Qinying Xu, Yanxun Xu, Hong Xue, Shinichi Yachida, Sergei Yakneen, Rui Yamaguchi, Takafumi N. Yamaguchi, Masakazu Yamamoto, Shogo Yamamoto, Hiroki Yamaue, Fan Yang, Huanming Yang, Jean Y. Yang, Liming Yang, Lixing Yang, Shanlin Yang, Tsun-Po Yang, Yang Yang, Xiaotong Yao, Marie-Laure Yaspo, Lucy Yates, Christina Yau, Chen Ye, Kai Ye, Venkata D. Yellapantula, Christopher J. Yoon, Sung-Soo Yoon, Fouad Yousif, Jun Yu, Kaixian Yu, Willie Yu, Yingyan Yu, Ke Yuan, Yuan Yuan, Denis Yuen, Christina K. Yung, Olga Zaikova, Jorge Zamora, Marc Zapatka, Jean C. Zenklusen, Thorsten Zenz, Nikolajs Zeps, Cheng-Zhong Zhang, Fan Zhang, Hailei Zhang, Hongwei Zhang, Hongxin Zhang, Jiashan Zhang, Jing Zhang, Junjun Zhang, Xiuqing Zhang, Xuanping Zhang, Yan Zhang, Zemin Zhang, Zhongming Zhao, Liangtao Zheng, Xiuqing Zheng, Wanding Zhou, Yong Zhou, Bin Zhu, Hongtu Zhu, Jingchun Zhu, Shida Zhu, Lihua Zou, Xueqing Zou, Anna deFazio, Nicholas van As, Carolien H. M. van Deurzen, Marc J. van de Vijver, L. van’t Veer, Christian von Mering

**Affiliations:** 1 The Broad Institute of MIT and Harvard, Cambridge, MA USA; 2 Center for Cancer Research, Massachusetts General Hospital, Charlestown, MA USA; 3 Harvard Medical School, Boston, MA USA; 4 Department of Molecular Medicine (MOMA), Aarhus University Hospital, Aarhus, Denmark; 5 Wellcome Trust Sanger Institute, Hinxton, UK; 6 Bioinformatics and Integrative Genomics, Harvard University, Cambridge, MA USA; 7 Department of Cancer Biology, Dana-Farber Cancer Institute, Boston, MA USA; 8 Harvard University, Cambridge, MA USA; 9 Division of Applied Bioinformatics, German Cancer Research Center (DKFZ), Heidelberg, Germany; 10 Institute for Research in Biomedicine (IRB Barcelona), The Barcelona Institute of Science and Technology, Barcelona, Spain; 11 Research Program on Biomedical Informatics, Universitat Pompeu Fabra, Barcelona, Spain; 12 Computational Biology Program, Ontario Institute for Cancer Research, Toronto, Ontario Canada; 13 Ontario Institute for Cancer Research, Toronto, Ontario Canada; 14 Department for BioMedical Research, University of Bern, Bern, Switzerland; 15 Graduate School of Cellular and Biomedical Sciences, University of Bern, Bern, Switzerland; 16 Department of Medical Oncology, Bern University Hospital, University of Bern, Bern, Switzerland; 17 Division of Theoretical Bioinformatics, German Cancer Research Center (DKFZ), Heidelberg, Germany; 18 Bioquant Center, Institute of Pharmacy and Molecular Biotechnology, University of Heidelberg, Heidelberg, Germany; 19 Department of Cell Biology, Harvard Medical School, Boston, MA USA; 20 cBio Center, Dana-Farber Cancer Institute, Harvard Medical School, Boston, MA USA; 21 Department of Genomic Medicine, University of Texas MD Anderson Cancer Center, Houston, TX USA; 22 Graduate Program in Structural and Computational Biology and Molecular Biophysics, Baylor College of Medicine, Houston, TX USA; 23 Laboratory for Medical Science Mathematics, RIKEN Center for Integrative Medical Sciences, Yokohama, Japan; 24 Department of Genitourinary Medical Oncology - Research, Division of Cancer Medicine, University of Texas MD Anderson Cancer Center, Houston, TX USA; 25 Department of Urology, Icahn school of Medicine at Mount Sinai, New York, NY USA; 26 Faculty of Biosciences, Heidelberg University, Heidelberg, Germany; 27 Department of Radiation Oncology, Harvard Medical School, Massachusetts General Hospital, Boston, MA USA; 28 Department of Physiology and Biophysics, Weill Cornell Medicine, New York, NY USA; 29 Institute for Computational Biomedicine, Weill Cornell Medicine, New York, NY USA; 30 Science for Life Laboratory, Department of Cell and Molecular Biology, Uppsala University Uppsala, Sweden; 31 European Bioinformatics Institute, European Molecular Biology Laboratory, Hinxton, UK; 32 Bioinformatics Research Centre (BiRC), Aarhus University, Aarhus, Denmark; 33 Department of Molecular and Cellular Biology, Baylor College of Medicine, Houston, TX USA; 34 Department of Medical Biophysics, University of Toronto, Toronto, Ontario Canada; 35 Division of Computational Biology, Memorial Sloan Kettering Cancer Center, New York, NY USA; 36 Institute of Molecular Life Sciences and Swiss Institute of Bioinformatics, University of Zurich, Zurich, Switzerland; 37 Samsung Medical Center, Sungkyunkwan University School of Medicine, Seoul, South Korea; 38 Institute of Computer Science, Polish Academy of Sciences, Warsaw, Poland; 39 Program in Computational Biology and Bioinformatics, Yale University, New Haven, CT USA; 40 SBGD Inc, Cambridge, MA USA; 41 Department of Haematology, University of Cambridge, Cambridge, UK; 42 Department of Molecular Biophysics and Biochemistry, Yale University, New Haven, CT USA; 43 Biotech Research & Innovation Centre (BRIC), The Finsen Laboratory, Rigshospitalet, University of Copenhagen, Copenhagen, Denmark; 44 Department of Genetics, Stanford University School of Medicine, Stanford, CA USA; 45 Department of Zoology, Genetics and Physical Anthropology, Universidade de Santiago de Compostela, Santiago de Compostela, Spain; 46 Centre for Research in Molecular Medicine and Chronic Diseases (CIMUS), Universidade de Santiago de Compostela, Santiago de Compostela, Spain; 47 The Biomedical Research Centre (CINBIO), Universidade de Vigo, Vigo, Spain; 48 Department of Oncology-Pathology, Science for Life Laboratory, Karolinska Institute, Stockholm, Sweden; 49 Genetics and Genome Biology Program, SickKids Research Institute, Toronto, Ontario Canada; 50 Department of Gene Technology, Tallinn University of Technology, Tallinn, Estonia; 51 Science for Life Laboratory, Department of Immunology, Genetics and Pathology, Uppsala University, Uppsala, Sweden; 52 New York Genome Center, New York, NY USA; 53 Department of Biostatistics and Computational Biology, Dana-Farber Cancer Institute, Boston, MA USA; 54 Department of Biomedical Informatics, Harvard Medical School, Boston, MA USA; 55 Department of Biology and Rosenstiel Basic Medical Sciences Research Center, Brandeis University, Waltham, MA USA; 56 Department of Pathology and Laboratory Medicine, and Englander Institute for Precision Medicine, and Institute for Computational Biomedicine, and Meyer Cancer Center, Weill Cornell Medicine, New York, NY USA; 57 MIT Computer Science and Artificial Intelligence Laboratory, Cambridge, MA USA; 58 Laboratory for Cancer Genomics, RIKEN Center for Integrative Medical Sciences, Tokyo, Japan; 59 Department of Computer Science, Princeton University, Princeton, NJ USA; 60 Department of Pathology and Laboratory Medicine, Weill Cornell Medicine, New York, NY USA; 61 Englander Institute for Precision Medicine, Weill Cornell Medicine, New York, NY USA; 62 Meyer Cancer Center, Weill Cornell Medicine, New York, NY USA; 63 Center for Biomolecular Science and Engineering, University of California at Santa Cruz, Santa Cruz, CA USA; 64 Department of Medical Science Mathematics, Medical Research Institute, Tokyo Medical and Dental University, Tokyo, Japan; 65 Laboratory for Medical Science Mathematics, Department of Biological Sciences, Graduate School of Science, The University of Tokyo, Tokyo, Japan; 66 Human Genome Sequencing Center, Baylor College of Medicine, Houston, TX USA; 67 Department of Computer Science, Yale University, New Haven, CT USA; 68 Catalan Institution for Research and Advanced Studies (ICREA), Barcelona, Spain; 70 Genome Biology Unit, European Molecular Biology Laboratory (EMBL), Heidelberg, Germany; 71 Department of Medical Oncology, Dana-Farber Cancer Institute, Boston, MA USA; 72 Department of Pathology, Massachusetts General Hospital, Boston, MA USA; 76 Department of Molecular Genetics, University of Toronto, Toronto, Ontario Canada; 77 Computational Biology Program, Ontario Institute for Cancer Research, Toronto, Ontario Canada; 78 Technical University of Denmark, Lyngby, Denmark; 79 University of Copenhagen, Copenhagen, Denmark; 80 University of Texas MD Anderson Cancer Center, Houston, TX USA; 81 Korea Advanced Institute of Science and Technology, Daejeon, South Korea; 82 Institute for Research in Biomedicine (IRB Barcelona), The Barcelona Institute of Science and Technology, Barcelona, Spain; 83 Research Program on Biomedical Informatics, Universitat Pompeu Fabra, Barcelona, Spain; 84 Barcelona Supercomputing Center (BSC), Barcelona, Spain; 85 Queensland Centre for Medical Genomics, Institute for Molecular Bioscience, The University of Queensland, St Lucia, Queensland Australia; 86 University of Milano Bicocca, Monza, Italy; 87 Peter MacCallum Cancer Centre, Melbourne, Victoria Australia; 88 Sir Peter MacCallum Department of Oncology, The University of Melbourne, Melbourne, Victoria Australia; 89 CNAG-CRG, Centre for Genomic Regulation (CRG), Barcelona Institute of Science and Technology (BIST), Barcelona, Spain; 90 Universitat Pompeu Fabra, Barcelona, Spain; 91 Biomolecular Engineering Department, University of California, Santa Cruz, Santa Cruz, CA USA; 92 Program in Computational Biology and Bioinformatics, Yale University, New Haven, CT USA; 93 Center for Precision Health, School of Biomedical Informatics, University of Texas Health Science Center, Houston, TX USA; 94 The Donnelly Centre, University of Toronto, Toronto, Ontario Canada; 95 Simon Fraser University, Burnaby, British Columbia Canada; 96 Research Core Center, National Cancer Centre Korea, Goyang-si, South Korea; 97 European Molecular Biology Laboratory, European Bioinformatics Institute (EMBL-EBI), Hinxton, Cambridge UK; 98 Genome Biology Unit, European Molecular Biology Laboratory (EMBL), Heidelberg, Germany; 99 Institute of Biomedicine, Sahlgrenska Academy at University of Gothenburg, Gothenburg, Sweden; 100 Department of Molecular Biophysics and Biochemistry, Yale University, New Haven, CT USA; 101 Department of Information Technology, Ghent University, Ghent, Belgium; 102 Department of Plant Biotechnology and Bioinformatics, Ghent University, Ghent, Belgium; 103 Department of Physiology and Biophysics, Weill Cornell Medicine, New York, NY USA; 104 Institute for Computational Biomedicine, Weill Cornell Medicine, New York, NY USA; 105 Englander Institute for Precision Medicine, Weill Cornell Medicine, New York, NY USA; 106 Yale School of Medicine, Yale University, New Haven, CT USA; 107 Institute for Research in Biomedicine (IRB Barcelona), The Barcelona Institute of Science and Technology, Barcelona, Spain; 108 Cheonan Industry-Academic Collaboration Foundation, Sangmyung University, Cheonan, South Korea; 109 Spanish National Cancer Research Centre, Madrid, Spain; 110 Department of Computer Science, Princeton University, Princeton, NJ USA; 111 Vall d’Hebron Institute of Oncology (VHIO), Barcelona, Spain; 112 Vancouver Prostate Centre, Vancouver, British Columbia Canada; 113 Indiana University, Bloomington, IN USA; 114 Peter MacCallum Cancer Centre, University of Melbourne, Melbourne, Victoria Australia; 115 Institució Catalana de Recerca i Estudis Avançats (ICREA), Barcelona, Spain; 116 Department of Clinical and Molecular Medicine, Faculty of Medicine and Health Sciences, Norwegian University of Science and Technology, Trondheim, Norway; 117 Department of Information Technology, Interuniversitair Micro-Electronica Centrum (IMEC), Ghent University, Ghent, Belgium; 118 The McDonnell Genome Institute, Washington University, St Louis, MO USA; 119 School of Computer Science and Technology, Xian Jiaotong University, Xian, China; 120 School of Electronic and Information Engineering, Xian Jiaotong University, Xian, China; 121 Oregon Health & Sciences University, Portland, OR USA; 122 Department of Medicine and Therapeutics, The Chinese University of Hong Kong, Shatin, Hong Kong China; 123 The University of Texas Health Science Center at Houston, Houston, TX USA; 124 Department of Biomedical Informatics, College of Medicine, The Ohio State University, Columbus, OH USA; 125 The Ohio State University Comprehensive Cancer Center (OSUCCC – James), Columbus, OH USA; 126 School of Biomedical Informatics, The University of Texas Health Science Center at Houston, Houston, TX USA; 127 Department of Biochemistry and Molecular Genetics, Feinberg School of Medicine, Northwestern University, Chicago, IL USA; 128 Department of Zoology, Genetics and Physical Anthropology, Universidade de Santiago de Compostela, Santiago de Compostela, Spain; 129 Centre for Research in Molecular Medicine and Chronic Diseases (CIMUS), Universidade de Santiago de Compostela, Santiago de Compostela, Spain; 130 The Biomedical Research Centre (CINBIO), Universidade de Vigo, Vigo, Spain; 131 Transmissible Cancer Group, Department of Veterinary Medicine, University of Cambridge, Cambridge, UK; 132 Department of Medical Biophysics, University of Toronto, Toronto, Ontario Canada; 133 Department of Pharmacology, University of Toronto, Toronto, Ontario Canada; 134 University of California Los Angeles, Los Angeles, CA USA; 135 Sir Peter MacCallum Department of Oncology, University of Melbourne, Melbourne, Victoria Australia; 136 National Center for Tumor Diseases (NCT) Heidelberg, Heidelberg, Germany; 137 Division of Applied Bioinformatics, German Cancer Research Center (DKFZ), Heidelberg, Germany; 138 German Cancer Genome Consortium (DKTK), Heidelberg, Germany; 139 Johns Hopkins School of Medicine, Baltimore, MD USA; 140 Department of Biochemistry, Microbiology and Immunology, Faculty of Medicine, University of Ottawa, Ottawa, Ontario Canada; 141 Centre for Molecular Science Informatics, Department of Chemistry, University of Cambridge, Cambridge, UK; 142 Department of Biomedical Informatics, Harvard Medical School, Boston, MA USA; 143 Ludwig Center, Harvard Medical School, Boston, MA USA; 144 Broad Institute of MIT and Harvard, Cambridge, MA USA; 145 Cancer Research UK Cambridge Institute, University of Cambridge, Cambridge, UK; 146 University of Cambridge, Cambridge, UK; 147 Centre for Genomic Regulation (CRG), The Barcelona Institute of Science and Technology, Barcelona, Spain; 148 Quantitative Genomics Laboratories (qGenomics), Barcelona, Spain; 149 The Azrieli Faculty of Medicine, Bar-Ilan University, Safed, Israel; 150 Genome Integrity and Structural Biology Laboratory, National Institute of Environmental Health Sciences (NIEHS), Durham, NC USA; 151 German Cancer Consortium (DKTK), Heidelberg, Germany; 152 Heidelberg Center for Personalized Oncology (DKFZ-HIPO), German Cancer Research Center (DKFZ), Heidelberg, Germany; 153 Hopp Childrens Cancer Center (KiTZ), Heidelberg, Germany; 154 Pediatric Glioma Research Group, German Cancer Research Center (DKFZ), Heidelberg, Germany; 155 Wellcome Sanger Institute, Hinxton, UK; 156 Skolkovo Institute of Science and Technology, Moscow, Russia; 157 A. A. Kharkevich Institute of Information Transmission Problems, Moscow, Russia; 158 Dmitry Rogachev National Research Center of Pediatric Hematology, Oncology and Immunology, Moscow, Russia; 159 Integrative Bioinformatics Support Group, National Institute of Environmental Health Sciences (NIEHS), Durham, NC USA; 160 Center For Medical Innovation, Seoul National University Hospital, Seoul, South Korea; 161 Department of Internal Medicine, Seoul National University Hospital, Seoul, South Korea; 162 Division of Genetics and Genomics, Boston Children’s Hospital and Harvard Medical School, Boston, MA USA; 163 Ludwig Center at Harvard, Boston, MA USA; 164 School of Medicine and School of Mathematics and Statistics, University of St Andrews, St Andrews, UK; 165 Harvard Medical School, Boston, MA USA; 166 Dana-Farber Cancer Institute, Boston, MA USA; 167 Department of Medical Oncology, University Hospital, University of Bern, Bern, Switzerland; 168 Department of Pathology, The University of Melbourne, Melbourne, Victoria Australia; 169 The Institute of Medical Science, The University of Tokyo, Tokyo, Japan; 170 Institute of Medical Genetics and Applied Genomics, University of Tübingen, Tübingen, Germany; 171 Department of Genetics and Computational Biology, QIMR Berghofer Medical Research Institute, Brisbane, Queensland Australia; 172 Institute for Molecular Bioscience, University of Queensland, St Lucia, Brisbane, Queensland Australia; 173 German Cancer Research Center (DKFZ), Heidelberg, Germany; 174 Center for Reproductive Biology, School of Molecular Biosciences, Washington State University, Pullman, WA USA; 175 Cancer Research Institute, Beth Israel Deaconess Medical Center, Boston, MA USA; 176 Ben May Department for Cancer Research, Department of Human Genetics, The University of Chicago, Chicago, IL USA; 200 Applied Tumor Genomics Research Program, Research Programs Unit, University of Helsinki, Helsinki, Finland; 201 Wellcome Sanger Institute, Wellcome Genome Campus, Hinxton, UK; 202 Memorial Sloan Kettering Cancer Center, New York, NY USA; 203 Genome Science Division, Research Center for Advanced Science and Technology, University of Tokyo, Tokyo, Japan; 204 Department of Surgery, University of Chicago, Chicago, IL USA; 205 Department of Surgery, Division of Hepatobiliary and Pancreatic Surgery, School of Medicine, Keimyung University Dongsan Medical Center, Daegu, South Korea; 206 Department of Oncology, Gil Medical Center, Gachon University, Incheon, South Korea; 207 Hiroshima University, Hiroshima, Japan; 208 Department of Bioinformatics and Computational Biology, The University of Texas MD Anderson Cancer Center, Houston, TX USA; 209 University of Texas MD Anderson Cancer Center, Houston, TX USA; 210 King Faisal Specialist Hospital and Research Centre, Al Maather, Riyadh, Saudi Arabia; 211 Bioinformatics Unit, Spanish National Cancer Research Centre (CNIO), Madrid, Spain; 212 Bioinformatics Core Facility, University Medical Center Hamburg, Hamburg, Germany; 213 Heinrich Pette Institute, Leibniz Institute for Experimental Virology, Hamburg, Germany; 214 Ontario Tumour Bank, Ontario Institute for Cancer Research, Toronto, ON Canada; 215 Department of Pathology, The University of Texas MD Anderson Cancer Center, Houston, TX USA; 216 Laboratory of Pathology, Center for Cancer Research, National Cancer Institute, Bethesda, MD USA; 217 Department of Cellular and Molecular Medicine and Department of Bioengineering, University of California San Diego, La Jolla, CA USA; 218 UC San Diego Moores Cancer Center, San Diego, CA USA; 219 Canada’s Michael Smith Genome Sciences Centre, BC Cancer, Vancouver, BC Canada; 220 Sir Peter MacCallum Department of Oncology, Peter MacCallum Cancer Centre, University of Melbourne, Melbourne, VIC Australia; 221 Centre for Research in Molecular Medicine and Chronic Diseases (CiMUS), Universidade de Santiago de Compostela, Santiago de Compostela, Spain; 222 Department of Zoology, Genetics and Physical Anthropology, (CiMUS), Universidade de Santiago de Compostela, Santiago de Compostela, Spain; 223 The Biomedical Research Centre (CINBIO), Universidade de Vigo, Vigo, Spain; 224 Royal National Orthopaedic Hospital - Bolsover, London, UK; 225 Department of Genomic Medicine, The University of Texas MD Anderson Cancer Center, Houston, TX USA; 226 Quantitative and Computational Biosciences Graduate Program, Baylor College of Medicine, Houston, TX USA; 227 The Jackson Laboratory for Genomic Medicine, Farmington, CT USA; 228 Genome Informatics Program, Ontario Institute for Cancer Research, Toronto, ON Canada; 229 Institute of Human Genetics, Christian-Albrechts-University, Kiel, Germany; 230 Institute of Human Genetics, Ulm University and Ulm University Medical Center, Ulm, Germany; 231 Queensland Centre for Medical Genomics, Institute for Molecular Bioscience, University of Queensland, St. Lucia, Brisbane, QLD Australia; 232 Salford Royal NHS Foundation Trust, Salford, UK; 233 Department of Surgery, Pancreas Institute, University and Hospital Trust of Verona, Verona, Italy; 234 Molecular and Medical Genetics, OHSU Knight Cancer Institute, Oregon Health and Science University, Portland, OR USA; 235 Department of Molecular Oncology, BC Cancer Research Centre, Vancouver, BC Canada; 236 The McDonnell Genome Institute at Washington University, St. Louis, MO USA; 237 University College London, London, UK; 238 Division of Cancer Genomics, National Cancer Center Research Institute, National Cancer Center, Tokyo, Japan; 239 DLR Project Management Agency, Bonn, Germany; 240 Tokyo Women’s Medical University, Tokyo, Japan; 241 Center for Molecular Oncology, Memorial Sloan Kettering Cancer Center, New York, NY USA; 242 Los Alamos National Laboratory, Los Alamos, NM USA; 243 Department of Pathology, University Health Network, Toronto General Hospital, Toronto, ON Canada; 244 Nottingham University Hospitals NHS Trust, Nottingham, UK; 245 Epigenomics and Cancer Risk Factors, German Cancer Research Center (DKFZ), Heidelberg, Germany; 246 Computational Biology Program, Ontario Institute for Cancer Research, Toronto, ON Canada; 247 Department of Molecular Genetics, University of Toronto, Toronto, ON Canada; 248 Vector Institute, Toronto, ON Canada; 249 Hematopathology Section, Institute of Pathology, Christian-Albrechts-University, Kiel, Germany; 250 Department of Pathology and Laboratory Medicine, School of Medicine, University of North Carolina at Chapel Hill, Chapel Hill, NC USA; 251 Department of Cancer Genetics, Institute for Cancer Research, Oslo University Hospital, The Norwegian Radium Hospital, Oslo, Norway; 252 Pathology, Hospital Clinic, Institut d’Investigacions Biomèdiques August Pi i Sunyer (IDIBAPS), University of Barcelona, Barcelona, Spain; 253 Department of Veterinary Medicine, Transmissible Cancer Group, University of Cambridge, Cambridge, UK; 254 Alvin J. Siteman Cancer Center, Washington University School of Medicine, St. Louis, MO USA; 255 Wolfson Wohl Cancer Research Centre, Institute of Cancer Sciences, University of Glasgow, Glasgow, UK; 256 Lineberger Comprehensive Cancer Center, University of North Carolina at Chapel Hill, Chapel Hill, NC USA; 257 Broad Institute of MIT and Harvard, Cambridge, MA USA; 258 Dana-Farber/Boston Children’s Cancer and Blood Disorders Center, Boston, MA USA; 259 Department of Pediatrics, Harvard Medical School, Boston, MA USA; 260 Leeds Institute of Medical Research @ St. James’s, University of Leeds, St. James’s University Hospital, Leeds, UK; 261 Department of Pathology and Diagnostics, University and Hospital Trust of Verona, Verona, Italy; 262 Department of Surgery, Princess Alexandra Hospital, Brisbane, QLD Australia; 263 Surgical Oncology Group, Diamantina Institute, University of Queensland, Brisbane, QLD Australia; 264 Department of Population and Quantitative Health Sciences, Case Western Reserve University School of Medicine, Cleveland, OH USA; 265 Research Health Analytics and Informatics, University Hospitals Cleveland Medical Center, Cleveland, OH USA; 266 Gloucester Royal Hospital, Gloucester, UK; 267 European Molecular Biology Laboratory, European Bioinformatics Institute (EMBL-EBI), Cambridge, UK; 268 Diagnostic Development, Ontario Institute for Cancer Research, Toronto, ON Canada; 269 Barcelona Supercomputing Center (BSC), Barcelona, Spain; 270 Arnie Charbonneau Cancer Institute, University of Calgary, Calgary, AB Canada; 271 Departments of Surgery and Oncology, University of Calgary, Calgary, AB Canada; 272 Department of Pathology, Oslo University Hospital, The Norwegian Radium Hospital, Oslo, Norway; 273 PanCuRx Translational Research Initiative, Ontario Institute for Cancer Research, Toronto, ON Canada; 274 Department of Oncology, Sidney Kimmel Comprehensive Cancer Center at Johns Hopkins University School of Medicine, Baltimore, MD USA; 275 University Hospital Southampton NHS Foundation Trust, Southampton, UK; 276 Royal Stoke University Hospital, Stoke-on-Trent, UK; 277 Genome Sequence Informatics, Ontario Institute for Cancer Research, Toronto, ON Canada; 278 Human Longevity Inc, San Diego, CA USA; 279 Olivia Newton-John Cancer Research Institute, La Trobe University, Heidelberg, VIC Australia; 280 Computer Network Information Center, Chinese Academy of Sciences, Beijing, China; 281 Genome Canada, Ottawa, ON Canada; 282 CNAG-CRG, Centre for Genomic Regulation (CRG), Barcelona Institute of Science and Technology (BIST), Barcelona, Spain; 283 Universitat Pompeu Fabra (UPF), Barcelona, Spain; 284 Buck Institute for Research on Aging, Novato, CA USA; 285 Duke University Medical Center, Durham, NC USA; 286 Department of Human Genetics, Hannover Medical School, Hannover, Germany; 287 Center for Bioinformatics and Functional Genomics, Cedars-Sinai Medical Center, Los Angeles, CA USA; 288 Department of Biomedical Sciences, Cedars-Sinai Medical Center, Los Angeles, CA USA; 289 The Hebrew University Faculty of Medicine, Jerusalem, Israel; 290 Barts Cancer Institute, Barts and the London School of Medicine and Dentistry, Queen Mary University of London, London, UK; 291 Department of Computer Science, Bioinformatics Group, University of Leipzig, Leipzig, Germany; 292 Interdisciplinary Center for Bioinformatics, University of Leipzig, Leipzig, Germany; 293 Transcriptome Bioinformatics, LIFE Research Center for Civilization Diseases, University of Leipzig, Leipzig, Germany; 294 Department of Medical Oncology, Dana-Farber Cancer Institute, Boston, MA USA; 295 Department of Cancer Biology, Dana-Farber Cancer Institute, Boston, MA USA; 296 Harvard Medical School, Boston, MA USA; 297 USC Norris Comprehensive Cancer Center, University of Southern California, Los Angeles, CA USA; 298 Department of Diagnostics and Public Health, University and Hospital Trust of Verona, Verona, Italy; 299 Department of Mathematics, Aarhus University, Aarhus, Denmark; 300 Department of Molecular Medicine (MOMA), Aarhus University Hospital, Aarhus N, Denmark; 301 Instituto Carlos Slim de la Salud, Mexico City, Mexico; 302 Department of Medical Biophysics, University of Toronto, Toronto, ON Canada; 303 Cancer Division, Garvan Institute of Medical Research, Kinghorn Cancer Centre, University of New South Wales (UNSW Sydney), Sydney, NSW Australia; 304 South Western Sydney Clinical School, Faculty of Medicine, University of New South Wales (UNSW Sydney), Liverpool, NSW Australia; 305 West of Scotland Pancreatic Unit, Glasgow Royal Infirmary, Glasgow, UK; 306 Center for Digital Health, Berlin Institute of Health and Charitè - Universitätsmedizin Berlin, Berlin, Germany; 307 Heidelberg Center for Personalized Oncology (DKFZ-HIPO), German Cancer Research Center (DKFZ), Heidelberg, Germany; 308 The Preston Robert Tisch Brain Tumor Center, Duke University Medical Center, Durham, NC USA; 309 Massachusetts General Hospital, Boston, MA USA; 310 National Institute of Biomedical Genomics, Kalyani, West Bengal India; 311 Institute of Clinical Medicine and Institute of Oral Biology, University of Oslo, Oslo, Norway; 312 University of North Carolina at Chapel Hill, Chapel Hill, NC USA; 313 ARC-Net Centre for Applied Research on Cancer, University and Hospital Trust of Verona, Verona, Italy; 314 The Institute of Cancer Research, London, UK; 315 Centre for Computational Biology, Duke-NUS Medical School, Singapore, Singapore; 316 Programme in Cancer and Stem Cell Biology, Duke-NUS Medical School, Singapore, Singapore; 317 Division of Oncology and Pathology, Department of Clinical Sciences Lund, Lund University, Lund, Sweden; 318 Department of Pediatric Oncology, Hematology and Clinical Immunology, Heinrich-Heine-University, Düsseldorf, Germany; 319 Laboratory for Medical Science Mathematics, RIKEN Center for Integrative Medical Sciences, Yokohama, Japan; 320 RIKEN Center for Integrative Medical Sciences, Yokohama, Japan; 321 Department of Internal Medicine/Hematology, Friedrich-Ebert-Hospital, Neumünster, Germany; 322 Departments of Dermatology and Pathology, Yale University, New Haven, CT USA; 323 Centre for Genomic Regulation (CRG), The Barcelona Institute of Science and Technology, Barcelona, Spain; 324 Radcliffe Department of Medicine, University of Oxford, Oxford, UK; 325 Canadian Center for Computational Genomics, McGill University, Montreal, QC Canada; 326 Department of Human Genetics, McGill University, Montreal, QC Canada; 327 Department of Human Genetics, University of California Los Angeles, Los Angeles, CA USA; 328 Department of Pharmacology, University of Toronto, Toronto, ON Canada; 329 Faculty of Medicine and Health Technology, Tampere University and Tays Cancer Center, Tampere University Hospital, Tampere, Finland; 330 Haematology, Leeds Teaching Hospitals NHS Trust, Leeds, UK; 331 Translational Research and Innovation, Centre Léon Bérard, Lyon, France; 332 Fox Chase Cancer Center, Philadelphia, PA USA; 333 International Agency for Research on Cancer, World Health Organization, Lyon, France; 334 Earlham Institute, Norwich, UK; 335 Norwich Medical School, University of East Anglia, Norwich, UK; 336 Department of Molecular Biology, Faculty of Science, Radboud Institute for Molecular Life Sciences, Radboud University, Nijmegen, HB The Netherlands; 337 CRUK Manchester Institute and Centre, Manchester, UK; 338 Department of Radiation Oncology, University of Toronto, Toronto, ON Canada; 339 Division of Cancer Sciences, Manchester Cancer Research Centre, University of Manchester, Manchester, UK; 340 Radiation Medicine Program, Princess Margaret Cancer Centre, Toronto, ON Canada; 341 Department of Pathology, Brigham and Women’s Hospital, Harvard Medical School, Boston, MA USA; 342 Department of Surgery, Division of Thoracic Surgery, The Johns Hopkins University School of Medicine, Baltimore, MD USA; 343 Division of Molecular Pathology, The Netherlands Cancer Institute, Oncode Institute, Amsterdam, CX The Netherlands; 344 Department of Biomolecular Engineering, University of California Santa Cruz, Santa Cruz, CA USA; 345 UC Santa Cruz Genomics Institute, University of California Santa Cruz, Santa Cruz, CA USA; 346 Division of Applied Bioinformatics, German Cancer Research Center (DKFZ), Heidelberg, Germany; 347 German Cancer Consortium (DKTK), German Cancer Research Center (DKFZ), Heidelberg, Germany; 348 National Center for Tumor Diseases (NCT) Heidelberg, Heidelberg, Germany; 349 Center for Biological Sequence Analysis, Department of Bio and Health Informatics, Technical University of Denmark, Lyngby, Denmark; 350 Novo Nordisk Foundation Center for Protein Research, University of Copenhagen, Copenhagen, Denmark; 351 Institute for Molecular Bioscience, University of Queensland, St. Lucia, Brisbane, QLD Australia; 352 Biomedical Engineering, Oregon Health and Science University, Portland, OR USA; 353 Division of Theoretical Bioinformatics, German Cancer Research Center (DKFZ), Heidelberg, Germany; 354 Institute of Pharmacy and Molecular Biotechnology and BioQuant, Heidelberg University, Heidelberg, Germany; 355 Federal Ministry of Education and Research, Berlin, Germany; 356 Melanoma Institute Australia, University of Sydney, Sydney, NSW Australia; 357 Pediatric Hematology and Oncology, University Hospital Muenster, Muenster, Germany; 358 Department of Pathology, Johns Hopkins University School of Medicine, Baltimore, MD USA; 359 McKusick-Nathans Institute of Genetic Medicine, Sidney Kimmel Comprehensive Cancer Center at Johns Hopkins University School of Medicine, Baltimore, MD USA; 360 Foundation Medicine, Inc, Cambridge, MA USA; 361 Department of Biomedical Data Science, Stanford University School of Medicine, Stanford, CA USA; 362 Department of Genetics, Stanford University School of Medicine, Stanford, CA USA; 363 Bakar Computational Health Sciences Institute and Department of Pediatrics, University of California, San Francisco, CA USA; 364 Institute of Clinical Medicine, Faculty of Medicine, University of Oslo, Oslo, Norway; 365 National Cancer Institute, National Institutes of Health, Bethesda, MD USA; 366 Royal Marsden NHS Foundation Trust, London and Sutton, UK; 367 Genome Biology Unit, European Molecular Biology Laboratory (EMBL), Heidelberg, Germany; 368 Department of Oncology, University of Cambridge, Cambridge, UK; 369 Li Ka Shing Centre, Cancer Research UK Cambridge Institute, University of Cambridge, Cambridge, UK; 370 Institut Gustave Roussy, Villejuif, France; 371 Cambridge University Hospitals NHS Foundation Trust, Cambridge, UK; 372 Department of Haematology, University of Cambridge, Cambridge, UK; 373 Anatomia Patológica, Hospital Clinic, Institut d’Investigacions Biomèdiques August Pi i Sunyer (IDIBAPS), University of Barcelona, Barcelona, Spain; 374 Spanish Ministry of Science and Innovation, Madrid, Spain; 375 University of Michigan Comprehensive Cancer Center, Ann Arbor, MI USA; 376 Department for BioMedical Research, University of Bern, Bern, Switzerland; 377 Department of Medical Oncology, Inselspital, University Hospital and University of Bern, Bern, Switzerland; 378 Graduate School for Cellular and Biomedical Sciences, University of Bern, Bern, Switzerland; 379 University of Pavia, Pavia, Italy; 380 University of Alabama at Birmingham, Birmingham, AL USA; 381 UHN Program in BioSpecimen Sciences, Toronto General Hospital, Toronto, ON Canada; 382 Department of Urology, Icahn School of Medicine at Mount Sinai, New York, NY USA; 383 Centre for Law and Genetics, University of Tasmania, Sandy Bay Campus, Hobart, TAS Australia; 384 Faculty of Biosciences, Heidelberg University, Heidelberg, Germany; 385 Department of Biochemistry, Microbiology and Immunology, Faculty of Medicine, University of Ottawa, Ottawa, ON Canada; 386 Division of Anatomic Pathology, Mayo Clinic, Rochester, MN USA; 387 Division of Cancer Epidemiology and Genetics, National Cancer Institute, National Institutes of Health, Bethesda, MD USA; 388 Illawarra Shoalhaven Local Health District L3 Illawarra Cancer Care Centre, Wollongong Hospital, Wollongong, NSW Australia; 389 BioForA, French National Institute for Agriculture, Food, and Environment (INRAE), ONF, Orléans, France; 390 Department of Biostatistics, Bloomberg School of Public Health, Johns Hopkins University, Baltimore, MD USA; 391 University of California San Diego, San Diego, CA USA; 392 Division of Experimental Pathology, Mayo Clinic, Rochester, MN USA; 393 Centre for Cancer Research, The Westmead Institute for Medical Research, University of Sydney, Sydney, NSW Australia; 394 Department of Gynaecological Oncology, Westmead Hospital, Sydney, NSW Australia; 395 PDXen Biosystems Inc, Seoul, South Korea; 396 Korea Advanced Institute of Science and Technology, Daejeon, South Korea; 397 Electronics and Telecommunications Research Institute, Daejeon, South Korea; 398 Institut National du Cancer (INCA), Boulogne-Billancourt, France; 399 Department of Genetics, Informatics Institute, University of Alabama at Birmingham, Birmingham, AL USA; 400 Division of Medical Oncology, National Cancer Centre, Singapore, Singapore; 401 Medical Oncology, University and Hospital Trust of Verona, Verona, Italy; 402 Department of Pediatrics, University Hospital Schleswig-Holstein, Kiel, Germany; 403 Hepatobiliary/Pancreatic Surgical Oncology Program, University Health Network, Toronto, ON Canada; 404 School of Biological Sciences, University of Auckland, Auckland, New Zealand; 405 Department of Surgery, University of Melbourne, Parkville, VIC Australia; 406 The Murdoch Children’s Research Institute, Royal Children’s Hospital, Parkville, VIC Australia; 407 Walter and Eliza Hall Institute, Parkville, VIC Australia; 408 Vancouver Prostate Centre, Vancouver, Canada; 409 Lunenfeld-Tanenbaum Research Institute, Mount Sinai Hospital, Toronto, ON Canada; 410 University of East Anglia, Norwich, UK; 411 Norfolk and Norwich University Hospital NHS Trust, Norwich, UK; 412 Victorian Institute of Forensic Medicine, Southbank, VIC Australia; 413 Department of Biomedical Informatics, Harvard Medical School, Boston, MA USA; 414 Department of Chemistry, Centre for Molecular Science Informatics, University of Cambridge, Cambridge, UK; 415 Ludwig Center at Harvard Medical School, Boston, MA USA; 416 Human Genome Sequencing Center, Baylor College of Medicine, Houston, TX USA; 417 Peter MacCallum Cancer Centre, University of Melbourne, Melbourne, VIC Australia; 418 Physics Division, Optimization and Systems Biology Lab, Massachusetts General Hospital, Boston, MA USA; 419 Department of Medicine, Baylor College of Medicine, Houston, TX USA; 420 University of Cologne, Cologne, Germany; 421 International Genomics Consortium, Phoenix, AZ USA; 422 Genomics Research Program, Ontario Institute for Cancer Research, Toronto, ON Canada; 423 Barking Havering and Redbridge University Hospitals NHS Trust, Romford, UK; 424 Children’s Hospital at Westmead, University of Sydney, Sydney, NSW Australia; 425 Department of Medicine, Section of Endocrinology, University and Hospital Trust of Verona, Verona, Italy; 426 Computational Biology Center, Memorial Sloan Kettering Cancer Center, New York, NY USA; 427 Department of Biology, ETH Zurich, Zürich, Switzerland; 428 Department of Computer Science, ETH Zurich, Zurich, Switzerland; 429 SIB Swiss Institute of Bioinformatics, Lausanne, Switzerland; 430 Weill Cornell Medical College, New York, NY USA; 431 Academic Department of Medical Genetics, University of Cambridge, Addenbrooke’s Hospital, Cambridge, UK; 432 MRC Cancer Unit, University of Cambridge, Cambridge, UK; 433 Departments of Pediatrics and Genetics, University of North Carolina at Chapel Hill, Chapel Hill, NC USA; 434 Seven Bridges Genomics, Charlestown, MA USA; 435 Annai Systems, Inc, Carlsbad, CA USA; 436 Department of Pathology, General Hospital of Treviso, Department of Medicine, University of Padua, Treviso, Italy; 437 Department of Computational Biology, University of Lausanne, Lausanne, Switzerland; 438 Department of Genetic Medicine and Development, University of Geneva Medical School, Geneva, CH Switzerland; 439 Swiss Institute of Bioinformatics, University of Geneva, Geneva, CH Switzerland; 440 The Francis Crick Institute, London, UK; 441 University of Leuven, Leuven, Belgium; 442 Institute of Medical Genetics and Applied Genomics, University of Tübingen, Tübingen, Germany; 443 Computational and Systems Biology, Genome Institute of Singapore, Singapore, Singapore; 444 School of Computing, National University of Singapore, Singapore, Singapore; 445 Big Data Institute, Li Ka Shing Centre, University of Oxford, Oxford, UK; 446 Biomedical Data Science Laboratory, Francis Crick Institute, London, UK; 447 Bioinformatics Group, Department of Computer Science, University College London, London, UK; 448 The Edward S. Rogers Sr. Department of Electrical and Computer Engineering, University of Toronto, Toronto, ON Canada; 449 Breast Cancer Translational Research Laboratory JC Heuson, Institut Jules Bordet, Brussels, Belgium; 450 Department of Oncology, Laboratory for Translational Breast Cancer Research, KU Leuven, Leuven, Belgium; 451 Institute for Research in Biomedicine (IRB Barcelona), The Barcelona Institute of Science and Technology, Barcelona, Spain; 452 Research Program on Biomedical Informatics, Universitat Pompeu Fabra, Barcelona, Spain; 453 Division of Medical Oncology, Princess Margaret Cancer Centre, Toronto, ON Canada; 454 Department of Physiology and Biophysics, Weill Cornell Medicine, New York, NY USA; 455 Institute for Computational Biomedicine, Weill Cornell Medicine, New York, NY USA; 456 Department of Pathology, UPMC Shadyside, Pittsburgh, PA USA; 457 Independent Consultant, Wellesley, USA; 458 Department of Cell and Molecular Biology, Science for Life Laboratory, Uppsala University, Uppsala, Sweden; 459 Department of Medicine and Department of Genetics, Washington University School of Medicine, St. Louis, St. Louis, MO USA; 460 Hefei University of Technology, Anhui, China; 461 Translational Cancer Research Unit, GZA Hospitals St.-Augustinus, Center for Oncological Research, Faculty of Medicine and Health Sciences, University of Antwerp, Antwerp, Belgium; 462 Simon Fraser University, Burnaby, BC Canada; 463 University of Pennsylvania, Philadelphia, PA USA; 464 Faculty of Science and Technology, University of Vic—Central University of Catalonia (UVic-UCC), Vic, Spain; 465 The Wellcome Trust, London, UK; 466 The Hospital for Sick Children, Toronto, ON Canada; 467 Department of Pathology, Queen Elizabeth University Hospital, Glasgow, UK; 468 Department of Genetics and Computational Biology, QIMR Berghofer Medical Research Institute, Brisbane, QLD Australia; 469 Department of Oncology, Centre for Cancer Genetic Epidemiology, University of Cambridge, Cambridge, UK; 470 Department of Public Health and Primary Care, Centre for Cancer Genetic Epidemiology, University of Cambridge, Cambridge, UK; 471 Prostate Cancer Canada, Toronto, ON Canada; 472 University of Cambridge, Cambridge, UK; 473 Department of Laboratory Medicine, Translational Cancer Research, Lund University Cancer Center at Medicon Village, Lund University, Lund, Sweden; 474 Heidelberg University, Heidelberg, Germany; 475 New BIH Digital Health Center, Berlin Institute of Health (BIH) and Charité - Universitätsmedizin Berlin, Berlin, Germany; 476 CIBER Epidemiología y Salud Pública (CIBERESP), Madrid, Spain; 477 Research Group on Statistics, Econometrics and Health (GRECS), UdG, Barcelona, Spain; 478 Quantitative Genomics Laboratories (qGenomics), Barcelona, Spain; 479 Icelandic Cancer Registry, Icelandic Cancer Society, Reykjavik, Iceland; 480 State Key Laboratory of Cancer Biology, and Xijing Hospital of Digestive Diseases, Fourth Military Medical University, Shaanxi, China; 481 Department of Medicine (DIMED), Surgical Pathology Unit, University of Padua, Padua, Italy; 482 Rigshospitalet, Copenhagen, Denmark; 483 Center for Cancer Genomics, National Cancer Institute, National Institutes of Health, Bethesda, MD USA; 484 Department of Biochemistry and Molecular Medicine, University of Montreal, Montreal, QC Canada; 485 Australian Institute of Tropical Health and Medicine, James Cook University, Douglas, QLD Australia; 486 Department of Neuro-Oncology, Istituto Neurologico Besta, Milano, Italy; 487 Bioplatforms Australia, North Ryde, NSW Australia; 488 Department of Pathology (Research), University College London Cancer Institute, London, UK; 489 Department of Surgical Oncology, Princess Margaret Cancer Centre, Toronto, ON Canada; 490 Department of Medical Oncology, Josephine Nefkens Institute and Cancer Genomics Centre, Erasmus Medical Center, Rotterdam, CN The Netherlands; 491 The University of Queensland Thoracic Research Centre, The Prince Charles Hospital, Brisbane, QLD Australia; 492 CIBIO/InBIO - Research Center in Biodiversity and Genetic Resources, Universidade do Porto, Vairão, Portugal; 493 HCA Laboratories, London, UK; 494 University of Liverpool, Liverpool, UK; 495 The Azrieli Faculty of Medicine, Bar-Ilan University, Safed, Israel; 496 Department of Neurosurgery, University of Florida, Gainesville, FL USA; 497 Department of Pathology, Graduate School of Medicine, University of Tokyo, Tokyo, Japan; 498 University of Milano Bicocca, Monza, Italy; 499 BGI-Shenzhen, Shenzhen, China; 500 Department of Pathology, Oslo University Hospital Ulleval, Oslo, Norway; 501 Center for Biomedical Informatics, Harvard Medical School, Boston, MA USA; 502 Department Biochemistry and Molecular Biomedicine, University of Barcelona, Barcelona, Spain; 503 Office of Cancer Genomics, National Cancer Institute, National Institutes of Health, Bethesda, MD USA; 504 Cancer Epigenomics, German Cancer Research Center (DKFZ), Heidelberg, Germany; 505 Department of Cancer Biology, The University of Texas MD Anderson Cancer Center, Houston, TX USA; 506 Department of Surgical Oncology, The University of Texas MD Anderson Cancer Center, Houston, TX USA; 507 Department of Computer Science, Yale University, New Haven, CT USA; 508 Department of Molecular Biophysics and Biochemistry, Yale University, New Haven, CT USA; 509 Program in Computational Biology and Bioinformatics, Yale University, New Haven, CT USA; 510 Center for Cancer Research, Massachusetts General Hospital, Boston, MA USA; 511 Department of Pathology, Massachusetts General Hospital, Boston, MA USA; 512 Department of Pathology, Memorial Sloan Kettering Cancer Center, New York, NY USA; 513 Division of Gastroenterology and Hepatology, Mayo Clinic, Rochester, MN USA; 514 University of Sydney, Sydney, NSW Australia; 515 University of Oxford, Oxford, UK; 516 Department of Surgery, Academic Urology Group, University of Cambridge, Cambridge, UK; 517 Department of Medicine II, University of Würzburg, Wuerzburg, Germany; 518 Sylvester Comprehensive Cancer Center, University of Miami, Miami, FL USA; 519 Institut Hospital del Mar d’Investigacions Mèdiques (IMIM), Barcelona, Spain; 520 Genome Integrity and Structural Biology Laboratory, National Institute of Environmental Health Sciences (NIEHS), Durham, NC USA; 521 St. Thomas’s Hospital, London, UK; 522 Osaka International Cancer Center, Osaka, Japan; 523 Department of Pathology, Skåne University Hospital, Lund University, Lund, Sweden; 524 Department of Medical Oncology, Beatson West of Scotland Cancer Centre, Glasgow, UK; 525 National Human Genome Research Institute, National Institutes of Health, Bethesda, MD USA; 526 Centre for Cancer Research, Victorian Comprehensive Cancer Centre, University of Melbourne, Melbourne, VIC Australia; 527 Department of Medicine, Section of Hematology/Oncology, University of Chicago, Chicago, IL USA; 528 German Center for Infection Research (DZIF), Partner Site Hamburg-Borstel-Lübeck-Riems, Hamburg, Germany; 529 Bioinformatics Research Centre (BiRC), Aarhus University, Aarhus, Denmark; 530 Department of Biotechnology, Ministry of Science and Technology, Government of India, New Delhi, Delhi India; 531 National Cancer Centre Singapore, Singapore, Singapore; 532 Brandeis University, Waltham, MA USA; 533 Department of Urologic Sciences, University of British Columbia, Vancouver, BC Canada; 534 Department of Internal Medicine, Stanford University, Stanford, CA USA; 535 The University of Texas Health Science Center at Houston, Houston, TX USA; 536 Imperial College NHS Trust, Imperial College, London, INY UK; 537 Senckenberg Institute of Pathology, University of Frankfurt Medical School, Frankfurt, Germany; 538 Department of Medicine, Division of Biomedical Informatics, UC San Diego School of Medicine, San Diego, CA USA; 539 Center for Precision Health, School of Biomedical Informatics, The University of Texas Health Science Center, Houston, TX USA; 540 Oxford Nanopore Technologies, New York, NY USA; 541 Institute of Medical Science, University of Tokyo, Tokyo, Japan; 542 Howard Hughes Medical Institute, University of California Santa Cruz, Santa Cruz, CA USA; 543 Wakayama Medical University, Wakayama, Japan; 544 Department of Internal Medicine, Division of Medical Oncology, Lineberger Comprehensive Cancer Center, University of North Carolina at Chapel Hill, Chapel Hill, NC USA; 545 University of Tennessee Health Science Center for Cancer Research, Memphis, TN USA; 546 Department of Histopathology, Salford Royal NHS Foundation Trust, Salford, UK; 547 Faculty of Biology, Medicine and Health, University of Manchester, Manchester, UK; 548 BIOPIC, ICG and College of Life Sciences, Peking University, Beijing, China; 549 Peking-Tsinghua Center for Life Sciences, Peking University, Beijing, China; 550 Children’s Hospital of Philadelphia, Philadelphia, PA USA; 551 Department of Bioinformatics and Computational Biology and Department of Systems Biology, The University of Texas MD Anderson Cancer Center, Houston, TX USA; 552 Karolinska Institute, Stockholm, Sweden; 553 The Donnelly Centre, University of Toronto, Toronto, ON Canada; 554 Department of Medical Genetics, College of Medicine, Hallym University, Chuncheon, South Korea; 555 Department of Experimental and Health Sciences, Institute of Evolutionary Biology (UPF-CSIC), Universitat Pompeu Fabra, Barcelona, Spain; 556 Health Data Science Unit, University Clinics, Heidelberg, Germany; 557 Massachusetts General Hospital Center for Cancer Research, Charlestown, MA USA; 558 Hokkaido University, Sapporo, Japan; 559 Department of Pathology and Clinical Laboratory, National Cancer Center Hospital, Tokyo, Japan; 560 Department of Genetics, University of North Carolina at Chapel Hill, Chapel Hill, NC USA; 561 Computational Biology, Leibniz Institute on Aging - Fritz Lipmann Institute (FLI), Jena, Germany; 562 University of Melbourne Centre for Cancer Research, Melbourne, VIC Australia; 563 University of Nebraska Medical Center, Omaha, NE USA; 564 Syntekabio Inc, Daejeon, South Korea; 565 Department of Pathology, Academic Medical Center, Amsterdam, AZ The Netherlands; 566 China National GeneBank-Shenzhen, Shenzhen, China; 567 Division of Molecular Genetics, German Cancer Research Center (DKFZ), Heidelberg, Germany; 568 Division of Life Science and Applied Genomics Center, Hong Kong University of Science and Technology, Clear Water Bay, Hong Kong, China; 569 Icahn School of Medicine at Mount Sinai, New York, NY USA; 570 Geneplus-Shenzhen, Shenzhen, China; 571 School of Computer Science and Technology, Xi’an Jiaotong University, Xi’an, China; 572 AbbVie, North Chicago, IL USA; 573 Institute of Pathology, Charité – University Medicine Berlin, Berlin, Germany; 574 Centre for Translational and Applied Genomics, British Columbia Cancer Agency, Vancouver, BC Canada; 575 Edinburgh Royal Infirmary, Edinburgh, UK; 576 Berlin Institute for Medical Systems Biology, Max Delbrück Center for Molecular Medicine, Berlin, Germany; 577 Department of Pediatric Immunology, Hematology and Oncology, University Hospital, Heidelberg, Germany; 578 German Cancer Research Center (DKFZ), Heidelberg, Germany; 579 Heidelberg Institute for Stem Cell Technology and Experimental Medicine (HI-STEM), Heidelberg, Germany; 580 Institute for Computational Biomedicine, Weill Cornell Medical College, New York, NY USA; 581 New York Genome Center, New York, NY USA; 582 Department of Urology, James Buchanan Brady Urological Institute, Johns Hopkins University School of Medicine, Baltimore, MD USA; 583 Department of Preventive Medicine, Graduate School of Medicine, The University of Tokyo, Tokyo, Japan; 584 Department of Molecular and Cellular Biology, Baylor College of Medicine, Houston, TX USA; 585 Department of Pathology and Immunology, Baylor College of Medicine, Houston, TX USA; 586 Michael E. DeBakey Veterans Affairs Medical Center, Houston, TX USA; 587 Technical University of Denmark, Lyngby, Denmark; 588 Department of Pathology, College of Medicine, Hanyang University, Seoul, South Korea; 589 Academic Unit of Surgery, School of Medicine, College of Medical, Veterinary and Life Sciences, University of Glasgow, Glasgow Royal Infirmary, Glasgow, UK; 590 Department of Pathology, Asan Medical Center, College of Medicine, Ulsan University, Songpa-gu, Seoul South Korea; 591 Science Writer, Garrett Park, MD USA; 592 International Cancer Genome Consortium (ICGC)/ICGC Accelerating Research in Genomic Oncology (ARGO) Secretariat, Ontario Institute for Cancer Research, Toronto, ON Canada; 593 University of Ljubljana, Ljubljana, Slovenia; 594 Department of Public Health Sciences, University of Chicago, Chicago, IL USA; 595 Research Institute, NorthShore University HealthSystem, Evanston, IL USA; 596 Department for Biomedical Research, University of Bern, Bern, Switzerland; 597 Centre of Genomics and Policy, McGill University and Génome Québec Innovation Centre, Montreal, QC Canada; 598 Carolina Center for Genome Sciences, University of North Carolina at Chapel Hill, Chapel Hill, NC USA; 599 Hopp Children’s Cancer Center (KiTZ), Heidelberg, Germany; 600 Pediatric Glioma Research Group, German Cancer Research Center (DKFZ), Heidelberg, Germany; 601 Cancer Research UK, London, UK; 602 Indivumed GmbH, Hamburg, Germany; 603 Genome Integration Data Center, Syntekabio, Inc, Daejeon, South Korea; 604 University Hospital Zurich, Zurich, Switzerland; 605 Clinical Bioinformatics, Swiss Institute of Bioinformatics, Geneva, Switzerland; 606 Institute for Pathology and Molecular Pathology, University Hospital Zurich, Zurich, Switzerland; 607 Institute of Molecular Life Sciences, University of Zurich, Zurich, Switzerland; 608 MRC Human Genetics Unit, MRC IGMM, University of Edinburgh, Edinburgh, UK; 609 Women’s Cancer Program at the Samuel Oschin Comprehensive Cancer Institute, Cedars-Sinai Medical Center, Los Angeles, CA USA; 610 Department of Biology, Bioinformatics Group, Division of Molecular Biology, Faculty of Science, University of Zagreb, Zagreb, Croatia; 611 Department for Internal Medicine II, University Hospital Schleswig-Holstein, Kiel, Germany; 612 Genetics and Molecular Pathology, SA Pathology, Adelaide, SA Australia; 613 Department of Gastric Surgery, National Cancer Center Hospital, Tokyo, Japan; 614 Department of Bioinformatics, Division of Cancer Genomics, National Cancer Center Research Institute, Tokyo, Japan; 615 A.A. Kharkevich Institute of Information Transmission Problems, Moscow, Russia; 616 Oncology and Immunology, Dmitry Rogachev National Research Center of Pediatric Hematology, Moscow, Russia; 617 Skolkovo Institute of Science and Technology, Moscow, Russia; 618 Department of Surgery, The George Washington University, School of Medicine and Health Science, Washington, DC USA; 619 Endocrine Oncology Branch, Center for Cancer Research, National Cancer Institute, National Institutes of Health, Bethesda, MD USA; 620 Melanoma Institute Australia, Macquarie University, Sydney, NSW Australia; 621 MIT Computer Science and Artificial Intelligence Laboratory, Massachusetts Institute of Technology, Cambridge, MA USA; 622 Tissue Pathology and Diagnostic Oncology, Royal Prince Alfred Hospital, Sydney, NSW Australia; 623 Cholangiocarcinoma Screening and Care Program and Liver Fluke and Cholangiocarcinoma Research Centre, Faculty of Medicine, Khon Kaen University, Khon Kaen, Thailand; 624 Controlled Department and Institution, New York, NY USA; 625 Englander Institute for Precision Medicine, Weill Cornell Medicine, New York, NY USA; 626 National Cancer Center, Gyeonggi, South Korea; 627 Department of Biochemistry, College of Medicine, Ewha Womans University, Seoul, South Korea; 628 Health Sciences Department of Biomedical Informatics, University of California San Diego, La Jolla, CA USA; 629 Research Core Center, National Cancer Centre Korea, Goyang-si, South Korea; 630 Department of Health Sciences and Technology, Sungkyunkwan University School of Medicine, Seoul, South Korea; 631 Samsung Genome Institute, Seoul, South Korea; 632 Breast Oncology Program, Dana-Farber/Brigham and Women’s Cancer Center, Boston, MA USA; 633 Department of Surgery, Memorial Sloan Kettering Cancer Center, New York, NY USA; 634 Division of Breast Surgery, Brigham and Women’s Hospital, Boston, MA USA; 635 Integrative Bioinformatics Support Group, National Institute of Environmental Health Sciences (NIEHS), Durham, NC USA; 636 Department of Clinical Science, University of Bergen, Bergen, Norway; 637 Center For Medical Innovation, Seoul National University Hospital, Seoul, South Korea; 638 Department of Internal Medicine, Seoul National University Hospital, Seoul, South Korea; 639 Institute of Computer Science, Polish Academy of Sciences, Warsawa, Poland; 640 Functional and Structural Genomics, German Cancer Research Center (DKFZ), Heidelberg, Germany; 641 Laboratory of Translational Genomics, Division of Cancer Epidemiology and Genetics, National Cancer Institute, , National Institutes of Health, Bethesda, MD USA; 642 Institute for Medical Informatics Statistics and Epidemiology, University of Leipzig, Leipzig, Germany; 643 Morgan Welch Inflammatory Breast Cancer Research Program and Clinic, The University of Texas MD Anderson Cancer Center, Houston, TX USA; 644 Department of Hematology and Oncology, Georg-Augusts-University of Göttingen, Göttingen, Germany; 645 Institute of Cell Biology (Cancer Research), University of Duisburg-Essen, Essen, Germany; 646 King’s College London and Guy’s and St. Thomas’ NHS Foundation Trust, London, UK; 647 Center for Epigenetics, Van Andel Research Institute, Grand Rapids, MI USA; 648 The University of Queensland Centre for Clinical Research, Royal Brisbane and Women’s Hospital, Herston, QLD Australia; 649 Department of Pediatric Oncology and Hematology, University of Cologne, Cologne, Germany; 650 University of Düsseldorf, Düsseldorf, Germany; 651 Department of Pathology, Institut Jules Bordet, Brussels, Belgium; 652 Institute of Biomedicine, Sahlgrenska Academy at University of Gothenburg, Gothenburg, Sweden; 653 Children’s Medical Research Institute, Sydney, NSW Australia; 654 ILSbio, LLC Biobank, Chestertown, MD USA; 655 Division of Genetics and Genomics, Boston Children’s Hospital, Harvard Medical School, Boston, MA USA; 656 Institute for Bioengineering and Biopharmaceutical Research (IBBR), Hanyang University, Seoul, South Korea; 657 Department of Statistics, University of California Santa Cruz, Santa Cruz, CA USA; 658 National Genotyping Center, Institute of Biomedical Sciences, Academia Sinica, Taipei, Taiwan; 659 Department of Vertebrate Genomics/Otto Warburg Laboratory Gene Regulation and Systems Biology of Cancer, Max Planck Institute for Molecular Genetics, Berlin, Germany; 660 McGill University and Genome Quebec Innovation Centre, Montreal, QC Canada; 661 biobyte solutions GmbH, Heidelberg, Germany; 662 Gynecologic Oncology, NYU Laura and Isaac Perlmutter Cancer Center, New York University, New York, NY USA; 663 Division of Oncology, Stem Cell Biology Section, Washington University School of Medicine, St. Louis, MO USA; 664 Department of Systems Biology, The University of Texas MD Anderson Cancer Center, Houston, TX USA; 665 Harvard University, Cambridge, MA USA; 666 Urologic Oncology Branch, Center for Cancer Research, National Cancer Institute, National Institutes of Health, Bethesda, MD USA; 667 University of Oslo, Oslo, Norway; 668 University of Toronto, Toronto, ON Canada; 669 Peking University, Beijing, China; 670 School of Life Sciences, Peking University, Beijing, China; 671 Leidos Biomedical Research, Inc, McLean, VA USA; 672 Hematology, Hospital Clinic, Institut d’Investigacions Biomèdiques August Pi i Sunyer (IDIBAPS), University of Barcelona, Barcelona, Spain; 673 Second Military Medical University, Shanghai, China; 674 Chinese Cancer Genome Consortium, Shenzhen, China; 675 Department of Medical Oncology, Beijing Hospital, Beijing, China; 676 Laboratory of Molecular Oncology, Key Laboratory of Carcinogenesis and Translational Research (Ministry of Education), Peking University Cancer Hospital and Institute, Beijing, China; 677 School of Medicine/School of Mathematics and Statistics, University of St. Andrews, St, Andrews, Fife UK; 678 Institute for Systems Biology, Seattle, WA USA; 679 Department of Biochemistry and Molecular Biology, Faculty of Medicine, University Institute of Oncology-IUOPA, Oviedo, Spain; 680 Institut Bergonié, Bordeaux, France; 681 Cancer Unit, MRC University of Cambridge, Cambridge, UK; 682 Department of Pathology and Laboratory Medicine, Center for Personalized Medicine, Children’s Hospital Los Angeles, Los Angeles, CA USA; 683 John Curtin School of Medical Research, Canberra, ACT Australia; 684 MVZ Department of Oncology, PraxisClinic am Johannisplatz, Leipzig, Germany; 685 Department of Information Technology, Ghent University, Ghent, Belgium; 686 Department of Plant Biotechnology and Bioinformatics, Ghent University, Ghent, Belgium; 687 Institute for Genomic Medicine, Nationwide Children’s Hospital, Columbus, OH USA; 688 Computational Biology Program, School of Medicine, Oregon Health and Science University, Portland, OR USA; 689 Department of Surgery, Duke University, Durham, NC USA; 690 Institució Catalana de Recerca i Estudis Avançats (ICREA), Barcelona, Spain; 691 Institut Català de Paleontologia Miquel Crusafont, Universitat Autònoma de Barcelona, Barcelona, Spain; 692 University of Glasgow, Glasgow, UK; 693 Institut d’Investigacions Biomèdiques August Pi i Sunyer (IDIBAPS), Barcelona, Spain; 694 Division of Oncology, Washington University School of Medicine, St. Louis, MO USA; 695 Department of Surgery and Cancer, Imperial College, London, INY UK; 696 Applications Department, Oxford Nanopore Technologies, Oxford, UK; 697 Department of Obstetrics, Gynecology and Reproductive Services, University of California San Francisco, San Francisco, CA USA; 698 Department of Biochemistry and Molecular Medicine, University California at Davis, Sacramento, CA USA; 699 STTARR Innovation Facility, Princess Margaret Cancer Centre, Toronto, ON Canada; 700 Discipline of Surgery, Western Sydney University, Penrith, NSW Australia; 701 Yale School of Medicine, Yale University, New Haven, CT USA; 702 Department of Genetics, Lineberger Comprehensive Cancer Center, University of North Carolina at Chapel Hill, Chapel Hill, NC USA; 703 Departments of Neurology and Neurosurgery, Henry Ford Hospital, Detroit, MI USA; 704 Precision Oncology, OHSU Knight Cancer Institute, Oregon Health and Science University, Portland, OR USA; 705 Institute of Pathology, University Medical Center Hamburg-Eppendorf, Hamburg, Germany; 706 Department of Health Sciences, Faculty of Medical Sciences, Kyushu University, Fukuoka, Japan; 707 Heidelberg Academy of Sciences and Humanities, Heidelberg, Germany; 708 Department of Clinical Pathology, University of Melbourne, Melbourne, VIC, Australia; 709 Department of Pathology, Roswell Park Cancer Institute, Buffalo, NY USA; 710 Department of Computer Science, University of Helsinki, Helsinki, Finland; 711 Institute of Biotechnology, University of Helsinki, Helsinki, Finland; 712 Organismal and Evolutionary Biology Research Programme, University of Helsinki, Helsinki, Finland; 713 Department of Obstetrics and Gynecology, Division of Gynecologic Oncology, Washington University School of Medicine, St. Louis, MO USA; 714 Penrose St. Francis Health Services, Colorado Springs, CO USA; 715 Institute of Pathology, Ulm University and University Hospital of Ulm, Ulm, Germany; 716 National Cancer Center, Tokyo, Japan; 717 Genome Institute of Singapore, Singapore, Singapore; 718 32Program in Computational Biology and Bioinformatics, Yale University, New Haven, CT USA; 719 German Cancer Aid, Bonn, Germany; 720 Programme in Cancer and Stem Cell Biology, Centre for Computational Biology, Duke-NUS Medical School, Singapore, Singapore; 721 The Chinese University of Hong Kong, Shatin, NT, Hong Kong China; 722 Fourth Military Medical University, Shaanxi, China; 723 The University of Cambridge School of Clinical Medicine, Cambridge, UK; 724 St. Jude Children’s Research Hospital, Memphis, TN USA; 725 University Health Network, Princess Margaret Cancer Centre, Toronto, ON Canada; 726 Center for Biomolecular Science and Engineering, University of California Santa Cruz, Santa Cruz, CA USA; 727 Department of Medicine, University of Chicago, Chicago, IL USA; 728 Department of Neurology, Mayo Clinic, Rochester, MN USA; 729 Cambridge Oesophagogastric Centre, Cambridge University Hospitals NHS Foundation Trust, Cambridge, UK; 730 Department of Computer Science, Carleton College, Northfield, MN USA; 731 Institute of Cancer Sciences, College of Medical Veterinary and Life Sciences, University of Glasgow, Glasgow, UK; 732 Department of Epidemiology, University of Alabama at Birmingham, Birmingham, AL USA; 733 HudsonAlpha Institute for Biotechnology, Huntsville, AL USA; 734 O’Neal Comprehensive Cancer Center, University of Alabama at Birmingham, Birmingham, AL USA; 735 Department of Pathology, Keio University School of Medicine, Tokyo, Japan; 736 Department of Hepatobiliary and Pancreatic Oncology, National Cancer Center Hospital, Tokyo, Japan; 737 Sage Bionetworks, Seattle, WA USA; 738 Lymphoma Genomic Translational Research Laboratory, National Cancer Centre, Singapore, Singapore; 739 Department of Clinical Pathology, Robert-Bosch-Hospital, Stuttgart, Germany; 740 Department of Cell and Systems Biology, University of Toronto, Toronto, ON Canada; 741 Department of Biosciences and Nutrition, Karolinska Institutet, Stockholm, Sweden; 742 Center for Liver Cancer, Research Institute and Hospital, National Cancer Center, Gyeonggi, South Korea; 743 Division of Hematology-Oncology, Samsung Medical Center, Sungkyunkwan University School of Medicine, Seoul, South Korea; 744 Samsung Advanced Institute for Health Sciences and Technology, Sungkyunkwan University School of Medicine, Seoul, South Korea; 745 Cheonan Industry-Academic Collaboration Foundation, Sangmyung University, Cheonan, South Korea; 746 NYU Langone Medical Center, New York, NY USA; 747 Department of Hematology and Medical Oncology, Cleveland Clinic, Cleveland, OH USA; 748 Department of Radiation Oncology, University of California San Francisco, San Francisco, CA USA; 749 Department of Health Sciences Research, Mayo Clinic, Rochester, MN USA; 750 Helen F. Graham Cancer Center at Christiana Care Health Systems, Newark, DE USA; 751 Heidelberg University Hospital, Heidelberg, Germany; 752 CSRA Incorporated, Fairfax, VA USA; 753 Research Department of Pathology, University College London Cancer Institute, London, UK; 754 Department of Research Oncology, Guy’s Hospital, King’s Health Partners AHSC, King’s College London School of Medicine, London, UK; 755 Faculty of Medicine and Health Sciences, Macquarie University, Sydney, NSW Australia; 756 University Hospital of Minjoz, INSERM UMR 1098, Besançon, France; 757 Spanish National Cancer Research Centre, Madrid, Spain; 758 Center of Digestive Diseases and Liver Transplantation, Fundeni Clinical Institute, Bucharest, Romania; 759 Cureline, Inc, South San Francisco, CA USA; 760 St. Luke’s Cancer Centre, Royal Surrey County Hospital NHS Foundation Trust, Guildford, UK; 761 Cambridge Breast Unit, Addenbrooke’s Hospital, Cambridge University Hospital NHS Foundation Trust and NIHR Cambridge Biomedical Research Centre, Cambridge, UK; 762 East of Scotland Breast Service, Ninewells Hospital, Aberdeen, UK; 763 Department of Genetics, Microbiology and Statistics, University of Barcelona, IRSJD, IBUB, Barcelona, Spain; 764 Department of Obstetrics and Gynecology, Medical College of Wisconsin, Milwaukee, WI USA; 765 Hematology and Medical Oncology, Winship Cancer Institute of Emory University, Atlanta, GA USA; 766 Department of Computer Science, Princeton University, Princeton, NJ USA; 767 Vanderbilt Ingram Cancer Center, Vanderbilt University, Nashville, TN USA; 768 Ohio State University College of Medicine and Arthur G. James Comprehensive Cancer Center, Columbus, OH USA; 769 Department of Surgery, Yokohama City University Graduate School of Medicine, Kanagawa, Japan; 770 Division of Chromatin Networks, German Cancer Research Center (DKFZ) and BioQuant, Heidelberg, Germany; 771 Research Computing Center, University of North Carolina at Chapel Hill, Chapel Hill, NC USA; 772 School of Molecular Biosciences and Center for Reproductive Biology, Washington State University, Pullman, WA USA; 773 Finsen Laboratory and Biotech Research and Innovation Centre (BRIC), University of Copenhagen, Copenhagen, Denmark; 774 Department of Laboratory Medicine and Pathobiology, University of Toronto, Toronto, ON Canada; 775 Department of Pathology, Human Oncology and Pathogenesis Program, Memorial Sloan Kettering Cancer Center, New York, NY USA; 776 University Hospital Giessen, Pediatric Hematology and Oncology, Giessen, Germany; 777 Oncologie Sénologie, ICM Institut Régional du Cancer, Montpellier, France; 778 Institute of Clinical Molecular Biology, Christian-Albrechts-University, Kiel, Germany; 779 Institute of Pathology, University of Wuerzburg, Wuerzburg, Germany; 780 Department of Urology, North Bristol NHS Trust, Bristol, UK; 781 SingHealth, Duke-NUS Institute of Precision Medicine, National Heart Centre Singapore, Singapore, Singapore; 782 Department of Computer Science, University of Toronto, Toronto, ON Canada; 783 Bern Center for Precision Medicine, University Hospital of Bern, University of Bern, Bern, Switzerland; 784 Englander Institute for Precision Medicine, Weill Cornell Medicine and New York Presbyterian Hospital, New York, NY USA; 785 Meyer Cancer Center, Weill Cornell Medicine, New York, NY USA; 786 Pathology and Laboratory, Weill Cornell Medical College, New York, NY USA; 787 Vall d’Hebron Institute of Oncology: VHIO, Barcelona, Spain; 788 General and Hepatobiliary-Biliary Surgery, Pancreas Institute, University and Hospital Trust of Verona, Verona, Italy; 789 National Centre for Biological Sciences, Tata Institute of Fundamental Research, Bangalore, India; 790 Indiana University, Bloomington, IN USA; 791 Department of Pathology, GZA-ZNA Hospitals, Antwerp, Belgium; 792 Analytical Biological Services, Inc, Wilmington, DE USA; 793 Sydney Medical School, University of Sydney, Sydney, NSW Australia; 794 cBio Center, Dana-Farber Cancer Institute, Harvard Medical School, Boston, MA USA; 795 Department of Cell Biology, Harvard Medical School, Boston, MA USA; 796 Advanced Centre for Treatment Research and Education in Cancer, Tata Memorial Centre, Navi Mumbai, Maharashtra India; 797 School of Environmental and Life Sciences, Faculty of Science, The University of Newcastle, Ourimbah, NSW Australia; 798 Department of Dermatology, University Hospital of Essen, Essen, Germany; 799 Bioinformatics and Omics Data Analytics, German Cancer Research Center (DKFZ), Heidelberg, Germany; 800 Department of Urology, Charité Universitätsmedizin Berlin, Berlin, Germany; 801 Martini-Clinic, Prostate Cancer Center, University Medical Center Hamburg-Eppendorf, Hamburg, Germany; 802 Department of General Internal Medicine, University of Kiel, Kiel, Germany; 803 German Cancer Consortium (DKTK), Partner site Berlin, Berlin, Germany; 804 Cancer Research Institute, Beth Israel Deaconess Medical Center, Boston, MA USA; 805 University of Pittsburgh, Pittsburgh, PA USA; 806 Department of Ophthalmology and Ocular Genomics Institute, Massachusetts Eye and Ear, Harvard Medical School, Boston, MA USA; 807 Center for Psychiatric Genetics, NorthShore University HealthSystem, Evanston, IL USA; 808 Van Andel Research Institute, Grand Rapids, MI USA; 809 Laboratory of Molecular Medicine, Human Genome Center, Institute of Medical Science, University of Tokyo, Tokyo, Japan; 810 Japan Agency for Medical Research and Development, Tokyo, Japan; 811 Korea University, Seoul, South Korea; 812 Murtha Cancer Center, Walter Reed National Military Medical Center, Bethesda, MD USA; 813 Human Genetics, University of Kiel, Kiel, Germany; 814 Department of Oncologic Pathology, Dana-Farber Cancer Institute, Harvard Medical School, Boston, MA USA; 815 Oregon Health and Science University, Portland, OR USA; 816 Center for RNA Interference and Noncoding RNA, The University of Texas MD Anderson Cancer Center, Houston, TX USA; 817 Department of Experimental Therapeutics, The University of Texas MD Anderson Cancer Center, Houston, TX USA; 818 Department of Gynecologic Oncology and Reproductive Medicine, The University of Texas MD Anderson Cancer Center, Houston, TX USA; 819 University Hospitals Coventry and Warwickshire NHS Trust, Coventry, UK; 820 Department of Radiation Oncology, Radboud University Nijmegen Medical Centre, Nijmegen, GA The Netherlands; 821 Institute for Genomics and Systems Biology, University of Chicago, Chicago, IL USA; 822 Clinic for Hematology and Oncology, St.-Antonius-Hospital, Eschweiler, Germany; 823 Computational and Systems Biology Program, Memorial Sloan Kettering Cancer Center, New York, NY USA; 824 University of Iceland, Reykjavik, Iceland; 825 Division of Computational Genomics and Systems Genetics, German Cancer Research Center (DKFZ), Heidelberg, Germany; 826 Dundee Cancer Centre, Ninewells Hospital, Dundee, UK; 827 Department for Internal Medicine III, University of Ulm and University Hospital of Ulm, Ulm, Germany; 828 Institut Curie, INSERM Unit 830, Paris, France; 829 Department of Gastroenterology and Hepatology, Yokohama City University Graduate School of Medicine, Kanagawa, Japan; 830 Department of Laboratory Medicine, Radboud University Nijmegen Medical Centre, Nijmegen, GA The Netherlands; 831 Division of Cancer Genome Research, German Cancer Research Center (DKFZ), Heidelberg, Germany; 832 Department of General Surgery, Singapore General Hospital, Singapore, Singapore; 833 Cancer Science Institute of Singapore, National University of Singapore, Singapore, Singapore; 834 Department of Medical and Clinical Genetics, Genome-Scale Biology Research Program, University of Helsinki, Helsinki, Finland; 835 East Anglian Medical Genetics Service, Cambridge University Hospitals NHS Foundation Trust, Cambridge, UK; 836 Irving Institute for Cancer Dynamics, Columbia University, New York, NY USA; 837 Institute of Molecular and Cell Biology, Singapore, Singapore; 838 Laboratory of Cancer Epigenome, Division of Medical Science, National Cancer Centre Singapore, Singapore, Singapore; 839 Universite Lyon, INCa-Synergie, Centre Léon Bérard, Lyon, France; 840 Department of Urology, Mayo Clinic, Rochester, MN USA; 841 Royal National Orthopaedic Hospital - Stanmore, Stanmore, Middlesex UK; 842 Department of Biochemistry, Genetics and Immunology, University of Vigo, Vigo, Spain; 843 Giovanni Paolo II / I.R.C.C.S. Cancer Institute, Bari, BA Italy; 844 Neuroblastoma Genomics, German Cancer Research Center (DKFZ), Heidelberg, Germany; 845 Fondazione Policlinico Universitario Gemelli IRCCS, Rome, Italy, Rome, Italy; 846 University of Verona, Verona, Italy; 847 Centre National de Génotypage, CEA - Institute de Génomique, Evry, France; 848 CAPHRI Research School, Maastricht University, Maastricht, ER The Netherlands; 849 Department of Biopathology, Centre Léon Bérard, Lyon, France; 850 Université Claude Bernard Lyon 1, Villeurbanne, France; 851 Core Research for Evolutional Science and Technology (CREST), JST, Tokyo, Japan; 852 Department of Biological Sciences, Laboratory for Medical Science Mathematics, Graduate School of Science, University of Tokyo, Yokohama, Japan; 853 Department of Medical Science Mathematics, Medical Research Institute, Tokyo Medical and Dental University (TMDU), Tokyo, Japan; 854 Cancer Ageing and Somatic Mutation Programme, Wellcome Sanger Institute, Hinxton, UK; 855 University Hospitals Birmingham NHS Foundation Trust, Birmingham, UK; 856 Centre for Cancer Research and Cell Biology, Queen’s University, Belfast, UK; 857 Breast Medical Oncology, The University of Texas MD Anderson Cancer Center, Houston, TX USA; 858 Department of Surgery, Johns Hopkins University School of Medicine, Baltimore, MD USA; 859 Department of Oncology-Pathology, Science for Life Laboratory, Karolinska Institute, Stockholm, Sweden; 860 School of Cancer Sciences, Faculty of Medicine, University of Southampton, Southampton, UK; 861 Department of Gene Technology, Tallinn University of Technology, Tallinn, Estonia; 862 Genetics and Genome Biology Program, SickKids Research Institute, The Hospital for Sick Children, Toronto, ON Canada; 863 Departments of Neurosurgery and Hematology and Medical Oncology, Winship Cancer Institute and School of Medicine, Emory University, Atlanta, GA USA; 864 Department of Clinical and Molecular Medicine, Faculty of Medicine and Health Sciences, Norwegian University of Science and Technology, Trondheim, Norway; 865 Argmix Consulting, North Vancouver, BC Canada; 866 Department of Information Technology, Ghent University, Interuniversitair Micro-Electronica Centrum (IMEC), Ghent, Belgium; 867 Nuffield Department of Surgical Sciences, John Radcliffe Hospital, University of Oxford, Oxford, UK; 868 Institute of Mathematics and Computer Science, University of Latvia, Riga, LV Latvia; 869 Discipline of Pathology, Sydney Medical School, University of Sydney, Sydney, NSW Australia; 870 Department of Applied Mathematics and Theoretical Physics, Centre for Mathematical Sciences, University of Cambridge, Cambridge, UK; 871 Department of Epidemiology and Biostatistics, Memorial Sloan Kettering Cancer Center, New York, NY USA; 872 Department of Statistics, Columbia University, New York, NY USA; 873 Department of Immunology, Genetics and Pathology, Science for Life Laboratory, Uppsala University, Uppsala, Sweden; 874 School of Electronic and Information Engineering, Xi’an Jiaotong University, Xi’an, China; 875 Department of Histopathology, Cambridge University Hospitals NHS Foundation Trust, Cambridge, UK; 876 Oxford NIHR Biomedical Research Centre, University of Oxford, Oxford, UK; 877 Georgia Regents University Cancer Center, Augusta, GA USA; 878 Wythenshawe Hospital, Manchester, UK; 879 Department of Genetics, Washington University School of Medicine, St.Louis, MO USA; 880 Department of Biological Oceanography, Leibniz Institute of Baltic Sea Research, Rostock, Germany; 881 Wellcome Centre for Human Genetics, University of Oxford, Oxford, UK; 882 Department of Molecular and Human Genetics, Baylor College of Medicine, Houston, TX USA; 883 Thoracic Oncology Laboratory, Mayo Clinic, Rochester, MN USA; 884 Institute for Genomic Medicine, Nationwide Children’s Hospital, Columbus, OH USA; 885 Department of Obstetrics and Gynecology, Division of Gynecologic Oncology, Mayo Clinic, Rochester, MN USA; 886 International Institute for Molecular Oncology, Poznań, Poland; 887 Poznan University of Medical Sciences, Poznań, Poland; 888 Genomics and Proteomics Core Facility High Throughput Sequencing Unit, German Cancer Research Center (DKFZ), Heidelberg, Germany; 889 NCCS-VARI Translational Research Laboratory, National Cancer Centre Singapore, Singapore, Singapore; 890 Edison Family Center for Genome Sciences and Systems Biology, Washington University, St. Louis, MO USA; 891 MRC-University of Glasgow Centre for Virus Research, Glasgow, UK; 892 Department of Medical Informatics and Clinical Epidemiology, Division of Bioinformatics and Computational Biology, OHSU Knight Cancer Institute, Oregon Health and Science University, Portland, OR USA; 893 School of Electronic Information and Communications, Huazhong University of Science and Technology, Wuhan, China; 894 Department of Applied Mathematics and Statistics, Johns Hopkins University, Baltimore, MD USA; 895 Department of Cancer Genome Informatics, Graduate School of Medicine, Osaka University, Osaka, Japan; 896 Institute of Computer Science, Heidelberg University, Heidelberg, Germany; 897 School of Mathematics and Statistics, University of Sydney, Sydney, NSW Australia; 898 Ben May Department for Cancer Research, University of Chicago, Chicago, IL USA; 899 Department of Human Genetics, University of Chicago, Chicago, IL USA; 900 Tri-Institutional PhD Program in Computational Biology and Medicine, Weill Cornell Medicine, New York, NY USA; 901 The First Affiliated Hospital, Xi’an Jiaotong University, Xi’an, China; 902 Department of Medicine and Therapeutics, The Chinese University of Hong Kong, Shatin, NT, Hong Kong China; 903 Department of Biostatistics, The University of Texas MD Anderson Cancer Center, Houston, TX USA; 904 Duke-NUS Medical School, Singapore, Singapore; 905 Department of Surgery, Ruijin Hospital, Shanghai Jiaotong University School of Medicine, Shanghai, China; 906 School of Computing Science, University of Glasgow, Glasgow, UK; 907 Division of Orthopaedic Surgery, Oslo University Hospital, Oslo, Norway; 908 Eastern Clinical School, Monash University, Melbourne, VIC Australia; 909 Epworth HealthCare, Richmond, VIC Australia; 910 Department of Biostatistics and Computational Biology, Dana-Farber Cancer Institute and Harvard Medical School, Boston, MA USA; 911 Department of Biomedical Informatics, College of Medicine, The Ohio State University, Columbus, OH USA; 912 The Ohio State University Comprehensive Cancer Center (OSUCCC – James), Columbus, OH USA; 913 The University of Texas School of Biomedical Informatics (SBMI) at Houston, Houston, TX USA; 914 Department of Biostatistics, University of North Carolina at Chapel Hill, Chapel Hill, NC USA; 915 Department of Biochemistry and Molecular Genetics, Feinberg School of Medicine, Northwestern University, Chicago, IL USA; 916 Faculty of Medicine and Health, University of Sydney, Sydney, NSW Australia; 917 Department of Pathology, Erasmus Medical Center Rotterdam, Rotterdam, GD The Netherlands; 918 Division of Molecular Carcinogenesis, The Netherlands Cancer Institute, Amsterdam, CX The Netherlands; 919 Institute of Molecular Life Sciences and Swiss Institute of Bioinformatics, University of Zurich, Zurich, Switzerland

**Keywords:** Cancer genomics, Computational biology and bioinformatics

## Abstract

The discovery of drivers of cancer has traditionally focused on protein-coding genes^1–4^. Here we present analyses of driver point mutations and structural variants in non-coding regions across 2,658 genomes from the Pan-Cancer Analysis of Whole Genomes (PCAWG) Consortium^5^ of the International Cancer Genome Consortium (ICGC) and The Cancer Genome Atlas (TCGA). For point mutations, we developed a statistically rigorous strategy for combining significance levels from multiple methods of driver discovery that overcomes the limitations of individual methods. For structural variants, we present two methods of driver discovery, and identify regions that are significantly affected by recurrent breakpoints and recurrent somatic juxtapositions. Our analyses confirm previously reported drivers^6,7^, raise doubts about others and identify novel candidates, including point mutations in the 5′ region of *TP53*, in the 3′ untranslated regions of *NFKBIZ* and *TOB1*, focal deletions in *BRD4* and rearrangements in the loci of AKR1C genes. We show that although point mutations and structural variants that drive cancer are less frequent in non-coding genes and regulatory sequences than in protein-coding genes, additional examples of these drivers will be found as more cancer genomes become available.

## Abstract (web summary)

Analyses of 2,658 whole genomes across 38 types of cancer identify the contribution of non-coding point mutations and structural variants to driving cancer.

## Main

Previous large-scale sequencing projects have identified many putative cancer genes, but most efforts have concentrated on mutations and copy-number alterations in protein-coding genes, mainly using whole-exome sequencing and single-nucleotide polymorphism arrays^1–4^. Whole-genome sequencing has made it possible to systematically survey non-coding regions for potential driver events, including single-nucleotide variants (SNVs), small insertions and deletions (indels) and larger structural variants. Whole-genome sequencing enables the precise localization of structural variant breakpoints and connections between distinct genomic loci (juxtapositions). Although previous whole-genome sequencing analyses of modestly sized cohorts have revealed candidate non-coding regulatory driver events^8–15^, the frequency and functional implications of these events remain understudied^6,7,13,16,17^.

Driver identification remains a far greater challenge in non-coding regions than in coding genes, owing to sequencing and mapping artefacts, poorly understood localized hypermutation processes^14,18,19^, incomplete annotation of regulatory regions, inaccurate estimation of the background mutation rate and the unknown functional effect of non-coding mutations. The discovery of drivers from structural variants is further complicated by their sparsity, the lack of obvious neutral events to build background models and their complex functional effects. Adequate statistical methods that address these issues are needed to reliably identify non-coding drivers.

The ICGC and TCGA PCAWG effort, which has collected and systematically analysed cancer genome sequences from 2,658 patients across 38 types of cancer^5^, offers an opportunity to characterize putative non-coding driver events that cannot be found using data from whole-exome sequencing or single-nucleotide polymorphism arrays. Here we describe a comprehensive search for non-coding somatic drivers. For point mutations (SNVs and indels), we combine results from multiple driver-discovery algorithms and, by carefully evaluating the significant hits, reveal that recurrent artefacts and poorly understood mutational processes have led to common false positives among previously reported non-coding drivers. For structural variants, we introduce two new methods for identifying both regions with significantly recurrent breakpoints (SRBs) and with significantly recurrent juxtapositions (SRJs), accounting for genomic heterogeneity in the rates of DNA break and repair and the three-dimensional architecture of the genome. Finally, to assess the potential for future non-coding driver discoveries, we quantify our statistical power in the PCAWG dataset and estimate the overall excess of point mutations in non-coding regulatory regions around known cancer genes.

## Hotspot mutations across cancer types

**Fig. 1:**
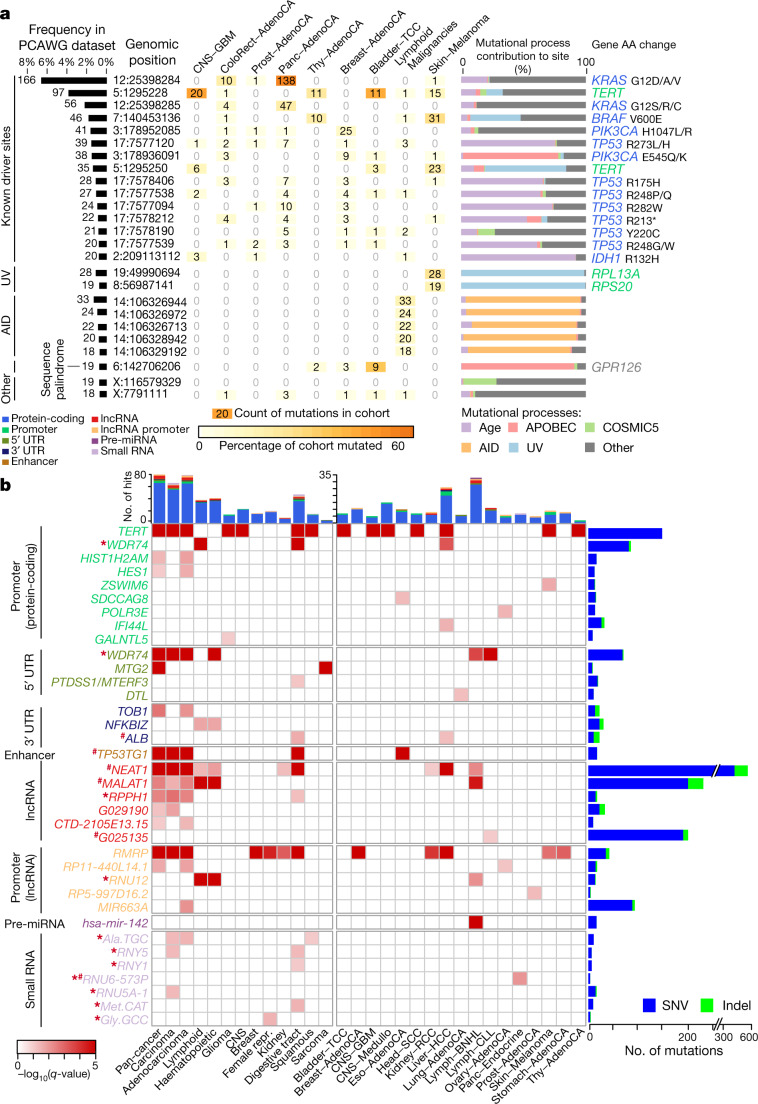
Non-coding point mutations in PCAWG. **a**, The bar chart (left) shows the total number of patients across PCAWG with mutations at a particular genomic hotspot (chromosome:position). The top 25 hotspots are grouped as known drivers or induced by mutational processes. The table (middle) shows the frequency of mutations across a subset of PCAWG cohorts. Lymphoid malignancies comprise Lymph–BNHL and Lymph–CLL. The stacked bar chart (right) shows the contribution of mutational processes to the hotspot mutations (Methods). Gene names are given when hotspots overlap functional elements (colour-coded), with amino acid (AA) alterations for protein-coding genes (solidus denotes substitution with any one of the indicated amino acids). Extended Data Fig. 1b shows the top 50 hotspots, and all cohorts. **b**, Significant non-coding elements (*Q* < 0.1 of Brown’s combined *P* values of up to 13 driver discovery methods; Methods) identified before manual review in cohorts with at least one hit. Colour represents significance levels. Details are provided in Supplementary Table 5. *Potential technical artefact; #targets affected by mutational processes. AdenoCA, adenocarcinoma; CNS, central nervous system; Eso, oesophageal; GBM, glioblastoma; HCC, hepatocellular carcinoma; Medullo, medulloblastoma; Panc, pancreatic; Prost, prostate; RCC, renal cell carcinoma; Repr., reproductive organs; SCC, squamous cell carcinoma; TCC, transitional cell carcinoma; Thy, thyroid. *HIST1H2AM* is also known as *H2AC17*; *Ala.TGC* as *TRA-TGC3-1*; *Met.CAT* as *TRM-CAT1-1*; and *Gly.GCC* as *TRG-GCC2-3*. *PTDSS1*/*MTERF3* denotes that 5′ UTR mutations in *PTDSS1* also overlap the *MTERF3* promoter.

**Extended Data Fig. 1:**
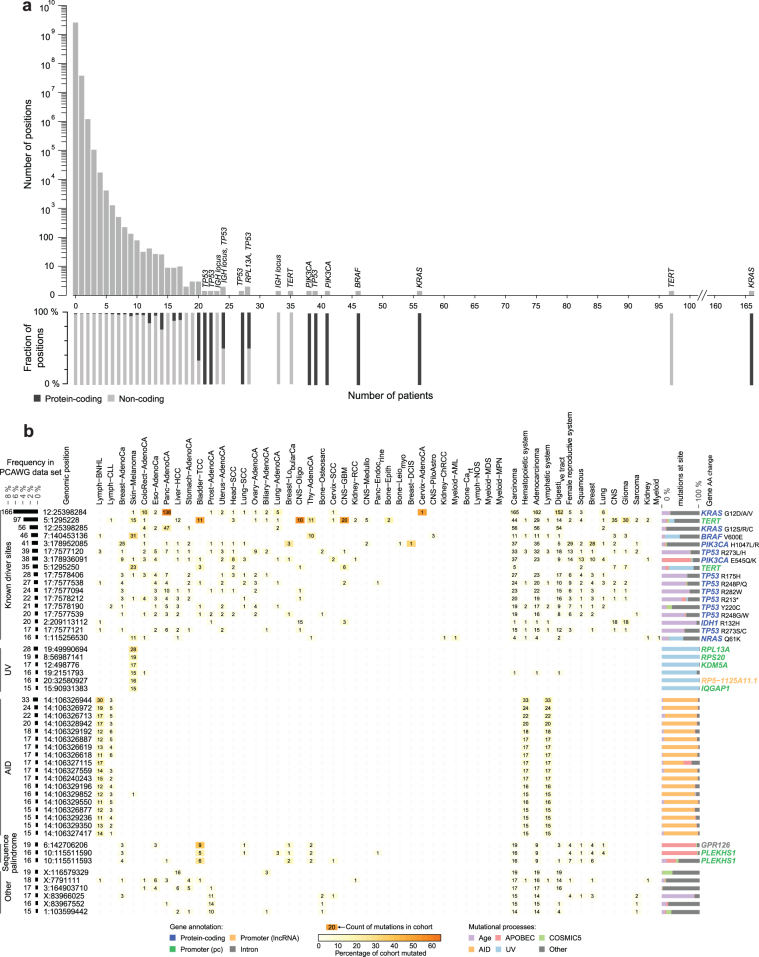
Mutational hotspots in additional tumour types. **a**, Bar plot of number of positions (*y* axis) mutated in *n*  patients (*x* axis). The stacked bar charts under the bar plot show the proportion of protein-coding (dark grey) and non-coding (light grey) positions. **b**, Distribution of SNVs in top 50 single-site hotspots across all analysed individual cohorts and meta-cohorts. Hotspots are grouped as known drivers or induced by mutational processes. The table (middle) shows the frequency of mutations across the PCAWG cohorts. Stacked bar chart (right) shows the contribution of mutational processes to the hotspot mutations (Methods). Gene names are given when hotspots overlap with functional elements (colour-coded), with amino acid alterations for protein-coding genes.

Many protein-coding driver mutations occur in single-site ‘hotspots’. In the PCAWG dataset, only 12 single-nucleotide positions were mutated in >1%, and 106 in >0.5%, of patients (Extended Data Fig. 1a, Methods). Although protein-coding regions span only about 1% of the genome, 15 out of 50 (30%) of the most frequently mutated sites were well-studied hotspots in cancer genes (*KRAS*, *BRAF*, *PIK3CA*, *TP53* and *IDH1*) (Fig. 1a, Extended Data Fig. 1b), along with the two canonical *TERT* promoter hotspots^6,7^.

The remaining non-coding hotspots could be attributed to the following localized mutational processes associated with passenger events: (i) damage from ultraviolet (UV) light and impaired nucleotide excision repair in melanoma at sites occupied by transcription factors^5,18–20^; (ii) somatic hypermutation by activation-induced cytosine deaminase (AID) in B-cell non-Hodgkin lymphoma (Lymph–BNHL) and chronic lymphocytic leukaemia (Lymph–CLL); (iii) palindromic sequence contexts believed to form hairpin DNA structures targeted by APOBEC enzymes (in an intron of *GPR126* (also known as *ADGRG6*) and the *PLEKHS1* promoter)^10^; and (iv) presumed technical artefacts (Fig. 1a, Supplementary Note 1). These findings suggest that—besides *TERT* promoter events—non-coding single-site hotspot drivers are infrequent or fall in regions with low sensitivity to detect mutations.

## Discovery of point-mutation drivers

**Extended Data Fig. 2:**
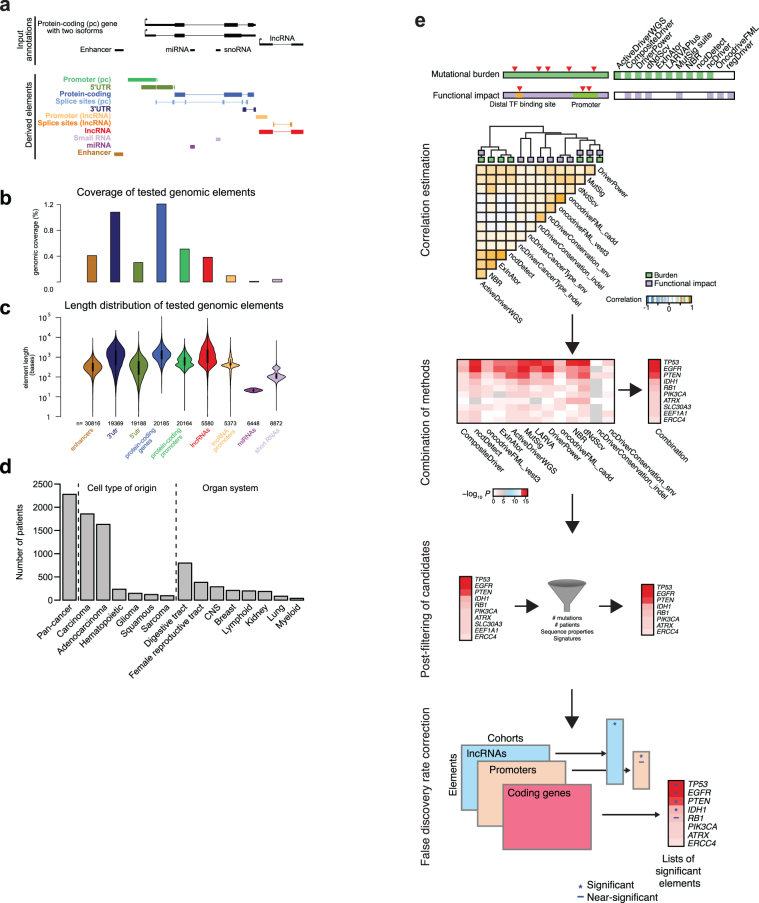
Element-based driver discovery and combination of *P* values. **a**, Schematic describing definition of types of functional element (Methods). Functional elements (black) are defined on the basis of transcript annotations from various databases. Elements arising from multiple transcripts with the same gene identity are collapsed, as seen here for the protein-coding isoforms. Promoter elements are defined as 200 bases upstream and downstream of the transcription start sites of the transcripts of a gene (green). Splice site elements extend 6 and 20 bases from the 3′ and 5′ exonic ends into intronic regions, respectively (light blue). Regions overlapping protein-coding bases and protein-coding splice sites are subtracted from other regions. **b**, Percentage of genomic coverage for each element type. **c**, Distribution of element lengths for each element type. Thick lines indicate interquartile ranges and short horizontal bars indicate the medians. **d**, Organization of meta-cohorts defined by tissue of origin and organ system. Pan-cancer contains all cancers, excluding Skin–Melanoma and lymphoid malignancies. **e**, Combination workflow: overview of methods of driver discovery and their lines of evidence to evaluate candidate gene drivers. Methods using each feature are marked with a box in the appropriate track. Heat map displaying Spearman’s correlation of *P* values across the different driver-discovery algorithms based on simulated (null model) mutational data. Dendrogram illustrates the relatedness of method *P* values, and algorithm approaches are marked by coloured boxes on dendrogram leaves. Next, *P* values are combined with Brown’s method on the basis of the calculated correlation structure. Individual method (left) and integrated (right) log-transformed *P* values are shown in a heat map (grey, missing data). Post-filtering used several criteria to identify likely suspicious candidates. Significant driver candidates were identified after controlling for multiple hypothesis testing based on an FDR *Q* value threshold of 0.1 (blue asterisk). Candidates with *Q* values below 0.25 (blue dash) were also considered of interest.

**Extended Data Fig. 3:**
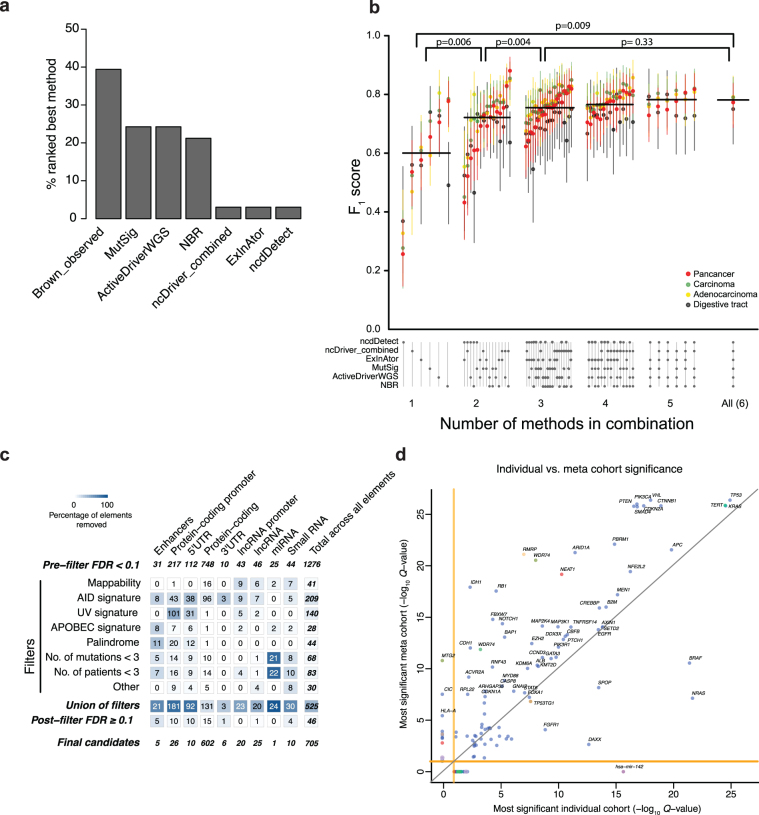
Sensitivity of driver-discovery methods and filter statistics. **a**, Percentage of coding-driver discovery runs (with stable F_1_ score, *n* = 33), across all cohorts, in which the method had the highest F_1_ score (Methods). **b**, F_1_ score of different methods of driver discovery, and different combinations evaluated in the four largest cohorts (pan-cancer (*n* = 2,278), carcinoma (*n* = 1,856), adenocarcinoma (*n* = 1,631) and digestive tract (*n* = 797)). Only methods that used the same algorithm to call coding and non-coding drivers were evaluated. Vertical lines indicate 95% confidence intervals. Horizontal black lines mark the median in each group. *P* values were calculated with the two-sided non-parametric Mann–Whitney *U* test. **c**, On top, the initial number of hits identified as recurrently mutated for each element type. The element types mature miRNA (*n* = 2 before filtering) and miRNA promoters (*n* = 16 before filtering) were omitted from the table. The heat map shows the number of hits filtered at each step in the sequential application of filters and post-filtering re-application of the FDR correction. Background colours indicate the corresponding percentage of input element removed. The final numbers of hits (including those that were later filtered by the comprehensive vetting procedures) are indicated below the heat map. **d**, Sensitivity versus specificity in individual cohorts versus meta-cohorts for candidate drivers: *Q* values for the most significant individual cohort (*x* axis) versus meta cohort (*y* axis) are shown. Driver elements are coloured by their element type. *Q* values derived from combination of *P* values from individual driver-discovery methods (Methods).

To identify recurrently mutated genomic elements, we first analysed somatic SNVs and indels in protein-coding regions, RNA genes (long and short non-coding RNAs and microRNAs (miRNAs)), and regulatory regions (promoters, 5′ untranslated regions (UTRs), 3′ UTRs and enhancers), totalling about 4% of the genome (Extended Data Fig. 2a–c, Methods, Supplementary Table 1). We analysed 2,583 tumours from 27 individual tumour types, and 15 meta-cohorts that grouped cancers by tissue of origin or organ system (Extended Data Fig. 2d, Methods). We identified candidate drivers—that is, cohort–element combinations with *Q* < 0.1 (10% false discovery rate (FDR))—by integrating 13 discovery algorithms, circumventing biases introduced by any one method (Extended Data Figs. 2e, 11, Supplementary Tables 2, 3, Supplementary Note 2). We benchmarked this approach by evaluating its ability to detect 603 known cancer genes (from the Cancer Gene Census (CGC)^21^, v.80), and found that combining methods improved performance compared to single algorithms (Extended Data Fig. 3a, b, Methods). Overall, we identified 1,294 significant hits that involved 520 unique candidates (Supplementary Tables 4, 5).

## Filtering the significant hits

Even after conservative FDR control, false-positive ‘driver’ loci can remain, owing to inaccurate background models, sequencing and mapping artefacts, or local increases in mutations due to unaccounted-for mutational processes. We therefore systematically filtered the candidate driver elements on the basis of technical and biological criteria, followed by careful review (Extended Data Fig. 3c, Methods, Supplementary Note 3). Examples of filtered elements include the promoters of *PIM1* (lymphoid tumours) and *RPL13A* (melanoma) because of associations with localized AID and UV-light mutational processes, respectively; *PLEKHS1*, *GPR126*, *TBC1D12* and *LEPROTL1* because of palindromic APOBEC target sequences^9,10^; and the *WDR74* 5′ UTR and promoter^8,10,14^, owing to mapping problems detected in downstream manual review (Supplementary Table 5, Supplementary Note 4). In combination, filtering and reapplying FDR control discarded 589 out of 1,294 (46%) of the original cohort–element hits and 341 out of 520 (66%) unique elements (Extended Data Fig. 3c, Supplementary Tables 4, 5).

## Candidate coding and non-coding drivers

Our stringent combination and filtering strategy yielded 705 hits in 179 genomic elements: 602 hits in 143 protein-coding genes and 103 hits in non-coding elements. We observed wide variability across different types of cancer, from one hit in clear-cell renal cancer to 80 in the pan-cancer meta-cohort (Fig. 1b, Supplementary Tables 4,5). Although most candidate drivers gained significance in larger meta-cohorts, some genes—such as *DAXX* (pancreatic endocrine tumour), *NRAS* (melanoma),* SPOP* (prostate adenocarcinoma), *FGFR1* (pilocytic astrocytoma) and *MIR142* (Lymph–BNHL)—scored higher in individual tumour types (Extended Data Fig. 3d). These results emphasize the trade-off between limiting driver discovery analyses to particular types of tumour and maximizing cohort size.

The candidate coding drivers we identified agreed with previous results: of the 143 genes that were significant in at least 1 cohort, 69% are in the CGC and nearly all have previously been implicated in cancer. In contrast to large whole-exome sequencing datasets, the fewer patients per cancer type in this dataset provided power sufficient only to detect genes with the strongest signal. We found 116 additional hits in 84 unique elements that were ‘near significance’ (0.1 < *Q* < 0.25). Fifty-one per cent of the 63 unique protein-coding genes in this set are in the CGC, which suggests that they would have been discovered in larger cohorts (Supplementary Table 4).

To nominate a significant non-coding element as a candidate driver, we reviewed the supporting evidence from the mutation calls, additional genomic data (chromosomal breakpoints, copy number, loss-of-heterozygosity and expression data), cancer gene databases and the literature (Methods, Supplementary Tables 6, 10). We describe the key candidates below, and in Supplementary Note 4.

**Extended Data Fig. 4:**
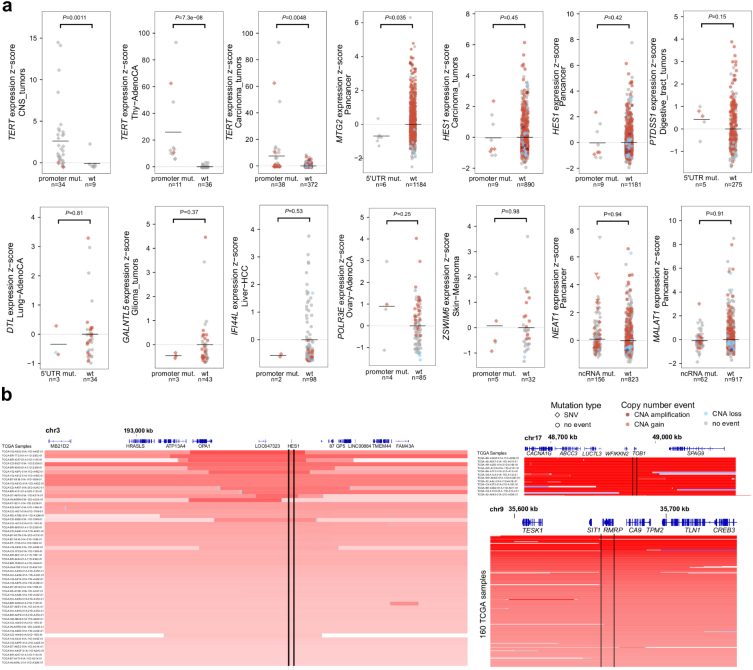
Mutation-to-expression correlation and focal copy-number alterations. **a**, Expression is compared between mutated and non-mutated samples. For each element, the *z* score of the expression values for mutated and wild type in the significant cohort is plotted. For copy number, CNA amplification indicates CNA > 10; CNA gain indicates CNA ≥ 3; CNA loss indicates CNA ≤ 1; and no events indicates CNA < 3 and CNA > 1. If a patient is mutated with multiple types of point mutation, indels are indicated over SNVs. For *TERT*, only samples powered to call mutation status were used. *P* values are based on a two-sided Wilcoxon rank-sum test. Bars indicate means. **b**, Copy-number profiles of 55 of 441 stomach adenocarcinomas from TCGA show copy-number gains around *HES1*. *TOB1* and its gene neighbour *WFIKKN2* are focally amplified in cancer (172 of 10,844 total samples from 33 cancer types are shown). *RMRP* focal amplifications in TCGA cancers (160 of 10,844 total tumours shown).

**Extended Data Fig. 5:**
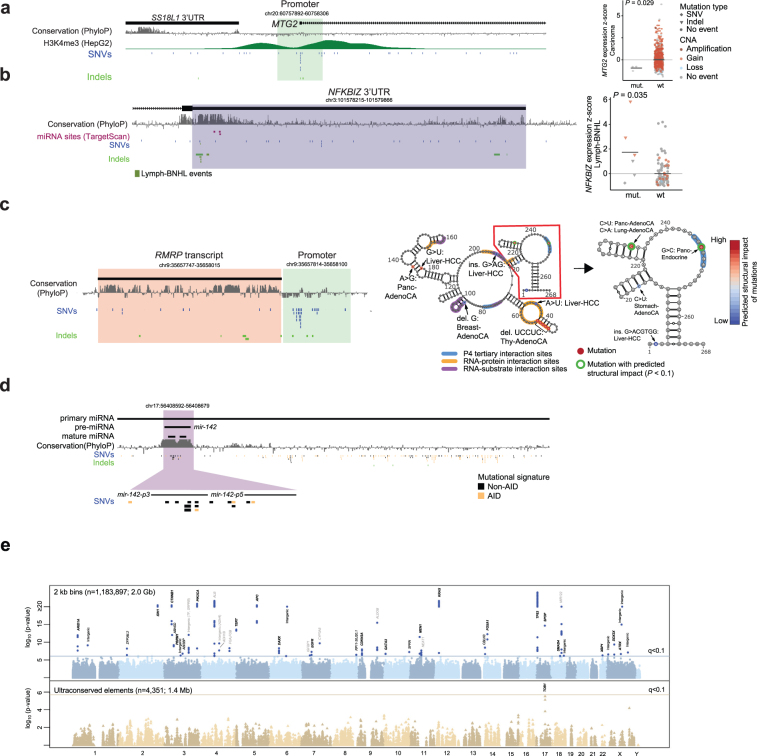
Non-coding driver candidates. **a**, *MTG2* promoter locus (left) and associated gene-expression changes in carcinoma tumours (right). Expression of *MTG2* in mutated (*n* = 3) versus the carcinoma meta-cohort wild-type cases (*n* = 896). Two-sided Wilcoxon rank-sum test. Bars represent means. **b**, Genomic locus of *NFKBIZ* 3′ UTR (left) and associated gene-expression changes in Lymph–BNHL (right). Expression of *NFKBIZ* in mutated (*n* = 6) versus wild-type cases (*n* = 98). Test and bars as in **b**. **c**, Genomic locus of the *RMRP* transcript and promoter region (left). RMRP is an RNA component of the endoribonuclease RNase MRP, the function of which depends on its RNA secondary and tertiary structure. The RNA secondary structure, tertiary structure interactions, protein and substrate interactions, and mutations with their predicted structural effect (right) of RMRP; lymphoma and melanoma mutations are excluded. **d**, *MIR142* locus and mutations in patients with lymphoma with the AID signature annotation. **e**, Manhattan-style plot showing significance of mutation recurrence enrichment for genomic bins (top) and ultraconserved elements (bottom) across cohorts (Methods; Supplementary Table 9).

The *TERT* promoter was the most frequently mutated non-coding driver in this dataset (14 cohorts) (Fig. 1b), and these mutations were strongly associated with higher *TERT* expression, as has previously been reported^9^ (Extended Data Fig. 4a, Supplementary Table 10). Mutations in the promoter and/or 5′ UTR of *MTG2* (which encodes a GTPase involved in the mitochondrial ribosome) were associated with an expression of *MTG2* that was marginally significantly lower, in both the pan-cancer (*P* = 0.036, fold difference = 0.8) and carcinoma (*P* = 0.029, fold difference = 0.8) meta-cohorts (Extended Data Figs. 4a, 5a). Mutations in the 5′ UTR have previously been shown to decrease *MTG2* expression in vitro^22^.

**Fig. 2:**
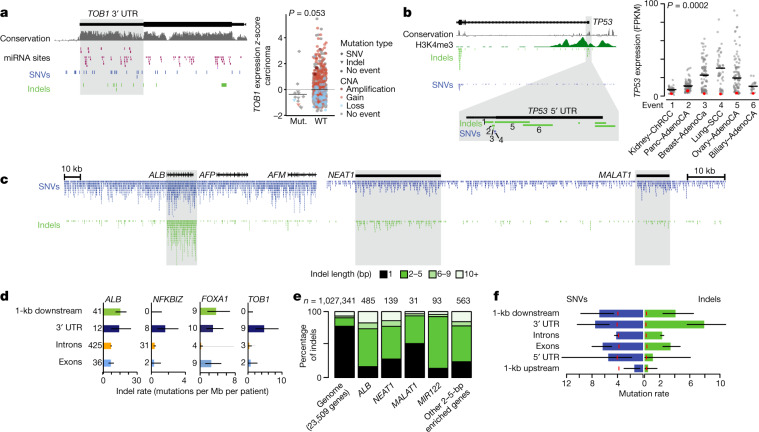
Newly identified non-coding driver candidates and localized transcription-associated mutational process. **a**, Recurrent mutations and associated gene expression in the highly conserved *TOB1* 3′ UTR. Tracks showing conservation score (PhyloP, grey), miRNA-binding sites (TargetScan (top track) and Ago-Clip (bottom track)), and observed SNVs (blue) and indels (green). Expression of *TOB1* in mutated (*n* = 13) and wild-type (*n* = 886) cases (right). *P* value based on two-sided Wilcoxon rank-sum test. Bars represent means. CNA, copy-number alteration. **b**, Indels and SNVs overlapping the *TP53* 5′ region and their effect on gene expression. H3K4me3 from the GM12878 cell line (ENCODE). Event numbers match with gene expression in the right panel (red dot, mutated sample; black bar, median). *P* value represents Fisher’s combination of permutation tests within each tumour type. ChRCC, chromophobe renal cell carcinoma; FPKM, fragments per kilobase of transcript per million mapped reads. **c**, Overall pan-cancer distribution of indels and SNVs in *ALB*, *NEAT1* and *MALAT1* genomic loci (lymphoid tumour samples were excluded owing to AID). **d**, Quantification of average indel rates for genes with significantly mutated 3′ UTRs. Error bars represent 95% binomial confidence intervals. **e**, Contribution of indels of different sizes in: all protein-coding and long non-coding RNA genes; *ALB*; *NEAT1*; *MALAT1*; *MIR122*; and the remaining genes enriched in 2–5-bp indels. **f**, SNV and indel rates (total events per Mb per patient) in different functional regions of 18 protein-coding genes enriched in 2–5-bp indels (without *ALB*, which contributed 47% of indels). Red lines indicate background indel and SNV rates estimated from all protein-coding genes. Error bars as in **d**; raw counts provided in Supplementary Table 18. **c**–**f**, Mutations analysed in all unique cases (*n* = 2,583).

Recurrent somatic events were identified in the 3′ UTRs of *TOB1* (carcinoma and pan-cancer meta-cohorts), *NFKBIZ* (lymphomas) and *ALB* (liver cancer) (Fig. 1b). *TOB1* encodes an anti-proliferation regulator that associates with ERBB2, and also affects migration and invasion in gastric cancer^23^. TOB1 regulates other mRNAs through binding to their 3′ UTR and promoting deadenylation^24^. Tumours with 3′ UTR mutations in *TOB1* showed a trend towards decreased expression (*P* = 0.053, fold difference = 0.7). The mutations did not concentrate in known miRNA-binding sites; however, the region is extremely conserved and thus probably functional (Fig. 2a). *TOB1* and its neighbouring gene *WFIKKN2* are focally amplified in breast cancer and pan-cancer, suggesting a complex role in cancer (Extended Data Fig. 4b). *NFKBIZ* is a transcription factor that is mutated in diffuse large B cell lymphoma and amplified in primary lymphomas^25^. Mutations in the 3′ UTR accumulated in a hotspot proximal to the stop codon and upstream of conserved miRNA-binding sites (Extended Data Fig. 5b). The enrichment of indels next to the stop codon suggests that this hotspot is not due to AID off-target activity. Previous functional experiments have associated these mutations with increased *NFKBIZ* expression^25^, which we observed in our lymphoma cohort (*P* = 0.035, fold difference = 3.2; after correction for copy number, *P* = 0.03) (Extended Data Fig. 5b).

Both the exon and promoter of the non-coding RNA *RMRP* were significantly mutated in multiple types of cancer (Fig. 1b, Extended Data Fig. 5c). Germline *RMRP* mutations cause cartilage–hair hypoplasia, and previous in vitro studies have shown that some somatic promoter mutations are functional^16^. The *RMRP* locus is also focally amplified in several types of tumour (Extended Data Fig. 4b). The enrichment of mutations in sites that can affect secondary structure suggests that these mutations are functional (*P* = 0.011, permutation test) (Extended Data Fig. 5c), although caution is required because this locus also appears to be affected by mapping artefacts or increased mutation rates (Supplementary Note 4).

The miR-142 precursor miRNA was significant in Lymph–BNHL and the lymphatic and haematopoietic cohorts (Fig. 1b; Extended Data Fig. 5d). The locus is a known AID off-target region in lymphoma^12,26^, but 7 out of 8  mutations in the mature miRNA *mir-142-p3*––for which the largest functional effect is expected––were not assigned to AID, which suggests that these mutations are under selection^12^.

## Unbiased genome-wide driver screen

To test whether we missed drivers by focusing on functionally annotated regions, we applied an unbiased genome-wide survey to all non-overlapping 2-kb windows for excess point mutations. Twenty-two of the resulting 67 significant windows overlap with known protein-coding drivers, and 28 overlap highly transcribed regions with an excess of 2–5-bp indels (described in the ‘Transcription-associated indel signature’ section below) (Extended Data Fig. 5e, Supplementary Table 9, Supplementary Note 5). The remaining 17 windows have no obvious link to cancer, and several appear to be affected by mapping artefacts. A separate analysis of 4,351 ultra-conserved non-coding regions did not yield new candidate drivers (Extended Data Fig. 5e, Supplementary Note 5). Both screens suggest that the paucity of non-coding point-mutation drivers found in this study is not due to the annotation of functional elements.

## Increasing power for known cancer genes

Finally, we performed restricted hypothesis testing to boost the statistical power to detect *cis*-regulatory driver mutations near cancer genes from the CGC^21^ (Supplementary Table 7). Restricted hypothesis testing of cancer gene promoters revealed a significant recurrence of *TP53* promoter mutations (11 patients in pan-cancer, *Q* = 0.044), mostly comprising SNVs and deletions that affect the transcription start site or donor splice site of the first non-coding exon. In 10 out of 11 cases, the mutation occurred in combination with loss-of-heterozygosity, and all samples with expression data showed decreased mRNA levels (Fig. 2b). None of these patients contained additional coding mutations that could instead be responsible for the downregulation of *TP53*. To our knowledge, this is the first report of a relatively infrequent—but impactful—form of *TP53* inactivation by non-coding mutations.

Focal gains or losses in cancer are selected for modulating expression levels of their target genes. Restricting the hypothesis testing to the non-coding elements of such genes (*n* = 216,986 cohort–element combinations, representing 5,201 unique elements) (Methods) yielded only one new hit, the 3′ UTR of the oncogene *FOXA1* in prostate cancer (Supplementary Table 11).

## Transcription-associated indel signature

**Extended Data Fig. 6:**
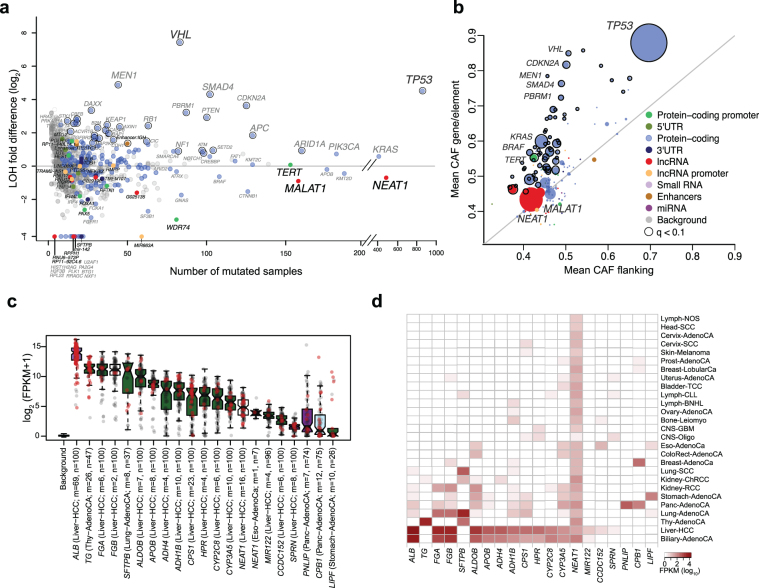
A transcriptional process creates passenger mutations in highly expressed, tissue-specific genes. **a**, Relative rate of loss-of-heterozygosity (LOH) compared between mutated and wild-type samples for all significant elements, coloured by element type and highlighting significant LOH enrichments with an outside black circle (Fisher’s exact test, one-sided; *Q* < 0.1). **b**, Average cancer allelic fraction (CAF) compared between each significant genomic element and the corresponding flanking regions (±2 kb and introns; overlapping coding exons were excluded). The size of the points represents the number of mutated samples for each particular element. Genes with significantly higher CAFs (*t*-test, one-sided; *Q* < 0.1) are highlighted with an outside black circle. **c**, mRNA expression of genes enriched in 2–5-bp indels in their respective tissues. Boxes show the interquartile range and median. The first box contains background gene-expression levels. Red and grey dots correspond to samples with (*m*) and without (*n* − *m*) indels in the corresponding gene. **d**, Heat map showing the levels of expression across types of cancer for the genes enriched in 2–5-bp indels.

Several significant non-coding elements (the *ALB* 3′ UTR, *NEAT1*, *MALAT1* and *MIR122*) were hit by many indels; all have previously been reported to be mutated in cancer^10,15,27^ (Figs. 1b, 2c). To explore whether *ALB* 3′ UTR events are under selection, we calculated indel rates across the functional regions of this gene. The indel rate is notably high throughout the UTRs, introns and exons, and even downstream of the polyadenylation site—a pattern inconsistent with selection (Fig. 2c, d). Similarly, *FOXA1* has high indel rates throughout its locus, whereas the indels in *NFKBIZ* and *TOB1* are in their 3′ UTRs, suggesting that these are driver events (Fig. 2d). *ALB*, *NEAT1* and *MALAT1* mutations were not associated with changes in gene expression (Extended Data Fig. 4a) and were not associated with high cancer cell fractions or biallelic loss (Extended Data Fig. 6a, b). Likewise, indels in *MIR122* were downstream of the mature miRNA, and were not associated with altered expression of the targets of this miRNA (Supplementary Note 5).

If the indels in these genes were due to a mutational process rather than selection, they might exhibit distinct features. Indeed, indels in *NEAT1*, *MALAT1*, *MIR122* and *ALB* were strongly enriched in 2–5-bp-long events (Fisher’s *P* < 6.8 × 10^−5^, for all) (Fig. 2e). A systematic search of coding and non-coding genes with significantly (*Q* < 0.1) increased rates of 2–5-bp indels revealed that this mutational process affects at least 18 additional genes in different types of tumour, most of which are highly expressed and tissue-specific (as has previously been reported for some of these genes^15^) (Extended Data Fig. 6e, f). Although less enriched, SNVs also occur at high frequencies in these regions (Fig. 2f). Overall, our findings suggest that the indels in *MALAT1*, *NEAT1*, *ALB* and *MIR122* are not driver events and are the result of a transcription-associated mutational process. The previously reported oncogenic effect of altered *MALAT1* and *NEAT1* expression^27–29^ may thus be unrelated to these mutations. Our findings also suggest that although *FOXA1* protein-coding indels are drivers, 3′ UTR indels might be passengers^30^.

## Breakpoints at driver and fragile sites

**Extended Data Fig. 7:**
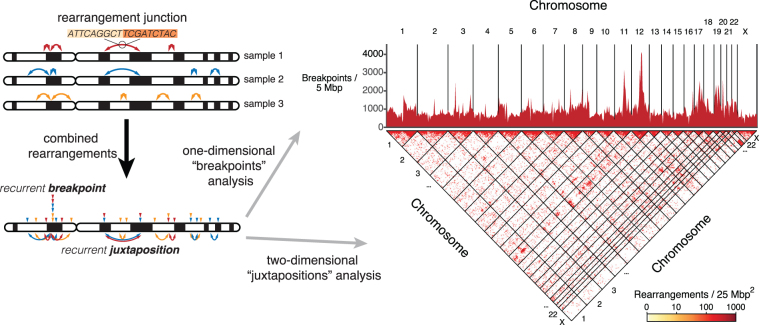
Overview of structural-variant analysis. Schematic indicating analysis approach. Left, rearrangements and rearrangement junctions in three hypothetical genomes (top) and the two analysis approaches (bottom): the 1D analysis for recurrent breakpoints and the 2D analysis for recurrent juxtapositions between pairs of loci. Right, the 1D density of breakpoints genome-wide (top) and 2D density of juxtapositions (bottom) across 2,693 cancer genomes (Methods).

Driver structural variants may act by disrupting one or both of their breakpoint loci (for example, deactivating a tumour suppressor), or by generating a novel juxtaposition between loci. We thus searched both for genomic regions with SRBs and for pairs of regions with SRJs (Extended Data Fig. 7).

**Fig. 3:**
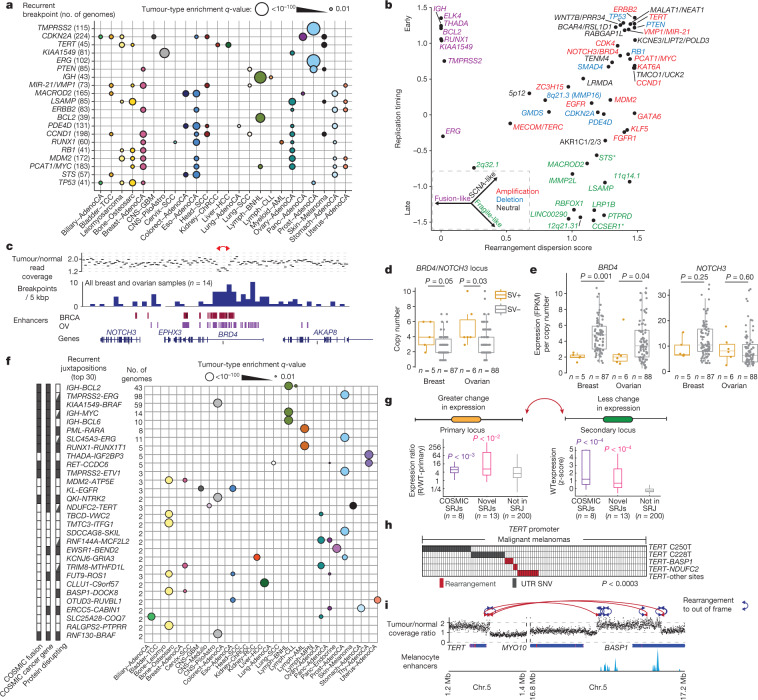
Significantly recurrent breakpoints and juxtapositions. **a**, Relative enrichment (Fisher’s exact test) for events per tumour type for the 20 most-significant SRBs (circle size). Loci are labelled by the likely driver gene from the CGC^21^. For gene symbols separated by a solidus, both or either of the genes are intended. **b**, Rearrangement dispersion score versus mean replication timing of the 53 SRBs. Colours indicate fusion (purple), fragile-like (green), deletion (blue), amplification (red) or copy-neutral (black) events. **c**, Tumour-to-normal read coverage ratio in an ovarian tumour with a *BRD4* microdeletion; red arrow indicates the rearrangement (top). Breakpoint density across PCAWG breast and ovarian cancers (middle). Enhancer locations from breast (BRCA) and ovarian (OV) tissue^51^ (bottom). **d**, Somatic copy number at the *BRD4* and *NOTCH3* locus in breast and ovarian cancers with (SV+) and without (SV−) rearrangements. **e**, Gene expression per absolute copy number for *BRD4* and *NOTCH3*. **f**, The 30 most-significant SRJs, with their relative enrichment (circle size) per tumour type, annotated with oncogenic fusions from the Catalogue of Somatic Mutations in Cancer (COSMIC) (left), CGC gene (centre) and protein disruption (right) (Methods). *ATP5E* is also known as *ATP5F1E*. **g**, Expression correlates of rearrangements in SRJs from COSMIC (purple), other SRJs (pink) or not in any SRJ (grey). For each rearrangement (R), the primary locus (left) is defined as the breakpoint within 100 kb of the gene that is most overexpressed in rearranged samples; the secondary locus (right) is the other breakpoint. Expression at the primary locus in samples with the rearrangement relative to samples without the rearrangement is greater for SRJs than for other rearrangements (left). The tissue-specific expression at the secondary locus in wild-type (WT) samples, relative to samples of different tissue types, is greater for SRJs than other rearrangements (right). *P* values represent comparisons to ‘not in SRJ’. **d**, **e**, **g**, Box plots show the interquartile range, median and 95% confidence interval; two-sided *t*-test. **h**, *TERT* promoter mutations and rearrangements across PCAWG melanomas. **i**, Rearrangements between *TERT* promoter and *BASP1* and *MYO10* locus result in focal amplification and relocation of distal enhancers to *TERT*. AML, acute myeloid leukaemia; Colorect, colorectal; Leiomyo, leiomyosarcoma; MPN, myeloproliferative neoplasm; Osteosarc, osteosarcoma; PiloAstro, pilocytic astrocytoma.

For SRBs, we first defined a background model to predict breakpoint density, using eight explanatory variables (Methods, Supplementary Table 13) and accounting for unexplained sources of variation^31^ (Supplementary Note 6). We identified 53 disjoint regions with SRBs (*Q* < 0.1) (Fig. 3a, Supplementary Table 14), which cleanly divided into two groups on the basis of the variability of the breakpoints at the other side of the rearrangements. Eight SRBs had partner breakpoints that were tightly clustered (had low rearrangement dispersion scores; Methods) and represented known oncogenic fusions. The remaining 45 SRBs had dispersed partner breakpoints (had high rearrangement dispersion scores), and were largely associated with previously identified somatic copy-number alterations (SCNAs) (Fig. 3b).

It has been difficult to distinguish recurrent driver SCNAs from passenger events at fragile sites^32^. At the resolution afforded by whole-genome sequencing, late replication timing predicted fragility-associated SRBs better than existing fragile site annotations (Supplementary Note 7), identifying 12 fragile-like SRBs (Fig. 3b). The remaining 33 SCNA-like SRBs comprised 14 amplifications, 8 deletions and 11 copy-neutral events (Supplementary Table 14).

**Extended Data Fig. 8:**
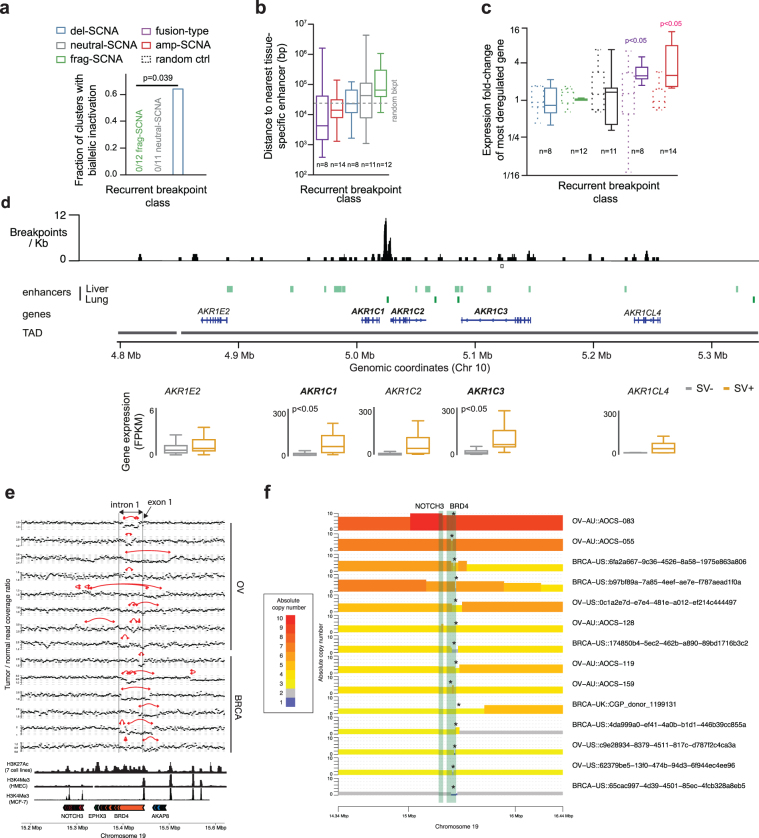
Gene-expression effects of SRBs. **a**, Fraction of recurrent breakpoint loci associated with biallelic inactivation of a known tumour suppressor gene (frag-SCNA, 0/12; neutral-SCNA, 0/14; del-SCNA, 5/8; Fisher’s exact test). **b**, Distance in bp to the nearest tissue-specific enhancer for each breakpoint class. Dashed grey line represents 1,000 randomly selected breakpoints from the same tumour samples. All box plots show the interquartile range, median and 95% confidence interval. **c**, Expression fold change for the gene with the most-altered expression within 1 Mb of the cluster centroid in samples with, compared to samples without, a breakpoint at the cluster locus. Random controls (in dashed boxes) represent 1,000 randomly selected breakpoints. *P* values are from two-sided *t*-tests (Methods). **d**, Breakpoint density near AKR1C genes (top), locations of enhancers (middle) and expression of local genes (bottom; *n* = 7 SV+ tumours, *n* = 41 SV− lung squamous cell tumours; two-sided *t*-test) in samples with and without local rearrangements. **e**, Ratio of tumour-to-normal read coverage across six breast tumours and eight ovarian tumours with focal *BRD4* exon 1 and intron 1 deletions. Red lines indicate rearrangements. **f**, Amplification structure (absolute copy number, *y* axis) of the *BRD4* and *NOTCH3* locus in breast and ovarian tumours with a *BRD4* focal deletion. In most cases, the copy-number caller identified the focal deletion. However, in some cases, the deletions were too small to be identified only using read depth. When combining read depth and rearrangement signals in **a**, there is clear evidence for focal deletions. Deletion locations are marked by an asterisk.

The different classes of SRB were associated with different effects on neighbouring genes. Five of the eight deletion-associated SRBs were associated with biallelic inactivation of nearby known tumour suppressors, compared to none of the 12 fragile-like SRBs (*P* = 0.039) (Extended Data Fig. 8a). The fragile-like SRBs were furthest from tissue-matched enhancers and caused the weakest expression changes, consistent with them being passenger events^32^. By contrast, fusion-like SRBs were closer to tissue-matched enhancers than the other SRBs (*P* < 0.01) (Extended Data Fig. 8b) and were associated with greater changes in expression than all other SRBs except amplifications (*P* < 0.05 for all types) (Extended Data Fig. 8c, Methods). Our analyses indicate that SRB driver events can be classified using rearrangement dispersion scores, replication timing and gene expression. Notably, neither rearrangement dispersion scores nor association with replication time can be accurately determined from microarrays or whole-exome sequencing, which highlights the importance of whole-genome sequencing. Altogether, we identified SRBs at 34 sites of known oncogenic fusions and recurrent SCNAs, 5 additional sites that are probably due to DNA fragility and 14 novel driver candidates (Supplementary Note 8).

## Novel structural-variant driver candidates

Although most SCNA-like SRBs act by altering gene copy numbers, several appeared to target regulatory elements. We identified three that were significantly (*Q* < 0.05) associated with expression changes of nearby genes after controlling for copy number (Methods), two of which we discuss here. The first comprised structural variants at 10p15, which were associated with a greater than twofold upregulation of *AKR1C1*, *AKR1C2* and *AKR1C3* in seven cases of lung squamous cell carcinoma and two cases of liver hepatocellular carcinoma (Extended Data Fig. 8d). AKR1C proteins are aldo-keto reductases involved in steroid homeostasis. Ectopic expression transforms cell lines, and germline mutations have previously been linked to an increased risk of developing lung cancer^33,34^. Three-quarters of the breakpoints are near (<10 kb) lineage-specific enhancers, potentially altering promoter–enhancer interactions (and hence gene expression). However, because the highest density of breakpoints lies between two long inverted repeats, the structural variants may have been induced by DNA secondary structure.

The second SRB contains recurrent microdeletions (<50 kb) involving the 5′ end of *BRD4* in ovarian (eight cases, *P* < 10^−7^) and breast tumours (six cases, *P* < 0.04) (Fig. 3c, Extended Data Fig. 8e). These deletions were highly enriched in cancers that amplified a segment that includes *BRD4* and *NOTCH3* (*P* < 0.004) (Fig. 3d, Extended Data Fig. 8f) but were not a direct consequence of these amplifications (Supplementary Note 9). *BRD4* is a chromatin regulator and a therapeutic target in several types of cancer^35,36^, including ovarian and triple-negative breast cancer^37,38^. Given the increased copy number of the full *BRD4* gene, we would expect increased gene expression. However, the microdeletions are associated with a lower expression of *BRD4* in breast (*P* = 0.001) and ovarian tumours (*P* = 0.04), but not of the neighbouring gene *NOTCH3* (Fig. 3e). The focal deletions in *BRD4* overlap a prominent exon-1 H3K4me3 peak and intron-1 enhancer elements in HMEC (normal breast) and MCF-7 (breast tumour) cells (Extended Data Fig. 8e), which suggests that these deletions disrupt regulatory elements. To our knowledge, this is the first evidence of a recurrent microdeletion limiting expression of an amplified gene.

## Recurrent fusions target gene regulation

**Extended Data Fig. 9:**
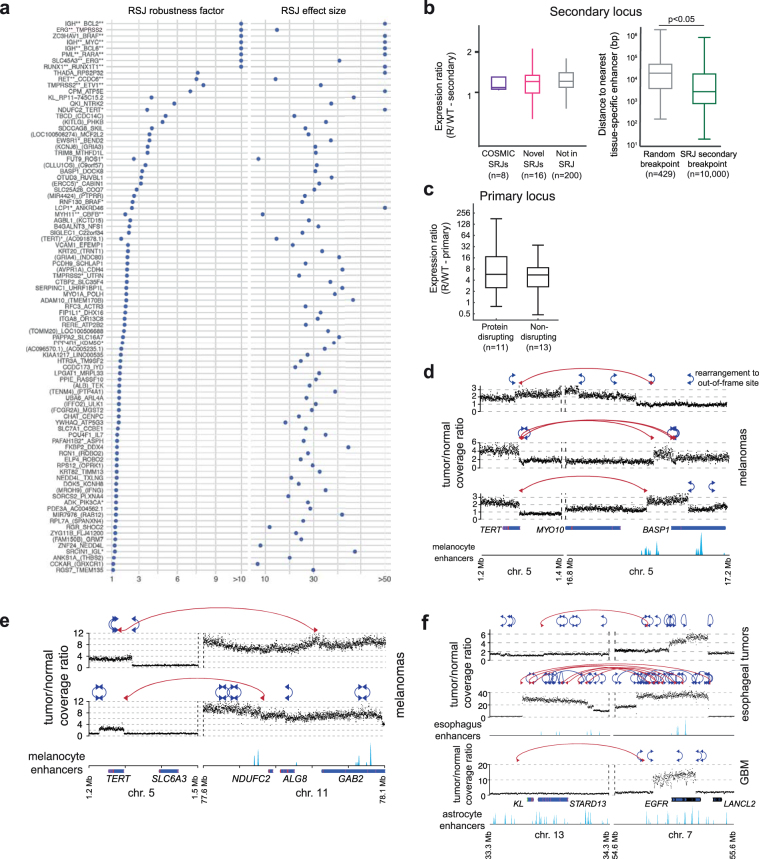
Gene-expression effects of SRJs. **a**, Assessment of SRJ robustness against unaccounted for mechanistic and technical confounders. Left, a robustness factor, defined as the ratio between the background probability value that would lower the *P* value of an SRJ below the genome-wide *P*-value threshold and the estimator for the background probability from our 2D model. Higher robustness values represent lower susceptibility to unaccounted variations in the background model. The top 48 SRJs have a robustness factor greater than 2, which suggests that these SRJs would remain significant even if the true background rate was twice as high as our model estimates. Right, the effect size is calculated as the difference in observed and estimated number of SRJs in units of standard deviation (assuming binomial distribution of structural variant count per 2D genomic region). Most SRJs are well above ten standard deviations of the predicted value. **b**, Characteristics of SRJ secondary loci. Left, fold expression enrichment of the most highly overexpressed gene in the secondary locus in cancer samples with these fusions relative to cancers of the same histology without the fusion. Right, the distance from the SRJ secondary locus (green) to the nearest enhancer is significantly smaller (*P* < 0.05; two-sided *t*-test) compared to randomly selected breakpoints (grey). **c**, Fold expression enrichment of the most highly overexpressed gene in the primary locus, for fusions that disrupt protein-coding sequences and fusions that do not. All box plots show the interquartile range, median and 95% confidence interval. **d**, Rearrangements between the *TERT* promoter and the *BASP1* and *MYO10* locus result in focal amplification of *TERT* and relocation of distal enhancers to *TERT*. **e**, *TERT-NDUFC2* fusion in two melanoma samples connecting *TERT* with an enhancer-rich region next to *NDUFC2*. Both samples also have focal amplifications of *TERT*. **f**, Recurrent translocation between *EGFR* in chromosome 7 and the *KL* and *STARD13* locus on chromosome 13. In all three samples, the rearrangement contributed to the amplification of *EGFR*.

Motivated by the detection of fusion-like SRBs, we specifically looked for genomic loci that were juxtaposed more often than expected by chance, after controlling for both the rate of breakpoints at each locus and the distance between them (Methods). We identified 90 such SRJs (Fig. 3f, Supplementary Table 15), including 13 known oncogenic fusions (including all 8 fusion-like SRBs) and 77 novel hits—18 of which linked to at least one known cancer gene (Supplementary Note 8). Previously reported oncogenic SRJs were observed more frequently (average 24 patients per fusion, range 2–98) than novel ones (most often 2 patients per fusion, range 2–4). As juxtapositions are unlikely to occur by chance, observing even two becomes highly significant. However, it is possible that some SRJs reflect inaccuracies in our background model rather than true drivers. We therefore further evaluated the SRJs on the basis of (i) a ‘robustness factor’ that indicates how much the background rate could increase before the SRJ would become insignificant, and (ii) the ratio between the observed and expected numbers of events under the current background model (‘effect size’) (Extended Data Fig. 9a). Twenty-six SRJs, including 11 of the 13 known drivers and 15 newly identified SRJs, are robust to tripling the expected background rate, and 22 others would remain significant with a doubled rate.

Most canonical driver rearrangements have previously been found in single tumour types, often associated with tissue-specific expression^39,40^. We found that 9 of our top 10 SRJs are tissue-specific, despite searching across 30 different types of tumour. Such tissue specificity is not observed for cancer genes affected by SCNAs, for which the top 10 are altered in 11.9 cancer types (on average), or by point mutations (for which the top 10 are altered in 6.7 cancer types, on average) (Supplementary Table 16).

The tissue specificity of SRJs suggests that they are strongly shaped by epigenetic state, either owing to mechanistic reasons (for example, tissue-specific three-dimensional proximity of the two DNA breakpoints) or to selection that connects tissue-specific regulatory elements with oncogenes^13,41–43^. The latter seems to be more likely because: (i) SRJs are associated with significant overexpression of only one of the rearrangement partners (the ‘primary locus’) relative to randomly selected rearrangements (primary locus, *P* < 10^−4^ (Fig. 3g left); secondary locus, *P* > 0.05 (Extended Data Fig. 9b left)); (ii) the rearrangement partner, in the secondary locus, tends to be highly expressed in that tissue type relative to others (Fig. 3g right); and (iii) the distance to the nearest tissue-specific enhancer is smaller for SRJs than for rearrangements overall (Extended Data Fig. 9b). These observations suggest that SRJs act in general by bringing regulatory elements to an oncogene that is otherwise expressed at a low level.

In many cases, SRJs generate truncated or chimeric proteins, and breakpoints within introns or exons were indeed overrepresented (68% versus 56% expected, *P* < 10^−7^). However, only 11 of the 30 (37%) most significant SRJs generated novel proteins in all samples, and 6 others sometimes generated novel proteins; the rest were either non-disruptive or contained breakpoints within the first two introns of the disrupted gene, leaving most of the protein intact^44^ (Fig. 3f). Moreover, SRJs that generate novel proteins exhibited expression changes similar to those that do not (*P* = 0.4) (Extended Data Fig. 9c). We conclude that altering gene expression is a key function of both classes of SRJs, and that SRJs are akin to non-coding driver point mutations that act on regulatory elements.

We found several SRJs that involve amplified oncogenes, including *MDM2*, *EGFR* and *TERT* (Fig. 3f, h, i, Extended Data Fig. 9d–f, Supplementary Table 15). The *TERT* promoter region was juxtaposed in four melanomas (*P* < 10^−7^) to a region in the *BASP1* gene (both on chromosome 5), and to a region near *NDUFC2* (t(5,11)) in two melanomas and one medulloblastoma (*P* < 10^−8^). Both juxtaposed regions were marked with melanocyte enhancers, which suggests that they could drive *TERT* expression. Among melanomas, these rearrangements are mutually exclusive with the C228T and C250T mutations of the *TERT* promoter (*P* < 10^−3^) (Fig. 3h). Because the juxtapositions were always part of complex events that also amplified *TERT*, increased *TERT* expression may be due to amplification, the juxtapositions or both.

## Paucity of non-coding drivers in cancer

**Fig. 4:**
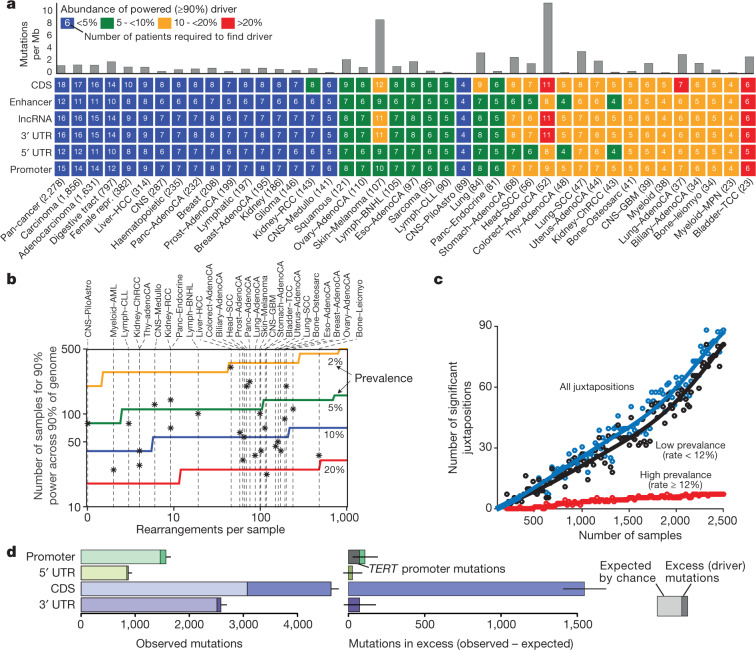
Power considerations and paucity of non-coding drivers. **a**, Heat map shows the minimal frequency of a driver element with ≥90% discovery power. Power is dependent on the background mutation frequency (above the heat map), the element length (median length depicted in Extended Data Fig. 2c) and the number of patients with mutations (cell numbers). For example, the pan-cancer cohort is powered to discover a protein-coding driver gene (coding sequence (CDS)) present in <1% (18 patients), whereas the Bladder–TCC cohort is only powered to discover drivers present in at least 27% (6 patients). **b**, Number of samples required to detect 90% of recurrent juxtapositions across 90% of pairs of loci, as a function of the median number of rearrangements per sample and the rate above background at which the fusion recurs (solid lines). The vertical dashed lines represent the median rearrangement rates of each cancer type, and the stars on these lines indicate the numbers of whole genomes analysed for that cancer type. **c**, Number of SRJs detected after downsampling the data to various sample sizes, separately indicating rearrangements that recur at high (≥12%; red) and low (<12%; black) rates above background; their sum (blue). **d**, Number of observed mutations (SNVs and indels) in *cis*-regulatory and coding regions of 603 protein-coding cancer genes with the expected numbers shown in lighter colours (left). Right, the number of excess mutations (that is, the estimated number of driver mutations) (right). The grey fraction of promoter mutations indicates *TERT* events. Error bars show 95% binomial confidence intervals. Only samples with high detection sensitivity were included (*n* = 936).

**Extended Data Fig. 10:**
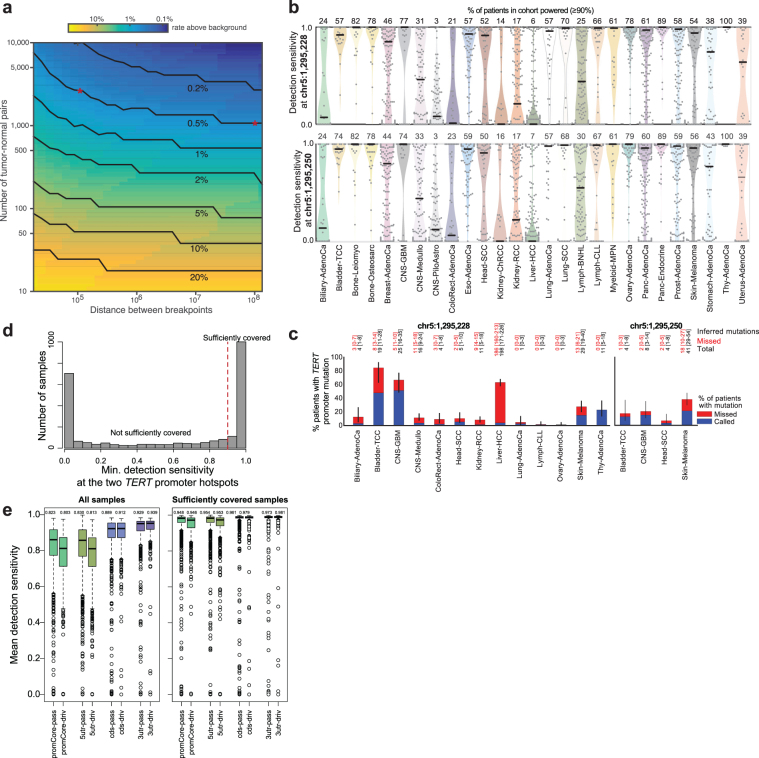
A lack of detection power in specific elements. **a**, Number of tumour–normal pairs needed to detect fusions with 90% power as a function of the span of the fusion and the rate above background at which it recurs. The red asterisks indicate the numbers of samples required to detect 100-kb and 100-Mb fusions that recur at 0.5% above their background rates. **b**, Distribution of *TERT* promoter hotspot (top, chromosome 5: 1,295,228; bottom, chromosome 5: 1,295,250; hg19) detection sensitivity for each patient, by cohort. Grey dots indicate values for individual patients inside estimated distribution (areas coloured by cohort). Horizontal black bars mark the medians. Numbers above distributions indicate the percentage of patients powered (detection sensitivity ≥ 90%) in each cohort. Cohort sizes as in Fig. 4a. **c**, Percentage of patients with observed (blue) and inferred missed (red) mutations at the chromosome 5: 1,295,228 and chromosome 5: 1,295,250 *TERT* promoter hotspot sites. Error bars indicate 95% Poisson confidence interval. Numbers above bars show the total inferred number of *TERT* promoter mutations for each site in this cohort. Red numbers indicate the absolute number of inferred missed mutations (owing to a lack of read coverage). Cohort sizes as in Fig. 4a. **d**, Detection sensitivity for the two *TERT* promoter hotspots across all samples showing the variation in powered samples. Red vertical line (*x* = 0.9) indicates cutoff for ‘sufficiently powered samples’. **e**, Mean detection sensitivity in 1,000 randomly selected putative passengers (pass) and 603 cancer genes (driv) across element types: promoters, 5′ UTRs, CDS and 3′ UTRs. The left panel shows the results for all samples and the right panel corresponds to the set of samples with high sensitivity at *TERT* hotspots. Boxes show the interquartile range and median; outliers are shown as circles. Weighted sensitivity means are shown at the top of the box plot.

**Extended Data Fig. 11:**
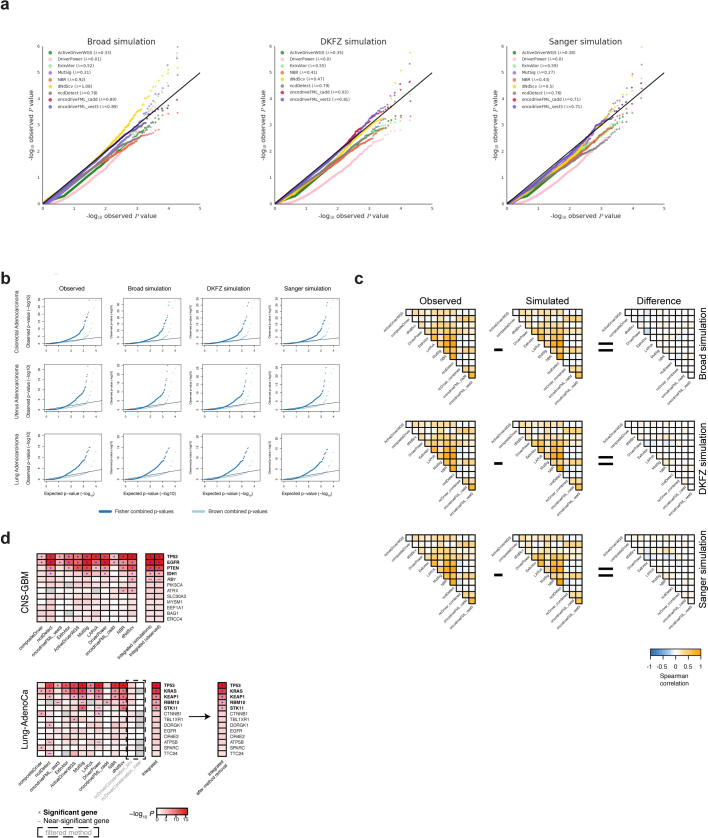
*P* value combination details. **a**, Quantile–quantile plots of *P* values reported by various driver-detection algorithms on the three simulated datasets (Broad, DKFZ and Sanger; shown for coding regions (*n* = 20,172) in the meta-carcinoma cohort; see Methods for details for the statistical background model or test of each algorithm) showed no major enrichment of mutations above the background rate. Results generally followed the expected null (uniform) distribution, and the *P* values reported on simulated data were subsequently used to assess the covariance of method results. **b**, Quantile–quantile plots of integrated *P* values using the Brown and Fisher methods for combining *P* values across the results from different driver-detection algorithms were generated for a few representative tumour cohorts (shown here for coding regions). Brown combined *P* values (light blue) generally followed the null distribution as expected, whereas Fisher combined *P* values were significantly inflated (dark blue), confirming that dependencies existed between the results reported by the various driver-detection algorithms. To simplify the integration procedure, we calculated covariances using *P* values from the observed data instead of simulated data and found that the integrated results based on the observed covariances (first column of plots) were essentially the same as the results obtained using the simulated covariances (second, third, and fourth columns of plots). **c**, Triangular heat maps showing the Spearman correlations of *P* values among the various driver-detection methods in observed versus simulated data (coding regions (*n* = 20,172), colorectal adenocarcinoma cohort) are highly similar. Differences in the observed and simulated correlation values (shown in the heat maps on the far right) were minimal, and thus the final integration of *P* values across methods was performed using covariances estimated on observed data. **d**, Brown combined *P* values based on observed and simulated covariance estimations (shown on the right, top heat map, for coding regions in glioblastoma) did not differ noticeably. In cases in which individual methods reported results that yielded substantially fewer hits than the median across all methods (bottom heat map, methods in light grey with results in dashed box), removing the methods from the integration did not affect the number of significant genes identified (right column of results in bottom heat map, shown for coding regions in lung adenocarcinoma). Number of coding regions as in **c**.

Our analyses of genomic hotspots, functional elements, genomic windows and SRJs all suggest that non-coding drivers are rare compared to protein-coding drivers. This might, in part, be due to a lack of discovery power^3^. We therefore evaluated the discovery power of mutational-burden tests for recurrent events across the different types of element in our tumour cohorts, focusing first on point mutations^3,16^. We found that the fraction of mutated patients required for a driver to reach 90% discovery power ranged from <1% in large cohorts with low background-mutation densities to 25% in small cohorts with high background-mutation densities (Fig. 4a). Different types of element were similarly powered, suggesting that the paucity of drivers in non-coding versus coding elements is not due to a lack of power. Similarly, our power to detect SRJs was higher in large cohorts with low rearrangement rates, and for long and interchromosomal rearrangements owing to their lower overall rates (Extended Data Fig. 10a): we were only powered to detect events that recur in 5–20% of samples in most types of cancer (Fig. 4b). Moreover, beginning with about 2,500 tumours, we expect to find a new SRJ with every 25 additional genomes (Fig. 4c).

Low sequencing coverage (for example, in GC-rich regions^45^) also limits driver discovery. To measure this effect in the PCAWG data, we quantified our ability to detect mutations (detection sensitivity)^16^ in cancer gene promoters. Although the mean detection sensitivity in promoters is high (41.9% of genomic positions have mean detection sensitivity >80% across tumours), only 4.1% of the promoters had detection sensitivity >90% in >90% of bases. In particular, the two canonical *TERT* promoter hotspots had highly variable detection sensitivity among patients and cohorts, from only 3% of patients in the central-nervous-system pilocytic astrocytoma cohort to 100% in the thyroid adenocarcinoma cohort (Extended Data Fig. 10b). From these data, we inferred the expected number of *TERT* events in each tumour type (Extended Data Fig. 10c) and found that about 263 (95% confidence interval 232–295) *TERT* hotspot mutations were probably missed owing to a lack of detection sensitivity. Moreover, on average 9.9% (1.3–13.0% interquartile range) of the cancer gene promoter territory in the tumour of each patient was severely underpowered (an average detection sensitivity of <10%). Therefore, the lack of coverage in promoters may contribute to the paucity of non-coding drivers.

To determine whether the paucity of non-coding drivers discovered thus far could be due to the limited statistical power of current datasets, we estimated the overall excess of point mutations above background (that is, the expected number of driver events) in coding and *cis*-regulatory non-coding sequences in 603 cancer genes^46^ (Methods, Supplementary Table 7, Supplementary Note 11). To minimize the effect of samples with low detection sensitivity, we included only 936 samples with >90% detection sensitivity at the two *TERT* promoter hotspots (Extended Data Fig. 10c, d, Supplementary Note 11). Overall, this approach predicted more than 1,475 driver mutations (95% confidence interval 1,410–1,687; 1,069 SNVs and 406 indels) in the protein-coding sequences of these cancer genes (Fig. 4d), compared to only 96 (95% confidence interval 30–190) estimated driver mutations in promoters (73 attributed to *TERT*), 22 (95% confidence interval 0–88) in 5′UTRs, and 68 (95% confidence interval 0–178) in 3′ UTRs. Non-coding mutations in cancer-gene promoters were also not generally associated with loss-of-heterozygosity or altered expression, as one would expect if they were enriched with drivers (Supplementary Note 12). These results collectively indicate that, independently of statistical power, non-coding *cis*-regulatory driver mutations in known cancer genes besides *TERT* are much less frequent than protein-coding drivers.

## Discussion

The accurate and reliable discovery of genomic drivers in tumours may have critical implications for patients with cancer. Our findings and the methods introduced here for the discovery of point-mutation and structural-variant drivers, method integration, vetting of candidates and identification of local hypermutation and fragile sites represent an important contribution to the collective effort towards charting all malignant changes that drive the cancer of each patient^5^.

Among the most interesting candidate non-coding driver elements we uncovered are the 5′-end mutations in *TP53*; 3′ UTR mutations in *NFKBIZ* and *TOB1*; and rearrangements involving AKR1C genes and *BRD4*. By careful analysis of the whole-genome sequencing data, we found that several previously reported and frequently altered non-coding elements may not be genuine drivers, including (i) the non-coding RNAs, *NEAT1* and *MALAT1* (which contain a high density of indels, seemingly owing to a transcription-associated mutational process) and (ii) recurrent structural variants in regions of late replication, indicating DNA fragility.

This study yielded unexpectedly few non-coding driver point mutations and structural variants. SRJs, which appear to act largely through the rearrangement of regulatory elements, are less frequent than SCNA-like SRBs, which directly amplify or delete coding sequences. The results from five analyses––hotspot recurrence, driver-element discovery, structural variants, discovery power and aggregated mutational excess––suggest that this paucity is not caused by a particular analysis strategy, but that regulatory elements truly contribute a much smaller number of recurrent cancer-driving events than protein-coding sequences. This paucity of non-coding drivers contrasts with the distribution of germline polymorphisms associated with heritability of complex traits, which are most frequently located outside of protein-coding genes^47^.

At least two factors contribute to the relative paucity of non-coding driver mutations in cancer: (i) the differential fitness effects of coding and non-coding mutations and (ii) the target size of functional elements. The paucity of promoter driver mutations in well-established cancer genes suggests that point mutations markedly affect the function of non-coding regulatory elements only rarely. This highlights *TERT* as a notable exception, perhaps because even a modest increase in *TERT* expression may suffice to circumvent normal telomere shortening. For other cancer genes, directly mutating protein-coding sequences or altering expression levels by copy-number change may provide larger phenotypic effects. For example, complete loss-of-function by nonsense mutations or deletions may be easier to achieve than by disrupting or translocating regulatory regions.

Technical shortcomings (such as coverage ‘blind spots’ in GC-rich promoters and different filtering strategies) may cause genuine drivers to be missed^48^. Therefore, the discovery of non-coding drivers will benefit from technical improvements, including even sequence coverage, longer and accurate reads, and improved variant-calling methods. Moreover, better annotation of functional non-coding elements will increase both the power to discover infrequently mutated driver elements and their interpretability. As datasets grow, yet-unidentified mutational mechanisms targeting particular genomic regions will emerge and require improved background models, including additional covariates and more-sophisticated statistical models. The analysis of structural variants has greater challenges because (i) accurately modelling their background density is complicated by their lower frequency and larger fraction of drivers (Supplementary Note 6); (ii) their target genes may be far from the breakpoints, as in SCNAs; (iii) the space for modelling SRJs is much larger (the genome squared); and (iv) many structural variants are part of complex events that often involve multiple chromosomes^31^, so that the resultant topology cannot be deduced without technologies such as long- or linked-read sequencing^49,50^. For these reasons, experimental validation remains important for all—and especially for non-coding—candidate drivers.

Our work suggests that larger datasets and technological advances will continue to identify new non-coding drivers, albeit at considerably lower frequencies than protein-coding drivers. We anticipate that the approaches developed here will provide a solid foundation for the incipient era of driver discovery from ever-larger numbers of cancer whole genomes.

## Methods

No statistical methods were used to predetermine sample size. The experiments were not randomized and investigators were not blinded to allocation during experiments and outcome assessment.

Detailed methods are provided as Supplementary Methods.

### Dataset generation

Out of 2,955 samples, we selected 2,583 unique donor samples for SNV and indel driver-discovery analysis on the basis of SNV quality control (Supplementary Methods). We found that 110 additional myeloid–AML samples had robust structural variant calls despite SNV artefacts; we included these in structural variant analyses, for a total of 2,693 samples. For tumour-type cohort analyses, we used only cohorts with at least 20 patients. Tumour meta-cohorts were defined by cell type of origin or by organ system (for example, lung for lung adenocarcinoma and lung squamous cell carcinoma). A pan-cancer meta-cohort was created by combining all tumour cohorts except for Skin–Melanoma and lymphoid tumours (Supplementary Methods).

### Hotspot SNV analysis

We selected the 50 most-frequent SNV hotspots. These were analysed to identify known driver events; mutational signature biases related to sequence palindromes, immunoglobulin loci and so on; and potential artefacts, including regional mapping problems (Supplementary Methods).

### Mutational signatures

We performed de novo global-signature discovery and signature attributions with SignatureAnalyzer’s Bayesian non-negative matrix factorization method^52^, based on 1,697 channels—including 1,536 pentanucleotide sequence contexts for single-base substitutions, 83 indel features, and 78 doublet-nucleotide substitution classes (Supplementary Methods).

### Definition of genomic elements

GENCODE v.19 (ref.^53^) and other genomic resources were used to define functional genomic elements, including protein-coding genes (CDS, splice sites, 5′ UTR, 3′ UTR and promoters), long non-coding RNAs (gene body, splice site and promoters), short RNAs, miRNAs and enhancers (Supplementary Methods).

### Candidate-driver-mutation identification methods and combination of results

We obtained results (*P* values) from 13 methods of driver discovery, including ActiveDriverWGS^54^, CompositeDriver, DriverPower^55^, dndscv^46^, ExInAtor^56^, LARVA^57^, MutSig tools^3^, NBR^10^, ncdDetect^58^, ncDriver^59^, OncodriveFML^60^ and regDriver^61^. We integrated the results of all these methods using a custom framework based on a previously published method^62^ for combining *P* values. Results from individual methods that showed large deviations from the expected uniform null distribution of *P* values were excluded. This approach was evaluated on real and simulated data. We controlled the FDR within each of the sets of tested genomic elements by concatenating all combined Brown’s *P* values from across all tumour-type cohorts and applying the Benjamini–Hochberg procedure^63^. Cohort–element combinations with *Q* values < 0.1 were designated as significant hits, and combinations with 0.1 ≤ *Q* < 0.25 as ‘near significance’. Extensive details are provided in the Supplementary Methods. In addition, we tested for element-independent recurrence with the NBR method on 2-kb bins spanning the entire genome, and non-coding ultraconserved regions^64^.

### Post-filtering of driver mutation candidates

We applied stringent filters to discern positive selection from technical artefacts and mutational processes. We required at least three mutations to be present in candidate elements, in at least three patients of the tested cohort; more than 50% of mutations in mappable regions; less than 50% of mutations in palindromic DNA; and less than 50% of mutations attributed to APOBEC activity. For lymphoid tumours and skin melanoma, we required that <35% and <50% of mutations were attributed to the AID and UV-light mutational signatures, respectively. The FDR was recalculated after post-filtering.

### Candidate driver structural-variant analyses

We applied separate analyses to detect recurrent structural variant breakpoints and recurrent juxtapositions. For each analysis, we first binned breakpoints, accepting only one breakpoint per sample per bin. We then determined which bins had more breakpoints than expected by chance (the SRB analysis), and which pairs of bins (or ‘tiles’) were joined by more rearrangements than expected by chance (the SRJ analysis).

### Candidate driver breakpoints

We calculated the background rate of breakpoints per bin based on a Gamma–Poisson model^15^ that took into account genomic covariates, breakpoint counts normalized by the number of bases within each bin that had sufficient mappability to be eligible for breakpoint detection and accounted for an observed overdispersion of breakpoint counts that probably reflects unaccounted-for covariates (Supplementary Methods). We used the Gamma–Poisson model to calculate the *P* value for each bin (that is, the probability that each bin would exhibit the observed number of breakpoints (or greater) by chance alone), applying the Benjamini–Hochberg procedure^63^ to correct for multiple hypotheses.

### Post-filtering of driver breakpoint candidates

We scored each recurrent breakpoint locus on the basis of the average replication timing of its breakpoints, and filtered those loci with scores >0.5 as probable fragile sites^65^.

### Candidate driver juxtapositions

We developed a background model to indicate the probability that two loci would be joined, taking into account the observed rate at which each locus underwent DNA breaks (from the breakpoint analysis), the distance between them and the propensity for these rearrangements to reflect a break followed by invasion versus two breaks that were then joined. We determined the probability that each tile would contain the observed number of rearrangements using a binomial test, followed by controlling for multiple hypothesis testing using the Benjamini–Hochberg procedure^63^.

### Gene-expression analyses

Gene-expression data were provided by the PCAWG Transcriptome Core Group^66^, and also generated using the same approach for an extended set of non-coding transcripts (Supplementary Methods).

### Additional evidence for selection

In addition to associations between mutations or structural variants and expression, we looked for signals of copy-number-alteration recurrence using the GISTIC2 algorithm^67^. We also tested whether driver candidates showed significantly higher frequency of loss-of-heterozygosity in mutated samples using Fisher’s exact test. We calculated cancer allelic fractions using ploidy and tumour purity predictions from a previous publication^68^.

### Mutational process and indel enrichment

For every gene, we calculated the proportion of indels of length 2–5 bp out of the total number of indels. This proportion was compared to the genome background proportion using a binomial test. We also compared the indel rate per gene (not distinguishing by length) to the background. Both sets of *P* values were corrected with the FDR method.

### Power calculations

We estimated our power to discover driver elements mutated at a particular frequency in the population as previously described^3,16^, but solving for the lowest frequency for a driver element in the patient population that is powered (≥90%) for discovery. The calculation of this lowest frequency takes into account (i) the average background mutation frequencies for each cohort–element combination; (ii) the median length and average detection sensitivity for each element type and patient cohort size; and (iii) a global desired false-positive rate of 10%. The effect of element length is discussed in Supplementary Note 10, and details are provided in Supplementary Methods. Power calculations for detection of recurrent juxtapositions was performed similarly, except over a two-dimensional genomic fusion map divided into 100 × 100-kb tiles (Supplementary Methods). We performed this analysis first as a function of the distance between breakpoints (Extended Data Fig. 10a) and second as a function of the median number of rearrangements per sample, spanning values represented by histologies with more than 15 samples (Fig. 4b).

### Estimation of the number of mutations in non-coding regions of known cancer genes

NBR was used to estimate the background mutation rate expected across cancer genes, using a conservative list of 19,082 putative passenger genes as background and including as covariates the local mutation rate, gene expression and averaged copy-number states. The resulting model predicted the number of passenger SNVs and indels expected by chance. By aggregating the expected numbers over 603 known cancer genes from the CGC^69^ (CGC v.80) (Supplementary Table 7), we compared the observed and expected numbers of mutations. For this analysis, we excluded samples with problems of low detection sensitivity (Supplementary Methods).

### Reporting summary

Further information on research design is available in the Nature Research Reporting Summary linked to this paper.

## Online content

Any methods, additional references, Nature Research reporting summaries, source data, extended data, supplementary information, acknowledgements, peer review information; details of author contributions and competing interests; and statements of data and code availability are available at 10.1038/s41586-020-1965-x.

## Supplementary information

Supplementary Methods This file contains the Supplementary Methods – see contents page for details.

Reporting Summary

Supplementary Information This file contains Supplementary Notes, including Supplementary Figures 1-15, a list of the Supplementary Tables (Supplementary Tables supplied as a separate spreadsheet), Supplementary references and a full list of participants in the PCAWG Consortium – see contents page for details.

Supplementary Tables This file contains Supplementary Tables 1-20.

## Data Availability

Data associated with this Article are available at https://dcc.icgc.org/releases/PCAWG/drivers. SRBs and SRJs are available at www.svscape.org. A list of data files used for analyses in this paper is provided in Supplementary Table 20. Somatic and germline variant calls, mutational signatures, subclonal reconstructions, transcript abundance, splice calls and other core data generated by the ICGC and TCGA PCAWG Consortium are described in an accompanying Article^5^, and are available for download at https://dcc.icgc.org/releases/PCAWG. Additional information on accessing the data, including raw read files, can be found at https://docs.icgc.org/pcawg/data/. In accordance with the data access policies of the ICGC and TCGA projects, most molecular, clinical and specimen data are in an open tier that does not require access approval. To access information that could potentially identify participants, such as germline alleles and the underlying sequencing data, researchers will need to apply to the TCGA data access committee via dbGaP (https://dbgap.ncbi.nlm.nih.gov/aa/wga.cgi?page=login) for access to the TCGA portion of the dataset, and to the ICGC data access compliance office (http://icgc.org/daco) for the ICGC portion of the dataset. In addition, to access somatic single-nucleotide variants derived from TCGA donors, researchers will also need to obtain dbGaP authorization.

## Code availability

The core computational pipelines used by the PCAWG Consortium for alignment, quality control and variant calling are available to the public at https://dockstore.org/search?search=pcawg under the GNU General Public License v.3.0, which allows for reuse and distribution. Code for *P* value combination from multiple driver methods is available from https://github.com/broadinstitute/getzlab-PCAWG-pvalue_combination/. Power calculation methods are available from https://github.com/broadinstitute/getzlab-PCAWG-power_calculations. Structural variant methods are located at https://github.com/mskilab/fishHook, https://github.com/walaj/ginseng and https://github.com/walaj/SVsig. Links to individual driver discovery methods are provided in the corresponding section of the Supplementary Methods.
