# Supplementary material for: Analyses of non-coding somatic drivers in 2,658 cancer whole genomes
Source: Nature. 2020 Feb 5;578(7793):102–11. doi: 10.1038/s41586-020-1965-x (PMC7054214; doi:10.1038/s41586-020-1965-x)
Supplement: Supplementary file 1 — This file contains the Supplementary Methods – see contents page for details. [file 41586_2020_1965_MOESM1_ESM.pdf]

nature

https://doi.org/10.1038/s41586-020-1965-x

Supplementary information

Analyses of non-coding somatic drivers in 2,658 cancer whole genomes

In the format provided by the authors and unedited

Supplementary Methods for "Analyses of non-coding somatic drivers in 2,658 cancer whole genomes" by Rheinbay, Nielsen, Abascal, Wala, Shapira et al.

1. Patient cohorts 1

2. Mutational hotspot analysis 3

3. Mutational signatures 4

4. Definition of genomic elements 6

5. Candidate driver identification methods 8

6. Simulated data sets 16

7. Statistical framework for the combination of results from multiple driver discovery methods 17

8. Post-filtering of candidates 20

9. Restricted hypothesis testing (RHT) 21

10. Sensitivity and precision analysis of driver predictions 22

12. Gene expression analyses 23

13. Normalization for copy number variation 24

14. Mutation-to-expression association 25

16. Power calculations 26

17. Associations between mutation and signatures of selection: loss of heterozygosity and cancer allelic fractions 27

19. Mutational process and indel enrichment 31

20. Structural variation analysis 31

21. RNA structural analysis 31

22. Cancer associated germline variant distance to non-coding driver candidates. 33

23. Assessing the significance of somatic rearrangement breakpoints 33

24. Classification of rearrangement patterns at sites of recurrent breakpoints 36

26. Annotation of potential functional effects of rearrangements 38

27. Power calculations for rearrangements 41

1. Patient cohorts

Generation of high-quality tumor set (Loris Mularoni)

We selected a total of 2,583 samples to be included in the point mutation driver discovery analyses. This list contains all the samples that were not flagged as problematic by the PCAWG Technical Working Group. A single aliquot was assigned to each sample; in cases where multiple aliquots were present, we selected a single aliquot based on the following criteria, in order of importance:

- we prioritized primary tumors over metastatic or recurrent tumors
- we selected aliquots with an OxoG score higher than 40
- we prioritized aliquots with the highest quality (as indicated by the Stars values)
- we prioritized aliquots with RNA-seq data availability
- we prioritized aliquots with the lowest contamination (as indicated by the ContEst values)
- if a selection could not be made after applying the above filters, we selected an aliquot randomly

Selection of tumor cohorts for analysis (Esther Rheinbay)

Individual tumor type cohorts from the high-quality tumor set were selected for analysis if they met a minimum size. This size was determined based on the cumulative number of patients, such that no more than 2.5% of total patients were excluded. This led to a minimum cohort size criterion of 20 patients, and removed the Bone-Cart (9 donors), Bone-Epith (11), Bone-Osteoblast (5), Breast-DCIS (3), Breast-LobularCa (13), Cervix-AdenoCa (2), Cervix-SCC (18), CNS-Oligo (18), Lymph-NOS (2), Myeloid-AML (13) and Myeloid-MDS (2) individual cohorts. Samples from these cohorts were still included in meta-cohort analysis (see below).

Tumor meta-cohorts (Esther Rheinbay)

Tumor meta-cohorts were assembled for identification of drivers and increase of discovery power across cell lineages and organ systems. The following meta cohorts were used in driver analyses:

By cell type of origin:

> **Epithelial: Carcinoma** (comprised of tumor cohorts Bladder-TCC, Biliary-AdenoCa, Breast-AdenoCa, Breast-LobularCa, Cervix-AdenoCa, ColoRect-AdenoCa, Eso-AdenoCa, Kidney-ChRCC, Kidney-RCC, Liver-HCC, Lung-AdenoCa, Ovary-AdenoCa, Panc-AdenoCa, Panc-Endocrine, Prost-AdenoCa, Stomach-AdenoCa, Thy-AdenoCa, Uterus-AdenoCa,Head-SCC, Cervix-SCC, Lung-SCC)**, Adenocarcinoma** (Biliary-

AdenoCa, Breast-AdenoCa, Breast-LobularCa, Cervix-AdenoCa, ColoRect-AdenoCa, Eso-AdenoCa, Kidney-ChRCC, Kidney-RCC, Liver-HCC, Lung-AdenoCa, Ovary-AdenoCa, Panc-AdenoCa, Prost-AdenoCa, Stomach-AdenoCa, Thy-AdenoCa, Uterus-AdenoCa), **squamous epithelium** (Head-SCC, Cervix-SCC, Lung-SCC)

Mesenchymal cells/sarcoma (Bone-Cart, Bone-Epith, Bone-Leiomyo, Bone-Osteosarc)

Glioma (CNS-PiloAstro, CNS-Oligo, CNS-GBM)

Hematopoietic system (Lymph-BNHL, Lymph-CLL, Lymph-NOS, Myeloid-AML, Myeloid-MDS, Myeloid-MPN)

By organ system:

Digestive tract (Liver-HCC, ColoRect-AdenoCa, Panc-AdenoCa, Eso-AdenoCa, Stomach-AdenoCa, Biliary-AdenoCa), **kidney** (Kidney-RCC, Kidney-ChRCC), **lung** (Lung-AdenoCa, Lung-SCC), **lymphatic system** (Lymph-BNHL, Lymph-CLL, Lymph-NOS), **myeloid** (Myeloid-AML, Myeloid-MDS, Myeloid-MPN), **breast** (Breast-AdenoCa, Breast-LobularCa), **female reproductive system** (Breast-AdenoCa, Breast-LobularCa, Cervix-AdenoCa, Cervix-SCC, Ovary-AdenoCa, Uterus-AdenoCa), **central nervous system** (CNS-PiloAstro, CNS-Oligo, CNS-Medullo, CNS-GBM)

Pan-cancer:

Two “Pan-cancer” cohorts were created: “Pancan-no-skin-melanoma” containing all tumor types with the exception of Skin-Melanoma to remove issues caused by very high mutation rate tumors; and “Pancan-no-skin-melanoma-lymph” with the additional removal of lymphoid tumors (Lymph-BNHL, Lymph-CLL, Lymph-NOS) that have local somatic hypermutation caused by AID.

2. Mutational hotspot analysis (Randi Istrup Juul)

We selected the top 50 single-position hotspots based on the number of patients with an SNV mutation. The individual positions marked as problematic by the site-specific noise filter (see below) analysis were excluded.

Each hotspot was defined by its genomic position and annotated by the number of patients with an SNV mutation in the given hotspot. We also annotated each hotspot with whether it fell into one of the genomic element types analyzed in the driver discovery. We further overlapped with loop-regions of palindromes, which are hypothesized to fold into DNA-level hairpins, and with

location in immunoglobulin loci. When a hotspot overlapped a protein-coding gene, we extracted the corresponding amino acids changes from Oncotator[1].

We identified known driver hotspots, by overlap with the somatic driver positions compiled in the Cancer Genome Interpreter repository (https://www.cancergenomeinterpreter.org/mutations), which among others include mutations from ClinVar, DoCM, and the literature[2].

For each hotspot, we calculated the proportion of mutations in the defined cohorts and meta-cohorts. Only cohorts with at least 20 patients, and at least 10 patients or 10% of patients with an SNV, were included in **Fig. 1a** (for the distribution in all cohorts and meta-cohorts, see **Extended Data Fig. 1b**). Lymph-BNHL and Lymph-CLL were shown together as Lymphoid malignancies.

Based on mutational signature analysis of all the cancer samples, we extracted the posterior probability that each hotspot mutation from a given patient was generated by one of 60 identified mutational signatures. In lymphoid malignancies, somatic hyper-mutations generated by AID come in clusters along the genome. Posterior probabilities for the ten signatures relevant for the lymphatic system cohorts were therefore derived from models that consider the correlation of AID mutations along the genome. For each hotspot, the collected posterior probabilities were averaged.

3. Mutational signatures (Jaegil Kim)

We performed a *de novo* global signature discovery to identify mutational signatures operating in PCAWG WGS cohort (n = 2,780). A total of 1,697 features or channels including 1,536 penta-nucleotide sequence contexts for single-nucleotide base substitutions (SBS), 83 insertion/deletion features (ID), and 78 doublet nucleotide substitutions (DBS) features, were ingested by *SignatureAnalyzer* exploiting Bayesian non-negative matrix factorization algorithm (NMF)[3,4,5] (COMPOSITE signatures[6]). A two-step signature extraction approach was applied to minimize "signature bleeding" or bias of hyper- or ultra-mutated samples on the signature extraction. In step 1, global signature extraction was performed for the low mutation burden samples (n = 2,624). These excluded hyper-mutated tumors with putative polymerase epsilon (*POLE*) defects or mismatch repair defects (microsatellite unstable tumors [MSI]), skin tumors (which had intense UV mutagenesis), and one tumor with temozolomide (TMZ) exposure. In step 2, additional signatures unique to hyper-mutated samples were extracted while allowing all signatures found

in the low mutation burden-samples to explain some of the spectra of hyper-mutated samples. Only signatures discovered in the low-mutation rate sample set were attributed to mutations in those samples. In contrast, mutations from hyper-mutated samples could be attributed to signatures discovered in either the low- or hyper-mutated sample set. Our *de novo* signature discovery extracted 35 COMPOSITE signatures covering most COSMIC signatures (https://cancer.sanger.ac.uk/cosmic/signatures) for non-hypermutated samples, including three APOBEC-related signatures (BI_COMPOSITE_SBS 2, 13, 69) and a split of COSMIC 17 (BI_COMPOSITE_SBS 17a and 17b). In addition, we extracted an additional 35 COMPOSITE signatures unique to hyper-mutated samples, including refinements of signatures. This included eight UV-related signatures (BI_COMPOSITE_SBS 7a, 7b, 7c, 38, 55, 65, 67, 75), five *POLE*-related signatures (BI_COMPOSITE_SBS 10a, 61, 62, 63, 66), and six MSI-related signatures (BI_COMPOSITE_SBS 6, 14, 15, 21, 26, and 73)[6].

A similar strategy was used for determining signature attributions; we performed a separate attribution process for low- and hyper-mutated samples using the COMPOSITE signatures. Signature attribution in low-mutation burden samples was performed separately in each tumor type (i.e., Biliary-AdenoCA, Bladder-TCC, Bone-Osteosarc, etc.). Attribution was also separately performed in MSI (n = 39), *POLE* (n = 9), skin (n = 107), and a single TMZ-treated tumor. In each separate cohort, the signature activity (e.g., which signatures are active or not) was primarily inferred through the automatic relevance determination process applied to the activity matrix H only, while fixing the normalized signature matrix, W. The attribution in low-mutation burden samples was performed using only signatures found in step 1 of the signature extraction. Two additional rules were applied in SBS signature attribution to enforce biological plausibility and minimize a signature bleeding; (i) allow signature BI_COMPOSITE_SBS4 (smoking signature) only in lung and head and neck cases; (ii) allow signature BI_COMPOSITE_SBS11 (TMZ signature) in a single GBM sample. This was enforced by introducing a binary, signature by sample, indicator matrix Z (1 - allowed and 0 - not allowed), which was multiplied by the H matrix in every multiplication update of H.

Local signatures analysis: We also performed a *de novo* local signature analysis in lymphoma samples (n = 197) to identify mutational signatures of the activation-induced cytosine deamination (AID). Since a majority of mutations from AID-related processes cluster near the *IgH* locus and several known off-target sites, we considered the clustering information of mutations as an additional feature in the signature discovery[3]. In brief, we first calculated, for each mutation, the

nearest mutation distance (NMD) to all other mutations on the same chromosome in the same patient. We then stratified mutations into two groups of ‘clustered’ (NMD ≤ 1kb) and ‘non-clustered’ mutations (NMD > 1kb). Next, the clustered and non-clustered mutation sets in each sample set were analyzed as a separate entity with the mutation count matrix of 1536-by-2N matrix, which enabled the discovery of mutational signatures unique to the clustered and non-clustered mutations. This analysis yielded ten mutational signatures, including two specific to clustered mutations (W3, W10). The profile of the signature W3 was characterized by predominant C>T/G at RCY motifs (R = purine, and Y = pyrimidine), resembling the known canonical AID signature, while the signature W10 had mostly T>A/C/G at TW motif (or A>T/G/G at WA) corresponding to the known hotspots of the non-canonical AID related to the error-prone translesion DNA synthesis. In addition, the signature profile of W7 was most similar to the previously identified AID signature (COSMIC9) but most mutations of W7 were scattered along the genome and not clustered. Here, we calculated AID activity as the sum of attributions in the three signatures: W3, W7, and W10.

4. Definition of genomic elements (Morten Muhlig Nielsen; Nasa Sinnott-Armstrong)

Elements pertaining to protein-coding genes (protein-coding promoters, 5’UTR, protein-coding sequence, protein-coding splice sites, and 3’UTR) were defined based on GENCODE annotations (v.19)[7]:

Coding elements (CDS): The set of coding bases collapsed across all coding transcripts with a given GENCODE gene ID.

Protein-coding splice site elements (pc_SS): Intronic regions extending six bases from donor splice sites and 20 bases from acceptor splice sites were collected for all coding transcripts.

Bases were collapsed across all coding transcripts with a given GENCODE gene ID. The global set of CDS bases were subtracted.

5'UTR elements (5UTR): The set of 5'UTR bases collapsed across all coding transcripts with a given GENCODE gene ID. The global set of CDS and pc_SS bases were subtracted.

3'UTR elements (3UTR): The set of 3'UTR bases collapsed across all coding transcripts with a given GENCODE gene ID. The global set of CDS, pc_SS and 5UTR bases were subtracted.

Protein-coding promoter elements (promCore): Regions extending 200 bases in both directions from all protein-coding transcripts' transcription start sites (5' ends). Bases were collapsed across all coding transcripts with a given GENCODE gene ID. The global set of CDS and pc_SS bases were subtracted.

lncRNA elements: lncRNA transcripts were defined based on annotations from GENCODE (v.19) and MiTranscriptome (v.2)[8] not overlapping GENCODE. Transcripts were included if fulfilling criteria 1-5 and 6 or 7 below:

1) No sense overlap to protein-coding gene regions
2) More than 5kb away from protein-coding genes on sense strand
3) Longer than 200 bases
4) Not annotated as the following biotypes: immunoglobulin, T-cell receptor, Mt_rRNA, Mt_tRNA, miRNA, misc_RNA, rRNA, scRNA, snRNA, snoRNA, ribozyme, sRNA or scaRNA.
5) Not overlapping genomic regions aligning back to the human genome (self-chained regions).
6) More than 20% of bases overlap conserved elements (except if annotated as pseudogene)
7) Expressed in more than 10% of PCAWG samples with RNAseq data

Genes corresponding to the selected transcripts were supplemented with a set of known functional lncRNA genes from the literature in addition to GENCODE annotated non-coding snoRNA and miRNA host genes. The elements were made by collapsing bases across transcripts with given gene ID. The global set of CDS, pc_SS, 5UTR, 3UTR, promCore and lncRNA_SS bases were subtracted.

lncRNA splice site elements (lncRNA_SS): Intronic regions extending six bases from donor splice sites and 20 bases from acceptor splice sites were collected for all lncRNA transcripts. Bases were collapsed across all lncRNA transcripts with a given gene ID. The global set of CDS, pc_SS, 5UTR, 3UTR and promCore bases were subtracted.

lncRNA promoter elements: Regions extending 200 bases in both directions from all lncRNA transcripts' transcription start sites (5' ends). Bases were collapsed across all lncRNA transcripts with a given gene ID. The global set of CDS, pc_SS, 5UTR, 3UTR, promCore and lncRNA_SS bases were subtracted.

Short RNA elements: Short RNA transcripts were defined based on annotations from databases Rfam (v.11)[9], tRNAscanSE (v.2.0)[10] and snoRNAdb (v.3)[11] in addition to GENCODE transcripts with biotype annotations mt_rRNA, mt_tRNA, misc_RNA, rRNA and snoRNA. Bases were collapsed across all smallRNA transcripts with a given gene ID. The global set of CDS, pc_SS, 5UTR, 3UTR and promCore bases were subtracted.

microRNA elements: Precursor and mature miRNAs were defined based on mirBase (v.20)[12] and a set of potential novel miRNAs[13]

Enhancer elements: Contiguous 15-state ChromHMM called enhancers correlated between H3K4me1 and RNA-seq across 57 human tissues were downloaded from Roadmap Epigenomics Consortium extended data[14]. Associated links, defined by co-occurring activity in a given cell type, were merged across cell types at FDR = 0.1. HoneyBadger2[15] p10 calls for all DNase I sites were filtered to peaks with signal strength 0.8 or greater and intersected with enhancer elements. The union of all DNase I peaks which overlapped with a given element, with all CDS regions filtered out, were used as the input to driver detection.

5. Candidate driver identification methods

A summary of approaches used by each method is listed in Supplementary Table 2.

ActiveDriverWGS (Juri Reimand)

Driver analysis with ActiveDriverWGS[16] was performed after discarding hypermutated samples (>90,000 mutations) from the PCAWG cancer cohort. To avoid leakage of signals from known cancer drivers, we removed missense mutations in analyses of non-coding regions. ActiveDriverWGS is a local mutation enrichment method for genome-wide discovery of cancer driver mutations with increased mutation burden of single nucleotide variants (SNVs) and indels. ActiveDriverWGS performs a model-based test whether a given genomic element is significantly more mutated than adjacent background genomic sequence (+/- 10kb and introns). Statistical significance of mutations is computed with a Poisson-linked generalised linear regression model. The null model treats all SNVs with trinucleotide context as cofactor, while indels are modelled with a separate cofactor for all nucleotides. Mutation counts per nucleotide are presented as the response variable. The alternative model tests whether the element has different mutation burden than the background sequence. The null and alternative models are compared with chi-square tests and confidence intervals of expected mutations were derived from the null model using resampling. If the confidence intervals indicated significant excess of mutation in the background and depletion in the element of interest, we inverted corresponding small *P* values (*P* = 1-*P*p if *P* < 0.5). Elements with no mutations were automatically assigned *P* = 1. Elements with no mutations were automatically assigned *P* = 1. ActiveDriverWGS is available in CRAN at https://cran.r-project.org/web/packages/ActiveDriverWGS/index.html

CompositeDriver0.2 (Eric Minwei Liu, Ekta Khurana)

We have developed CompositeDriver (https://github.com/khuranalab/CompositeDriver) – a computational method that combines signals of mutation recurrence and the functional impact score derived from FunSeq2 scheme[17] to identify coding and non-coding elements under positive selection[18]. CompositeDriver assigns a score to each region of interest (i.e., CDS, promoter, UTR, enhancer, or ncRNA) through summation of positional mutation recurrence multiplied by the functional impact score for all mutations within the region. A null CompositeDriver score distribution is built to calculate the *P* values for a region of interest. Mutations in the same element type but outside the region of interest are defined as background mutations. To build the null distribution, the same numbers of mutated positions are repeatedly drawn (default is 10^5 times)

from background mutations with similar replication timing and similar mutation context[19]. By drawing random mutations from the same element type, CompositeDriver incorporates DNase I hypersensitive sites and histone modification marks as covariates into the null model[20]. Finally, the Benjamini–Hochberg method is used for multiple hypothesis correction[21].

dNdScv (Inigo Martincorena)

dNdScv (https://github.com/im3sanger/dndscv) is a maximum-likelihood algorithm designed to test for positive or negative selection in cancer genomes or other sparse resequencing studies. dNdScv models somatic mutations in a given gene as a Poisson process, accounting for sequence composition and mutational signatures using 192 trinucleotide substitution rates. Mutation rates are also known to vary across genes, often co-varying with functional features of the human genome, such as replication time and chromatin state. This information is exploited by dNdScv to refine the estimates of the background mutation rate of each gene, using a negative binomial regression. This regression removes known sources of variation of the mutation rates and models the remaining unexplained variation of the mutation rate across genes as being Gamma distributed, which protects the method against overconfidence in the estimated background mutation rate for a gene. Overall, the local mutation rate for a gene is estimated accounting for mutational signatures in the samples analysed, the sequence composition of a gene in a trinucleotide context, 20 epigenomic covariates and the local number of synonymous mutations in the gene. Inferences on selection are carried out separately for missense substitutions, truncating substitutions (nonsense and essential splice site mutations) and indels, and then combined into a global *P* value per gene. dNdScv has been described in much greater detail elsewhere[22].

DriverPower (Shimin Shuai)

DriverPower is a combined burden and functional impact test for coding and non-coding cancer driver elements. In the DriverPower framework, randomized non-coding genome elements are used as training set. In total 1,373 reference features covering nucleotide compositions, conservation, replication timing, expression levels, epigenomic marks, and compartments are collected for downstream modelling. For the modelling, a feature selection step by randomized Lasso is performed at first. Then, the expected background mutation rate is estimated with selected highly important features by binomial generalized linear model. The predicted mutation rate is further calibrated with functional impact scores measured by CADD and Eigen scores. Finally, a *P* value is generated for each test element by binomial test with the alternative

hypothesis that the observed mutation rate is higher than the adjusted mutation rate. DriverPower is available at https://github.com/smshuai/DriverPower[23].

ExInAtor (Andres Lanzos; Rory Johnson)
ExInAtor (https://github.com/alanzos/ExInAtor) was specifically created for predicting cancer driver lncRNAs, but is agnostic to gene type and can also be used for protein-coding genes. The exons of each gene are identified and collapsed across transcript isoforms. For each gene, the trinucleotide content of the exonic region is calculated. The remaining intronic regions, along with 10 kb of sequence upstream and downstream, are defined as the background region. From this background, a new background region is created by randomly sampling the maximum number of nucleotides, such that the trinucleotide content exactly matches that of the exonic region. Next, the number of mutations in the exonic and sampled background regions are compared by hypergeometric test. Genes with elevated exonic mutational density are considered candidate driver genes. ExInAtor was used with a randomisation seed of 256. Otherwise, ExInAtor was run exactly as described in Lanzós et al.[24].

LARVA (Jing Zhang; Lucas Lochovsky, http://larva.gersteinlab.org/)
LARVA[25], or Large-scale Analysis of Recurrent Variants in noncoding Annotations, is a computational method that detects significantly elevated somatic mutation burdens in genomic elements — both coding and non-coding — to identify putative cancer-driving elements. Given a cancer cohort variant call set, and a list of genomic elements, LARVA models the expected background somatic mutation rate by fitting a beta-binomial distribution to the elements' variant counts. This model properly accounts for the high mutation rate variability seen throughout the genome, which improves over some previous models' assumption of a constant mutation rate. LARVA's model also incorporates the influence of mutation rate covariates, such as DNA replication timing. LARVA's output lists each genomic element from the input, along with a *P* value based on the deviation of the element's observed variant count from the expected variant count under LARVA's model.

MutSig (Julian Hess, Esther Rheinbay)
The MutSig suite[26] classifies whether genomics features, both coding and non-coding, are highly mutated relative to a predicted background mutation rate (BMR), which varies on a macroscopic-level across patients (patient-specific mutation rates can span orders of magnitude across pan-

cancer cohorts) and genes (known covariates such as replication timing are strongly correlated with mutation rate) as well as on a microscopic level across sequence contexts (since mutational signatures are heterogeneous across a cohort and highly context-dependent). MutSig accounts for all three of these to compute the joint BMR distribution across genes/patients/contexts, and then convolves across the latter two dimensions to estimate the expected distribution of total background burden for a given gene across a whole cohort. Genes are then scored by how their total non-background burden exceeds this null distribution. Furthermore, MutSig includes in the BMR calculation the number of bases in each feature that are sufficiently covered for mutation calling. This prevents underestimation of the BMR when a feature is only partially covered. Because this property sensitized MutSig to the randomizations performed without accounting for coverage, we removed from the analysis shown in **Extended Data Fig. 11** those CDS for which coverage was below 90% of the region.

MutSig estimates a gene's BMR by its synonymous mutation rate for coding genes, and by its mutation rate at non-conserved positions for non-coding genes. If the number of background mutations in a given gene is insufficient to provide a confident estimate of its BMR, MutSig will incorporate the background counts from other genes with similar covariate profiles into its estimator.

MutSig (MutSig2CV) was originally designed for coding regions only[26]. Modifications to this version of the algorithm to run on non-coding regions were made for this study's analyses of non-coding regions, as well as the sensitivity and benchmarking analysis in **Extended Data Fig. 3**. This version does not include significance evaluation based on functional impact or positional clustering, and is available from https://github.com/broadinstitute/getzlab-PCAWG-MutSig2CV_NC. The protein-coding MutSig2CV package, which includes tests for enrichment functional impact/clustering, was run on observed and simulated data for CDS.

NBR *(Inigo Martincorena)*

NBR (https://github.com/im3sanger/dndscv) is a method that tests for evidence of higher mutation density than expected by chance in a given region of the genome, while accounting for trinucleotide mutational signatures, sequence composition, and the local density of mutations around each element. This method has been described in detail in a previous publication[27], where it was used to identify candidate driver noncoding elements across 560 breast cancer whole-genomes.

Based on some of the features of dNdScv, NBR involves two main steps. First, all mutations across all elements tested are used to obtain maximum-likelihood estimates for the 192 rate parameters (r_j) describing each of the possible trinucleotide substitutions in a strand-specific manner. $r_j = n_j/L_j$, where n_j is the total number of mutations observed across samples of a given trinucleotide class (j), and L_j is the number of available sites for each trinucleotide. These rates are used to estimate the total number of mutations across samples expected under neutrality in each element considering the mutational signatures active in the cohort and the sequence of the elements ($E_h = S_j\ r_jL_{j,h}$). This estimate assumes no variation of the mutation rate across elements in the genome. Second, a negative binomial regression is used to refine this estimate of the background mutation rate of an element, using covariates and E_h as an offset. In this study, the local density of somatic mutations (normalized by sequence composition) was used as a covariate, using a window around the element of a variable size across cohorts to ensure sufficient numbers of mutations in each window around each element and excluding coding sequences and previously identified candidate noncoding driver regions. Replication time and average gene expression level for 100 kb genomic bins were also used as covariates. The negative binomial regression models mutation counts as Poisson-distributed within an element with mutation rates varying across elements according to a Gamma distribution. As in dNdScv, this provides a refined estimate of the background mutation rate for each element (E_h*) as well as a data-driven measure of uncertainty around this estimate (q, the overdispersion parameter of the negative binomial regression). *P* values for each element are calculated using a cumulative negative binomial distribution with the mean (E_h*) and dispersion (q) parameters estimated by the negative binomial regression.

To protect against neutral indel hotspots or indel artifacts, unique indel sites rather than total indels per element were used. To protect against misannotation of a mutation clusters as sets of independent events, a maximum of two mutations per region and per sample were considered in the analysis.

ncdDetect (Malene Juul)
ncdDetect[28] (http://moma.ki.au.dk/ncddetect/, https://github.com/MaleneJuul/ncdDetectTools) is a driver detection method tailored for the non-coding part of the genome. It uses a burden-based approach, in which the frequency of mutations is considered to reveal signs of recurrent positive selection across cancer genomes. For each candidate region, the observed mutation

frequency is compared to a sample- and position-specific background mutation rate. A scoring scheme is applied to further account for functional impact in the significance evaluation of a candidate cancer driver element. In the present application, the scoring scheme is defined as log-likelihoods, i.e., minus the natural logarithm of the sample- and position-specific probabilities of mutation.

The position- and sample-specific probabilities of mutation used by ncdDetect are obtained by a statistical null model, inferred from somatic mutation calls of a collection of cancer samples[29] (https://github.com/MultinomialMutations). The model includes a set of genomic annotations, known to correlate with the mutation rate in cancer. These are replication timing, trinucleotides (the nucleotide under consideration and its left and right flanking bases), genomic segment (a variable segmenting the genome into regulatory element types), and a position-specific measure of the local mutation rate (a weighted average of the mutation rate, calculated across samples in a 40 kb window flanking each specific position plus/minus 10 kb).

ncDriver (Henrik Hornshøj)
The *ncDriver* method[30] (http://moma.ki.au.dk/ncDriver/) provides separate evaluations of the significance for two mutation properties, the level of conservation, and the level of cancer type specificity. In the *ncDriverConservation* test, the conservation levels of mutated positions were evaluated *locally* for being surprisingly high, given the distribution of conservation within the element. The *P* value of the mean mutation phyloP conservation score for an element was obtained by Monte Carlo simulation of 100,000 mean phyloP scores based on the observedsame number of mutations. Each mutated element was also evaluated *globally* by looking up the rank of the element mean phyloP conservation score among all elements annotated as the same type. This provided *P* values for both *local* and *global* mutation conservation level, which were combined into a single conservation *P* value using Fisher's method[31]. In the *ncDriverCancerType* test, the distribution of observed mutation counts of an element across the cancer types were evaluated for being surprising compared to expected counts estimated from a background null model (as described for the *ncdDetect* method) that accounts for cancer type specific mutation signatures and other covariates. A goodness-of-fit test with Monte Carlo simulation was used to determine whether the distribution of observed mutation counts across cancer types within the element is surprising given the expected mutation counts based on cancer types, mutation contexts, and element type. For indels, the expected mutation counts were estimated solely from the mutation rates calculated from the

mutation context, cancer type, and element type. *P* values from SNV and indel runs were combined using Fisher's method[31].

OncodriveFML (Loris Mularoni)
OncodriveFML[32] (https://bitbucket.org/bbglab/oncodrivefml/src/master/) is a method designed to estimate the accumulated functional impact bias of tumor somatic mutations in genomic regions of interest, both coding and non-coding, based on a local simulation of the mutational process affecting it. The rationale behind OncodriveFML is that the observation of somatic mutations on a genomic element across tumors, whose average impact score is significantly greater than expected for said element constitutes a signal that these mutations have undergone positive selection during tumorigenesis. This, in turn, is considered as a direct indication that this element drives tumorigenesis.

OncodriveFML first computes the average functional impact score of the observed mutations in the element of interest. The functional impact scores of mutations have been calculated using both CADD[33] (coding and non-coding regions) and VEST3[34] (only coding regions). Then, the method randomly samples the same number of observed mutations following the probability of mutation of different tri-nucleotides, computed from the mutations observed in each cohort. The randomization step is repeated many times (1,000,000 in these analyses), and each time an average functional impact score is calculated. Finally, OncodriveFML derives an empirical *P* value for each element by comparing the average functional impact score observed in the element to its local expected average functional impact score resulting from the random sampling. The empirical *P* values are then corrected for false discovery rate, and genomic elements that remain significant after the correction are considered candidate drivers.

regDriver (Husen M. Umer)
regDriver (https://github.com/husensofteng/regDriver) assesses the significance of mutations affecting transcription factor motifs using tissue-specific functional annotations[35]. For each tumor cohort, functional annotations from the cell lines most similar to the respective tumor type are gathered. A functionality score is computed for each mutation based on its overlapping functional annotations. regDriver collects highly scored mutations in each of the defined elements and assesses the elements' significance by comparing its accumulative score to a background score distribution obtained from the simulated sets. Therefore, only candidate regulatory mutations are considered in evaluating mutation enrichment per element.

6. Simulated data sets

Broad simulations (Yosef Maruvka, Gad Getz)

Due to their differing context characteristics, we simulated SNVs and indels with different approaches. For SNVs, we divided the genome into 50 kb regions. For each region, we counted the number of mutations across all the PCAWG patients and divided this number by the total number of mutations. Every mutation was randomly assigned into a new region based on the region's rate. The position inside the region was chosen to maintain the trinucleotide context of each mutation (the 5' and 3' nearest neighbors and the mutated position itself) and the alternate allele. In addition, for every base, we counted how many times it was covered sufficiently in 401 tumor–normal WGS pairs, in order to enable calling of a mutation[36]. The fraction of patients with enough coverage at a given site was used as the position's probability for being mutated inside the new current region.

For indels, a new, randomized position was chosen in a region of 50 kb bases around the indel. The position of the new indel was chosen to match the indel 5' and 3' neighboring reference bases. For insertions, the inserted motif was the same as the original insertion; for deletions, however, only the length of the indels was kept, but not the exact sequence.

DKFZ simulations (Carl Hermann, Calvin Chan)

This simulation utilizes the SNV calls to perform a localised randomisation. The original SNV entries which do not map to chromosome 1–22, X, or Y are first filtered and excluded from randomization. All SNVs located in the protein-coding regions (CDS) corresponding to the GENCODE19 definition are erased before performing randomisation. The trinucleotide centered at each SNV position is determined, and an identical trinucleotide is randomly sampled within the 50 kb window. In the case of insertion, instead of the mutated trinucleotide, the neighboring nucleotide of the insertion site is scanned within the randomisation window. For deletion and multi-nucleotides variants, the altered sequence is scanned within the randomization window with a ranked probability assigned for each position. The randomised sample is then selected from the top 100 matched positions with scaled probability.

Sanger simulations (Inigo Martincorena)

This simulation aimed to generate data sets of neutral somatic mutations that retain key sources of variation in mutation rates known to exist in cancer genomes, including mutational signatures, and variable mutation rates across the genome and also among individuals and cancer types. To do so while minimizing the number of assumptions in the simulation, we used a simple local randomization approach. First, all coding mutations as well as mutations in the *TERT* promoter, *MALAT1,* or *NEAT1* were excluded. Second, each mutation in each patient was randomly moved to an identical trinucleotide within a 50 kb window, while retaining the patient ID. Third, mutations falling within 50 bp of their original position were filtered out. This simple randomization retains the variation of the mutation rate and mutational signatures across large regions of the genome, across individuals, and across cancer types.

7. Statistical framework for the combination of results from multiple driver discovery methods (Grace Tiao, Ziao Lin, Gad Getz)

The classical approach for combining *P* values obtained from independent tests of a given null hypothesis was described by R. A. Fisher in 1948. He noted that for a set of *k P* values, the sum *X* of the log-transformed *P* values, where

$$X = -2\ \Sigma^{k}_{i=1} \ln(p_i)$$

and p_i is the *P* value for the *i*th test, follows a chi-square distribution with 2*k* degrees of freedom[31]. Thus, to obtain a single combined *P* value for a set of independent tests, the new test statistic *X* is computed from the *P* values obtained from the tests and scored against a chi-square distribution with 2*k* degrees of freedom. Fisher's test is asymptotically optimal among all methods of combining independent tests[37]; however, in cases where tests exhibit positive correlation among the $\ln(p_i)$ values, the Fisher combined *P* value is generally too small (anti-conservative).

In this study, we combine *P* values from several driver detection methods, many of which share similar approaches and whose results are therefore not independent. To address this issue, we used an extension of the Fisher method developed by Morten Brown for cases in which there is dependence among a set of tests[37]. Using the same test statistic, renamed Ψ to indicate the

difference in the independence assumption, Brown observed that if Ψ were assumed to have a scaled chi-square distribution – i.e.,

$$\psi \sim c\, X^2_{2f}$$

then

$$f = E[\psi]^2/\text{var}(\psi) \text{ and } c = \text{var}(\psi)/\, 2E[\psi]$$

Note that $E[\Psi] = 2k$ irrespective of the independence requirement, and that

$$\text{var}(\psi) = 4k + 2\, \Sigma_{i<j}\, \text{cov}(-2\, \ln p_i, -2\, \ln p_j)$$

Thus when the p_i are independent, $\text{var}(\psi) = 4k$, which gives $f = k$ and $c = 1$, and the test statistic follows the chi-square distribution with $2k$ degrees of freedom described by Fisher. However, when the independence condition is relaxed, $\text{var}(\psi) \neq 4k$, and the test statistic generally follows a different, scaled, chi-square distribution whose scaling parameter c and degrees of freedom $2f$ are determined by the covariances of the p_i's. The covariances can be computed via numerical integration over the joint distributions of all p_i and p_j pairs, but this requires knowledge of the joint distribution; and even in cases where the joint distribution is known, the integration may not be computationally feasible for large and complex data sets[38].

In this study, following the example of Poole et al.[38], we computed the empirical covariance of p_i and p_j, using the samples $\boldsymbol{w_i}$ and $\boldsymbol{w_j}$, where $\boldsymbol{w_i}$ is the set of all reported P values for method i, and used the empirical covariance to approximate the Brown scaled chi-square distribution. The advantage to this approach is that the empirical covariance estimation is non-parametric – it does not assume an underlying joint distribution of p_i and p_j – and is thus applicable to complex and interrelated biological data sets where data is noisy and not regularly Gaussian. Poole et al. showed that the empirical covariance estimation approach is accurate, robust, and efficient for such data sets.

Implementing and evaluating the combination method on simulated and observed data

To evaluate the efficacy of the empirical Brown's method of dependent *P* value combination, we generated three sets of simulated mutation data (see above) and ran the driver detection algorithms on each of the simulated data sets. We checked that the *P* value results from the various driver detection algorithms followed the expected null (uniform) distribution (**Extended Data Fig. 11a**). Then, for each simulated data set, we calculated the empirical covariance for each pair of driver algorithm results. We then used these covariance values over simulated data sets to compute the combined Brown *P* values on observed data: for each gene in the observed PCAWG somatic mutation data set, we computed the Brown test statistic from the set of *P* values reported by the various driver detection algorithms. The Brown test statistic was then evaluated against the appropriate chi-square distribution, whose scale and degree parameters were approximated by the covariance values calculated on the simulated data (see above).

We ran this procedure, as well as the Fisher method, for six representative cohorts (three of which are shown: ColoRect-AdenoCa, Lung-AdenoCa, Uterus-AdenoCa) and found that the Brown combined *P* values generally followed the null distribution as expected (**Extended Data Fig. 11b**). The Fisher combined *P* values were significantly inflated (**Extended Data Fig. 11b**), confirming that dependencies existed between the results reported by the various driver detection algorithms.

To make this process more straightforward and reduce computation time, we explored whether computing the covariance values on observed data instead of simulated data would yield similar results. In each of the six representative cohorts, we calculated the empirical covariances on the observed data only and then computed the integrated Brown *P* values on the observed data using the observed covariances. Significant genes identified using only observed covariances remained mostly unchanged from the significant genes identified using the simulated covariances (**Extended Data Fig. 11d**), and examination of the differences in the covariance values between the simulated estimations and the observed estimations revealed only minor differences in values (**Extended Data Fig. 11c**). The significant drivers presented in this study were identified using this final approach – e.g., by computing integrated Brown *P* values using estimations of covariance on observed data only.

Combination of *P* values from observed data was performed for our 42 individual tumor-type and meta cohorts and 13 target element types. Methods were selected for each given data set (see below), and raw *P* values smaller than 10^{-16} were trimmed to that value before proceeding

with the combination. Methods with missing data for a given element (i.e., ones that failed to report a *P* value for a given element) were excluded from the calculation for that element, and therefore in some cases the integrated Brown *P* value was computed from *P* values reported by only a subset of all the driver detection algorithms contributing results for that data set.

Selecting methods to include in the combination of observed *P* values

In some cases, individual driver detection algorithms reported *P* values for a given data set that deviated strongly from the expected uniform null distribution. These were methods for which the quantile–quantile (QQ) plots demonstrated considerable inflation. We removed results that reported an unusual number of significant hits by calculating, for each set of results, the number of significant elements found by each individual method using the Benjamini–Hochberg FDR[21] with $Q < 0.1$ as the significance threshold. Any single method that reported four times the median number of significant elements identified by individual methods was discarded from the combination. In a separate analysis, we found that removing methods that yielded fewer hits than the median (i.e., methods with deflated QQ plots) did not affect the number of significant genes identified through the combination of the reported *P* values (**Extended Data Fig. 11d**); hence, we did not remove such methods.

8. Post-filtering of candidates (Esther Rheinbay, Morten Muhlig Nielsen, Lars Feuerbach, Henrik Tobias Madsen)

Post-filtering of significant hits was performed to remove those with accumulation of mutations caused by sequencing problems or mutational processes. In particular, we applied the following: (i) at least three mutations are present in the element, (ii) mutations are present in at least three patients of the tested cohort, (iii) less than 50% of mutations are located in palindromic DNA sequence[27], (iv) more than 50% of mutations are located in mappable genomic regions (CRG alignability, DAC blacklisted regions, and DUKE uniqueness[39]); and (v) manual review of sequence evidence for novel drivers. For lymphoid tumors, which contain regions of somatic hypermutation caused by AID enzyme activity, we (vi) further required less than 35% of mutations contributed by this process (signatures W3, W7, and W10, **Supplementary Fig. 1**); and for Skin-melanoma, we (vii) excluded elements with more than 50% of mutations belonging to the UV signature (BI_COMPOSITE_SBS signatures 7a_S, 7b_S, 7c_S, 38_S, 55_S, 65_S, 67_S and 75_S, and BI_DBS signatures 1_S, 13_S, 14_S and 15_S, **Supplementary Fig. 2**)[6,40–42]. For all tumor cohorts, we (viii) excluded elements with more than 50% of mutations attributed to APOBEC mutation signatures (BI_COMPOSITE_SBS signatures 2_P , 13_P and

69_P, **Supplementary Fig. 3**)[6]. For each of the signature dependent filters, we "rescued" elements found significant at the FDR < 0.1 level and passed all filters in at least one other cohort.

DNA palindromes

We define a palindrome as a sequence of DNA followed by its complementary reverse with a sequence of variable length in between. It is hypothesized that these palindromes can temporarily form DNA hairpins[43]. While in the hairpin state, the loop region is single-stranded and open to attack by APOBEC enzymes. Based on observations in breast cancer whole genome sequences[27], we decided to consider palindromes with a minimal repeat length of 6 bp and an intervening sequence (loop) length of 4–8 bp. We call these regions genome-wide using the algorithm described in Ye et al.[44], but using our own implementation (https://github.com/TobiasMadsen/detectIR). In total, we find 7.3 M palindrome regions covering a total of 135.2 Mb, of which 33.6 Mb are loop sequence.

Computing the false discovery rate

We controlled the false discovery rate (FDR) within each of the sets of tested genomic elements by concatenating all integrated Brown *P* values from across all tumor-type cohorts and applying the Benjamini–Hochberg procedure[21] to the integrated Brown *P* values. A *Q* value threshold of 0.1 was chosen to designate cohort-element combinations as significant hits. In addition, we defined cohort-element combinations in the range $0.1 \leq Q < 0.25$ as "near significance." We next applied several additional, mutation-based filtering criteria to each significant or near-significant candidate and assigned *P* values of 1 to candidates that failed these filtering criteria. Final Benjamini–Hochberg FDR values were then re-calculated on the adjusted sets of integrated Brown *P* values to arrive at a list of hits, ie. candidate driver cohort-element combinations.

9. Restricted hypothesis testing (RHT) (Ziao Lin, Esther Rheinbay, Federico Abascal, Iñigo Martincorena)

Localized amplification or deletion peaks were identified as copy number variant regions less than 1 Mb in size from GISTIC output files del_genes.conf_95.txt or amp_genes.conf_95.txt

(also see Section 15). 11,705 Gene-associated elements (promoter, UTRs, CDS, associated enhancers) located in these regions were used for RHT with the Benjamini–Hochberg FDR procedure[21] by target type. Significant ($Q < 0.1$) and nearly-significant hits ($0.1 \leq Q < 0.25$) for each target type were later concatenated (**Supplementary Table 11**). The indels impacting the 5'UTR of *TP53* were detected as a positive selection signal *T* with NBR doing RHT on the regulatory regions of 603 cancer genes.

10. Sensitivity and precision analysis of driver predictions (Ziao Lin, Iñigo Martincorena, Esther Rheinbay)

To evaluate the sensitivity and precision of different methods, and particularly of our approach for *P* value combination, we compared their relative performance in detecting known protein-coding cancer genes (603 genes from the manually curated Cancer Gene Census v80 database[45]). As the true set of cancer drivers to be discovered in any cohort is unknown, we defined the truth set as the union of significant CGC genes successfully identified by any single or combination of methods. For the negative set, we used all genes not in the truth set, which almost certainly contains yet-undiscovered cancer genes. Then, for each combination of methods, we intersected the predicted significant genes with the truth set (negative and positive, respectively) to calculate the F_1 score

$$F_1 = 2 * (precision * recall)/(precision + recall)$$

for each combination of methods. We ranked how individual methods performed based on their F_1 score in all protein-coding cohort runs, after removing cohorts with fewer than 5 true positives. In addition, we only included runs with stable F_1 scores and 95% confidence intervals <0.5 (see below), leaving 33 eligible cohorts. We used the unpaired Two-Sample Wilcoxon rank sum test to compare the F_1 scores between the different number of methods in combinations for the four largest cohorts because of their highest statistical power to discover drivers (Adenocarcinoma, Carcinoma, Digestive tract tumors and Pancan-no-skin-melanoma-lymph). Confidence intervals (95%) on F_1 scores were empirically estimated using 1,000 simulations of precision and recall values drawn from beta distributions. Methods were ranked based on cohorts with at least five genes in the positive truth set (see above), and F_1 scores with confidence interval size < 0.5.

11. Genome-wide driver discovery (Federico Abascal, Iñigo Martincorena)

To ensure that no highly recurrent genomic regions outside the defined functional elements were missed by our focused analysis, we searched for an excess of mutations in two additional element types: 1) non-overlapping 2-kb bins spanning the entire genome; and 2) 4,351 ultraconserved regions[46], of which only 36% overlapped with our functional element regions. We used the NBR negative binomial regression model described above, but without local mutation rate covariates because of problems arising in bins close to unmappable regions (e.g., peri-centromeres). To remove bins containing poor mappability regions, we calculated for each bin the number of bases matching the 1000 Genomes Project strict mask (ftp://ftp.1000genomes.ebi.ac.uk/vol1/ftp/release/20130502/supporting/accessible_genome_masks/) and/or simple repeats[47]. Only bins with less than 50% bases overlapping these tracks were kept for analysis. In total, we analyzed 1.2M bins covering ~66% of the genome. The set of ultraconserved regions (n = 4,351) covers a much smaller fraction of the genome (1.4 Mb, 0.05% of the total genome), but the shorter length of each region (mean of 325 bp) increases sensitivity to detect mutation recurrence. Genomic bin and UCNE analyses were done for all cohorts and meta-cohorts with a minimum of 100 samples, and excluding lymphoid tumors because of highly prevalent AID off-target activity. *P* values across all cohorts were globally corrected for multiple-hypothesis testing using the Benjamini–Hochberg FDR method.

12. Gene expression analyses (Samir B. Amin, Morten M. Nielsen, Andre Kahles, Nuno Fonseca, Lehmann Kjong, members of the PCAWG Transcriptome Working Group, and Jakob Skou Pedersen)

To extend the RNA-seq–based expression profiling of GENCODE annotations provided by the PCAWG Transcriptome Working Group[48], we profiled an extended set of gene annotations, including a comprehensive set of non-coding RNAs (described above and at https://dcc.icgc.org/releases/PCAWG/drivers/expression).

The profiling used a docker-based workflow for 1,180 RNA-seq donor libraries, matched to WGS data across 27 different cancer types[48]. In brief, raw sequence reads from donor libraries were uniformly evaluated for QC using FastQC tool, and subsequent alignment was performed

on QC-passed libraries using two methods: STAR (v2.4.0i)[49] and TopHat2 (v2.0.12)[50]. Resulting QC-passed bam files were independently used to quantify extended RNA-seq annotations at the gene-level counts using htseq-count method with following parameters: `-m intersection-nonempty --stranded=no --idattr gene_id`. This step resulted in two sets of gene-level counts files per donor library which were independently normalized using FPKM normalization and upper quartile normalization (FPKM-UQ). The final expression values were provided as a gene-centric table (rows as genes, columns as samples) with each value representing an average of the TopHat2 and STAR-based alignments FPKM values. Gene-centric tables based on both, GENCODE and extended RNA-seq annotations are available at https://dcc.icgc.org/releases/PCAWG/drivers/expression). Docker-based workflow for quantifying extended RNA-seq annotations is at https://github.com/dyndna/pcawg14_htseq.

13. Normalization for copy number variation (Henrik Tobias Madsen, Morten Muhlig Nielsen, Jakob Skou Pedersen)
To account for the effects of somatic copy number alterations (CNAs) on expression, we used two different approaches to create two additional versions of the expression profiles. First, we used a conservative approach wherein we remove all samples not having the regular bi-allelic copy number for the gene in question. Second, we used a less conservative approach, wherein we first built a regression model of expression data based on copy number (CN) data and then tested for an effect of somatic mutations on the residual (i.e., the expression that is not explained by copy number).

Generally, the higher the copy number of a particular gene, the higher its expression. The relationship between copy number and gene expression is not strictly linear, as various feedback mechanisms in the cell try to compensate for the mostly deleterious effects of CNAs. This is known as dosage compensation and has been studied extensively in the context of mammalian sex chromosomes, but also in evolution of yeast and in diseases caused by aneuploidy[51–54]. We therefore fit a linear regression model between the logarithm of expression and the logarithm of CN. This effectively amounts to a power-regression model.

A number of factors makes it difficult to learn the regression parameters for each gene and cancer type in isolation: (i) for some cancer types, we have only a limited number of samples; (ii) for some genes, there is not much variation in CN; and (iii) the variation in expression

between samples is generally high. We overcome these problems by employing a mixed model strategy that allows sharing of information between genes, effectively regularizing the parameter estimates for gene/cancer-type combinations that carry little information on their own.

Let $FPKM_{g,c,i}$ and $CNA_{g,c,i}$ denote the expression and CNA measurement respectively for gene $g$in cancer type c and sample i respectively. We then define:

$$logFPKM_{g,c,i} = \alpha_{g,c} + (\beta + \gamma_g + \lambda_{g,c})logCNA_{g,c,i} + \epsilon_{g,c,i}$$

where $\alpha_{g,c}$ and β are fixed effects, whereas γ_g and $\lambda_{g,c}$ are random effects, with $\gamma_g \sim \mathcal{N}(0, \sigma_\gamma^2)$ and $\lambda_{g,c} \sim \mathcal{N}(0, \sigma_\lambda^2)$. Finally the residual is $\varepsilon_{g,c,i} \sim \mathcal{N}(0, \sigma_\varepsilon^2)$.
Using this model we infer a global CNA to expression regression, β, but allow some regularized gene-specific and gene/cancer type-specific variation: γ_g and $\lambda_{g,c}$.
Thus, we exploit the similarity across genes and similarity within genes across cancer types.

Since the variance increases with the absolute value of the explanatory variable associated with a random slope, this kind of mixed model display heteroskedasticity. Furthermore, the model is not invariant under scaling of the explanatory variable, in this case CNA. We centralise log(CNA) such that normal diploid regions have the least variance.

14. Mutation-to-expression association (Morten Muhlig Nielsen, Henrik Tobias Madsen, Jakob Skou Pedersen)
The fraction of patients with accompanying RNA-seq data varies between cohorts, and thus the power to detect if mutations are associated with expression also varies. Overall, 1,190 patients (46%) have mRNA expression measurements. We report raw *P* values for mutation to expression association throughout the manuscript since some of the tests have significant sample overlap for meta-cohorts. The false discovery rate was controlled with the Benjamini–Hochberg procedure[21] and *Q* values together withRNA-seq sample counts for each of the tests presented in the manuscript can be found in **Supplementary Table 10**. Expression association *P* values, fold difference values, and RNA-seq sample counts for all candidates with expression associated to mutations are presented in **Supplementary Tables 4 and 5**.

Mutation-to-expression association was evaluated using non-parametric rank-sum based statistics on z-score normalized expression values. The use of z-scores equalizes the

expression means and variances for each cancer type and allows comparisons across cancer types. In particular, comparisons of expression between groups of mutated and non-mutated (wild-type) samples were evaluated using a two-sided Wilcoxon rank sum test both when samples came from a single cancer type or from multiple cancer types, such as the pan-cancer cohort or meta-cohorts. No assumptions on the distributions of expression z-scores were made given the use of the non-parametric statistics. Tied expression values were broken by adding a small random rank robust value.

Mutation-to-expression associations were evaluated both on the original, raw expression values as well as the two copy-number normalized expression sets mentioned above. For the *TERT* promoter, only samples powered to reliably detect mutation status were used. Fold difference values were calculated per mutation as the $\log_2$ ratio of the expression of the mutated tumor to the median of all wild-type tumors of the same cancer type. Reported fold difference (FD) values for an element with multiple mutations represent the median fold difference of all mutations in that element.

15. Copy number analyses (Esther Rheinbay)

We surveyed significant focal copy number alterations for candidate driver genes as orthogonal evidence for their “driverness”. Significant copy number alterations were obtained from the TCGA Copy Number Portal (http://portals.broadinstitute.org/tcga/home), analysis “2015-06-01-stddata-2015_04_02 regular peel-off”, a database of recurrent copy number alterations calculated by the GISTIC2 algorithm[55] across > 10,000 samples and 33 tumor types from TCGA. GISTIC2 results were included for candidate drivers if a gene was significant (residual *Q* < 0.1) and was located within a peak with ≤ 10 genes. Visualization was performed with the Integrative Genomics Viewer (IGV)[56].

16. Power calculations (Esther Rheinbay, Federico Abascal)

Estimation of total number of TERT promoter hotspot mutations. Detection sensitivity (d.s.) for all patients was calculated for the two most recurrent *TERT* promoter hotspot sites (chr5:1295228, chr5:1295250; hg19) using total read depth at these positions, sample purity, and average ploidy[57,58]. Detection sensitivity distributions (swarmplot/violinplot) were visualized

with the Python Seaborn package. Allele counts for these positions were generated by MuTect v1.1.4, using options --force_output and --force_alleles. For each cohort, the number and percentage of powered (≥ 90%) patients was obtained. The number of total expected mutations was then inferred as number of observed (called) mutations divided by the fraction of patients powered. The number of "missed" mutations is the difference between the total expected and observed mutations. Percentages of these numbers were calculated relative to the size of individual patient cohorts. Confidence intervals (95%) on the total percentage of patients with a *TERT* hotspot mutation were calculated using the beta distribution. Poisson confidence intervals were calculated for the number of missed mutations in the PCAWG cohort. Note that the inference of *TERT* mutations assumes exactly one mutation per patient. Estimates for the *FOXA1* promoter hotspot mutation (chr14:38064406; hg19) were conducted using the same procedure.

Calculation of the minimum powered mutation frequency in a population. Power to discover driver elements mutated at a certain frequency in the population were conducted as described before[26,59], but solving for the lowest frequency for a driver element in the patient population that is powered (≥ 90%) for discovery. The calculation of this lowest frequency takes into account (i) the average background mutation frequencies for each cohort/element combination, (ii) the median length and average detection sensitivity for each element type, patient cohort size, and (iii) a global desired false positive rate of 10%. The effect of element length is discussed in **Supplementary Note 10.**

17. Associations between mutation and signatures of selection: loss of heterozygosity and cancer allelic fractions (Federico Abascal, Iñigo Martincorena)

For protein-coding sequences, mutation recurrence can be analysed in the context of the functional impact of mutations (e.g., missense, truncating) to better distinguish the signal of

selection. In contrast, estimating the functional impact of mutations in non-coding elements of the genome is a difficult, yet unsolved problem. To overcome this limitation and be able to compare selection signatures for both coding and non-coding elements under a similar framework, we developed two measures of selection which are agnostic to the functional impact of mutations.

Association between mutation and loss of heterozygosity

When a tumor carries a driver mutation in one allele of a given gene, it may be the case that a second hit on the other allele confers a growth advantage and is positively selected. When one of the events involves the loss of one of the alleles, the process is referred to as loss of heterozygosity (LOH). This kind of biallelic losses are typical of, but not exclusive to, tumor suppressor genes (TSGs).

For each gene, we build a 2x2 contingency table indicating the number of cases in which the gene was mutated or not and the number of cases in which the gene was subject to LOH or not. We applied a Fisher's Exact test of proportions to identify which genes showed an excess of LOH associated to mutation. *P* values were corrected with the Benjamini-Hochberg FDR method to account for multiple hypotheses testing. This analysis was applied to each cohort separately and proved very successful in identifying TSG as well as some oncogenes (OGs).

Association between mutation and cancer allelic fractions

Driver mutations that provide an advantage for tumor cells are expected to show higher allelic fractions based on different interacting processes, including: early selection; amplification of the locus carrying the driver mutation; and loss of the non-mutated locus (LOH). Comparing cancer allelic fractions (CAF) can be informative to detect signatures of selection, both for TSGs and OGs.

CAFs are defined here as the proportion of reads coming from the tumor and carrying the mutation. To transform observed fractions (VAFs) into CAFs, tumor purity and local ploidy need to be taken into account according to the following formula:

$$CAF = VAF * (Lp * Pt + 2 * (1 - Pt)) / (Lp * Pt)$$

Where *Lp* corresponds to the local ploidy for the mutated locus, and *Pt* denotes the tumor purity. Ploidy and tumor purity predictions were obtained from Gerstung et al[57].

To determine whether CAFs for a given gene or element were higher than expected we compared them to the CAFs observed in flanking regions. To define flanking regions, we took 2 kb at each side of the gene/element, excluding any eventually overlapping coding exons, but included introns (if present). The two sets of CAFs associated to each gene/element, i.e., those CAFs lying within the gene/element and those flanking it, were compared with a t-test to detect significant deviations. *P* values were corrected with the FDR method. This approach was able to identify most known TSGs and OGs.

18. Estimation of the number of driver mutations in non-coding regions of known cancer genes (Federico Abascal, Iñigo Martincorena)

We conducted a series of analyses on regions combined across genes to determine whether the paucity of driver mutations found in non-coding regions was related to lack of statistical power in single-gene analyses. For protein-coding sequences, the number of driver mutations was estimated using dN/dS ratios as described in (Ref [22]). For non-coding, regulatory regions of protein-coding genes (promoter and UTRs), we relied on a modified version of the NBR negative binomial regression model described above to quantify the overall excess of driver mutations. We applied a second approach to determine whether there was an enrichment of LOH associated to mutations in the different types of non-coding regions associated to protein-coding genes.

Observed vs. expected numbers of mutations based on the NBR mutation model

NBR was used to estimate the background mutation rate expected across cancer genes, using a conservative list of 19,082 putative passenger genes as background. The resulting model is used to predict the numbers of passenger SNVs and indels expected by chance per element type per gene, and to aggregate sums across genes. For this analysis we used the curated list of 603 cancer genes from the Cancer Gene Census[45] (CGC v80; **Supplementary Table 7)**. To be as accurate as possible, we used a diverse set of covariates in the NBR model, including: local mutation rate (estimated on putative neutral regions +/- 100 kb around each gene), gene expression covariates (first 8 principal components of the matrix of average gene expression values in each tumor type, as well as two binary variables marking the 500 genes with highest

expression values in any tumor and 1,229 genes with a maximum FPKM lower than 0.1 across tumor types), and averaged copy-number calls for each gene across all samples (see **Supplementary Note 12** for more details). To reduce systematic biases, we removed samples with detection sensitivity (d.s.) problems associated to GC sequencing biases. Our d.s. estimates at the two main *TERT* hotspots revealed large variability in the d.s. between cases (**Extended Data Fig. 10d**). We thus required samples to have d.s. > 90% in both *TERT* hotspots, resulting in a set of 1,112 samples. We also removed hypermutators (>50,000 mutations/genome), and restricted the analysis to the Pancan-no-skin-melanoma cohort. The final set contained 936 samples and showed much better d.s. within promoter regions without significant differences in d.s. across element types (**Extended Data Fig. 10e**; **Supplementary Note 12**).

For each element type, the sum of observed mutations across the 603 cancer genes was compared to the sum of the expected rates to estimate the excess of mutations in regulatory and coding regions of cancer genes. An excess of observed mutations provides an estimate of the number of driver events[22]. Note that 5'UTRs and promoter definitions are partially overlapping, and so estimates of the numbers of driver mutations in these elements are not independent. Confidence intervals were calculated using the binomial approach for the ratio of two Poisson observations (*poisson.test* function in R), which are the number of mutations in the list of known cancer genes and in the list of passenger genes. It is important to know that these confidence intervals do not capture uncertainty in the assumptions of background model and should be interpreted with caution. For this reason, we systematically evaluated the impact of a diverse array of covariates on our estimates (**Supplementary Note 11**). We also note that this test can underestimate the number of non-coding drivers since some driver mutations can be present in the list of putative passenger genes, although this effect is expected to be quantitatively small if the density of driver mutations in regulatory regions of known cancer genes is higher than in those of putative passenger genes.

Mutation-LOH association for aggregates of genes
For this analysis, we combined data across known cancer genes, including 603 genes in the CGC and 154 additional significantly mutated genes found by exome studies[22,26]. To estimate whether there was an excess of LOH associated to mutation in regulatory and coding regions of cancer genes, we calculated the fold change in LOH for the aggregate of cancer genes and normalized it dividing by the fold change observed in passenger genes. Confidence intervals

were estimated using parametric bootstrapping (100,000 pseudoreplicates) for both cancer and passenger genes.

19. Mutational process and indel enrichment (Federico Abascal, Iñigo Martincorena)
For every protein-coding and long-noncoding gene in the genome, we record the proportion of indels of length 2–5 bp out of the total number of indels and compared this proportion with the background proportion using a binomial test. The background proportion was calculated using all protein-coding and lncRNAs genes. For every gene, we also calculated the indel rate and compared it to the background indel rate using a binomial test. Both sets of *P* values were independently corrected with the FDR method. The analysis was done for each tumor type separately. Genes with a *Q* value < 0.1 both for enrichment in 2–5 bp indels and for higher indel rates were further analyzed as candidates to be under the process of localized indel hypermutation described in this study. The levels of expression of these genes were analyzed across all tumor types. The hits *SPRN*, *CCDC152* and *RP11-1151B14.3* (all in Liver-HCC) were not highly expressed. Rather, the signal of indel enrichment appeared to come from their highly expressed neighbouring genes *CYP2E1*, *SEPP1*, and *MIR122*, respectively.

20. Structural variation analysis (Morten Muhlig Nielsen, Lars Feuerbach)
Structural variant data was provided by the PCAWG Structural Variation Working Group[60]. The data provide *P* values for the observed breakpoint counts in 50kb bins along the genome. Candidate elements were overlapped with the bins, and Fisher's method was used to calculate a single *P* value for each element. The set of element *P* values were corrected with the FDR method.

21. RNA structural analysis (Radhakrishnan Sabarinathan, Ciyue Shen, Chris Sander, Jakob Skou Pedersen)
In order to test if the observed mutations (SNVs) in the *RMRP* gene are biased towards high RNA secondary structure impact, we performed a permutation test by following the steps used in oncodriveFML[32] together with the predicted structural impact scores from RNAsnp[61]. At first, the RNAsnp was run with the options `-m 1 -w 300` and other default parameters to obtain the minimum correlation coefficient (r_min) score for each possible mutations in the *RMRP* gene. The r_min scores were then transformed, 1-((r_min+1)/2), to range between 0 and 1, where 1

indicates high structural impact score. Further, we followed the steps of oncodriveFML (see above) with 1,000,000 randomizations and using per sample mutational signatures (i.e., the probability of observing a mutation in a particular trinucleotide context in a given sample) to compute the *P* value at the cohort and sample level.

Furthermore, the RNA secondary structure impact scores (r_min) of indels were computed by using a modified version of RNAsnp (since the current version of RNAsnp is limited to substitutions only). Briefly, we first computed the base pair probability matrices of wild-type and mutant sequences (by taking into account the insertion or deletion) and then adjusted the size of matrices to be equal (by introducing additional rows and columns with zeros in one of the matrices with respect to insertion or deletion). Further, by following the steps of RNAsnp, we computed the r_min score. The structure shown in **Extended Data Fig. 5c** is based on the conserved secondary structure annotation obtained from Rfam (RF00030)[62].

Tertiary structure contacts in *RMRP* were predicted using evolutionary couplings co-variation analysis (EC analysis[63]) of the multiple sequence alignment of 933 eukaryotic *RMRP* sequences from Rfam (RF00030). The EC analysis (software available at https://github.com/debbiemarkslab/plmc) was run with the options `-le 20.0, -lh 0.01, -t 0.2, -m 100` and the top 100 interactions were chosen as predicted contacts, either in secondary or tertiary structure, depending on local context. As no experimental 3D structure or cross-linking experiments of the mammalian *RMRP* are available, interaction sites were inferred by homology to the partially known yeast *RMRP* crystal structure. We (1) aligned the human *RMRP* sequence with the *Saccharomyces cerevisiae RMRP* sequence using the sequence family covariance model from Rfam and (2) mapped the locations of RNA-protein interactions within 4Å[64] from the crystal structure and the experimentally determined RNA-protein crosslinking sites[65], and RNA substrate crosslinking sites[66] from the yeast sequence to the human *RMRP* sequence. For the crosslinking sites, a ±3 nucleotide window is reported as the interaction site. In order to test if the locations of the observed indels are biased towards tertiary structure, protein- or substrate-interaction sites, 1,000,000 randomizations of five indels were performed assuming uniform distribution of indels across the *RMRP* gene body, and an empirical *P* value was calculated.

Two different overlapping deletion calls in the *RMRP* gene body were observed in the same thyroid cancer patient. After manual inspection of the tumor and normal bam files, it was found

that these calls were based on the same mutational event, and only one was included in the above analysis.

22. Cancer associated germline variant distance to non-coding driver candidates (Morten Muhlig Nielsen)

We used a set of genome-wide significant cancer associated germline SNPs (n = 650) from the NHGRI-EBI GWAS catalog[67] as collected by Sud et al.[68]. We evaluated the genomic distance from candidate non-coding drivers to the closest germline variant. All distances were above 50 kb with the exception of the *TERT* promoter, which was 1 kb away from a coding variant (rs2736098) in the *TERT* gene.

23. Assessing the significance of somatic rearrangement breakpoints (Jeremiah Wala, Marcin Imielinski, Ofer Shapira, Kiran Kumar, Rameen Beroukhim)

Modeling breakpoint counts with a Gamma-Poisson regression model

2,693 samples from PCAWG were included in the structural variation analysis, of which 2,605 had at least one structural variant. This includes 110 cases (mostly acute leukemias) that were deemed suboptimal for point mutation calling, but were suitable for breakpoint analysis.

To model the background rate of somatic breakpoints, we first established a discrete coordinate system on which to evaluate genomic covariates and breakpoint counts. We binned the genome into 50 kb bins, with 1 kb of overlap between bins to reduce edge effects, which produced 61,920 loci. Complex events with many tightly clustered breakpoints could dominate the breakpoint count at a single bin and cause an overestimation of the prevalence of breakpoints at those loci. To account for this, we only considered one breakpoint per sample per locus. After removing locus-sample duplicates, 336,496 breakpoints (55% of all breakpoints) were counted within our model. The number of breakpoints per bin ranged between 0 and 119, with a median of 5.0 and mean of 6.1. A large majority of bins (90.0%) of bins contained 20 or fewer breakpoints, and 2.6% contained zero breakpoints. The model was robust to varying the bin size. When we increased the bin size an order of magnitude to 500 kb, we found 25 significant loci, 21 of which overlapped with the significant loci from the 50 kb model (**Supplementary Table 17**).

The detected rate of breakpoints across the genome is also confounded by the mapping quality within a locus. Rearrangements in regions that are difficult to align to (e.g. alpha-satellite repeats) were rejected by our variant callers, leading to a relative depletion of events in regions with low mappability. To control for this effect, we use the concept of “eligible territory” from Imielinski et al[69], and normalized the breakpoint counts within each locus by the number of bases eligible for breakpoint detection. To establish an eligible territory, we used the “universal mask” described in Li 2014[70] and used in Imielinski et al[69] (https://data.broadinstitute.org/svaba/um75-hs37d5.covered.bed). Briefly, this mask filters regions of low mappability, low complexity, and sites of unusually high numbers of aberrant SNV calls from the 1,000 Genomes Project.

The distribution of breakpoint frequencies per bin was widely over-dispersed for a Poisson regression model (**Supplementary Fig. 8a**). We used Cameron and Trivedi’s Overdispersion Test (AER::dispersiontest in R-3.4.3) to determine the dispersion parameter alpha (equivalently parametrized as 1/theta), which is zero in a true Poisson regression model. The resulting alpha of 0.31 ($P < 2.2 \times 10^{-16}$) suggests a Gamma-Poisson (GP) fit to the data (or equivalently, a negative binomial). We therefore elected to model the breakpoint frequencies using a GP regression model, where the log of the expected value of the breakpoint counts per bin could be modeled as a linear combination of genomic covariates within each bin and a hyperparameter allowing for extra variance of the breakpoint counts, adapted from the model for SNVs and indels from Imielinski et al[69], and specified as:

$$B_i \sim GP\left(w_i e^{\beta_j x_{ji}}, \theta\right)$$

where w_i is the eligible territory of locus i, B_i is the breakpoint count at locus i, x_{ji} is the matrix describing the values of covariate j at locus i, and (theta) is a single scalar representing the shape parameter of the distribution. The regression coefficients (beta) were then found by maximum likelihood estimation using MASS::glm.nb in R-3.4.3 which utilizes the NB2 parameterization of the GP function. The source code for the GP model is available at https://github.com/mskilab/fish.hook. See **Supplementary Note 6** for further benchmarking of the GP model.

Genomic covariates that predict breakpoint frequencies

We hypothesized that local sequence features (e.g., density of repetitive elements), replication-timing, chromatin state, epigenetic modifications, and other genomic features, could be predictive of breakpoints rates within our GP model. We therefore fit our GP model using both “interval”

covariates that indicate genomic regions (e.g., SINE elements), and “numeric” tracks that indicate values (e.g., GC content) associated with genomic regions. The complete list of genomic covariates and their coefficients are listed in **Supplementary Table 13**.

Assessing the significance of loci with high breakpoint rates

We used the full GP model to estimate the background rates for each locus and to calculate the probability that c_i or more events would be observed at locus *i*. The count data c_i is restricted to a non-negative integer, and the probabilities will be a slight overestimate of the true value. To correct for this, we use the procedure employed in Imielinski et al[69] to select a random probability from a uniform distribution between the probability of observing c_i breakpoints and the probability of observing $c_i + 1$ breakpoints. To correct for multiple hypothesis testing, we calculated the false discovery rate (FDR) using the Benjamini–Hochberg method[21]. The significant loci were defined as those with an FDR of < 10%. We created a final significant loci list by joining significant loci and their intervening regions if they were separated by fewer than 1 Mbp. Analysis of the *P* values and quantile–quantile plot (**Supp. Fig. 8c**) shows a uniform distribution without apparent biases of *P* values.

We next attempted to determine which breakpoints at each significantly recurrent locus were themselves likely driver rearrangements. We noted that the breakpoint counts at many loci were dominated by rearrangements from a small subset of tumor types, suggesting that the rearrangements in these tumor types were drivers. Some rearrangements from other tumor types, however, would often also be seen at background rates expected for these tumor types. We therefore calculated an enrichment *P* value (binomial test) that tumor type *T* was enriched at that locus:

$$p_T = 1 - \sum_{i=0}^{k} \binom{n}{i} r_T^i (1 - r_T)^{n-i}$$

Commented [MOU1]: Note that I changed the “n-1” at the exponent at the end to “n-i” which I believe to be the correct equation.

where *k* is the number of breakpoints from tumor type *T* intersecting the locus, *n* is the total number of breakpoints intersecting the locus, and r_T is the fraction of breakpoints from tumor type *T* within the entire PCAWG cohort. Using this enrichment score, we considered as driver rearrangements only rearrangements from the most enriched tumor-type and any tumor-type *x* with $\log(p_x/p_{top}) < 3$.

Comparison of recurrent breakpoint loci with significantly recurrent SCNAs and known fusions

We compared the significantly recurrent breakpoint loci with sites of significantly recurrent SCNAs obtained from GISTIC2[55] analyses and the COSMIC[71] cancer database curated list of gene fusions in cancer (http://cancer.sanger.ac.uk/cosmic/fusion). Recurrent breakpoint loci that overlapped a GISTIC peak region (deletion or amplification) from either the pan-cancer (all_cancers) analysis or any tumor-type specific analysis were considered as representing a recurrent SCNA. Recurrent fusions were considered supported by the literature if the two loci involved in the recurrent fusion overlapped both genes from an entry in the COSMIC fusion database.

24. Classification of rearrangement patterns at sites of recurrent breakpoints (Jeremiah Wala, Joachim Weischenfeldt, Rameen Beroukhim)

To predict the functional effects of the recurrent breakpoint loci, we scored each locus based on its pattern of rearrangements and genomic covariates. For each rearrangement containing a significantly recurrent breakpoint, we calculated the rearrangement dispersion (RD) score, which we defined as the median absolute deviation (MAD) of the breakpoint-breakpoint distance (or 10^9 for inter-chromosomal rearrangements) divided by the median breakpoint-breakpoint distance, considering rearrangements within enriched tumor-types at that locus (see above). For inter-chromosomal rearrangements, we evaluated only rearrangements to the most frequent chromosome. Rearrangements at sites of known recurrent oncogenic fusions exhibited low RD-scores (e.g. *IGH-BCL2*, RD-score: 0.01), while breakpoints at known fragile and driver SCNA sites exhibited a high RD-score. Hartigans' dip-test (in R v3.3 - diptest::dip.test) supported a non-unimodal distribution ($P = 0.02$) with a discriminant of 0.07. The RD score for all significant loci is listed in **Supplementary Table 14**. Recurrent breakpoints with RD < 0.07 were classified as supporting fusion-type driver events. For each recurrent breakpoint locus not classified as fragile-type or fusion-type, we classified the locus as amplified, deleted, or neutral by whether it overlapped with a known GISTIC peak, or had a significantly higher mean tumor–normal read-depth ratio within the region compared with the surrounding region.

25. **Assessing the significance of somatic juxtapositions** (Ofer Shapira, Jeremiah Wala, David Craft, Marcin Imielinski, Kiran Kumar, Joachim Weischenfeldt, Rameen Beroukhim)

We used a linear combination of two models to predict the density of juxtapositions. The first hypothesizes that the background probability is $p_{ij}^{bi} = q_i s_{ij} + q_j s_{ji}$, where q_i is the marginal probability of a rearrangement initiated in locus *i*, and s_{ij} is the conditional probability that a break at *i* will connect to site *j.* Since we cannot distinguish between the start and end sites, we also add the reciprocal term, to yield a probability proportional to the local rate of retreatments connecting sites *i* and *j.* The marginal of the start site, q_i, is determined from the empirical breakpoint density, R_i, by applying preconditioned conjugate gradient descent optimization to the following problem:

$$q_i + \sum_j q_j s_{ji} = r_i \quad \forall i$$

$$\sum_i q_i = 1$$

$$q_i \geq 0 \quad \forall i$$

The conditional probability matrix is determined from the empirical distribution of rearrangement spans distances between paired breakpoints. The second model hypothesizes that the background probability is $p_{ij}^{db} = r_i r_j l_{ij}$, where r_i and r_j are the breakpoint densities and l_{ij} is a span factor connecting sites *i* and *j* found by solving the following constrained nonlinear optimization problem:

$$argmin_{l'} \left\{ \sum_i \left\| f(r_i r_j l_{ij}) - l \right\|_2 \right\}$$

$$\sum_j r_j l_{ij} = 1 \quad \forall i$$

The function *f* transforms the probability matrix to a span distribution function corresponding to the empirical distribution, *l*. Explicitly, the elements of l_{ij} take values corresponding to the distance between loci *i* and *j* and are given by *l'*, a vector that maps the distance |*i-j*| to a value in *R*. In this analysis, both *l* and *l'* are discrete numerical vectors with 10 elements each. The function *f* than calculates the global span distribution by drawing rearrangements from the distribution $p_{ij} = r_i r_j l_{ij}$ and binning their span. This value is generally different than *l'*. To construct the probability matrices of the break-invasion, p_{ij}^{bi}, and double-break join, p_{ij}^{db},models, we divided the genome into bins containing a target of 100 rearrangements per bin. To avoid cases in which a

cluster of rearrangements is divided into two bins, we imposed a minimal distance between breakpoints of 2 kb; if a bin boundary falls between two breakpoints not meeting this condition, the bin is extended until the condition is met. The normalized distribution of number of breakpoint is the parameter r_i used to construct the two models. After binning the genome, we constructed the rearrangement matrix, k_{ij}, by assigning each rearrangement in our dataset to a tile. Each sample was only allowed to contribute up to one rearrangement per tile.

The overall background rate of events is therefore represented by $p_{ij} = \alpha_k p_{ij}^{bi} + (1 - \alpha_k) p_{ij}^{db}$, where the linear combination is taken over a set of weighting parameters α^k. We chose to use the distance between breakpoints (span) as a natural choice for the weighting parameters in this two-dimensional genomic representation. We divided the 2D space into short (≤1 Mbp), long (>1 Mbp), and inter-chromosomal translocations, and obtained the values of α^k by minimizing the Bayesian Inference Criteria (BIC). A list of recurrent rearrangements for the long subset was then generated by calculating a *P* value in each tile with a binomial test statistic against k_{ij}, followed by control of multiple hypotheses using the Benjamini–Hochberg FDR procedure at a threshold of 0.1 and a minimum count of two rearrangements per tile.

26. Annotation of potential functional effects of rearrangements (Jeremiah Wala, Ofer Shapira, Nikos Sidiropoulos, Joachim Weischenfeldt, Rameen Beroukhim)

We annotated the potential functional effects of each rearrangement based on the locations and orientations of its breakpoints. Gene definitions for genome build hg19 were obtained from the UCSC Table Browser[72]. Rearrangements were evaluated for whether they could produce a possible in-frame sense fusion transcript. The CCDS database[73] from hg19 was obtained from the UCSC Table Browser. With the CCDS intervals, breakpoints contained within a gene were annotated by which intron or exon they overlapped with, and the coding frame (1,2, or 3) of the first exon opposite the direction of the breakpoints. Candidate fusions were called as in-frame and sense if 1) the relative orientations of the breakpoints and directionality of the gene resulted in a potential sense fusion and 2) the two breakpoints were in the same coding frame.

We used a classification scheme described in our companion paper[60] to segregate rearrangements according to likely shared mechanisms of formation. We considered five major

classes of rearrangements: isolated deletions, inversions, tandem duplications, interchromosomal translocations, and complex rearrangements.

Breakpoint association with gene expression in cis

To identify SRBs associated with nearby gene expression change, we applied CESAM which integrates rearrangement-derived breakpoints with RNA-seq data (FPKM-UQ) to identify expression changes associated with breakpoints in *cis*, as previously described[74]. In brief, normalized RNA-seq expression is regressed on a rearrangement breakpoint matrix, using tissue-type, total number of rearrangements, and first principal components of the breakpoint matrix as covariates. Expression data was dosage-adjusted prior to the analysis by normalizing the expression level of each gene (FPKM-UQ) to the copy number level of the gene in each tumor sample. This was done to remove effects due to copy-number dosage effects, i.e., not attributable to *cis*-effects. Only breakpoint bins with at least three tumors having associated RNA-seq data were evaluated. To assess whether SRB-CESAM hits were associated with juxtaposition of normally distant enhancer elements, the distal breakpoint of a rearrangement (relative to the breakpoint closest to the SRB centroid) was intersected with tissue-matched enhancer regions[15] with a window of +/- 20 kb. Significance was assessed by random shuffling of breakpoint positions on the mappable genome (alpha < 0.1).

The pan-cancer association between copy number-adjusted gene expression changes of CESAM hits with breakpoints was assessed by computing the average of copy number-adjusted gene expression fold-change for each histology type to alleviate cell-type specific biases in gene expression.

Rearrangement types and effect on expression, enhancer-distance, and TSGs

For each cluster of rearrangements, the genomic centroid position of the breakpoints was used to identify the most deregulated gene within a window of +/- 1 Mb of the centroid. Fold-change expression was calculated as the ratio between the median of gene expression for tumor samples with (SV+) versus without (SV-) a breakpoint at the cluster. A randomized background set was calculated for each cluster by random sampling (n = 1,000) a breakpoint from the complete set of rearrangement and computing fold-change as above with the same set of SV+ and SV- samples. This was done to remove sample-specific biases in gene expression levels.

The distance to the nearest tissue-specific enhancer was computed as described above. Briefly, when available, we matched the tissue of origin from the tumor to the cell type from the enhancer track. Where no match was available, we compared the breakpoints with the complete enhancer set across cell types.

Biallelic inactivation events

To identify tumor suppressor two-hit events, we defined biallelic inactivation as a gene locus $G_{A/B}$, where alleles A and B are genetically altered, leading to a genetic $G_{mut/mut}$ state. The biallelic inactivation assessment includes three genetic inactivation event types consisting of somatic or germline deletions (“Loss”), somatic or germline SVs (“Break”), and somatic or germline SNVs (“Mutation”). Given a heterozygous $G_{A/B}$ locus, we required a loss of the A allele of the gene, leading to a hemizygous $G_{-/B}$ state, and genetic inactivation of the remaining B allele, specifically requiring the second event to overlap the loss on the A allele, leading to biallelic inactivation. We considered four classes of biallelic inactivations: i) Loss/Mutation, nonsynonymous driver mutations of the B allele; ii) Loss/Loss, two deletion events that overlap an exon and the copy-number derived allele count is 0 both for A and B allele; iii) Loss/Break, SVs where one or both breakpoints are situated in an exon of the B allele; and iv) Mutation/Mutation, a nonsynonymous germline SNV and a nonsynonymous driver somatic SNV of the same gene. We infer the germline mutation to occur on the A allele and the somatic mutation on the B allele, with the assumption that two independent driver mutation events are highly unlikely to occur on the same allele (https://bitbucket.org/weischenfeldt/biallelic_inactivation). Only curated tumor-suppressor genes were assessed. Enrichment of biallelic inactivation for each rearrangement cluster type was assessed by comparing the frequencies to a permuted set (Fisher’s Exact test, n = 1,000), showing enrichment of biallelic inactivation at deletion-type ($P < 0.005$), neutral-type ($P < 0.001$) and fragile-type ($P < 0.001$), and depletion of amplification-type ($P < 0.001$) and fusion-type ($P < 0.001$) rearrangement clusters.

SV portal for visualisation of SV recurrence

SVscape is an interactive R Shiny server build for browsing structural variant and breakpoint distribution along user defined genes or genomic loci. 1D breakpoint enrichment *P* values are estimated by Fisher’s Exact test, and asterisks denote significant 2D rearrangements involving the given locus. SVscape is available as a public instance at www.svscape.org and can be downloaded and deployed locally at www.bitbucket.org/weischenfeldt/svscape.git.

27. Power calculations for rearrangements (Ofer Shapira, Kiran Kumar)

To analyze the number of tumor–normal pairs needed to reach saturation in the detection of fusions, we employed a binomial power model[26]. We defined a null distribution, H_{NULL} ~ Binomial (N, p_{NULL}) where $p_{NULL} \sim 1 - (1 - p_{90})^m$, is the probability of a patient having at least one rearrangement, p_{90} is the 90th percentile value of p_{ij} from our background model probabilities, and m is the median number of rearrangements per sample. The two-dimensional genomic fusions map was divided into 100 x 100 Kbp tiles in this power analysis.

We performed the analysis first as a function of the distance between breakpoints with median number of rearrangements per sample of the entire cohort (**Extended Data Fig. 10a**). The second analysis was performed as a function of the median number of rearrangements per sample, spanning values represented by the ICGC histologies with more than 15 samples (**Fig. 4b**). For each total number of tumour–normal pairs, N, the general procedure involved: 1) finding the minimal number of patients needed to reach significance level ---of $P < 0.1/(\# \text{ of tiles})$ based on H_{null}; 2) using this value, calculating the minimal rate above background, r, that yields 90% power of the alternative distribution, H_{alt} ~ Binomial(N, p_{null} + r); and 3) calculating contour lines of constant value rates above background.

References

1. Ramos, A. H. *et al.* Oncotator: cancer variant annotation tool. *Hum. Mutat.* **36**, E2423–9 (2015).
2. Tamborero, D. *et al.* Cancer Genome Interpreter annotates the biological and clinical relevance of tumor alterations. *Genome Med.* **10**, 25 (2018).
3. Kasar, S. *et al.* Whole-genome sequencing reveals activation-induced cytidine deaminase signatures during indolent chronic lymphocytic leukaemia evolution. *Nat. Commun.* **6**, 8866 (2015).
4. Kim, J. *et al.* Somatic ERCC2 mutations are associated with a distinct genomic signature in urothelial tumors. *Nat. Genet.* **48**, 600–606 (2016).
5. Polak, P. *et al.* A mutational signature reveals alterations underlying deficient homologous recombination repair in breast cancer. *Nat. Genet.* **49**, 1476–1486 (2017).
6. Alexandrov, L. B. *et al.* The Repertoire of Mutational Signatures in Human Cancer. *Nature* (2019).
7. Harrow, J. *et al.* GENCODE: the reference human genome annotation for The ENCODE Project. *Genome Res.* **22**, 1760–1774 (2012).
8. Iyer, M. K. *et al.* The landscape of long noncoding RNAs in the human transcriptome. *Nat. Genet.* **47**, 199–208 (2015).
9. Burge, S. W. *et al.* Rfam 11.0: 10 years of RNA families. *Nucleic Acids Res.* **41**, D226–32 (2013).
10. Lowe, T. M. & Eddy, S. R. tRNAscan-SE: a program for improved detection of transfer RNA genes in genomic sequence. *Nucleic Acids Res.* **25**, 955–964 (1997).
11. Lestrade, L. & Weber, M. J. snoRNA-LBME-db, a comprehensive database of human H/ACA and C/D box snoRNAs. *Nucleic Acids Res.* **34**, D158–62 (2006).
12. Kozomara, A. & Griffiths-Jones, S. miRBase: annotating high confidence miRNAs using

deep sequencing data. *Nucleic Acids Res.* **42**, D68–D73 (2014).

13. Londin, E. *et al.* Analysis of 13 cell types reveals evidence for the expression of numerous novel primate- and tissue-specific microRNAs. *Proc. Natl. Acad. Sci. U. S. A.* **112**, E1106–15 (2015).
14. Fingerman, I. M. *et al.* NCBI Epigenomics: a new public resource for exploring epigenomic data sets. *Nucleic Acids Res.* **39**, D908–12 (2011).
15. Roadmap Epigenomics Consortium *et al.* Integrative analysis of 111 reference human epigenomes. *Nature* **518**, 317–330 (2015).
16. Zhu et al., Candidate Cancer Driver Mutations in Distal Regulatory Elements and Long-Range Chromatin Interaction Networks, Molecular Cell (2020), https://doi.org/10.1016/j.molcel.2019.12.027. in press.
17. Fu, Y. *et al.* FunSeq2: a framework for prioritizing noncoding regulatory variants in cancer. *Genome Biol.* **15**, 480 (2014).
18. Liu, E. M. *et al.* Identification of Cancer Drivers at CTCF Insulators in 1,962 Whole Genomes. *Cell Syst* **8**, 446–455.e8 (2019).
19. Alexandrov, L. B. *et al.* Signatures of mutational processes in human cancer. *Nature* **500**, 415–421 (2013).
20. Polak, P. *et al.* Cell-of-origin chromatin organization shapes the mutational landscape of cancer. *Nature* **518**, 360–364 (2015).
21. Benjamini, Y. & Hochberg, Y. Controlling the false discovery rate: a practical and powerful approach to multiple testing. *J. R. Stat. Soc. Series B Stat. Methodol.* (1995).
22. Martincorena, I. *et al.* Universal Patterns of Selection in Cancer and Somatic Tissues. *Cell* (2017). doi:10.1016/j.cell.2017.09.042
23. Shuai, S., Gallinger, S., Stein, L. & on behalf of the PCAWG Drivers and Functional Interpretation Group and the ICGC/TCGA Pan-Cancer Analysis of Whole Genomes Network. DriverPower: Combined burden and functional impact tests for cancer driver

discovery. doi:10.1101/215244

24. Lanzos, A. *et al.* Discovery of Cancer Driver Long Noncoding RNAs across 1112 Tumour Genomes: New Candidates and Distinguishing Features. (2016). doi:10.1101/065805
25. Lochovsky, L., Zhang, J., Fu, Y., Khurana, E. & Gerstein, M. LARVA: an integrative framework for large-scale analysis of recurrent variants in noncoding annotations. *Nucleic Acids Res.* **43**, 8123–8134 (2015).
26. Lawrence, M. S. *et al.* Discovery and saturation analysis of cancer genes across 21 tumour types. *Nature* **505**, 495–501 (2014).
27. Nik-Zainal, S. *et al.* Landscape of somatic mutations in 560 breast cancer whole-genome sequences. *Nature* **534**, 47–54 (2016).
28. Juul, M. *et al.* Non-coding cancer driver candidates identified with a sample- and position-specific model of the somatic mutation rate. *Elife* **6**, (2017).
29. Bertl, J. *et al.* A site specific model and analysis of the neutral somatic mutation rate in whole-genome cancer data. *BMC Bioinformatics* **19**, 147 (2018).
30. Hornshøj, H. *et al.* Pan-cancer screen for mutations in non-coding elements with conservation and cancer specificity reveals correlations with expression and survival. *NPJ Genom Med* **3**, 1 (2018).
31. Anscombe, F. J. & Fisher, R. A. Statistical Methods for Research Workers. *J. R. Stat. Soc. Ser. A* **118**, 486 (1955).
32. Mularoni, L., Sabarinathan, R., Deu-Pons, J., Gonzalez-Perez, A. & López-Bigas, N. OncodriveFML: a general framework to identify coding and non-coding regions with cancer driver mutations. *Genome Biol.* **17**, 128 (2016).
33. Kircher, M. *et al.* A general framework for estimating the relative pathogenicity of human genetic variants. *Nat. Genet.* **46**, 310–315 (2014).
34. Douville, C. *et al.* Assessing the Pathogenicity of Insertion and Deletion Variants with the Variant Effect Scoring Tool (VEST-Indel). *Hum. Mutat.* **37**, 28–35 (2016).

35. Umer, H. M. *et al.* A Significant Regulatory Mutation Burden at a High-Affinity Position of the CTCF Motif in Gastrointestinal Cancers. *Hum. Mutat.* **37**, 904–913 (2016).
36. Cibulskis, K. *et al.* Sensitive detection of somatic point mutations in impure and heterogeneous cancer samples. *Nat. Biotechnol.* **31**, 213–219 (2013).
37. Brown, M. B. 400: A Method for Combining Non-Independent, One-Sided Tests of Significance. *Biometrics* **31**, 987 (1975).
38. Poole, W., Gibbs, D. L., Shmulevich, I., Bernard, B. & Knijnenburg, T. A. Combining dependentP-values with an empirical adaptation of Brown's method. *Bioinformatics* **32**, i430–i436 (2016).
39. Derrien, T. *et al.* Fast computation and applications of genome mappability. *PLoS One* **7**, e30377 (2012).
40. Perera, D. *et al.* Differential DNA repair underlies mutation hotspots at active promoters in cancer genomes. *Nature* **532**, 259–263 (2016).
41. Sabarinathan, R., Mularoni, L., Deu-Pons, J., Gonzalez-Perez, A. & López-Bigas, N. Nucleotide excision repair is impaired by binding of transcription factors to DNA. *Nature* **532**, 264–267 (2016).
42. Fredriksson, N. J. *et al.* Recurrent promoter mutations in melanoma are defined by an extended context-specific mutational signature. *PLoS Genet.* **13**, e1006773 (2017).
43. Pearson, C. E., Zorbas, H., Price, G. B. & Zannis-Hadjopoulos, M. Inverted repeats, stem-loops, and cruciforms: significance for initiation of DNA replication. *J. Cell. Biochem.* **63**, 1–22 (1996).
44. Ye, C., Ji, G., Li, L. & Liang, C. detectIR: a novel program for detecting perfect and imperfect inverted repeats using complex numbers and vector calculation. *PLoS One* **9**, e113349 (2014).
45. Futreal, P. A. *et al.* A census of human cancer genes. *Nat. Rev. Cancer* **4**, 177–183 (2004).
46. Dimitrieva, S. & Bucher, P. UCNEbase—a database of ultraconserved non-coding

elements and genomic regulatory blocks. *Nucleic Acids Res.* **41**, D101–D109 (2012).

47. Benson, G. Tandem repeats finder: a program to analyze DNA sequences. *Nucleic Acids Res.* **27**, 573–580 (1999).
48. PCAWG Transcriptome Core Group *et al.* Genomic basis for RNA alterations revealed by whole-genome analyses of 27 cancer types. *Nature* (2019).
49. Dobin, A. *et al.* STAR: ultrafast universal RNA-seq aligner. *Bioinformatics* **29**, 15–21 (2013).
50. Kim, D. *et al.* TopHat2: accurate alignment of transcriptomes in the presence of insertions, deletions and gene fusions. *Genome Biol.* **14**, R36 (2013).
51. Hose, J. *et al.* Dosage compensation can buffer copy-number variation in wild yeast. *Elife* **4**, (2015).
52. Lockstone, H. E. *et al.* Gene expression profiling in the adult Down syndrome brain. *Genomics* **90**, 647–660 (2007).
53. Birchler, J. A. Reflections on studies of gene expression in aneuploids. *Biochem. J* **426**, 119–123 (2010).
54. Heard, E. & Disteche, C. M. Dosage compensation in mammals: fine-tuning the expression of the X chromosome. *Genes Dev.* **20**, 1848–1867 (2006).
55. Mermel, C. H. *et al.* GISTIC2.0 facilitates sensitive and confident localization of the targets of focal somatic copy-number alteration in human cancers. *Genome Biol.* **12**, R41 (2011).
56. Thorvaldsdóttir, H., Robinson, J. T. & Mesirov, J. P. Integrative Genomics Viewer (IGV): high-performance genomics data visualization and exploration. *Brief. Bioinform.* **14**, 178–192 (2013).
57. Gerstung, M. *et al.* The evolutionary history of 2,658 cancers. *Nature* (2019).
58. Carter, S. L. *et al.* Absolute quantification of somatic DNA alterations in human cancer. *Nat. Biotechnol.* **30**, 413–421 (2012).
59. Rheinbay, E. *et al.* Recurrent and functional regulatory mutations in breast cancer. *Nature* (2017). doi:10.1038/nature22992

60. Li, Y. *et al.* Patterns of somatic structural variation in human cancer genomes. *Nature* (2019).

61. Sabarinathan, R. *et al.* RNAsnp: efficient detection of local RNA secondary structure changes induced by SNPs. *Hum. Mutat.* **34**, 546–556 (2013).

62. Nawrocki, E. P. *et al.* Rfam 12.0: updates to the RNA families database. *Nucleic Acids Res.* **43**, D130–7 (2015).

63. Weinreb, C. *et al.* 3D RNA and Functional Interactions from Evolutionary Couplings. *Cell* **165**, 963–975 (2016).

64. Perederina, A., Esakova, O., Quan, C., Khanova, E. & Krasilnikov, A. S. Eukaryotic ribonucleases P/MRP: the crystal structure of the P3 domain. *EMBO J.* **29**, 761–769 (2010).

65. Khanova, E., Esakova, O., Perederina, A., Berezin, I. & Krasilnikov, A. S. Structural organizations of yeast RNase P and RNase MRP holoenzymes as revealed by UV-crosslinking studies of RNA–protein interactions. *RNA* **18**, 720–728 (2012).

66. Esakova, O., Perederina, A., Berezin, I. & Krasilnikov, A. S. Conserved regions of ribonucleoprotein ribonuclease MRP are involved in interactions with its substrate. *Nucleic Acids Res.* **41**, 7084–7091 (2013).

67. Welter, D. *et al.* The NHGRI GWAS Catalog, a curated resource of SNP-trait associations. *Nucleic Acids Res.* **42**, D1001–6 (2014).

68. Sud, A., Kinnersley, B. & Houlston, R. S. Genome-wide association studies of cancer: current insights and future perspectives. *Nat. Rev. Cancer* **17**, 692–704 (2017).

69. Imielinski, M., Guo, G. & Meyerson, M. Insertions and Deletions Target Lineage-Defining Genes in Human Cancers. *Cell* **168**, 460–472.e14 (2017).

70. Li, H. Toward better understanding of artifacts in variant calling from high-coverage samples. *Bioinformatics* **30**, 2843–2851 (2014).

71. Forbes, S. A. *et al.* COSMIC: somatic cancer genetics at high-resolution. *Nucleic Acids*

Res. **45**, D777–D783 (2017).

72. Karolchik, D. *et al.* The UCSC Table Browser data retrieval tool. *Nucleic Acids Res.* **32**, D493–6 (2004).

73. Pruitt, K. D. *et al.* The consensus coding sequence (CCDS) project: Identifying a common protein-coding gene set for the human and mouse genomes. *Genome Res.* **19**, 1316–1323 (2009).

74. Weischenfeldt, J. *et al.* Pan-cancer analysis of somatic copy-number alterations implicates IRS4 and IGF2 in enhancer hijacking. *Nat. Genet.* **49**, 65–74 (2017).
