# Supplementary material for: Analyses of non-coding somatic drivers in 2,658 cancer whole genomes
Source: Nature. 2020 Feb 5;578(7793):102–11. doi: 10.1038/s41586-020-1965-x (PMC7054214; doi:10.1038/s41586-020-1965-x)
Supplement: Supplementary file 2 — Reporting Summary [file 41586_2020_1965_MOESM2_ESM.pdf]

nature research

Corresponding author(s): Gad Getz
Last updated by author(s): Oct 18, 2019

Reporting Summary

Nature Research wishes to improve the reproducibility of the work that we publish. This form provides structure for consistency and transparency in reporting. For further information on Nature Research policies, see Authors & Referees and the Editorial Policy Checklist.

Statistics

For all statistical analyses, confirm that the following items are present in the figure legend, table legend, main text, or Methods section.

n/a	Confirmed	
☐	☒	The exact sample size (*n*) for each experimental group/condition, given as a discrete number and unit of measurement
☐	☒	A statement on whether measurements were taken from distinct samples or whether the same sample was measured repeatedly
☐	☒	The statistical test(s) used AND whether they are one- or two-sided<br>*Only common tests should be described solely by name; describe more complex techniques in the Methods section.*
☐	☒	A description of all covariates tested
☐	☒	A description of any assumptions or corrections, such as tests of normality and adjustment for multiple comparisons
☐	☒	A full description of the statistical parameters including central tendency (e.g. means) or other basic estimates (e.g. regression coefficient) AND variation (e.g. standard deviation) or associated estimates of uncertainty (e.g. confidence intervals)
☐	☒	For null hypothesis testing, the test statistic (e.g. *F*, *t*, *r*) with confidence intervals, effect sizes, degrees of freedom and *P* value noted<br>*Give P values as exact values whenever suitable.*
☐	☒	For Bayesian analysis, information on the choice of priors and Markov chain Monte Carlo settings
☐	☒	For hierarchical and complex designs, identification of the appropriate level for tests and full reporting of outcomes
☐	☒	Estimates of effect sizes (e.g. Cohen's *d*, Pearson's *r*), indicating how they were calculated

Our web collection on statistics for biologists contains articles on many of the points above.

Software and code

Policy information about availability of computer code

Data collection

Data and metadata were collected from International Cancer Genome Consortium (ICGC) consortium members using custom software packages designed by the ICGC Data Coordinating Centre. The general-purpose core libraries and utilities underlying this software have been released under the GPLv3 open source license as the "Overture" package and are available at https://www.overture.bio. Other data collection software used in this effort, such as ICGC-specific portal user interfaces, are available upon request to contact@overture.bio.

Data analysis

The workflows executing core WGS alignment, QC and variant-calling software are packaged as executable Dockstore images and available at: https://dockstore.org/search?labels.value.keyword=pcawg&searchMode=files. Individual software components are as follows: BWA-MEM v0.78.8-r455; DELLY v0.6.6; ACEseq v1.0.189; DKFZ somatic SNV workflow v1.0.132-1; Platypus v0.7.4; ascatNgs v1.5.2; BRASS v4.012; grass v1.1.6; CaVEMan v1.50; Pindel v1.5.7; ABSOLUTE/JaBbA v1.5; SvABA 2015-05-20; dRanger 2016-03-13; BreakPointer 2015-12-22; MuTect v1.1.4; MuSE v1.0rc; SMuFIN 2014-10-26; OxoG 2016-4-28; VAGrENT v2.1.2; ANNOVAR v2014Nov12; VariantBAM v2017Dec12; SNV-Merge v2017May26; SV-MERGE v2017Dec12; DKFZ v2016Dec15

The method for combining p-values is available from https://github.com/broadinstitute/getzlab-PCAWG-pvalue_combination/. Power calculations are available from https://github.com/broadinstitute/getzlab-PCAWG-power_calculations. The method for identifying significantly recurrent breakpoints by controlling for covariates is available at: https://github.com/mskilab/fish.hook. The method for permuting rearrangement breakpoint pairs to identify covariates affecting rearrangement partner selection is available as the "swap" module of: https://github.com/walaj/ginseng. The method for identify significantly recurrent rearrangements is available as the "2D" method at: https://github.com/ofershapira/SVsig. The method for identifying cis-expression consequences of SVs, CESAM, is available at: https://bitbucket.org/weischen/cesam

For manuscripts utilizing custom algorithms or software that are central to the research but not yet described in published literature, software must be made available to editors/reviewers. We strongly encourage code deposition in a community repository (e.g. GitHub). See the Nature Research guidelines for submitting code & software for further information.

Data

Policy information about availability of data

All manuscripts must include a data availability statement. This statement should provide the following information, where applicable:

- Accession codes, unique identifiers, or web links for publicly available datasets
- A list of figures that have associated raw data
- A description of any restrictions on data availability

WGS somatic and germline variant calls, mutational signatures, subclonal reconstructions, transcript abundance, splice calls and other core data generated by the ICGC/TCGA Pan-cancer Analysis of Whole Genomes Consortium are available for download at https://dcc.icgc.org/releases/PCAWG. Additional information on accessing the data, including raw read files, can be found at https://docs.icgc.org/pcawg/data/. In accordance with the data access policies of the ICGC and TCGA projects, most molecular, clinical and specimen data are in an open tier which does not require access approval. To access potentially identification information, such as germline alleles and underlying sequencing data, researchers will need to apply to the TCGA Data Access Committee (DAC) via dbGaP (https://dbgap.ncbi.nlm.nih.gov/aa/wga.cgi?page=login) for access to the TCGA portion of the dataset, and to the ICGC Data Access Compliance Office (DACO; http://icgc.org/daco) for the ICGC portion. In addition, to access somatic single nucleotide variants derived from TCGA donors, researchers will also need to obtain dbGaP authorization.

Field-specific reporting

Please select the one below that is the best fit for your research. If you are not sure, read the appropriate sections before making your selection.

☒ Life sciences ☐ Behavioural & social sciences ☐ Ecological, evolutionary & environmental sciences

For a reference copy of the document with all sections, see nature.com/documents/nr-reporting-summary-flat.pdf

Life sciences study design

All studies must disclose on these points even when the disclosure is negative.

Sample size	We compiled an inventory of matched tumour/normal whole cancer genomes in the ICGC Data Coordinating Centre. Most samples came from treatment-naïve, primary cancers, but there were a small number of donors with multiple samples of primary, metastatic and/or recurrent tumours. Our inclusion criteria were: (i) matched tumour and normal specimen pair; (ii) a minimal set of clinical fields; and (iii) characterisation of tumour and normal whole genomes using Illumina HiSeq paired-end sequencing reads.<br>For analyses specific to this study, we generated sample subsets as described in the methods.
Data exclusions	After quality assurance, data from 176 donors were excluded as unusable. Reasons for data exclusions included inadequate coverage, extreme bias in coverage across the genome, evidence for contamination in samples and excessive sequencing errors (for example, through 8-oxoguanine).
Replication	In order to evaluate the performance of each of the mutation-calling pipelines and determine an integration strategy, we performed a large-scale deep sequencing validation experiment. We selected a pilot set of 63 representative tumour/normal pairs, on which we ran the three core pipelines, together with a set of 10 additional somatic variant-calling pipelines contributed by members of the SNV Calling Working Group. Overall, the sensitivity and precision of the consensus somatic variant calls were 95% (CI90%: 88-98%) and 95% (CI90%: 71-99%) respectively for SNVs. For somatic indels, sensitivity and precision were 60% (34-72%) and 91% (73-96%) respectively. Regarding SVs, we estimate the sensitivity of the merging algorithm to be 90% for true calls generated by any one caller; precision was estimated as 97.5% - that is, 97.5% of SVs in the merged SV call-set have an associated copy number change or balanced partner rearrangement.
Randomization	No randomisation was performed.
Blinding	No blinding was undertaken.

Reporting for specific materials, systems and methods

We require information from authors about some types of materials, experimental systems and methods used in many studies. Here, indicate whether each material, system or method listed is relevant to your study. If you are not sure if a list item applies to your research, read the appropriate section before selecting a response.

Materials & experimental systems

n/a	Involved in the study	
☒	☐	Antibodies
☒	☐	Eukaryotic cell lines
☒	☐	Palaeontology
☒	☐	Animals and other organisms
☐	☒	Human research participants
☒	☐	Clinical data

Methods

n/a	Involved in the study	
☒	☐	ChIP-seq
☒	☐	Flow cytometry
☒	☐	MRI-based neuroimaging

Human research participants

Policy information about studies involving human research participants

Population characteristics	Patient-by-patient clinical data are provided in the marker paper for the PCAWG consortium (Extended Data Table 1 of that manuscript). Demographically, the cohort included 1,469 males (55%) and 1,189 females (45%), with a mean age of 56 years (range, 1-90 years). Using population ancestry-differentiated single nucleotide polymorphisms (SNPs), the ancestry distribution was heavily weighted towards donors of European descent (77% of total) followed by East Asians (16%), as expected for large contributions from European, North American and Australian projects. We consolidated histopathology descriptions of the tumour samples, using the ICD-0-3 tumour site controlled vocabulary. Overall, the PCAWG data set comprises 38 distinct tumour types. While the most common tumour types are included in the dataset, their distribution does not match the relative population incidences, largely due to differences among contributing ICGC/TCGA groups in numbers sequenced.
Recruitment	Patients were recruited by the participating centres following local protocols.
Ethics oversight	The Ethics oversight for the PCAWG protocol was undertaken by the TCGA Program Office and the Ethics and Governance Committee of the ICGC. Each individual ICGC and TCGA project that contributed data to PCAWG had their own local arrangements for ethics oversight and regulatory alignment.

Note that full information on the approval of the study protocol must also be provided in the manuscript.
