# Supplementary material for: Analyses of non-coding somatic drivers in 2,658 cancer whole genomes
Source: Nature. 2020 Feb 5;578(7793):102–11. doi: 10.1038/s41586-020-1965-x (PMC7054214; doi:10.1038/s41586-020-1965-x)
Supplement: Supplementary file 3 — This file contains Supplementary Notes, including Supplementary Figures 1-15, a list of the Supplementary Tables (Supplementary Tables supplied as a separate spreadsheet), Supplementary references and a full list of participants in the PCAWG Consortium – see contents page for details. [file 41586_2020_1965_MOESM3_ESM.pdf]

Supplementary Notes for “Analyses of non-coding somatic drivers in 2,658 cancer whole genomes” by Rheinbay, Nielsen, Abascal, Wala, Shapira et al.

Table of contents

1. Overview of uncategorized non-coding hotspots in top 50 2

2. P value distributions under the null hypothesis for discrete statistics 3

3. Calibration of systematic element filters 4

4. Discussion of additional significant non-coding elements 8

5. Driver discovery in 2 kb genomic bins and mutational processes 15

6. Quantifying the performance of the pan-cancer breakpoint significance model 18

7. Distinguishing fragile sites from positive selection in regions of recurrent breakpoints 27

8. Comparison of known SCNAs and fusions with recurrent breakpoints and rearrangements 28

9. Amplification structure of the BRD4/NOTCH3 locus in the context of recurrent BRD4 deletion 30

10. Effect of element length on power calculations 31

11. Impact of covariates on the estimation of driver mutations in functional regions of cancer genes 34

12. Relative paucity of regulatory non-coding drivers at cancer genes 37

13. References 39

14. Participants in PCAWG Consortium 43

15. A list of Supplementary Tables 85

1. Overview of uncategorized non-coding hotspots in top 50

Six of the 35 non-coding hotspots, among the top 50, are not assigned to the mutational processes described in the main text (2 in the top 25 hotspots, **Fig. 1a**; 6 in the top 50 hotspots, **Extended Data Fig. 1b**). Below we describe these six hotspots in detail.

Four of the six remaining non-coding hotspots were found on the X chromosome (X:116579329, X:7791111, X:83966025, and X:83967552). The mutations in these hotspots were mainly contributed by males (61–94% of mutations per hotspot), suggesting the possibility of uncharacterized noise. Two of these hotspots (X:83966025, and X:83967552) are located in a self-chain region, where two stretches of approximately 400 bp (1000 bp apart) match each other except for 6 positions. All six of these positions have mutation calls in either or both of the regions. All but one of the 85 mutations in these positions match the reference base from the opposite region. Another one of these hotspots is located in palindromic DNA (X:7791111). The DNA sequence around this position is composed of two mononucleotide repeats of pairing bases (ATTTTTAAAAAAAAAT), which fits our definition of palindromic structures (**Methods**). The sequence context around this hotspot do not match the sequence recognized by APOBEC enzymes[1,2], and the hotspot had a low APOBEC signature probability (**Fig. 1a; Extended Data Fig. 1b**). It is therefore unlikely to be caused by the same mutational processes as the other palindromic hotspots in the top 50 hotspots, but rather likely represents noise associated with homopolymer runs (PCAWG variants paper). The last of these hotspots (X:116579329) has a median cancer allele fraction (CAF) of 0.21 (**Methods**), compared to a median CAF of 0.97 in the surrounding mutations in these patients. Generally, a CAF of one is expected on the X chromosome in males unless there are changes in ploidy in the region. The low CAF is thus consistent with a low frequency of mismatches caused by misaligned reads.

A hotspot (3:164903710) contained mutations in multiple cancer types, with most mutations contributed by Liver-HCC (6/17). It is located about 1 kb downstream of *SLITRK3*, two base pairs from an annotated CTCF transcription factor binding site. CTCF sites and the base pairs immediately downstream are known to be highly mutated in several cancer types including Liver-HCC. This position is lowly conserved, suggesting that it would not have a functional impact[3,4].

The last non-coding hotspot (1:103599442) had a high proportion of mutations attributed to the SBS5 signature. It overlaps a repetitive LINE region, suggesting potential mapping issues.

Moreover, the position is flanked by two other positions, two base pairs away in both directions, which also have mutations in the same samples and reads; however, these positions were called problematic in the Panel-of-Normals (PoN) filter, further suggesting mapping issues.

2. *P* value distributions under the null hypothesis for discrete statistics

When using continuous test statistics, the *P* value distribution under the null hypothesis is expected to follow a uniform distribution. However, when using discrete statistics (such as a function of the number of mutations in an element), with low counts, *P* values under the null can yield QQ plots with a point mass at *P* value = 1 (e.g., for elements with zero mutations), and the rest of the distribution below the diagonal, approaching it for small *P* values[5,6]. Thus, well-calibrated methods are expected to show QQ plots with values below the diagonal, in line with the results shown in **Extended Data Fig. 11**. In other words, discrete test statistics lead to sub-uniform distributions under the null ($\Pr[P_i \leq t \mid H0] \leq t$). For exactly this reason, the Benjamini–Hochberg method is still applicable to discrete data, although the resulting *Q* values will be conservative[55,6]. We note, however, that slightly conservative *Q* values are not undesirable in this study.

These considerations still apply to the integrated *P* values, at least under usual conditions. As described in **Methods S7**, Fisher's method uses the product of *P* values to calculate a statistic that follows a Chi-squared distribution ($X^2 \sim -2\Sigma \ln(p)$). Thus, when using conservative *P* values, the resulting X^2 statistic will be lower than that under non-conservative *P* values, resulting in a conservative integrated *P* value distribution. Brown's method is an extension of Fisher's method and thus will likely yield conservative *P* values, although the data-driven estimation of the covariance matrix between methods introduces some uncertainty. Nevertheless, the performance analyses shown in **Extended Data Fig. 3** reveal that the integrated approach performs well across datasets and tends to outperform individual methods, in terms of overall sensitivity and specificity.

3. Calibration of systematic element filters

The performance of the driver discovery methods depends on how well their null models capture the mutational processes at play. Some mutational processes are hard to capture accurately. This includes localised mutational processes that affect some regions more than others. When localised mutational processes, not included in the null models, affect a given region and contribute significantly to the observed mutations, the region may be called as a false positive by the driver discovery methods. Though regions affected by localised mutational processes may be true drivers, it is challenging to distinguish them from neutral regions with passenger mutations, which *a priori* are expected to dominate.

To take a conservative approach, we identify and filter elements if a substantial amount of the mutations appear to be derived by localized mutational processes. This approach is applied to (i) activation-induced cytidine deaminase (AID)-derived mutations in lymphoma samples; (ii) UV-derived mutations in melanoma; and (iii) APOBEC-derived mutations in all cohorts.

For the lymphoma cohorts, we first apply a threshold for calling mutations as AID based on the distribution of AID signature probabilities for all lymphoma mutations **(Supplementary Fig. 1a**). We next investigate what fraction of elements would be filtered at a given threshold for the fraction of permitted AID mutations (**Supplementary Fig. 1b**). The chosen threshold is trade-off between maximizing the number of filtered elements overlapping a set of known AID off-target regions[7] and minimizing the number of filtered coding elements in the Cancer Gene Census[8] (CGC).

A similar approach was used for melanoma mutations **(Supplementary Fig. 2)** to provide a filter for localized UV mutations known to be enriched in sites escaping nucleotide excision repair[9,10], and for mutations in all cohorts to provide a filter for mutations caused by APOBEC enzymes (**Supplementary Fig. 3**).

Given that APOBEC editing appears to target DNA-level palindromic sequences in several cases[1,11], we evaluated if SNVs in driver candidate RNA genes with palindromic sequences (*RMRP, RPPH1, RNU5A-1, RNU6-573P,* and *mir-142*) could be explained by APOBEC editing. Only 9.7% of SNVs had posterior APOBEC probability >0.5, suggesting that APOBEC editing contributed only a small fraction of their SNVs.

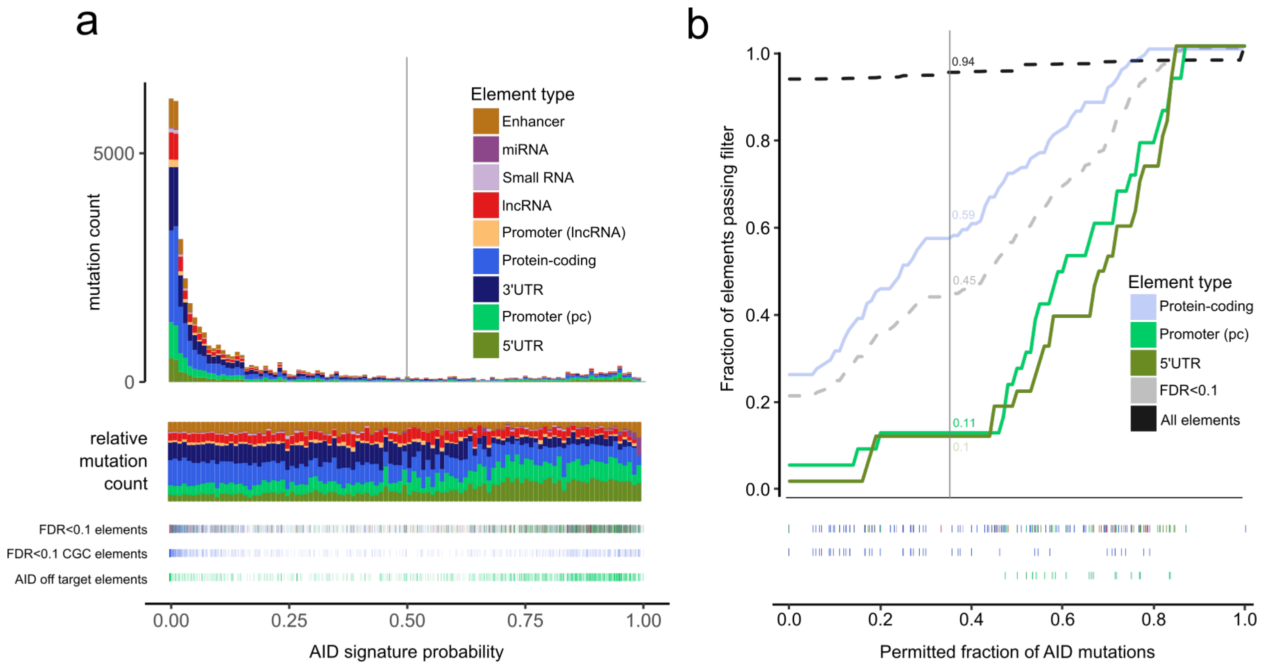

Supplementary Figure 1: a, Top: distribution of AID signature probability for mutations in all tested elements divided into element types. The AID call threshold (0.5) used for mutations is indicated with a gray line. Middle: The relative distribution among element types. Bottom**:** AID signature probabilities for mutations in significant (Benjamini–Hochberg FDR Q < 0.1) elements (67% mutations above threshold), in significant known cancer elements from CGC (44% mutations above threshold), and in known AID off-target promoter elements (76% mutations above threshold). **b**, Top: Fraction of elements passing the AID filter at different thresholds of permitted AID called mutations. (fraction filtered: 57% significant elements; 27% significant known cancer elements; 100% AID off-target promoter elements). A gray line indicates the filter threshold (0.35). Bottom: Fraction of AID mutations in elements as defined in (a).

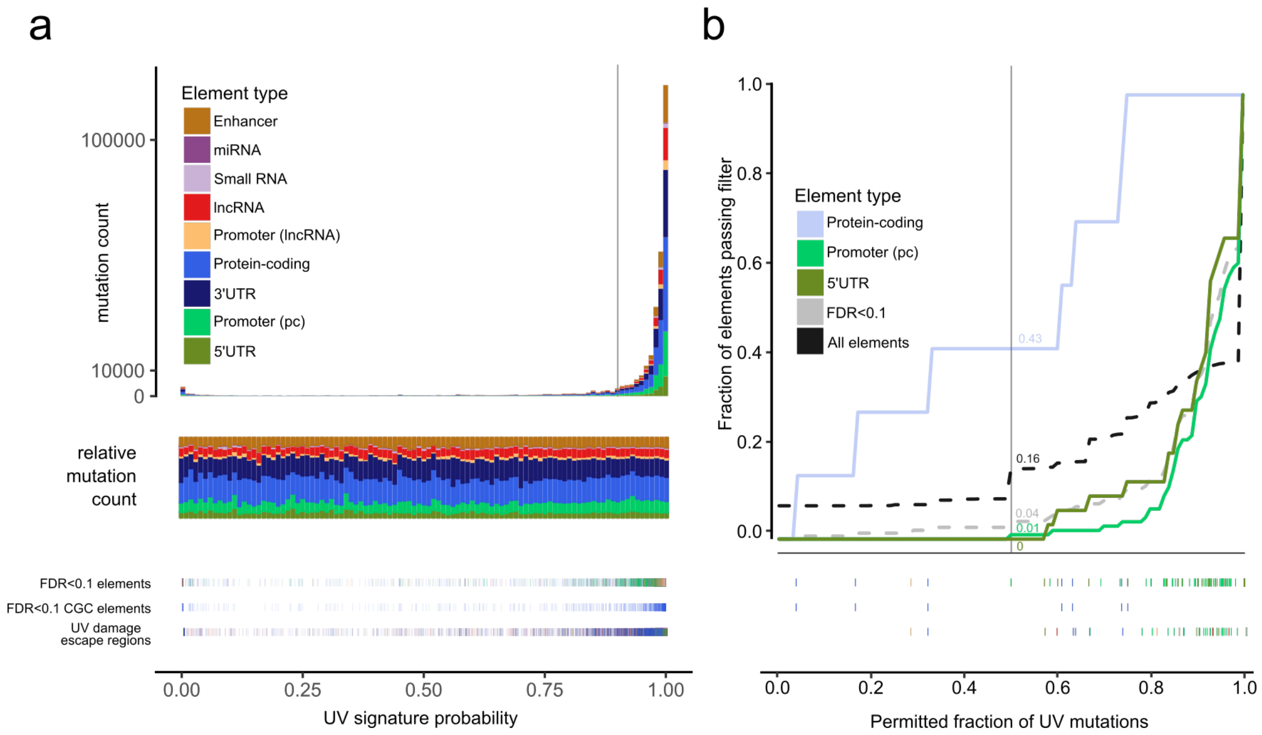

Supplementary Figure 2: a, Top: Distribution of UV signature probabilities for mutations in all tested elements divided into element types. The UV call threshold (0.9) used for mutations is indicated with a gray line. Middle: The relative distribution among element types. Bottom**:** UV signature probabilities for mutations in significant (Benjamin–Hochberg FDR<0.1) elements (86% mutations above threshold), in significant known cancer elements (71% mutations above threshold), and in regions escaping repair of UV induced damage[9] (87% mutations above threshold). **b**, Top: Fraction of elements passing the UV filter at different thresholds of permitted UV called mutations (fraction filtered: 96% significant elements; 57% significant known cancer elements; 97% UV damage escape overlapping elements). A gray line indicates the filter threshold (0.5). Bottom**:** Fraction of UV mutations in elements as defined in (a). UV damage escape elements are defined to contain one or more mutations in a UV damage escape region.

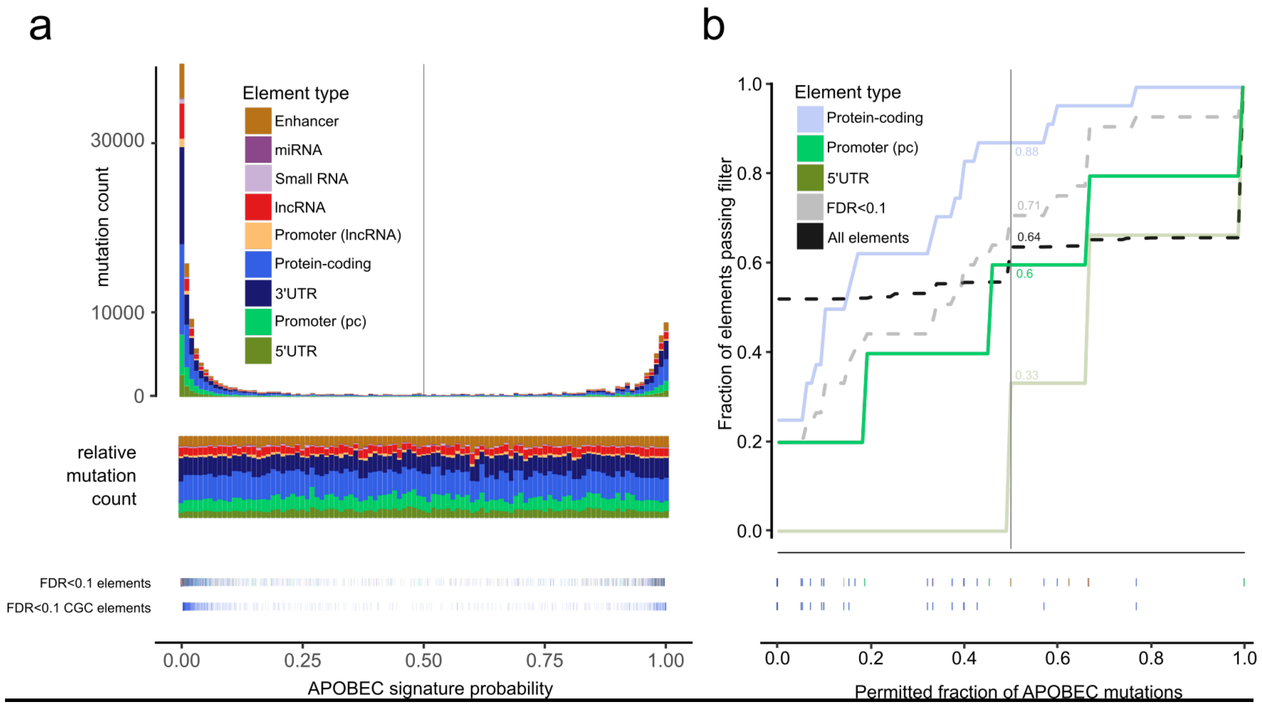

Supplementary Figure 3: a, Top: Distribution of APOBEC signature probabilities for mutations in cohorts with an average APOBEC signature probability above 0.3 (Bladder, Uterus, Head, Cervix, and Breast cancers) in all tested elements divided into element types. The APOBEC call threshold (0.5) used for mutations is indicated with a gray line. Middle: The relative distribution among element types. Bottom**:** APOBEC signature probabilities for mutations in significant (Benjamini–Hochberg FDR<0.1) elements (38% mutations above threshold) and in significant known cancer elements (32% mutations above threshold). **b**, Top: Fraction of elements passing the APOBEC filter at different thresholds of permitted APOBEC called mutations (fraction filtered: 29% significant elements; 10% significant known cancer elements). A gray line indicates the filter threshold (0.5). Bottom**:** Fraction of APOBEC mutations in elements as defined in (a).

4. Discussion of additional significant non-coding elements

Promoters:

WDR74

The *WDR74* promoter has already been suggested as a potential driver in several studies[11–14]. However, we found that mutations are concentrated inside a *U2* RNA where the density of putative polymorphisms is abnormally high (**Supplementary Fig. 4a** below). This outstanding level of diversity could in principle be due to higher mutability in the germline. However, given the repetitive nature of the *U2* element (there are hundreds of copies in the genome) and the extreme levels of putative diversity, it is more likely that this region is a source of mapping artifacts.

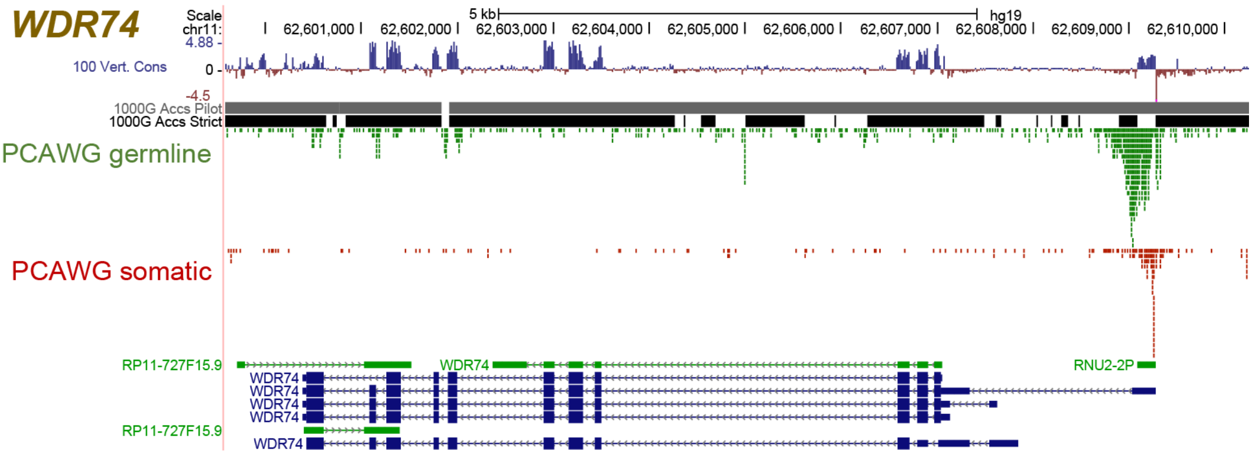

Supplementary Figure 4. PCAWG germline and somatic mutations overlapping a *U2* element in the promoter of *WDR74*.

HES1

HES1 promoter mutations were significant in Carcinoma and Pan-cancer, but showed no association with gene expression (**Extended Data Fig. 4a**). *HES1* is a NOTCH signalling target[15], and is focally amplified in gastric cancers (**Extended Data Fig. 4b**).

IFI44L, HIST1H2AM, GALNTL5, ZSWIM6, SDCCAG8/CEP170, and *POLR3E*

Tumors with mutations in the promoters of *IFI44L, GALNTL5, ZSWIM6, POLR3E* showed trends towards increased or decreased expression, although the small number of samples prevented us from drawing definitive conclusions (**Extended Data Fig. 4a**). *HIST1H2AM* lacks expression data as part of the histone family of genes, which are not polyadenylated and therefore not

represented in the RNA-seq libraries, and *SDCCAG8* is mutated in a cohort that lacks expression data. Both *GALNTL5* and *SDCCAG8* are located in an amplification peak in glioblastoma (http://portals.broadinstitute.org/tcga/gistic/browseGisticByGene). However, *SDCCAG8* is proximal to *AKT3*, which is likely the true driver of this focal amplification. The other recurrently mutated genes were not present in significantly amplified or deleted focal peaks (**Methods**). Validation of these hits with additional data in further studies will be needed to evaluate whether they are genuine drivers.

Enhancers:

TP53TG1

An enhancer near *TP53TG1* — an RNA gene suggested to be a p53-associated tumor suppressor epigenetically silenced in cancer[16] — reached significance in several cohorts. Mutations concentrated around a conserved region (**Supplementary Fig. 5**) and overlapped sites bound by NFIC and ZBTB7 transcription factors in HepG2 cells[17]. Ten of 16 mutations in this region were contributed by esophageal cancers: one di-nucleotide variant (DNV) and nine SNVs, of which five had a signature 17 contribution of >0.5 (the remaining four had contributions between 0.20 and 0.49), raising the possibility that these reflect a localized mutational process rather than a driver event[18].

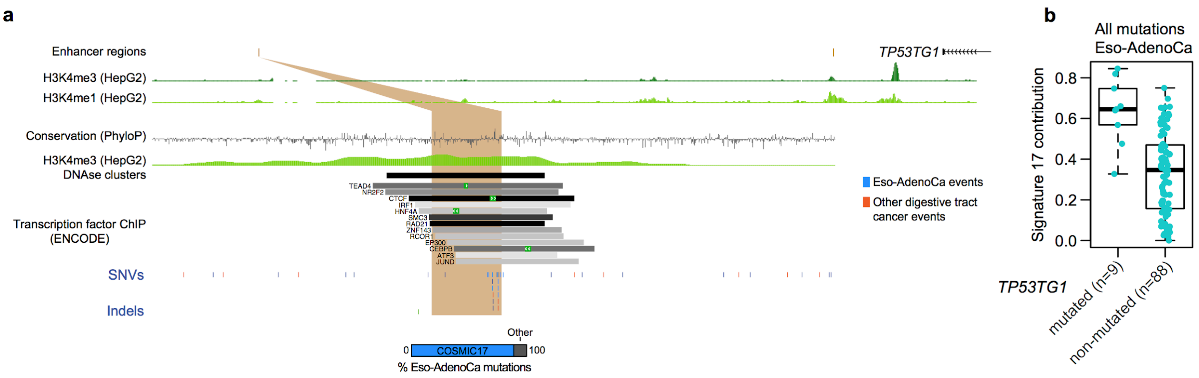

Supplementary Figure 5: a, An enhancer associated with *TP53TG1* contains mutations mostly attributed to the COSMIC17 mutational signature associated with esophageal cancer. **b,** Boxplot showing the contribution of signature 17 in Eso-AdenoCa for TP53TG1 mutated and non-mutated samples. Boxes show the interquartile range and median.

Non-coding RNAs:

RMRP (additional information)

RMRP is presented in the main text, and we here provide some further details and results of the mutational analysis. The non-coding RNA *RMRP* is significantly mutated in multiple cancer types, in both its gene body and promoter (**Fig 1b; Extended Data Fig. 5c; Supplementary Table 5**). The gene body mutations (7 SNVs in pan-cancer) show a significant bias towards high structural impact (rank-sum test, $P = 0.011$). Three of these are individually significant (each with $P < 0.1$, sample level permutation tests; **Extended Data Fig. 5c**). Of the four gene-body indels, three are located in or near protein-binding sites ($P = 0.08$), including a deletion that is predicted to affect the secondary structure. Given *RMRP*'s role in replication of the mitochondrial genome[19–22], we tested whether mutations in this locus were associated with altered mitochondrial genome copy number. Indeed, mutated samples showed a trend towards higher mitochondrial copy number (two-sided rank-sum test, $P = 0.1$).

The high density of SNPs in the *RMRP* locus suggests that the *RMRP* locus may be either subject to an elevated germline mutation rate or to unidentified technical issues (**Supplementary Fig. 6a**). The consequence of somatic mutations should therefore be interpreted with caution.

Fifteen ncRNAs hits not described in detail in the main text passed the systematic post-filter (*RPPH1*, *G025135*, *CTD-2105E13.15*, and *G029190* lncRNAs; *MIR663A*, *RNU12*, *RP5-997D16.2*, and *RP11-440L14.1* lncRNA promoters; *RNU5A-1, RNY1, RNY5, and RNU6-573P* small RNAs; Ala.TGC [id.trna.192], Met.CAT [id.trna.419], and Gly.GCC [id.trna.336] tRNAs). The driver role of these were generally not supported by additional lines of evidence, lacked functional evidence, or appeared to be affected by technical artifacts. The candidates are described below, and individual *Q* values are in **Supplementary Table 5**.

RPPH1

RPPH1 forms the RNA component of the RNase P ribonucleoprotein, which matures precursor-tRNAs by cleaving their 5' end[23]. It is transcribed from a divergent promoter together with the protein-coding gene *PARP2*; however, no association with expression was observed for either gene. It was found significant in Adenocarcinoma, Carcinoma, Digestive tract tumors, and Pan-cancer. The region has a high level of germline polymorphisms, i.e., the SNP density is 5.1

times higher within the region compared to 5 kb flanking regions (inside/outside SNP density enrichment), which indicates mapping issues or a higher germline mutation rate (**Supplementary Fig. 6b**).

G025135

G025135 is a lncRNA element (from MiTranscriptome[24]) identified here as a significant driver candidate in Lymph-CLL. This ncRNA element was also found in Lymph-BNHL, Lymphomas and Hematopoietic system tumors, where it was filtered based on a high proportion of AID mutations. In Lymph-CLL, the number of AID mutations was just below our threshold. Furthermore, some individual patients have many mutations, further supporting that it may be a target of AID.

MIR663A

The promoter of *MIR663A* was recurrently mutated in Carcinoma. It is the primary transcript of *MiR-663*, which has tumorigenic functions in gastric cancer and nasopharyngeal carcinoma[25]. The mutations were not correlated with expression. Unexpectedly high SNP density in this element suggests the presence of mapping artefacts.

RNY1

RNY1 encodes Y1 that is a Y RNA. Y RNAs constitutes the RNA part of ribonucleoproteins and they are required for chromosomal DNA replication[26]. It was found significant in Digestive tract tumors. The expression of Y1 has previously been found elevated in several types of cancer, including colorectal cancer[27]. The inside/outside SNP density enrichment is 5.6, suggesting mapping issues or a high germline mutation rate.

Other ncRNAs

Five functionally uncharacterised ncRNAs were found significant in one, two, or three cohorts, but we could not find any further supporting driver evidence: (1) The lncRNA *CTD-2105E13.15*, which partially overlaps three exons of *TMEM190*, was found significant in Carcinoma and Pan-cancer. (2) The *G029190* lncRNA (from MiTranscriptome) had significance in Adenocarcinoma and Pan-cancer. (3) The promoter of *RP5-997D16.2* was found significant in Prost-AdenoCa, and it has a hotspot with two mutations in this cohort. (4) The promoter of *RP11-440L14.1* was significant in Ovary-AdenoCa, Carcinoma, and Pan-cancer. It has a hotspot position with four mutations overlapping two different deletions. Finally, (5) the small RNA *RNU6-573P* was

detected in Panc-Endocrine with three mutations. However, it appears to be a nonfunctional pseudogene recently inserted in the human lineage. Not only the locus, but the wider region is subject to increased mutational burden, further supporting that mutational mechanisms or technical issues rather than selection underlies the mutational recurrence.

In addition, six ncRNA hits overlap regions with high densities of germline mutations, which suggests mapping issues or high germline mutation rates. These include (1) the promoter of the lncRNA *RNU12* (Lymph-BNHL, Lymphomas and Hematopoietic system tumors; inside/outside SNP density enrichment: 8.7; **Supplementary Fig. 6c**); (2) the small RNAs *RNY5* (Adenocarcinoma and Digestive tract tumors; inside/outside SNP density enrichment: 4.5) and (3) *RNU5A-1* (Adenocarcinoma; inside/outside SNP density enrichment: 5.4); the tRNAs (4) Ala.TGC (Adenocarcinoma and Carcinoma; inside/outside SNP density enrichment: 3.7), (5) Met.CAT (Digestive tract tumors; inside/outside SNP density enrichment: 2.9), and (6) Gly.GCC (Female reproductive system tumors; inside/outside SNP density enrichment: 7.7).

Finally, the promoter of the lncRNA *RNU12* overlap the promoter of *POLDIP3* without significant expression-correlation in any of the cohorts.

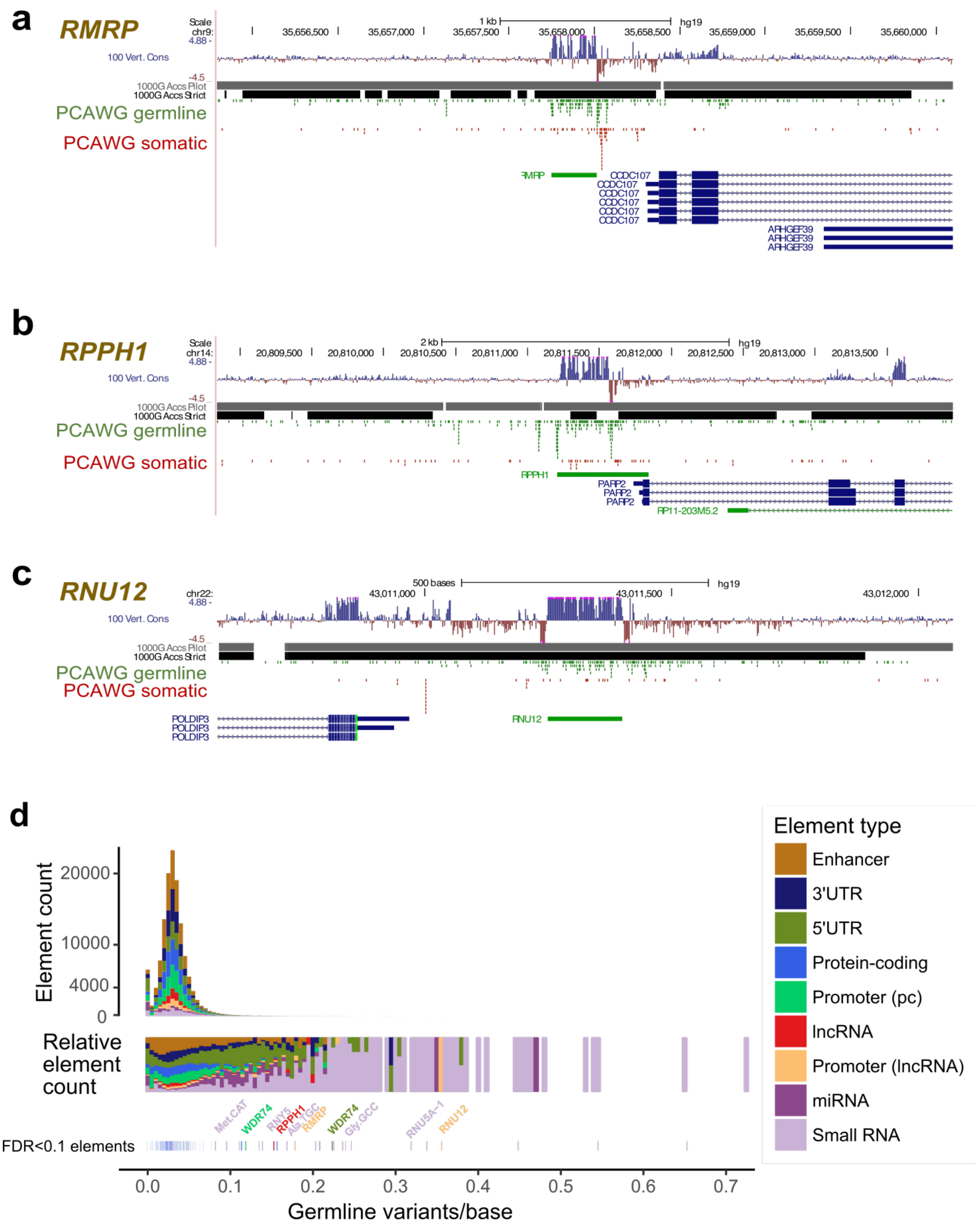

Supplementary Figure 6: The non-coding RNA candidates (**a**) *RMRP,* (**b**) *RPPH1,* and (**c**) *RNU12* all had high SNP densities. **d**, SNP density among element types. (Top) Stacked distribution of SNP densities for different element types. (Middle) Relative distribution among element types for given SNP density. (Bottom) Distribution of significant (Benjamini–Hochberg

FDR<0.1) elements. Non-coding RNA candidates generally have high densities of SNPs, including the candidates shown in **a-c**, amongst the highest in genome, which raises concerns about the relevance or authenticity of the somatic mutations hitting these loci.

5. Driver discovery in 2 kb genomic bins and mutational processes

The paucity of non-coding driver mutations identified in our analyses could in principle be due to the list of functional elements used, which cover only a small fraction of the genome. We partially addressed this limitation in the unbiased analysis of hotspots, finding no convincing non-coding driver candidates other than the *TERT* promoter. Here we interrogate the rest of the genome in a similarly unbiased way. We divided the genome in 2-kb bins and looked for bins with an excess of mutations, while accounting for sequence composition and trinucleotide substitution rates (NBR method). To avoid the risk of mapping artifacts, we first excluded bins with potential mappability problems, considering a total of 1.2M bins covering 66% of the genome (**Methods**). This analysis was performed on all cohorts and meta-cohorts of more than 100 samples, using a global FDR adjustment across all of them. Many bins were recurrently mutated in lymphoid tumors, likely due to AID-derived localized somatic hypermutations. Lymphoid tumors were therefore excluded from the analysis. The NBR method without covariates identified 67 significantly mutated bins across meta-cohorts and tumor types (178 significant hits in total; **Extended Data Fig. 5a; Supplementary Table 9**). 22 bins (74 hits) overlap 17 known cancer drivers, including *TP53* (28 hits), *KRAS* (6), *APC* (5) or *ARID1A* (5). Of the remaining significant bins, 29 (87 hits) overlap genes and regions affected by localized mutational processes. Mutations in one of these bins (4 hits) lie within a CTCF binding site; the rest of the bins overlap highly transcribed loci and are rich in indels, particularly of length 2–5 bp (**Fig. 2e; Supplementary Table 9**), hence likely being associated to the transcriptional mutational process described for *ALB*, *MALAT1*, *NEAT1,* and other genes.

Interestingly, *MIR122*, a known tumor suppressor, overlapped significantly mutated bins in liver. However, the scattered distribution of mutations along several consecutive bins and the enrichment of indels (especially of length 2–5 bp) suggests that this locus is also affected by the transcriptional mutational process described in the manuscript (**Supplementary Fig. 7a; Fig. 2e**) — indeed, *MIR122* is one of the most highly transcribed genes in liver[28,29]. To explore the potential functional implications of these mutations, we investigated if *MIR122* mutations are associated with altered expression. Since only two mutated samples had miRNA expression profiling, we did this by associating mutations with different expression of *MIR122* target genes by integrating expression across genes with *MIR122* target sites in their 3'UTRs[4]. Mutations were associated with a weak non-significant decrease in target gene expression. This provided no convincing evidence that mutations may be associated with decreased *MIR122* expression (**Supplementary Fig. 7b**). No single mutation hit the miR-122, and the indel hotspot occurs in a

region with four direct AAG repeats (**Supplementary Fig. 7a**). Overall, it is thus plausible that most mutations in *MIR122* are passenger mutations resulting from the mechanism of transcription-associated mutagenesis described for *MALAT1*, *NEAT1,* and *ALB*, among others.

Within the remaining 16 bins (17 hits) that were not associated to known drivers or mutational processes, some appear to be caused by mapping artefacts. For instance, the bin chrX:83966001-83968000 contains a duplication-inversion (chrX:83966018-83966396 and chrX:83967485-83967863) with somatic mutations hitting divergent sites between the two duplicated regions. Other unreliable cases include a bin overlapping an olfactory receptor in a region with very high germline SNP density, and four bins with mutations in LINE elements and poor mappability according to 1,000 Genomes masks (**Supplementary Table 9**). For the rest of the significant bins (10), we found no obvious potential association to cancer, as most were far from genes in regions with no apparent regulatory functions, while others were borderline significant.

One limitation of this binned approach is that the signal of mutational recurrence may become diluted in the relatively long bins. We conducted a complementary analysis focusing on non-coding ultraconserved regions, of which only 36% overlapped our defined functional elements. Here, regions are less numerous (n = 4,351) and much smaller (mean length 325 bp), avoiding the dilution of the signal and increasing the statistical power, though the fraction of the genome covered is largely restricted (1.4 Mb; 0.05%). None of the ultraconserved regions tested were significant (**Extended Data Fig. 5e**). Overall, these results suggest that drivers are rare in the fraction of the genome not represented in our definition of functional elements.

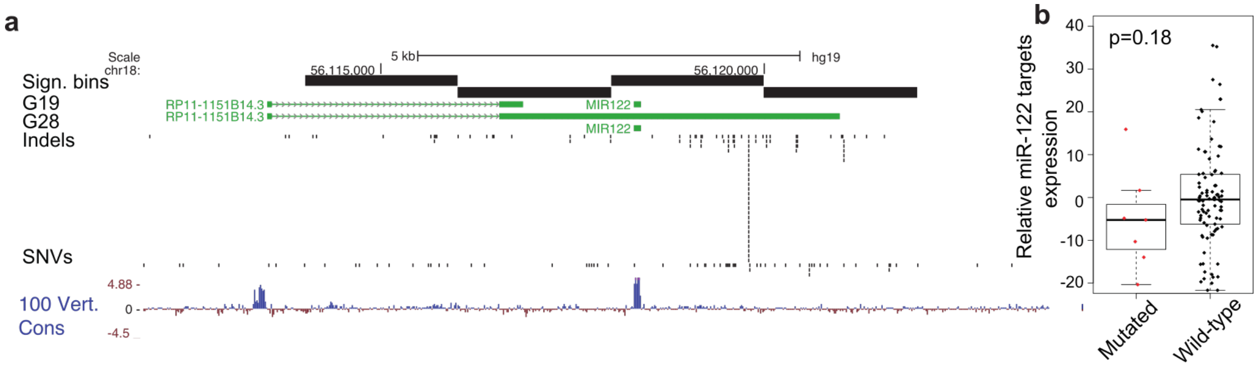

Supplementary Figure 7: **a,** Scattered distribution of mutations in the *MIR122* locus in Liver-HCC across four different 2-kb bins. **b,** Lack of enriched expression of genes containing miR-

122 target motifs in their 3’UTRs between tumors mutated (n = 7) in *MIR122* and the corresponding wild type (n = 93). For each sample, we evaluated if target genes show higher expression than non-target genes given the overall gene expression in the cohort. Lower expression of target genes leads to a negative value indicative of a correspondingly higher activity of *MIR122*[30]. Box plots indicate inter quartile range with median lines. *P* values evaluated with two-sided Wilcoxon rank sum test.

6. Quantifying the performance of the pan-cancer breakpoint significance model

Background and Motivation

For tumors with very low burdens of somatic alterations, the probability that a single gene would be recurrently altered simply by chance is low. Barring confounding by technical artifacts, in very simple cancer genomes, it is relatively straightforward to conclude that recurrent alterations reflect evidence of positive selection by the tumor. However, most tumors acquire a vast number of alterations throughout their evolution, and distinguishing “driver” alterations (those that are positively selected for in cancer evolution) from “passenger” alterations (those that are under neutral or negative selection) is more challenging. This is likely more challenging for SVs than point mutations, for reasons given below.

Building a background model: positive selection, negative selection, and mechanism

The ideal background model would account for every biological process (mechanism) that generates alterations, thereby perfectly recreating the expected distribution under neutral selection and allowing for accurate identification of the effects of positive selection. Currently, such strong *a priori* predictions of the background distribution are not sufficient to develop background models owing to our still limited understanding of each possible mechanism by which cells sustain somatic alterations as well as the vast heterogeneity in tumor tissue of origin, germline background, and environmental exposures.

To overcome our limited ability to predict alteration distributions from first principles, background models are typically estimated from the observed alteration distribution. The most naive background model would simply assume as the background distribution the observed distribution. In this scenario, no sites of positive selection would be identified because the difference between the observed and expected at each gene would be zero by definition. Instead, since the distribution of alterations is a combination of the mechanisms by which alterations form and selective pressures, a more useful background model would capture the genome-wide *patterns* found in the observed data, but without overfitting and washing out signal from true positive selection. To do this, background models are parameterized by known mechanisms of alteration formation (e.g., a tendency for errors to accrue in regions of late replication timing), while the effect sizes of each alteration are estimated from the observed data. This method carries the

assumption that the vast majority of alterations are passenger alterations (i.e., determined solely by mechanistic explanations rather than selection), rather than drivers. This appears to largely be the case, as the average number of driver alterations per tumor is estimated to be fewer than ten[31] while the number of total alterations per tumor can be in the tens of thousands[32]. However, such assumptions break down for very low mutation tumors like leukemias and pediatric malignancies, in which case the background model can be assumed from other tumor types. It should be noted that the assumption that passenger alterations are truly under neutral selection is being questioned[33].

Somatic alterations that disrupt essential cellular functions, expose a cancer cell to immune surveillance, or in any other way decrease the fitness of a cell, are said to be under negative selection. Negative selection is particularly challenging to estimate, as it is marked by the absence of alterations. In principle, with enough biological knowledge about cellular function, one could predict *a priori* whether an alteration affecting a given gene will decrease cellular fitness. In practice, as with modeling the effects of mechanism, understanding the effects of negative selection requires an interplay between known biological processes (e.g., essential genes) and tracking backward from the observed data. Distinguishing the effects of mechanism from negative selection is difficult, as there is no known bound on the number of genes / loci that could be under negative selection. Recent studies evaluating SNVs indicate minimal negative selection in regions with two or more copies[34]. However, SVs were not evaluated in these studies. Without explicit accounting for negative selection, the effect sizes in the background model obtained from modeling the observed data will be a combination of mechanism and negative selection.

Detection of somatic alterations relies on a technical pipeline that includes sample collection; library preparation and sequence or microarray generation; read mapping to the reference genome; and finally variant calling and filtering. As such, the observed distribution of alterations is not only shaped by mechanism and selection, but by the technical biases inherent to any detection pipeline. In practice, such technical biases tend to surface quickly, as regions that have a high number of false positive variant calls will tend to deviate significantly from the background model and come to early attention. However, more subtle technical variations (e.g., from batch effects[35]) are still possible and must be accounted for whenever identified.

Considering the above effects, when compared with the density predicted by the background distribution, the density of an alteration could be observed to be at a higher rate than predicted

for four reasons: (i) there are unaccounted for mechanistic reasons for a higher alteration density at a certain locus; (ii) there is a relative lack of negative selection at a site compared with similar loci elsewhere in the genome; (iii) there is a systematic technical artifact; or (iv) the alteration confers a growth or survival advantage to a cell and it is under positive selection. One approach to testing the robustness of significant "hits" is to determine how high the true background rate would have to be for the locus to become insignificant. For example, most RSJs detected in our analysis would remain significant even if the background rate were twice as high as we have estimated (**Extended Data Fig. 9a**)

Driver alterations have been systematically distinguished from passenger alteration using pan-cancer data for both somatic SNVs/indels[32,36–38] and for SCNAs[39,40]. However, to our knowledge, this has never been done for rearrangements. We decided to use a similar overall approach, but with new methods that account for the specific challenges of rearrangements. In our approach, we first build a model to account for as much of the variation in the breakpoint distribution as possible, similar to approaches that have used replication timing and gene expression to account for variance in the somatic SNV distribution[32]. Our approach uses an expanded set of covariates that have more relevance for breakpoints (e.g., fragility, repetitive elements). We further build a model that accounts for the two-dimensional connections that are unique to rearrangement data, and utilize this to discover recurrent fusions.

Accounting for stochastic variation

In any finite dataset, the fraction of samples exhibiting a rearrangement ("rearrangement rate") at any single locus will differ from the actual prevalence of rearrangements at that locus, due to random chance. We estimate the level of stochastic variation in our dataset by comparing rearrangement rates determined from half the samples (chosen at random) to rearrangement rates determined from the other half, to generate a correlation coefficient (R); R^2 roughly corresponds to the fraction of the variation in rearrangement rates that can be attributed to random chance, and therefore cannot be predicted. We then calculate the fraction of the "predictable" variation that can be attributed to known mechanistic and selective sources of variation, and to unknown sources.

Given the size of the genome, and the relatively small number of rearrangements in our dataset, it is uncommon for a single base to be involved in a rearrangement twice. Therefore, when

comparing rearrangement rates between datasets at a given “locus”, we aggregated all rearrangements in a “bin” encompassing that locus. Specifically, we divided the genome into a set of bins, and compared rates of rearrangements within each bin between datasets. We also determined the expected rates of rearrangement at the bin level from our mechanistic models, and considered loci under selection to involve the entirety of the bin encompassing them.

This bin size can determine the amount of variation one attributes to stochastic, mechanistic, and selective forces. Larger bins will aggregate larger numbers of rearrangements, and therefore appear to exhibit less stochastic variation. In contrast, larger bins will also average over more widely varying sources of mechanistic and selective variation, reducing the apparent variation attributed to each of these sources. Therefore, the specific R^2 values we report need to be considered in light of the bin size, or resolution of the analysis.

We performed three analyses to determine the quality and robustness of the pan-cancer breakpoint significance model. First, we used the Akaike Information Criteria (AIC) to evaluate the quality of the model and to avoid over-fitting. Second, using an R^2 coefficient, (the squared Pearson correlation coefficient) between the predicted breakpoint counts per bin and the observed breakpoint counts as a metric for the goodness-of-fit, we estimated the relative effects of breakpoint formation mechanism, positive selection, stochasticity, and statistical power to explain the distribution of breakpoints in our cohort. Third, we compared the breakpoint model with a somatic SNV model. In addition, we accounted for additional sources of non-stochastic variation that were not accounted for in our model by using a Gamma-Poisson function to estimate the distribution of rearrangement breakpoints per bin. This fits the observed data better than a simple Poisson distribution (**Supplementary Fig. 8a**).

Evaluating model performance with AIC

To determine the covariates to use in the optimal pan-cancer model, we used forward selection to create a set of nested models reflecting different combinations of 28 genomic covariates. We generated a one-covariate model by searching all 28 covariates for the one covariate that minimized AIC. We then searched for a second covariate out of the remaining 27, which minimized the AIC to add to the one covariate model. We repeated this process until we had all 28 covariates in the model. We standardize the AIC by subtracting the minimized AIC at each model size by the minimum AIC over all model sizes. Each model is then ranked by this value

(AIC - minimum AIC), which we define as delta_i, where i denotes the model size. **Supplementary Fig. 8b** shows the AIC for the best model at all possible model sizes. According to and Burnham & Anderson[41], a delta_i of less than seven indicates a covariate that may be adding significant information. We therefore used this cutoff, resulting in the selection of seven covariates: Hyper-Fragility, Fragility, Gene Density, SINE, Mappability, Expression, and Weak Repressed Polycomb. Finally, we provide a quantile–quantile (QQ) plot comparing the observed *P* value distribution to the expected distribution (**Supplementary Fig. 8c-d**). The strengths of these covariates differed slightly, but reproducibly, between cancer types, as can be observed by clustering together two independent breast cancer datasets (**Supplementary Fig. 8e**).

Estimating the effect size of positive selection, mechanism, and stochasticity in the pan-cancer breakpoint model

As described above, the distribution of breakpoints in the cancer genome is influenced by the physical mechanisms by which they are formed; by the effects of positive and negative selection; and by inherent stochasticity. We calculated a goodness-of-fit metric (Pearson R^2) and estimated how much of it could be attributed to positive selection and inherent breakpoint stochasticity in addition to fragile sites and novel breakpoints.

We next evaluated the source of the missing explanatory power between a perfect model and our pan-cancer model. A model that perfectly predicts the distribution of breakpoints would have an R^2 of 1.0. However, we expect that inherent randomness in the physical process of breakpoint production (e.g., random thermodynamic fluctuation in DNA conformation) implies an inherent upper limit to the predictive power of a model in a finite dataset. Further, because the statistical power to distinguish stochasticity from true effects is dependent on the size of the dataset, the size of the dataset itself contributes to an upper bound on the goodness-of-fit for even a perfectly specified model. The breakpoint distribution itself is a reflection of the combined effects of selection, mechanism and stochasticity.

To estimate the effect of stochasticity versus mechanism and selection, we randomly divided the sample set into two halves and calculated the fraction of breakpoints in each genomic bin separately for each half. We then calculated the R^2 between these measurements, hereafter termed “self-correlation”. Self-correlation provides an estimate for the stochasticity in the data

because the effects of selection and mechanism should be the same across two random halves of the data and contribute to a higher R^2. Conversely, stochasticity should generate variations between random halves of the data, leading to lower R^2. Using this procedure and a bin size of 50 Kbp, we obtained an R^2 of 0.510 (+/- 0.02, S.D. of random samplings).

This is similar to the expected level of stochasticity in a dataset of our size. We used our mechanistic model that predicts breakpoint density from genomic features to simulate breakpoints perfectly consistent with the model. In a simulated dataset with 584,506 breakpoints, as in our actual dataset, we obtained an R^2 of 0.56 when comparing the simulated breakpoint counts to the known model.

To obtain an estimate of the effect of positive selection, we took the bins with significantly recurrent breakpoints (FDR < 0.1) as predicted by the pan-cancer GP model and re-ran the model using them as a covariate. Because the significant bins could either be from positive selection or unaccounted-for mechanisms, we used only the significant bins that were within 500 Kbp of a known cancer gene as defined by the Cancer Gene Census[8]. This provided 31 bins (**Supplementary Table 17**). Adding these as covariates to the pan-cancer model, we obtained an R^2 of 0.30 (increased from 0.22). Thus, we find that a substantial fraction of the remaining explanatory power of our model is due to positive selection. We also obtained estimates of the effect of fragile sites and novel RSBs using a similar method. We found that adding RSBs (known and novel) increased the R^2 to 0.36.

Comparison between the GP breakpoint model and GP SNV model

We next sought to compare the performance of the breakpoint model with a similar model for SNVs. To build a GP model for SNVs, we used replication timing, gene expression, GC%, heterochromatin, and mappability as covariates, which have been shown to account for a majority of the non-selection variance in somatic SNV distribution[32]. (Note that this model, designed to mirror the SV background model, is different from the models used in this manuscript for SNV driver discovery.) We used 44.6 million somatic SNVs called by the PCAWG consortium on the same genomes used for the breakpoint analysis. This produced an R^2 of 0.669. To estimate the limit of what could be explained given a perfect model operating on this dataset, we calculated the self-correlation to be 0.964. When operating on a downsampled dataset of 580,00 SNVs (to match the number of breakpoints), the R^2 for the full model becomes 0.460, with a self-correlation

of 0.509. We conclude that we are better able to account for the distribution of SNVs than SVs. Note that for both SNVs and SVs, our analyses are restricted to the mappable genome. It is possible that SVs are more biased towards unmappable regions because they are composed of repetitive sequences that are likely to recombine.

Variability among tumor-types of genomic features predicting breakpoint density

Although we could predict much of the variability in breakpoint density using these genomic features, breakpoint densities in sarcomas, cervical cancer, and prostate cancer were substantially less predictable than in other tumors, particularly adenocarcinomas. This suggests their breakpoints are generated by mechanisms that are governed either by stochasticity or genomic features that we have not yet considered. These tumor types tend to undergo singular catastrophic rearrangements: prostate tumors through chains of deletions in chromoplexy[42], and sarcomas through high rates of chromothripsis[43,44].

To quantify the extent to which different tumor types have shared mechanisms of breakpoint formation, we clustered the tumor types by their correlations with genomic features (using the *hclust* R package; **Supplementary Fig. 8c**). Tumors from similar tissues and cell types tended to cluster together (e.g., esophageal and stomach adenocarcinomas). Breast tumors from PCAWG closely clustered with an independent cohort of breast tumors from Nik-Zainal et al[11], supporting the robustness of our approach across different patients and rearrangement analysis pipelines. Interestingly, ovarian and glioblastoma tumors were closely clustered, as were lung and pancreatic adenocarcinomas, suggesting similar rearrangement mechanisms despite different cell types and tumor microenvironments.

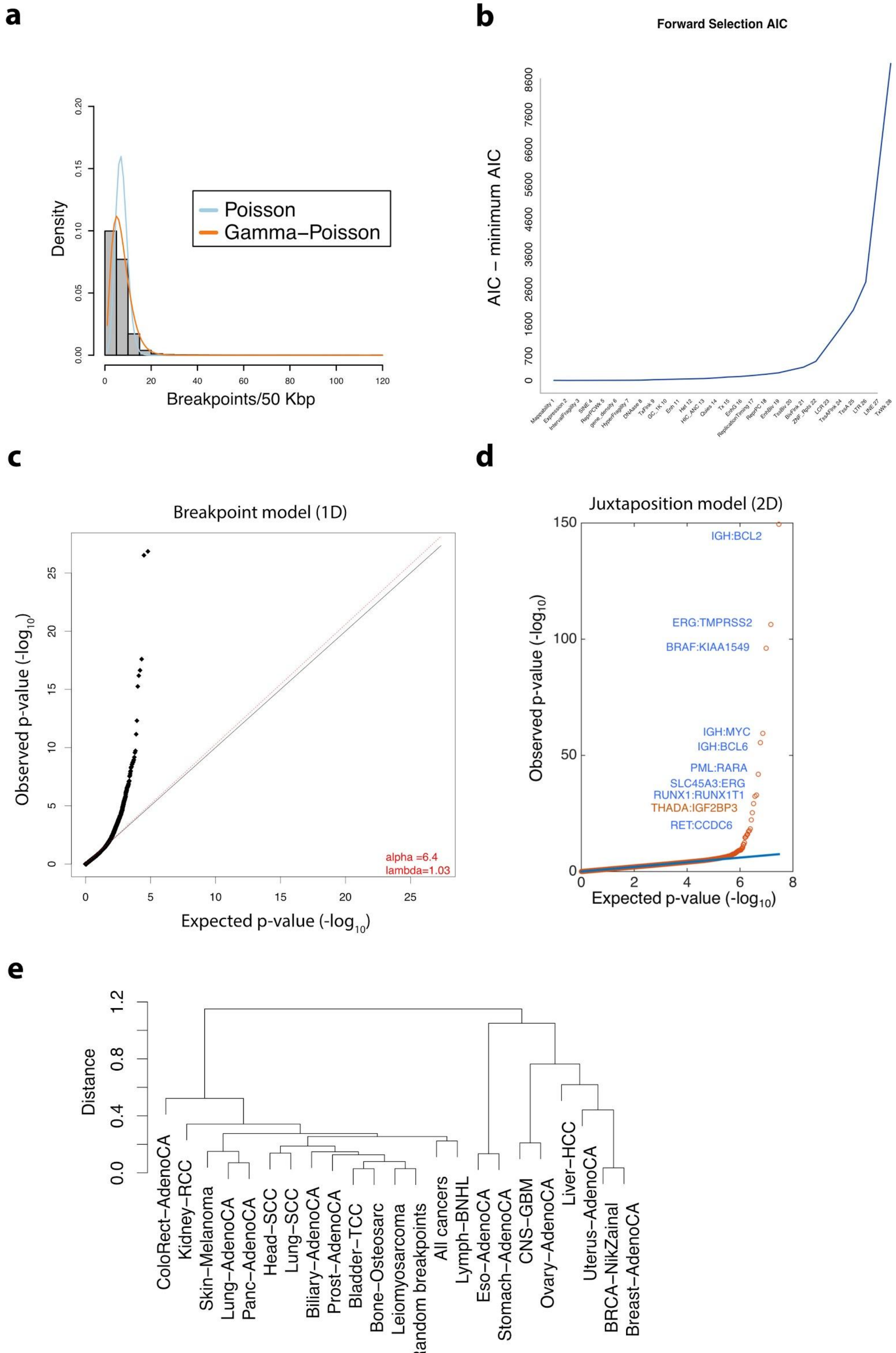

a
Density
Poisson
Gamma−Poisson
Breakpoints/50 Kbp
b
Forward Selection AIC
AIC − minimum AIC
c
Breakpoint model (1D)
Observed p-value (-log10)
Expected p-value (-log10)
alpha =6.4
lambda=1.03
d
Juxtaposition model (2D)
IGH:BCL2
ERG:TMPRSS2
BRAF:KIAA1549
IGH:MYC
IGH:BCL6
PML:RARA
SLC45A3:ERG
RUNX1:RUNX1T1
THADA:IGF2BP3
RET:CCDC6
Observed p-value (-log10)
Expected p-value (-log10)
e
Distance
ColoRect−AdenoCA
Kidney−RCC
Skin−Melanoma
Lung−AdenoCA
Panc−AdenoCA
Head−SCC
Lung−SCC
Biliary−AdenoCA
Prost−AdenoCA
Bladder−TCC
Bone−Osteosarc
Leiomyosarcoma
Random breakpoints
All cancers
Lymph−BNHL
Eso−AdenoCA
Stomach−AdenoCA
CNS−GBM
Ovary−AdenoCA
Liver−HCC
Uterus−AdenoCA
BRCA−NikZainal
Breast−AdenoCA

Supplementary Figure 8: a, Histogram of breakpoint counts per 50 Kbp bin (with only one sample-breakpoint per donor per bin). The distribution more closely follows a Gamma-Poisson (orange) than a Poisson (blue). **b,** Standardized Akaike Information Criteria (AIC) of the best model for each number of covariates. The best model was chosen to have seven covariates based on cutoffs cited in Burnham & Anderson[41]. **c,** Quantile–quantile (QQ) plot for the 61,920 bins in the 1D model. The overdispersion (alpha) parameter is 6.4 for the Gamma-Poisson regression. The slope of the QQ plot (lambda) is slightly above 1. Most bins are not undergoing positive selection, while the remaining bins that are positively selected lie to the left of the y = x line. These fit parameters indicate a good model fit. **d,** QQ plot for the $3x10^7$ tiles in the 2D model. **e,** Clustering of tumor-types by the genomic feature effect sizes from the 1D Gamma-Poisson model.

7. Distinguishing fragile sites from positive selection in regions of recurrent breakpoints

Among SCNAs, a major difficulty has been distinguishing recurrent alterations that are primarily driven by genome fragility from those resulting from positive selection, which has particularly hampered the detection of deleted tumor suppressor genes[45]. Further, common fragile sites are defined by aphidicolin break experiments, and are non-specific and poorly predictive of somatic rearrangements. For instance, 17% of the non-centromeric genome is covered by a fragile site, including known cancer genes like *BCL2* (FRA18B). Late-replicating regions have been proposed as fragile sites, and SCNA breakpoints have been found to be enriched in late-replicating regions[46]. However, SCNA breakpoints are imprecise, and rearrangements encompass a potentially larger set of breakpoints than SCNAs. Whether replication timing alone can robustly distinguish fragile sites in regions of recurrent rearrangements is unknown.

We enquired whether the precise breakpoint locations of the rearrangements that generate frequently rearranged regions might make the distinction between fragility and positive selection clearer. Among the 53 RSBs, 8 overlapped common fragile sites from aphidicolin break experiments. Among these, two were clearly driven by selection rather than fragility (*BCL2* and *MIR21*), and one was part of a large and non-specific fragile site (20 Mbp). The remaining five RSBs had markedly late-replication timing (mean: -0.76). We further noted that 13 of the 15 most frequently rearranged genes in the genome had a replication timing later than -0.5; notably, although many of these were adjacent to known common fragile sites, only 3 overlapped with one. Twelve RSBs also had replication times later than -0.5, including nine that have not been identified by aphidicolin break-induction experiments[47]. Although eight of these 12 RSBs had previously been described as recurrently deleted, none involve known oncogenes or tumor suppressor genes. Conversely, 26 of the other 41 RSBs involve known oncogenes or tumor suppressor genes. We therefore considered all of these late-replicating RSBs to reflect DNA fragility rather than positive selection.

8. Comparison of known SCNAs and fusions with recurrent breakpoints and rearrangements

We compared the recurrent breakpoints (RSBs) and recurrent juxtapositions (RSJ) detected in our 1D and 2D models with databases of known SCNAs and fusions. We defined known SCNAs as those that overlap with peak regions of recurrent focal amplifications or deletions in GISTIC pan-cancer analyses of 10,844 TCGA samples, as indicated in the TCGA copy number portal (www.broadinstitute.org/tcga). In total, 19 of 53 RSBs are novel, including 1 deletion, 3 amplifications, 4 fragile sites, and 11 neutral rearrangements. These results are available in **Supplementary Table 14**.

For recurrent juxtapositions, we used the COSMIC[48] fusions list (v87; cancer.sanger.ac.uk) to annotate known fusions. We only considered fusions that appear in more than one sample in COSMIC. To supplement the COSMIC list, we also searched for papers describing the RSJs in PubMed. For each RSJ, we queried both affected genes along with the terms “cancer” and “fusion”. Among the 90 RSJs, we found 77 to be novel. These results are available in **Supplementary Table 15**.

To further evaluate the applicability of our statistical methods and power calculations to the detection of RSJs, we evaluated how well our model performed at detecting known fusions from the curated COSMIC fusion list. From 167 COSMIC fusions represented in > 1 non-review article and > 1 sample, we identified 23 COSMIC fusions with at least one rearrangement bridging the two genes to within 100 Kbp of each gene (note that the majority of COSMIC fusions are reported in rare tumors not included in PCAWG). Of these 23 fusions, 10 were detected as an RSJ, and 7 were present in a single sample and thus could not be detected as recurrent. Of the remaining fusions, 5 had spans < 100 Kbp and fell below our power to detect. On further inspection, of the 39 samples that harbored these 5 fusions, only two (*FGFR3–TACC3* rearrangements in bladder cancer, neither supported by fusion transcripts) were consistent with the previously reported tumor type. Rather, the observed counts are consistent with the expected number of background rearrangements between neighboring genes separated by < 50 Kbp. The only bonafide oncogenic fusion our statistical methods missed was an in-frame *ERC1–RET* fusion in two thyroid adenocarcinoma samples (4% prevalence in thyroid adenocarcinoma, 0.1% overall prevalence). However, our models accurately detected four other bonafide rearrangements with tumor-type specific prevalences < 5% in our cohort (*QKI–NTRK2*, *TMPRSS2–ETV1*, *RNF130–BRAF*,

MYH11–CBFB), indicating that our overall false-negative rate is consistent with our power calculations for very low prevalence rearrangements. Thus, we find that accounting for statistical power to detect recurrent rearrangements avoids the pitfall of attributing selection to low-frequency rearrangements that could be more parsimoniously explained by background noise.

In the comparisons between the ten most significant SRJs and the ten most significant SNVs and SCNAs, we used SNV and SCNA lists from www.tumorportal.org and www.broadinstitute.org/tcga, respectively.

9. Amplification structure of the *BRD4/NOTCH3* locus in the context of recurrent *BRD4* deletion

The broad amplifications involving the *BRD4* and *NOTCH3* locus, in cases with a focal *BRD4* deletion, were typically tens of Mbps in length, sometimes involving the entire chromosome (**Extended Data Fig. 8f**). The *BRD4*/*NOTCH3* locus is in a significant amplification peak (GISTIC analysis) in breast and ovarian cancers. The only other recurrent amplification on chromosome 19 in breast and ovarian cancers is *CCNE1* (chr19: 30.3 Mbp). Indeed, many of these amplifications involved both loci, and more samples would be required to determine the exact amplification target. Regardless, the finding that these focal deletions tend to occur concomitantly with amplifications implies that amplification of the *NOTCH3*/*BRD4* region changes the selective landscape to favor *BRD4* intron 1 deletions.

10. Effect of element length on power calculations

The number of patients that need to be sequenced to achieve a certain power to discover a recurrently mutated driver with specific mutation abundance in the cohort also depends on the length of the analyzed elements. Prior power calculations have focused on fixed mean element lengths for either coding[49] or promoter regions[49,50]. We here add power calculations for different driver abundances and different element lengths of the range representative of coding and non-coding functional regions (**Supplementary Fig. 9).**

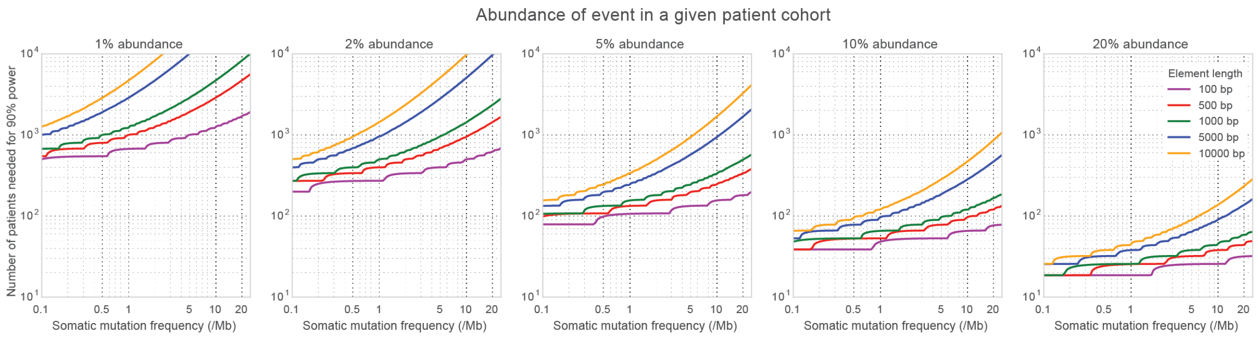

Supplementary Figure 9: Changes in discovery power depending on element length for different abundances.

We further note that element length L and background mutation frequency f are mathematically related in the power calculation, such that the number of patients required for a new length L_2 can be obtained from a power plot using element length L_1 (for example, from Lawrence et al, 2014). This can be obtained through the following relationship between the two background mutation frequencies f_1 and f_2 as follows:

$$f_2 = 1 - (1 - f_1)^{L_1/L_2}$$

The number of patients required for length L_2 given a fixed driver abundance in the population can then be read off the plot at the x-axis position f_2, representing an adjustment of the background mutation rate to achieve the same power as with element length L_1 and background mutation frequency f_1.

For example for $L_1 = 500\ bp$, $f_1 = 5 * 10^{-6}$ mut/bp and new element length $L_2 = 5000\ bp$, $f_2 = 1 - (1 - f_1)^{L_1/L_2} = 1 - (1 - 5 * 10^{-6})^{(500/5000)} = 5 * 10^{-7} = 0.5 * 10^{-6}$mut/bp.

In **Supplementary Fig. 10**, we can see that the number of patients required for 90% power to detect an element with 5% frequency in a patient cohort is equivalent for elements of length 500 bp (red line) and background mutation frequency $5 * 10^{-6}$mut/bp and elements of length 5000 bp (blue line) and background mutation frequency $0.5 * 10^{-6}$mut/bp, as calculated above.

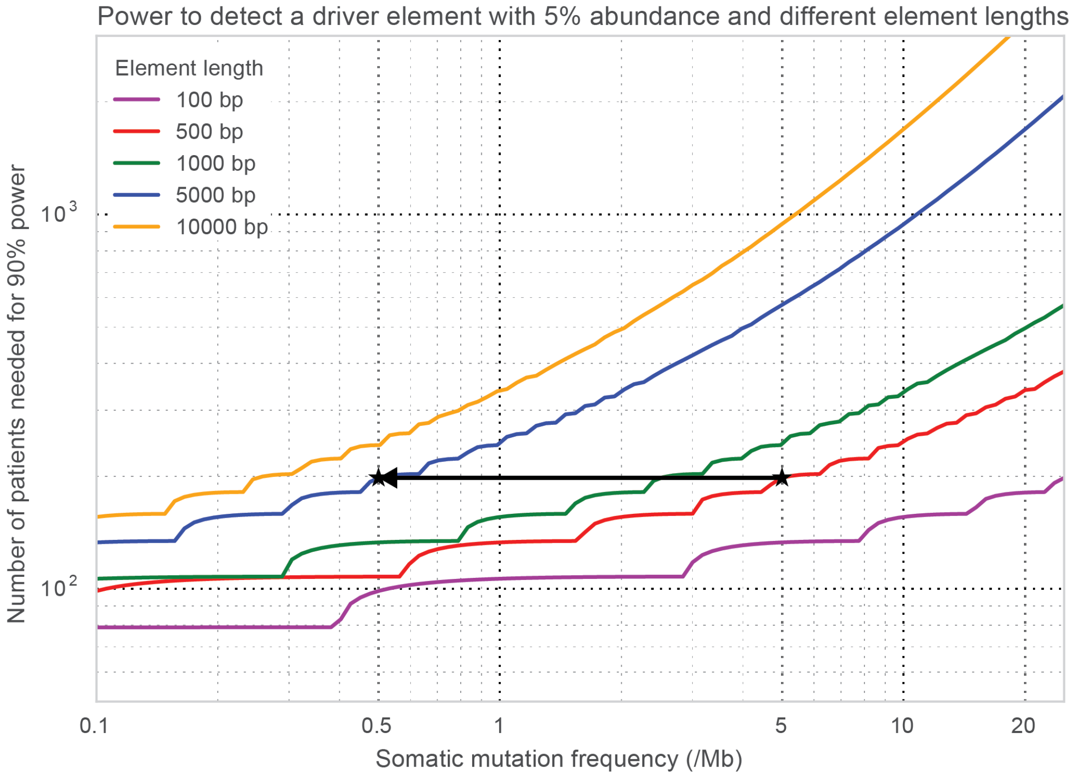

Supplementary Figure 10: Background mutation frequency and element length are mathematically related in the power equation. For a power calculation with fixed driver abundance in the population, one can calculate the position on the power graph for length L_1 for a new length L_2.

11. Impact of covariates on the estimation of driver mutations in functional regions of cancer genes

As described in **Methods**, we estimated the abundance of driver mutations in coding and regulatory regions (promoter and UTRs) of 603 known cancer genes using the NBR background model fitted on putative passenger genes.

Promoters and 5'UTR regions are particularly sensitive to GC-sequencing biases, resulting in overall lower detection sensitivities. To alleviate these biases, we selected a set of samples with good d.s. by requiring each sample to have sensitivity > 90% in the two main *TERT* promoter hotspots. After this selection, despite some outliers, average sensitivity was much higher. We compared detection sensitivity in putative passenger genes and the 603 cancer genes to search for potential systematic biases that may compromise the analysis. Although there are some differences between passengers and drivers, these are small, and overall detection sensitivity values are high (**Extended Data Fig. 10e**).

Different covariates were included to improve the fit of the model. The local mutation rate, calculated on neutral regions within +/-100 kb around each element, was included to account for regional variation of mutation rates. A second type of covariate was included in the model to account for associations between gene expression levels and mutation rates. Starting with a matrix of mean FPKM expression values for each gene and tumor type, we log-transformed and scaled the expression matrix using pseudocounts and applied Principal Component Analysis to reduce the dimensionality. We selected the first 8 components as covariates, which together explained 95.5% of the variance. In addition, we added two additional covariates to account for non-linearity between expression and mutation rate in the tails of the expression spectrum. To accomplish this, we created two binary variables, one marking the 500 genes with highest maximum expression values across tumor types, the other marking 1,229 genes whose expression did not exceed FPKM values of 0.1 in any tumor type. Finally, since tumors are rich in amplifications and deletions and these events may result in seemingly increased or decreased mutation rates, we included a copy-number covariate, calculated as the average copy number of each gene across all PCAWG samples.

Supplementary Table 8 shows the impact of using different covariates on the 603 genes selected for this analysis. Reassuringly, this shows that the estimates are broadly consistent across models with different covariates, with variations typically within the confidence intervals

of alternative models. This confirms that the overall conclusions are largely unaffected by the use of different models. **Supplementary Fig. 11** shows the results of this analysis, including the observed-to-expected ratios, the percentage of mutations predicted to be drivers, and the estimated numbers of driver mutations.

To evaluate the performance of the NBR model, we compared the number of driver substitutions predicted by NBR in the CDS regions of the 603 cancer genes to the number predicted by dN/dS (calculated by dNdScv). dN/dS offers an independent estimate of the number of driver substitutions in a group of genes using the local density of synonymous mutations to estimate the neutral expectation[51], instead of predicting the background mutation rate by extrapolation from putative passenger genes using a regression model. Reassuringly, in these 603 genes, NBR predicts 1,118 ($CI_{95\%}$: 991–1,248) driver substitutions, and dN/dS predicts 1,176 ($CI_{95\%}$: 914–1,460).

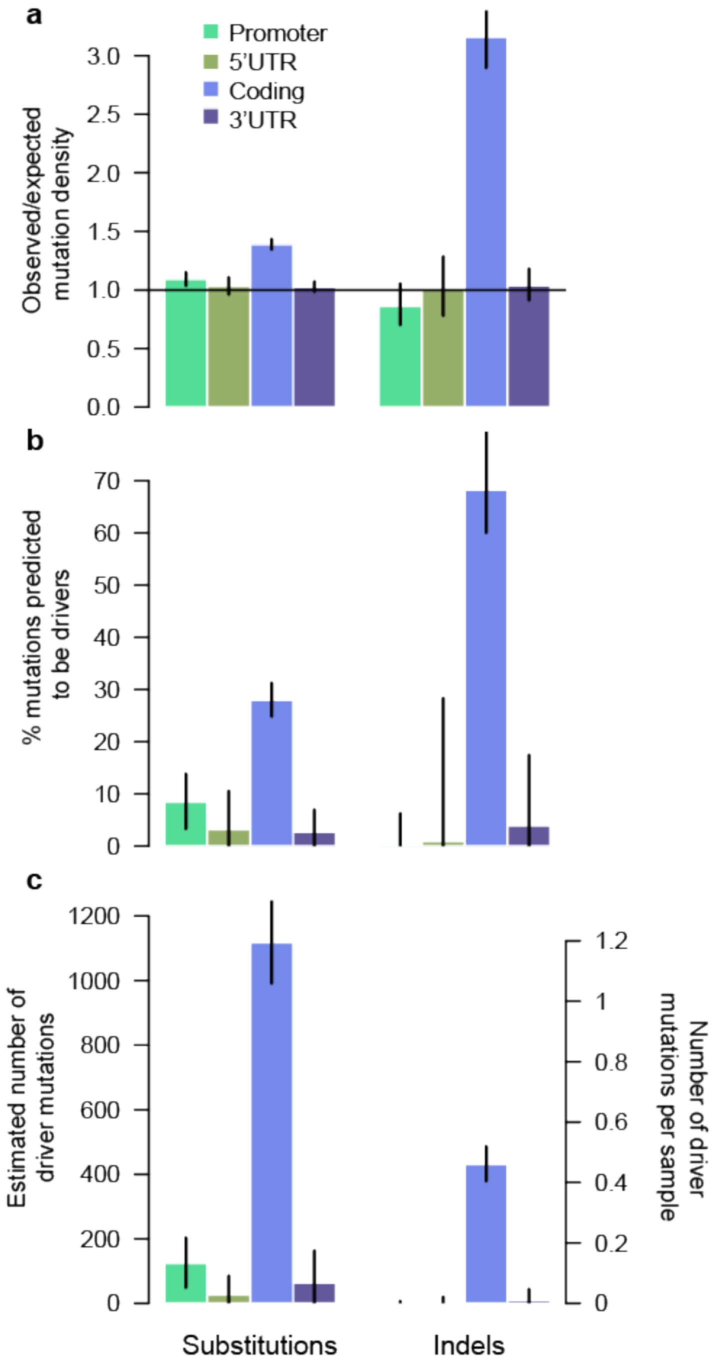

Supplementary Figure 11: Estimation of the excess substitutions (left) and indels (right) in regulatory and protein-coding regions of 603 known cancer genes. **a**, Ratios of observed vs. expected number of mutations; **b**, the percentage of mutations predicted to be drivers; **c**, and the total number of predicted drivers in all cancers and in each patient. **a-c,** Black bars are Binomial 95% confidence intervals; only samples with high detection sensitivity from the Pancancer cohort excluding Skin-Melanoma and lymphoid malignancies included (n = 936).

12. Relative paucity of regulatory non-coding drivers at cancer genes

Collectively, mutations occurring in the promoter region of the 757 cancer genes (603 CGC genes combined with 157 genes recurrently mutated in exome studies; **Methods**) did not have a significantly different association with expression than synonymous mutations (**Supplementary Fig. 12a**). Similarly, promoter and UTR mutations in cancer genes are not significantly enriched in LOH with respect to mutations in putative passenger genes (**Methods**; **Supplementary Fig. 12b**). This is consistent with the prediction that only a very small fraction of the mutations observed in the promoters and UTRs of known cancer genes are genuine driver events.

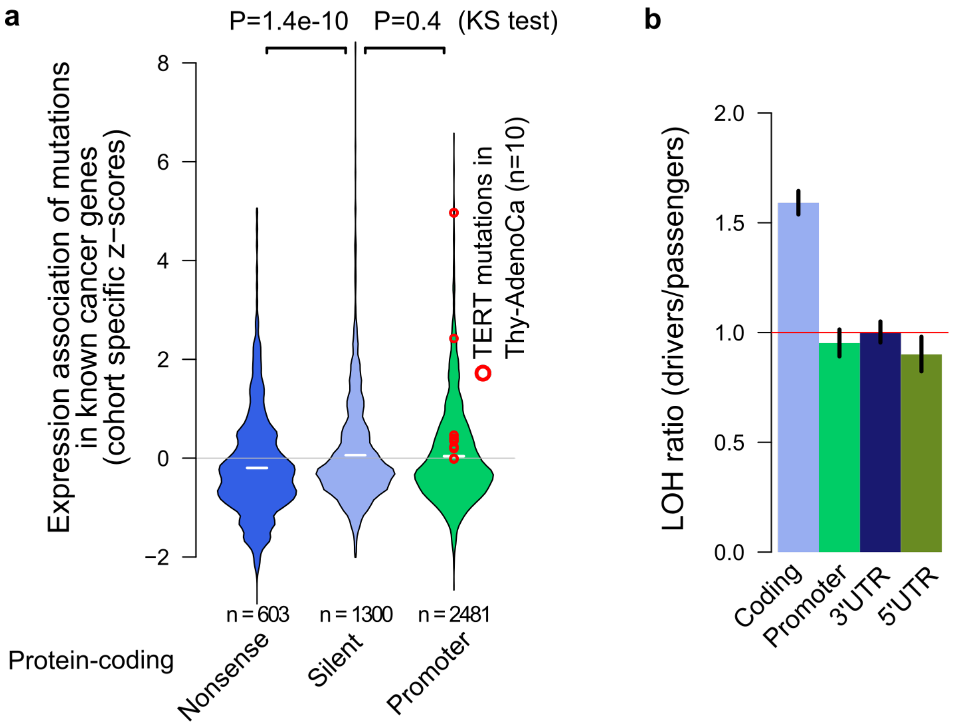

Supplementary Figure 12: a, Expression associated with mutations in coding and promoter regions of cancer genes. Z-score expressions associated with nonsense mutations deviate significantly from silent mutations, likely through nonsense mediated decay, whereas expressions associated with promoter mutations do not differ from that of silent mutations. Only mutations in diploid positions were used. White bars indicate means. Two-sided Kolmogorov–Smirnov (KS) test was used to evaluate *P* values. **b,** Excess of LOH associated to mutation in regulatory and coding regions of cancer genes. The y-axis shows the ratio of fold changes in cancer vs. passenger genes, with fold changes representing the excess or depletion of LOH associated with mutation. The analysis is performed across the full set of PCAWG samples (n = 2,583) and the set of 757 cancer genes (**Supplementary Table 7**), using 19,107 genes as

putative passengers or controls (**Methods**). Confidence intervals were estimated using parametric bootstrapping (100,000 pseudoreplicates) for both cancer and passenger genes (**Methods**). Details available in **Supplementary Table 19**.

13. References

1. Buisson, R. *et al.* Passenger hotspot mutations in cancer driven by APOBEC3A and mesoscale genomic features. *Science* **364**, (2019).
2. Alexandrov, L. B. *et al.* Signatures of mutational processes in human cancer. *Nature* **500**, 415–421 (2013).
3. Katainen, R. *et al.* CTCF/cohesin-binding sites are frequently mutated in cancer. *Nat. Genet.* **47**, 818–821 (2015).
4. Hornshøj, H. *et al.* Pan-cancer screen for mutations in non-coding elements with conservation and cancer specificity reveals correlations with expression and survival. *NPJ Genom Med* **3**, 1 (2018).
5. Jiang, L. *et al.* Discrete False-Discovery Rate Improves Identification of Differentially Abundant Microbes. *mSystems* **2**, (2017).
6. Yekutieli, D. & Benjamini, Y. under dependency. *The Annals of Statistics* **29**, 1165–1188 (2001).
7. Khodabakhshi, A. H. *et al.* Recurrent targets of aberrant somatic hypermutation in lymphoma. *Oncotarget* **3**, 1308–1319 (2012).
8. Futreal, P. A. *et al.* A census of human cancer genes. *Nat. Rev. Cancer* **4**, 177–183 (2004).
9. Hu, J., Adebali, O., Adar, S. & Sancar, A. Dynamic maps of UV damage formation and repair for the human genome. *Proc. Natl. Acad. Sci. U. S. A.* **114**, 6758–6763 (2017).
10. Sabarinathan, R., Mularoni, L., Deu-Pons, J., Gonzalez-Perez, A. & López-Bigas, N. Nucleotide excision repair is impaired by binding of transcription factors to DNA. *Nature* **532**, 264–267 (2016).
11. Nik-Zainal, S. *et al.* Landscape of somatic mutations in 560 breast cancer whole-genome sequences. *Nature* **534**, 47–54 (2016).

12. Weinhold, N., Jacobsen, A., Schultz, N., Sander, C. & Lee, W. Genome-wide analysis of noncoding regulatory mutations in cancer. *Nat. Genet.* **46**, 1160–1165 (2014).

13. Khurana, E. *et al.* Integrative annotation of variants from 1092 humans: application to cancer genomics. *Science* **342**, 1235587 (2013).

14. Fujimoto, A. *et al.* Whole-genome mutational landscape and characterization of noncoding and structural mutations in liver cancer. *Nat. Genet.* **48**, 500–509 (2016).

15. Kopan, R. & Ilagan, M. X. G. The canonical Notch signaling pathway: unfolding the activation mechanism. *Cell* **137**, 216–233 (2009).

16. Diaz-Lagares, A. *et al.* Epigenetic inactivation of the p53-induced long noncoding RNA TP53 target 1 in human cancer. *Proc. Natl. Acad. Sci. U. S. A.* **113**, E7535–E7544 (2016).

17. ENCODE Project Consortium. An integrated encyclopedia of DNA elements in the human genome. *Nature* **489**, 57–74 (2012).

18. Dulak, A. M. *et al.* Exome and whole-genome sequencing of esophageal adenocarcinoma identifies recurrent driver events and mutational complexity. *Nat. Genet.* **45**, 478–486 (2013).

19. Shadel, G. S. & Clayton, D. A. Mitochondrial DNA maintenance in vertebrates. *Annu. Rev. Biochem.* **66**, 409–435 (1997).

20. Schmitt, M. E. & Clayton, D. A. Nuclear RNase MRP is required for correct processing of pre-5.8S rRNA in Saccharomyces cerevisiae. *Mol. Cell. Biol.* **13**, 7935–7941 (1993).

21. Gill, T., Cai, T., Aulds, J., Wierzbicki, S. & Schmitt, M. E. RNase MRP cleaves the CLB2 mRNA to promote cell cycle progression: novel method of mRNA degradation. *Mol. Cell. Biol.* **24**, 945–953 (2004).

22. Esakova, O. & Krasilnikov, A. S. Of proteins and RNA: the RNase P/MRP family. *RNA* **16**, 1725–1747 (2010).

23. Baer M, E. al. Structure and transcription of a human gene for H1 RNA, the RNA component of human RNase P. - PubMed - NCBI. Available at: https://www.ncbi.nlm.nih.gov/pubmed/2308839. (Accessed: 7th July 2017)

24. Iyer, M. K. *et al.* The landscape of long noncoding RNAs in the human transcriptome. *Nat. Genet.* **47**, 199–208 (2015).

25. Yi, C. *et al.* MiR-663, a microRNA targeting p21(WAF1/CIP1), promotes the proliferation and tumorigenesis of nasopharyngeal carcinoma. *Oncogene* **31**, 4421–4433 (2012).

26. Christov, C. P., Gardiner, T. J., Szüts, D. & Krude, T. Functional requirement of noncoding Y RNAs for human chromosomal DNA replication. *Mol. Cell. Biol.* **26**, 6993–7004 (2006).

27. Christov, C. P., Trivier, E. & Krude, T. Noncoding human Y RNAs are overexpressed in tumours and required for cell proliferation. *Br. J. Cancer* **98**, 981–988 (2008).

28. Chang, J. *et al.* miR-122, a mammalian liver-specific microRNA, is processed from hcr mRNA and may downregulate the high affinity cationic amino acid transporter CAT-1. *RNA Biol.* **1**, 106–113 (2004).

29. Lagos-Quintana, M. *et al.* Identification of tissue-specific microRNAs from mouse. *Curr. Biol.* **12**, 735–739 (2002).

30. Nielsen, M. M., Tataru, P., Madsen, T., Hobolth, A. & Pedersen, J. S. Regmex: a statistical tool for exploring motifs in ranked sequence lists from genomics experiments. *Algorithms Mol. Biol.* **13**, 17 (2018).

31. Tomasetti, C., Marchionni, L., Nowak, M. A., Parmigiani, G. & Vogelstein, B. Only three driver gene mutations are required for the development of lung and colorectal cancers. *Proc. Natl. Acad. Sci. U. S. A.* **112**, 118–123 (2015).

32. Lawrence, M. S. *et al.* Mutational heterogeneity in cancer and the search for new

cancer-associated genes. *Nature* **499**, 214–218 (2014).

33. McFarland, C. D. *et al.* The Damaging Effect of Passenger Mutations on Cancer Progression. *Cancer Res.* **77**, 4763–4772 (2017).

34. Martincorena, I. *et al.* Universal Patterns of Selection in Cancer and Somatic Tissues. *Cell* **173**, 1823 (2018).

35. Korn, J. M. *et al.* Integrated genotype calling and association analysis of SNPs, common copy number polymorphisms and rare CNVs. *Nat. Genet.* **40**, 1253–1260 (2008).

36. Hodis, E. *et al.* A landscape of driver mutations in melanoma. *Cell* **150**, 251–263 (2012).

37. Fu, Y. *et al.* FunSeq2: a framework for prioritizing noncoding regulatory variants in cancer. *Genome Biol.* **15**, 480 (2014).

38. Dees, N. D. *et al.* MuSiC: Identifying mutational significance in cancer genomes. *Genome Res.* **22**, 1589–1598 (2012).

39. Zack, T. I. *et al.* Pan-cancer patterns of somatic copy number alteration. *Nat. Genet.* **45**, 1134–1140 (2013).

40. Beroukhim, R. *et al.* The landscape of somatic copy-number alteration across human cancers. *Nature* **463**, 899–905 (2010).

41. Burnham, K. P. & Anderson, D. R. Multimodel Inference: Understanding AIC and BIC in Model Selection. *Sociol. Methods Res.* **33**, 261–304 (2004).

42. Baca, S. C. *et al.* Punctuated Evolution of Prostate Cancer Genomes. *Cell* **153**, 666–677 (2013).

43. Mehine, M. *et al.* Characterization of uterine leiomyomas by whole-genome sequencing. *N. Engl. J. Med.* **369**, 43–53 (2013).

44. Stephens, P. J. *et al.* Massive Genomic Rearrangement Acquired in a Single Catastrophic Event during Cancer Development. *Cell* **144**, 27–40 (2011).

45. Bignell, G. R. *et al.* Signatures of mutation and selection in the cancer genome.

Nature **463**, 893–898 (2010).

46. De, S. & Michor, F. DNA secondary structures and epigenetic determinants of cancer genome evolution. *Nat. Struct. Mol. Biol.* **18**, 950–955 (2011).

47. Mrasek, K. *et al.* Global screening and extended nomenclature for 230 aphidicolin-inducible fragile sites, including 61 yet unreported ones. *Int. J. Oncol.* **36**, 929–940 (2010).

48. Forbes, S. A. *et al.* COSMIC: somatic cancer genetics at high-resolution. *Nucleic Acids Res.* **45**, D777–D783 (2017).

49. Lawrence, M. S. *et al.* Discovery and saturation analysis of cancer genes across 21 tumour types. *Nature* **505**, 495–501 (2014).

50. Rheinbay, E. *et al.* Recurrent and functional regulatory mutations in breast cancer. *Nature* **547**, 55–60 (2017).

51. Martincorena, I. *et al.* Universal Patterns of Selection in Cancer and Somatic Tissues. *Cell* **171**, 1029–1041.e21 (2017).

14. Participants in PCAWG Consortium

Steering committee

Peter J Campbell#[1,2], **Gad Getz#**[3,4,5,6], **Jan O Korbel#**[7,8], **Lincoln D Stein#**[9,10] and **Joshua M Stuart#**[11]

Executive committee

Sultan T Al-Sedairy[12], Axel Aretz[13], Cindy Bell[14], Miguel Betancourt[15], Christiane Buchholz[16], Fabien Calvo[17], Christine Chomienne[18], Michael Dunn[19], Stuart Edmonds[20], Eric Green[21], Shailja Gupta[22], Carolyn M Hutter[21], Karine Jegalian[23], Jennifer L Jennings[24,25], Nic Jones[26], Hyung-Lae Kim[27], Youyong Lu[28,29,30], Hitoshi Nakagama[31], Gerd Nettekoven[32], Laura Planko[32], David Scott[26], Tatsuhiro Shibata[33,34], Kiyo Shimizu[35], Lincoln D Stein[9,10], Michael Rudolf Stratton[1], Takashi Yugawa[36], Giampaolo Tortora[37,38], K VijayRaghavan[22], Huanming Yang[39] and Jean C Zenklusen[40]

Ethics and legal working group

Don Chalmers#[41], Yann Joly[42], **Bartha M Knoppers#**[42], Fruzsina Molnár-Gábor[43], Mark Phillips[42], Adrian Thorogood[42] and David Townend[44]

Technical working group

Brice Aminou[45], Javier Bartolome[46], Keith A Boroevich[47,48], Rich Boyce[7], Alvis Brazma[7], Angela N Brooks[3,49,50], Alex Buchanan[51], Ivo Buchhalter[52,53,54], Adam P Butler[1], Niall J Byrne[45], Andy Cafferkey[7], Peter J Campbell[1,2], Zhaohong Chen[55], Sunghoon Cho[56], Wan Choi[57], Peter Clapham[1], Brandi N Davis-Dusenbery[58], Francisco M De La Vega[59,60,61], Jonas Demeulemeester[63,64], Michelle T Dow[55], Lewis Jonathan Dursi[9,65], Juergen Eils[66,67], Roland Eils[52,54,66,67], Kyle Ellrott[51], Claudiu Farcas[55], Nodirjon Fayzullaev[45], Vincent Ferretti[45,68], Paul Flicek[7], Nuno A Fonseca[7,69], Josep Ll Gelpi[46,70], Gad Getz[3,4,5,6], Bob Gibson[45], Robert L Grossman[71], Olivier Harismendy[72], Allison P Heath[73], Michael C Heinold[52,54], Julian M Hess[3,74], Oliver Hofmann[75], Jongwhi H Hong[76], Thomas J Hudson[77,78], Barbara Hutter[79,80,81], Carolyn M Hutter[21], Daniel Hübschmann[54,66,82,83,84], Seiya Imoto[85,86], Sinisa Ivkovic[87], Seung-Hyup Jeon[57], Wei Jiao[9], Jongsun Jung[88], Rolf Kabbe[52], Andre Kahles[89,90,91,92,93], Jules NA Kerssemakers[52], Hyung-Lae Kim[27], Hyunghwan Kim[57], Jihoon Kim[94], Youngwook Kim[95,96], Kortine Kleinheinz[52,54], Jan O Korbel[7,8], Michael Koscher[97], Antonios Koures[55], Milena Kovacevic[87], Chris Lawerenz[67], Ignaty Leshchiner[3], Jia Liu[98], Dimitri Livitz[3], George L Mihaiescu[45], Sanja Mijalkovic[87], Ana Mijalkovic Mijalkovic-Lazic[87], Satoru Miyano[86], Naoki Miyoshi[86], Hardeep K Nahal-Bose[45], Hidewaki Nakagawa[48], Mia Nastic[87], Steven J Newhouse[7], Jonathan Nicholson[1], **Brian D O'Connor#**[45,50], David Ocana[7], Kazuhiro Ohi[85], Lucila Ohno-Machado[55], Larsson Omberg[99], BF Francis Ouellette[100,101], Nagarajan Paramasivam[52,80], Marc D Perry[45,102], Todd D Pihl[103], Manuel

Prinz[52], Montserrat Puiggròs[46], Petar Radovic[87], Keiran M Raine[1], Esther Rheinbay[3,6,104], Mara Rosenberg[3,104], Romina Royo[46], Gunnar Rätsch[89,92,105,106,107,108], Gordon Saksena[3], Matthias Schlesner[52,109], Solomon I Shorser[9], Charles Short[7], Heidi J Sofia[21], Jonathan Spring[71], **Lincoln D Stein#**[9,10], Adam J Struck[51], Grace Tiao[3], Nebojsa Tijanic[87], David Torrents[46,110], Peter Van Loo[63,64], Miguel Vazquez[46,111], David Vicente[46], Jeremiah A Wala[3,6,49], Zhining Wang[40], Sebastian M Waszak[8], Joachim Weischenfeldt[8,112,113], Johannes Werner[52,114], Ashley Williams[55], Youngchoon Woo[57], Adam J Wright[9], Qian Xiang[115], **Sergei Yakneen#**[8], Liming Yang[40], Denis Yuen[9], **Christina K Yung#**[45] and **Junjun Zhang#**[45]

Annotations working group

Angela N Brooks[3,49,50], Ivo Buchhalter[52,53,54], Peter J Campbell[1,2], Priyanka Dhingra[116,117], Lars Feuerbach[118], Mark Gerstein[119,120,121], Gad Getz[3,4,5,6], Mark P Hamilton[122], Henrik Hornshøj[123], Todd A Johnson[47], Andre Kahles[89,90,91,92,93], Abdullah Kahraman[124,125,126], Manolis Kellis[3,127], **Ekta Khurana#**[116,117,128,129], Jan O Korbel[7,8], Morten Muhlig Nielsen[123], Jakob Skou Pedersen[123,130], Paz Polak[3,4,6], Jüri Reimand[9,131], Esther Rheinbay[3,6,104], Nicola D Roberts[1], Gunnar Rätsch[89,92,105,106,107,108], Richard Sallari[3], Nasa Sinnott-Armstrong[3,61], Alfonso Valencia[46,110], Miguel Vazquez[46,111], Sebastian M Waszak[8], Joachim Weischenfeldt[8,112,113] and Christian von Mering[126,132]

Quality control working group

Sergi Beltran[133,134], Ivo Buchhalter[52,53,54], Peter J Campbell[1,2], Roland Eils[52,54,66,67], Daniela S Gerhard[135], Gad Getz[3,4,5,6], **Ivo G Gut#**[133,134], Marta Gut[133,134], Barbara Hutter[79,80,81], Daniel Hübschmann[54,66,82,83,84], Kortine Kleinheinz[52,54], Jan O Korbel[7,8], Dimitri Livitz[3], Marc D Perry[45,102], Keiran M Raine[1], Esther Rheinbay[3,6,104], Mara Rosenberg[3,104], Gordon Saksena[3], Matthias Schlesner[52,109], Miranda D Stobbe[133,134], Jean-Rémi Trotta[133], Johannes Werner[52,114] and Justin P Whalley[133]

Novel somatic mutation calling methods

Matthew H Bailey[136,137], Beifang Niu[138], Matthias Bieg[80,139], Paul C Boutros[9,131,140,141], Ivo Buchhalter[52,53,54], Adam P Butler[1], Ken Chen[142], Zechen Chong[143], **Li Ding#**[136,137,144], Oliver Drechsel[134,145], Lewis Jonathan Dursi[9,65], Roland Eils[52,54,66,67], Kyle Ellrott[51], Shadrielle MG Espiritu[9], Yu Fan[146], Robert S Fulton[136,137,144], Shengjie Gao[147], Josep Ll Gelpi[46,70], Mark Gerstein[119,120,121], Gad Getz[3,4,5,6], Santiago Gonzalez[7,8], Ivo G Gut[133,134], Faraz Hach[148,149], Michael C Heinold[52,54], Julian M Hess[3,74], Jonathan Hinton[1], Taobo Hu[150], Vincent Huang[9], Yi Huang[151,152], Barbara Hutter[79,80,81], David R Jones[1], Jongsun Jung[88], Natalie Jäger[52], Hyung-Lae Kim[27], Kortine Kleinheinz[52,54], Sushant Kumar[120,121], Yogesh Kumar[150], Christopher M Lalansingh[9], Ignaty Leshchiner[3], Ivica Letunic[153], Dimitri Livitz[3], Eric Z Ma[150], Yosef E Maruvka[3,74,104], R Jay Mashl[137,154], Michael D McLellan[136,137,144], Andrew Menzies[1], Ana Milovanovic[46], Morten Muhlig

Nielsen[123], Stephan Ossowski[134,145,155], Nagarajan Paramasivam[52,80], Jakob Skou Pedersen[123,130], Marc D Perry[45,102], Montserrat Puiggròs[46], Keiran M Raine[1], Esther Rheinbay[3,6,104], Romina Royo[46], S Cenk Sahinalp[149,156,157], Gordon Saksena[3], Iman Sarrafi[149,157], Matthias Schlesner[52,109], **Jared T Simpson#**[9,158], Lucy Stebbings[1], Chip Stewart[3], Miranda D Stobbe[133,134], Jon W Teague[1], Grace Tiao[3], David Torrents[46,110], Jeremiah A Wala[3,6,49], Jiayin Wang[137,152,159], Wenyi Wang[146], Sebastian M Waszak[8], Joachim Weischenfeldt[8,112,113], Michael C Wendl[137,160,161], Johannes Werner[52,114], David A Wheeler[162,163], Zhenggang Wu[150], Hong Xue[150], Sergei Yakneen[8], Takafumi N Yamaguchi[9], Kai Ye[159,164], Venkata D Yellapantula[165,166], Christina K Yung[45] and Junjun Zhang[45]

Drivers and functional interpretation

Federico Abascal[1], Samirkumar B Amin[167,168,169], Gary D Bader[10], Pratiti Bandopadhayay[3,170,171], Jonathan Barenboim[9], Rameen Beroukhim[3,6,172], Johanna Bertl[123,173], Keith A Boroevich[47,48], Søren Brunak[174,175], Peter J Campbell[1,2], Joana Carlevaro-Fita[176,177,178], Dimple Chakravarty[179,180], Calvin Wing Yiu Chan[52,181], Ken Chen[142], Jung Kyoon Choi[182], Jordi Deu-Pons[183,184], Priyanka Dhingra[116,117], Klev Diamanti[185], Lars Feuerbach[118], J Lynn Fink[46,186], Nuno A Fonseca[7,69], Joan Frigola[183], Carlo Gambacorti-Passerini[187], Dale W Garsed[188,189], **Mark Gerstein#**[119,120,121], **Gad Getz#**[3,4,5,6], Qianyun Guo[130], Ivo G Gut[133,134], David Haan[11], Mark P Hamilton[122], Nicholas J Haradhvala[3,104], Arif O Harmanci[121,190], Mohamed Helmy[191], Carl Herrmann[52,54,192], Julian M Hess[3,74], Asger Hobolth[130,173], Ermin Hodzic[157], Chen Hong[118,181], Henrik Hornshøj[123], Keren Isaev[9,131], Jose MG Izarzugaza[174], Rory Johnson[177,193], Todd A Johnson[47], Malene Juul[123], Randi Istrup Juul[123], Andre Kahles[89,90,91,92,93], Abdullah Kahraman[124,125,126], Manolis Kellis[3,127], Ekta Khurana[116,117,128,129], Jaegil Kim[3], Jong K Kim[194], Youngwook Kim[95,96], Jan Komorowski[185,195], Jan O Korbel[7,8], Sushant Kumar[120,121], Andrés Lanzós[177,178,193], Erik Larsson[89], **Michael S Lawrence#**[3,47,104], Donghoon Lee[121], Kjong-Van Lehmann[89,91,92,93,196], Shantao Li[121], Xiaotong Li[121], Ziao Lin[3,197], Eric Minwei Liu[116,117,198], Lucas Lochovsky[168,199,200,201], Shaoke Lou[120,121], Tobias Madsen[123], Kathleen Marchal[202,203], Iñigo Martincorena[1], Alexander Martinez-Fundichely[116,117,128], Yosef E Maruvka[3,74,104], Patrick D McGillivray[120], William Meyerson[121,204], Ferran Muiños[184,205], Loris Mularoni[184,205], Hidewaki Nakagawa[48], Morten Muhlig Nielsen[123], Marta Paczkowska[9], Keunchil Park[206,207], Kiejung Park[208], **Jakob Skou Pedersen#**[123,130], Tirso Pons[209], Sergio Pulido-Tamayo[202,203], **Benjamin J Raphael#**[210], Jüri Reimand[9,131], Iker Reyes-Salazar[205], Matthew A Reyna[210], Esther Rheinbay[3,6,104], Mark A Rubin[129,193,211,212,213], Carlota Rubio-Perez[184,205,214], S Cenk Sahinalp[149,156,157], Gordon Saksena[3], Leonidas Salichos[120,121], Chris Sander[49,89,215,216], Steven E Schumacher[3,217], Mark Shackleton[189,218], Ofer Shapira[3,49], Ciyue Shen[216,219], Raunak Shrestha[149], Shimin Shuai[9,10], Nikos Sidiropoulos[112], Lina Sieverling[118,181], Nasa Sinnott-Armstrong[3,61], Lincoln D Stein[9,10], **Joshua M Stuart#**[11], David Tamborero[184,205], Grace Tiao[3], Tatsuhiko Tsunoda[47,220,221,222], Husen M Umer[185,223], Liis Uusküla-Reimand[224,225], Alfonso Valencia[46,110], Miguel Vazquez[46,111], Lieven PC Verbeke[203,226], Claes Wadelius[227], Lina Wadi[9], Jiayin Wang[137,152,159], Jonathan Warrell[120,121], Sebastian M Waszak[8], Joachim Weischenfeldt[8,112,113], **David A Wheeler#**[162,163], Guanming Wu[228], Jun Yu[229], Jing Zhang[121], Xuanping Zhang[152,230], Yan Zhang[121,231,232], Zhongming Zhao[233], Lihua Zou[234] and Christian von Mering[126,132]

Integration of transcriptome and genome

Samirkumar B Amin[167,168,169], Philip Awadalla[9,10], Peter J Bailey[235], **Alvis Brazma#**[7], **Angela N Brooks#**[3,49,50], Claudia Calabrese[7,8], Aurélien Chateigner[45], Isidro Cortés-Ciriano[236,237,238], Brian Craft[239], David Craft[3,240], Chad J Creighton[241], Natalie R Davidson[89,91,92,108,196], Deniz Demircioğlu[242,243], Serap Erkek[8], Nuno A Fonseca[7,69], Milana Frenkel-Morgenstern[244], Mary J Goldman[239], Liliana Greger[7], Jonathan Göke[242,245], Yao He[246], Katherine A Hoadley[247,248], Yong Hou[39,249], Matthew R Huska[250], Andre Kahles[89,90,91,92,93], Ekta Khurana[116,117,128,129], Helena Kilpinen[251], Jan O Korbel[7,8], Fabien C Lamaze[9], Kjong-Van Lehmann[89,91,92,93,196], Chang Li[39,249], Siliang Li[39,249], Xiaobo Li[39,249], Xinyue Li[39], Dongbing Liu[39,249], Fenglin Liu[246,252], Xingmin Liu[39,249], Maximillian G Marin[50], Julia Markowski[250], Matthew Meyerson[3,6,49,177,253], Tannistha Nandi[254], Morten Muhlig Nielsen[123], Akinyemi I Ojesina[255,256,257], BF Francis Ouellette[100,101], Qiang Pan-Hammarström[39,258], Peter J Park[237,259], Chandra Sekhar Pedamallu[3,6,172], Jakob Skou Pedersen[123,130], Marc D Perry[45,102], **Gunnar Rätsch#**[89,92,105,106,107,108], Roland F Schwarz[7,83,250,260], Yuichi Shiraishi[86], Reiner Siebert[261,262], Cameron M Soulette[50], Stefan G Stark[92,196,263,264], Oliver Stegle[7,8,265], Hong Su[39,249], Patrick Tan[254,266,267,268], Bin Tean Teh[266,267,268,269,270], Lara Urban[7,8], Jian Wang[39], Sebastian M Waszak[8], Kui Wu[39,249], Qian Xiang[115], Heng Xiong[39,249], Sergei Yakneen[8], Huanming Yang[39], Chen Ye[39,249], Christina K Yung[45], Fan Zhang[246], Junjun Zhang[45], Xiuqing Zhang[39], Zemin Zhang[246,271], Liangtao Zheng[246], Jingchun Zhu[239] and Shida Zhu[39,249]

Integration of epigenome and genome

Hiroyuki Aburatani[272], **Benjamin P Berman#**[273,274,275], Hans Binder[276,277], **Benedikt Brors#**[81,118,278], Huy Q Dinh[273], Lars Feuerbach[118], Shengjie Gao[147], Ivo G Gut[133,134], Simon C Heath[133,134], Steve Hoffmann[276,277,279,280], Charles David Imbusch[118], Ekta Khurana[116,117,128,129], Helene Kretzmer[277,280], Peter W Laird[281], Jose I Martin-Subero[110,282], Genta Nagae[272,283], **Christoph Plass#**[284], Paz Polak[3,4,6], Hui Shen[285], Reiner Siebert[261,262], Nasa Sinnott-Armstrong[3,61], Miranda D Stobbe[133,134], Qi Wang[97], Dieter Weichenhan[284], Sergei Yakneen[8] and Wanding Zhou[285]

Patterns of structural variations, signatures, genomic correlations, retrotransposons, mobile elements

Kadir C Akdemir[142], Eva G Alvarez[286,287,288], Adrian Baez-Ortega[289], **Rameen Beroukhim#**[3,6,172], Paul C Boutros[9,131,140,141], David D L Bowtell[188,290], Benedikt Brors[81,118,278], Kathleen H Burns[291], John Busanovich[3,292], **Peter J Campbell#**[1,2], Kin Chan[293], Ken Chen[142], Isidro Cortés-Ciriano[236,237,238], Ana Dueso-Barroso[46], Andrew J Dunford[3], Paul A Edwards[294,295], Xavier Estivill[145,296], Dariush Etemadmoghadam[188,189], Lars Feuerbach[118], J Lynn Fink[46,186], Milana Frenkel-Morgenstern[244], Dale W Garsed[188,189], Mark Gerstein[119,120,121], Dmitry A Gordenin[297], David Haan[11], James E Haber[298], Julian M Hess[3,74], Barbara Hutter[79,80,81], Marcin Imielinski[299,300], David TW Jones[301,302], Young Seok Ju[1,182], Marat D Kazanov[303,304,305], Leszek J Klimczak[306], Youngil Koh[307,308], Jan O Korbel[7,8], Kiran Kumar[3], Eunjung Alice Lee[309], Jake June-Koo Lee[237,259],

Yilong Li[1], Andy G Lynch[294,295,310], Geoff Macintyre[294], Florian Markowetz[294,295], Iñigo Martincorena[1], Alexander Martinez-Fundichely[116,117,128], Matthew Meyerson[3,6,49,177,253], Satoru Miyano[86], Hidewaki Nakagawa[48], Fabio CP Navarro[120], Stephan Ossowski[134,145,155], Peter J Park[237,259], John V Pearson[311,312], Montserrat Puiggròs[46], Karsten Rippe[83], Nicola D Roberts[1], Steven A Roberts[313], Bernardo Rodriguez-Martin[286,287,288], Steven E Schumacher[3,217], Ralph Scully[314], Mark Shackleton[189,218], Nikos Sidiropoulos[112], Lina Sieverling[118,181], Chip Stewart[3], David Torrents[46,110], Jose MC Tubio[286,287,288], Izar Villasante[46], Nicola Waddell[311,312], Jeremiah A Wala[3,6,49], Joachim Weischenfeldt[8,112,113], Lixing Yang[315], Xiaotong Yao[299,316], Sung-Soo Yoon[308], Jorge Zamora[1,286,287,288] and Cheng-Zhong Zhang[3,6,49]

Mutation signatures and processes

Ludmil B Alexandrov[1,317], Erik N Bergstrom[318], Arnoud Boot[267,319], Paul C Boutros[9,131,140,141], Kin Chan[293], Kyle Covington[163], Akihiro Fujimoto[48], Gad Getz[3,4,5,6], Dmitry A Gordenin[297], Nicholas J Haradhvala[3,104], Mi Ni Huang[267,319], S. M. Ashiqul Islam[317], Marat D Kazanov[303,304,305], Jaegil Kim[3], Leszek J Klimczak[306], Michael S Lawrence[3,47,104], Iñigo Martincorena[1], John R McPherson[267,319], Sandro Morganella[1], Ville Mustonen[320,321,322], Hidewaki Nakagawa[48], Alvin Wei Tian Ng[323], Serena Nik-Zainal[1,324,325,326], Paz Polak[3,4,6], Stephenie D Prokopec[9], Steven A Roberts[313], **Steven G Rozen**#[267,268,319], Radhakrishnan Sabarinathan[184,205,327], Natalie Saini[297], Tatsuhiro Shibata[33,34], Yuichi Shiraishi[86], **Michael Rudolf Stratton**#[1], **Bin Tean Teh**#[266,267,268,269,270], Ignacio Vázquez-García[1,165,328,329], Yang Wu[267,319], Fouad Yousif[9] and Willie Yu[330]

Germline cancer genome

Ludmil B Alexandrov[1,317], Eva G Alvarez[286,287,288], Adrian Baez-Ortega[289], Matthew H Bailey[136,137], Mattia Bosio[46,134,145], G Steven Bova[331], Alvis Brazma[7], Alicia L Bruzos[286,287,288], Ivo Buchhalter[52,53,54], Carlos D Bustamante[60,61], Atul J Butte[332], Andy Cafferkey[7], Claudia Calabrese[7,8], Peter J Campbell[1,2], Stephen J Chanock[333], Nilanjan Chatterjee[334,335], Jieming Chen[121,336], Francisco M De La Vega[59,59,60,61,62], Olivier Delaneau[337,338,339], German M Demidov[134,145,340], Anthony DiBiase[341], Li Ding[136,137,144], Oliver Drechsel[134,145], Lewis Jonathan Dursi[9,65], Douglas F Easton[342,343], Serap Erkek[8], Georgia Escaramis[145,344,345], **Xavier Estivill**#[145,296], Erik Garrison[1], Mark Gerstein[119,120,121], Gad Getz[3,4,5,6], Dmitry A Gordenin[297], Nina Habermann[8], Olivier Harismendy[72], Eoghan Harrington[346], Shuto Hayashi[86], Seong Gu Heo[347], José María Heredia-Genestar[348], Aliaksei Z Holik[145], Eun Pyo Hong[347], Xing Hua[333], Kuan-lin Huang[137,349], Seiya Imoto[85,86], Sissel Juul[346], Ekta Khurana[116,117,128,129], Hyung-Lae Kim[27], Youngwook Kim[95,96], Leszek J Klimczak[306], **Jan O Korbel**#[7,8], Roelof Koster[350], Sushant Kumar[120,121], Ivica Letunic[153], Yilong Li[1], Tomas Marques-Bonet[110,133,348,351], R Jay Mashl[137,154], Simon Mayes[352], Michael D McLellan[136,137,144], Lisa Mirabello[333], Francesc Muyas[134,145,340], Hidewaki Nakagawa[48], Arcadi Navarro[110,133,348], Steven J Newhouse[7], Stephan Ossowski[134,145,155], Ji Wan Park[347], Esa Pitkänen[8], Aparna Prasad[134], Raquel Rabionet[134,145,353], Benjamin Raeder[8], Tobias Rausch[8], Steven A Roberts[313], Bernardo Rodriguez-Martin[286,287,288], Vasilisa A Rudneva[354], Gunnar Rätsch[89,92,105,106,107,108], Natalie Saini[297], Matthias Schlesner[52,109], Roland F Schwarz[7,83,250,260],

Ayellet V Segre[3,3,355], Tal Shmaya[59], Suyash S Shringarpure[61], Nikos Sidiropoulos[112], Reiner Siebert[261,262], Jared T Simpson[9,158], Lei Song[333], Oliver Stegle[7,8,265], Hana Susak[134,145], Tomas J Tanskanen[356], Grace Tiao[3], Marta Tojo[288], Jose MC Tubio[286,287,288], Daniel J Turner[352], Lara Urban[7,8], Sebastian M Waszak[8], David C Wedge[1,357,358], Joachim Weischenfeldt[8,112,113], David A Wheeler[162,163], Mark H Wright[61], Dai-Ying Wu[59], Tian Xia[359], Sergei Yakneen[8], Kai Ye[159,164], Venkata D Yellapantula[165,166], Jorge Zamora[1,286,287,288] and Bin Zhu[333]

Tumour subtypes and clinical translation

Fatima Al-Shahrour[360], Gurnit Atwal[9,10,361], Peter J Bailey[235], **Andrew V Biankin#**[362,363,364,365], Paul C Boutros[9,131,140,141], Peter J Campbell[1,2], David K Chang[363,365], Susanna L Cooke[365], Vikram Deshpande[104], Bishoy M Faltas[108], William C Faquin[104], **Levi Garraway#**[49], Gad Getz[3,4,5,6], **Sean M Grimmond#**[366], Syed Haider[9], **Katherine A Hoadley#**[247,248], Wei Jiao[9], Vera B Kaiser[367], Rosa Karlić[368], Mamoru Kato[369], Kirsten Kübler[3,6,104], Alexander J Lazar[370], Constance H Li[9,131], David N Louis[104], Adam Margolin[371], Sancha Martin[1,372], Hardeep K Nahal-Bose[45], G Petur Nielsen[104], Serena Nik-Zainal[1,324,325,326], Larsson Omberg[99], Christine P'ng[9], Marc D Perry[45,102], Paz Polak[3,4,6], Esther Rheinbay[3,6,104], Mark A Rubin[129,193,211,212,213], Colin A Semple[367], Dennis C Sgroi[104], Tatsuhiro Shibata[33,34], Reiner Siebert[261,262], Jaclyn Smith[371], **Lincoln D Stein#**[9,10], Miranda D Stobbe[133,134], Ren X Sun[9], Kevin Thai[45], Derek W Wright[373,374], Chin-Lee Wu[104], Ke Yuan[294,372,375] and Junjun Zhang[45]

Evolution and heterogeneity

David J Adams[1], Pavana Anur[376], Rameen Beroukhim[3,6,172], Paul C Boutros[9,131,140,141], David D L Bowtell[188,290], Peter J Campbell[1,2], Shaolong Cao[146], Elizabeth L Christie[188], Marek Cmero[377,378,379], Yupeng Cun[380], Kevin J Dawson[1], Jonas Demeulemeester[63,64], Stefan C Dentro[1,64,357], Amit G Deshwar[381], Nilgun Donmez[149,157], Ruben M Drews[294], Roland Eils[52,54,66,67], Yu Fan[146], Matthew W Fittall[64], Dale W Garsed[188,189], Moritz Gerstung[7,8], Gad Getz[3,4,5,6], Santiago Gonzalez[7,8], Gavin Ha[3], Kerstin Haase[64], Marcin Imielinski[299,300], Lara Jerman[8,382], Yuan Ji[383,384], Clemency Jolly[64], Kortine Kleinheinz[52,54], Juhee Lee[385], Henry Lee-Six[1], Ignaty Leshchiner[3], Dimitri Livitz[3], Geoff Macintyre[294], Salem Malikic[149,157], Florian Markowetz[294,295], Iñigo Martincorena[1], Thomas J Mitchell[1,295,386], Quaid D Morris[361,387], Ville Mustonen[320,321,322], Layla Oesper[388], Martin Peifer[380], Myron Peto[389], Benjamin J Raphael[210], Daniel Rosebrock[3], Yulia Rubanova[158,361], S Cenk Sahinalp[149,156,157], Adriana Salcedo[9], Matthias Schlesner[52,109], Steven E Schumacher[3,217], Subhajit Sengupta[390], Ruian Shi[387], Seung Jun Shin[264], **Paul T Spellman#**[391], Oliver Spiro[3], Lincoln D Stein[9,10], Maxime Tarabichi[1,64], **Peter Van Loo#**[63,64], Shankar Vembu[387,392], Ignacio Vázquez-García[1,165,328,329], Wenyi Wang[146], **David C Wedge#**[1,357,358], David A Wheeler[162,163], Jeffrey A Wintersinger[191,361,393], Tsun-Po Yang[380], Xiaotong Yao[299,316], Kaixian Yu[394], Ke Yuan[294,372,375] and Hongtu Zhu[395,396]

Exploratory: portals, visualization and software infrastructure

Fatima Al-Shahrour[360], Elisabet Barrera[7], Wojciech Bazant[7], Alvis Brazma[7], Isidro Cortés-Ciriano[236,237,238], Brian Craft[239], David Craft[3,240], Vincent Ferretti[45,68], Nuno A Fonseca[7,69], Anja Füllgrabe[7], Mary J Goldman[239], **David Haussler#**[239,397], Wolfgang Huber[8], Maria Keays[7], Alfonso Muñoz[7], Brian D O'Connor[45,50], Irene Papatheodorou[7], Robert Petryszak[7], Elena Piñeiro-Yáñez[360], Alfonso Valencia[46,110], **Miguel Vazquez#**[46,111], John N Weinstein[398,399], Qian Xiang[115], Junjun Zhang[45] and **Jingchun Zhu#**[239]

Exploratory: mitochondrial variants and HLA/immunogenicity

Peter J Campbell[1,2], Yiwen Chen[146], Chad J Creighton[241], Li Ding[136,137,144], Akihiro Fujimoto[48], Masashi Fujita[48], Gad Getz[3,4,5,6], Leng Han[230], Takanori Hasegawa[86], Shuto Hayashi[86], Seiya Imoto[85,86], Young Seok Ju[1,182], Hyung-Lae Kim[27], Youngwook Kim[95,96], Youngil Koh[307,308], Mitsuhiro Komura[86], Jun Li[146], **Han Liang#**[400], Iñigo Martincorena[1], Satoru Miyano[86], Shinichi Mizuno[401], **Hidewaki Nakagawa#**[48], Keunchil Park[206,207], Eigo Shimizu[86], Yumeng Wang[146,402], John N Weinstein[398,399], Yanxun Xu[403], Rui Yamaguchi[86], Fan Yang[387], Yang Yang[230], Christopher J Yoon[182], Sung-Soo Yoon[308], Yuan Yuan[146], Fan Zhang[246] and Zemin Zhang[246,271]

Exploratory: pathogens

Malik Alawi[404,405], Ivan Borozan[9], Daniel S Brewer[406,407], Colin S Cooper[407,408,409], Nikita Desai[45], **Roland Eils#**[52,54,66,67], Vincent Ferretti[45,68], Adam Grundhoff[404,410], Murat Iskar[411], Kortine Kleinheinz[52,54], **Peter Lichter#**[79,411], Hidewaki Nakagawa[48], Akinyemi I Ojesina[255,256,257], Chandra Sekhar Pedamallu[3,6,172], Matthias Schlesner[52,109], Xiaoping Su[142] and Marc Zapatka[411]

Tumor Specific Providers – Australia (Ovarian cancer)

Kathryn Alsop[412,413], Australian Ovarian Cancer Study Group[188,311,414], **David D L Bowtell#**[188,290], Timothy JC Bruxner[186], Angelika N Christ[186], Elizabeth L Christie[188], Stephen M Cordner[415], Prue A Cowin[188], Ronny Drapkin[416], Dariush Etemadmoghadam[188,189], Sian Fereday[417], Dale W Garsed[188,189], Joshy George[168], Sean M Grimmond[366], Anne Hamilton[188], Oliver Holmes[311,312], Jillian A Hung[418,419], Karin S Kassahn[186,420], Stephen H Kazakoff[311,312], Catherine J Kennedy[421,422], Conrad R Leonard[311,312], Linda Mileshkin[188], David K Miller[186,363,423], Gisela Mir Arnau[188], Chris Mitchell[188], Felicity Newell[311,312], Katia Nones[311,312], Ann-Marie Patch[311,312], John V Pearson[311,312], Michael C Quinn[311,312], Mark Shackleton[189,218], Darrin F Taylor[186], Heather Thorne[188], Nadia Traficante[188], Ravikiran Vedururu[188], Nick M Waddell[312], Nicola Waddell[311,312], Paul M Waring[253], Scott Wood[311,312], Qinying Xu[311,312] and Anna deFazio[424,425,426]

Tumor Specific Providers – Australia (Pancreatic cancer)

Matthew J Anderson[186], Davide Antonello[427], Andrew P Barbour[428,429], Claudio Bassi[427], Samantha Bersani[430], **Andrew V Biankin#**[362,363,364,365], Timothy JC Bruxner[186], Ivana Cataldo[430,431], David K Chang[363,365], Lorraine A Chantrill[363,432], Yoke-Eng Chiew[424], Angela Chou[363,433], Angelika N Christ[186], Sara Cingarlini[37], Nicole Cloonan[434], Vincenzo Corbo[431,435,436], M V Davi[436],Fraser R Duthie[437,438], J Lynn Fink[46,186], Anthony J Gill[363,439], Janet S Graham[365,440], **Sean M Grimmond#**[366], Ivon Harliwong[186], Oliver Holmes[311,312], Nigel B Jamieson[364,365,441], Amber L Johns[363,423], Karin S Kassahn[186,420], Stephen H Kazakoff[311,312], James G Kench[363,439,442], Luca Landoni[427], Rita T Lawlor[431], Conrad R Leonard[311,312], Andrea Mafficini[431], Neil D Merrett[427,443], David K Miller[186,363,423], Marco Miotto[427], Elizabeth A Musgrove[365], Adnan M Nagrial[363], Felicity Newell[311,312], Katia Nones[311,312], Karin A Oien[253,444], Marina Pajic[363], Ann-Marie Patch[311,312], John V Pearson[311,312], Mark Pinese[445], Michael C Quinn[311,312], Alan J Robertson[186], Ilse Rooman[363], Borislav C Rusev[431], Jaswinder S Samra[427,439], Maria Scardoni[430], Christopher J Scarlett[363,446], Aldo Scarpa[431], Elisabetta Sereni[427], Katarzyna O Sikora[431], Michele Simbolo[435], Morgan L Taschuk[45], Christopher W Toon[363], Giampaolo Tortora[37,38], Caterina Vicentini[431], Nick M Waddell[312], Nicola Waddell[311,312], Scott Wood[311,312], Jianmin Wu[363], Qinying Xu[311,312] and Nikolajs Zeps[447]

Tumor Specific Providers – Australia (Skin cancer)

Lauri A Aaltonen[448], Andreas Behren[449], Hazel Burke[450], Jonathan Cebon[449], Rebecca A Dagg[451], Ricardo De Paoli-Iseppi[452], Ken Dutton-Regester[311], Matthew A Field[453], Anna Fitzgerald[454], Sean M Grimmond[366], **Nicholas K Hayward#**[311,450], Peter Hersey[450], Oliver Holmes[311,312], Valerie Jakrot[450], Peter A Johansson[311], Hojabr Kakavand[452], Stephen H Kazakoff[311,312], Richard F Kefford[455], Loretta MS Lau[456], Conrad R Leonard[311,312], Georgina V Long[457], **Graham J Mann#**[458,459], Felicity Newell[311,312], Katia Nones[311,312], Ann-Marie Patch[311,312], John V Pearson[311,312], Hilda A Pickett[456], Antonia L Pritchard[311], Gulietta M Pupo[460], Robyn PM Saw[457], Sarah-Jane Schramm[461], **Richard A Scolyer#**[425,457,462,463], Mark Shackleton[189,218], Catherine A Shang[464], Ping Shang[457], Andrew J Spillane[457], Jonathan R Stretch[457], Varsha Tembe[461], John F Thompson[457], Ricardo E Vilain[462], Nick M Waddell[312], Nicola Waddell[311,312], James S Wilmott[457], Scott Wood[311,312], Qinying Xu[311,312] and Jean Y Yang[465]

Tumor Specific Providers – Canada (Pancreatic cancer)

John Bartlett[466,467], Prashant Bavi[468], Ivan Borozan[9], Dianne E Chadwick[469], Michelle Chan-Seng-Yue[468], Sean Cleary[468,470], Ashton A Connor[471,472], Karolina Czajka[473], Robert E Denroche[468], Neesha C Dhani[474], Jenna Eagles[78], Vincent Ferretti[45,68], Steven Gallinger[468,471,472], Robert C Grant[468,475], David Hedley[474], Michael A Hollingsworth[476], **Thomas J Hudson#**[77,78], Gun Ho Jang[468], Jeremy Johns[78], Sangeetha Kalimuthu[468], Sheng-Ben Liang[477], Ilinca Lungu[468,478], Xuemei

Luo[9], Faridah Mbabaali[78], **John D McPherson#**[78,468,479], Treasa A McPherson[475], Jessica K Miller[78], Malcolm J Moore[474], Faiyaz Notta[468,480], Danielle Pasternack[78], Gloria M Petersen[481], Michael H A Roehrl[131,468,482,483,484], Michelle Sam[78], Iris Selander[475], Stefano Serra[253], Sagedeh Shahabi[477], **Lincoln D Stein#**[9,10], Morgan L Taschuk[45], Sarah P Thayer[485], Lee E Timms[78], Gavin W Wilson[9,468], Julie M Wilson[468] and Bradly G Wouters[486]

Tumor Specific Providers – Canada (Prostate cancer)

Timothy A Beck[45,487], Vinayak Bhandari[9], **Paul C Boutros#**[9,131,140,141], **Robert G Bristow#**[131,488,489,490,491], Colin C Collins[149], Shadrielle MG Espiritu[9], Neil E Fleshner[492], Natalie S Fox[9], Michael Fraser[9], Syed Haider[9], Lawrence E Heisler[493], Vincent Huang[9], Emilie Lalonde[9], Julie Livingstone[9], John D McPherson[78,468,479], Alice Meng[494], Veronica Y Sabelnykova[9], Adriana Salcedo[9], Yu-Jia Shiah[9], Theodorus Van der Kwast[495] and Takafumi N Yamaguchi[9]

Tumor Specific Providers – China (Gastric cancer)

Shuai Ding[496], Daiming Fan[497], Yong Hou[39,249], Yi Huang[151,152], Lin Li[39], Siliang Li[39,249], Dongbing Liu[39,249], Xingmin Liu[39,249], **Youyong Lu#**[28,29,30], Yongzhan Nie[497,498], Hong Su[39,249], Jian Wang[39], Kui Wu[39,249], Xiao Xiao[152], Rui Xing[29,499], **Huanming Yang#**[39], Shanlin Yang[496], Yingyan Yu[500], Xiuqing Zhang[39], Yong Zhou[39] and Shida Zhu[39,249]

Tumor Specific Providers – EU: France (Renal cancer)

Rosamonde E Banks[501], Guillaume Bourque[502,503], Alvis Brazma[7], Paul Brennan[504], **Mark Lathrop#**[503], Louis Letourneau[505], Yasser Riazalhosseini[503], Ghislaine Scelo[504], **Jörg Tost#**[506], Naveen Vasudev[507] and Juris Viksna[508]

Tumor Specific Providers – EU: United Kingdom (Breast cancer)

Sung-Min Ahn[509], Ludmil B Alexandrov[1,317], Samuel Aparicio[510], Laurent Arnould[511], MR Aure[512], Shriram G Bhosle[1], E Birney[7], Ake Borg[513], S Boyault[514], AB Brinkman[515], JE Brock[516], A Broeks[517], Adam P Butler[1], AL Børresen-Dale[512], C Caldas[518,519], Peter J Campbell[1,2], Suet-Feung Chin[518,519], Helen Davies[1,324,325], C Desmedt[520], L Dirix[521], S Dronov[1], Anna Ehinger[522], JE Eyfjord[523], GG Van den Eynden[524], A Fatima[217], Jorge Reis-Filho[525], JA Foekens[526], PA Futreal[527], Øystein Garred[528,529], Moritz Gerstung[7,8], Dilip D Giri[525], D Glodzik[1], Dorthe Grabau[530], Holmfridur Hilmarsdottir[523], GK Hooijer[531], Jocelyne Jacquemier[532], SJ Jang[533], Jon G Jonasson[523], Jos Jonkers[534], HY Kim[532], Tari A King[535,536], Stian Knappskog[1,537], G Kong[532], S Krishnamurthy[538], S Van Laere[521], SR Lakhani[539], Denis Larsimont[540], HJ Lee[533], JY Lee[541], Ming Ta Michael Lee[527], Yilong Li[1], Ole Christian Lingjærde[542], Gaetan MacGrogan[543], Sancha Martin[1,372], Iñigo Martincorena[1], Andrew Menzies[1], Sandro Morganella[1], Ville Mustonen[320,321,322], Serena Nik-

Zainal[1,324,325,326], Sarah O'Meara[1], I Pauporté[18], Sarah Pinder[544], X Pivot[545], Elena Provenzano[546], CA Purdie[547], Keiran M Raine[1], M Ramakrishna[1], K Ramakrishnan[1], AL Richardson[217], M Ringnér[513], Javier Bartolomé Rodriguez[46], FG Rodríguez-González[175], G Romieu[548], Roberto Salgado[253], Torill Sauer[542], R Shepherd[1], AM Sieuwerts[177], PT Simpson[539], M Smid[549], C Sotiriou[55], PN SpanSpan[550], Lucy Stebbings[1], Ólafur Andri Stefánsson[551], Alasdair Stenhouse[552], **Michael Rudolf Stratton**#[1], HG Stunnenberg[249,553], Fred Sweep[554], BK Tan[555], Jon W Teague[1], Gilles Thomas[556], AM Thompson[552], S Tommasi[557], I Treilleux[558,559], Andrew Tutt[217], NT Ueno[396], Peter Van Loo[63,64], P Vermeulen[521], Alain Viari[431], A Vincent-Salomon[553], David C Wedge[1,357,358], Bernice Huimin Wong[560], Lucy Yates[1], X Zou[1], CHM van Deurzen[561], MJ van de Vijver[253] and L van't Veer[562,563]

Tumor Specific Providers – Germany (Malignant lymphoma)

Ole Ammerpohl[564,565], Sietse Aukema[566,567], Anke K Bergmann[568], Stephan H Bernhart[276,277,280], Hans Binder[276,277], Arndt Borkhardt[569], Christoph Borst[570], Benedikt Brors[81,118,278], Birgit Burkhardt[571], Alexander Claviez[572], Roland Eils[52,54,66,67], Maria Elisabeth Goebler[573], Andrea Haake[564], Siegfried Haas[570], Martin Hansmann[574], Jessica I Hoell[569], Steve Hoffmann[276,277,279,280], Michael Hummel[575], Daniel Hübschmann[54,66,82,83,84], Dennis Karsch[576], Wolfram Klapper[567], Kortine Kleinheinz[52,54], Michael Kneba[576], Jan O Korbel[7,8], Helene Kretzmer[277,280], Markus Kreuz[577], Dieter Kube[578], Ralf Küppers[579], Chris Lawerenz[67], Dido Lenze[575], Peter Lichter[79,411], Markus Loeffler[577], Cristina López[262,564], Luisa Mantovani-Löffler[580], Peter Möller[581], German Ott[582], Bernhard Radlwimmer[411], Julia Richter[564,567], Marius Rohde[583], Philip C Rosenstiel[584], Andreas Rosenwald[585], Markus B Schilhabel[584], Matthias Schlesner[52,109], Stefan Schreiber[586], **Reiner Siebert**#[261,262], Peter F Stadler[276,277,280], Peter Staib[587], Stephan Stilgenbauer[588], Stephanie Sungalee[8], Monika Szczepanowski[567], Umut H Toprak[54,589], Lorenz HP Trümper[578], Rabea Wagener[262,564] and Thorsten Zenz[81]

Tumor Specific Providers – Germany (Pediatric Brain cancer)

Ivo Buchhalter[52,53,54], Juergen Eils[66,67], Roland Eils[52,54,66,67], Volker Hovestadt[411], Barbara Hutter[79,80,81], David TW Jones[301,302], Natalie Jäger[52], Christof von Kalle[83], Marcel Kool[97,301], Jan O Korbel[7,8], Andrey Korshunov[97], Pablo Landgraf[590, 770], Chris Lawerenz[67], Hans Lehrach[591], **Peter Lichter**#[79,411], Paul A Northcott[592], Stefan M Pfister[97,301,593], Bernhard Radlwimmer[411], Guido Reifenberger[590], Matthias Schlesner[52,109], Hans-Jörg Warnatz[591], Joachim Weischenfeldt[8,112,113], Stephan Wolf[594], Marie-Laure Yaspo[591] and Marc Zapatka[411]

Tumor Specific Providers – Germany (Prostate cancer)

Yassen Assenov[595], Benedikt Brors[81,118,278], Juergen Eils[66,67], Roland Eils[52,54,66,67], Lars Feuerbach[118], Clarissa Gerhauser[284], Jan O Korbel[7,8], Chris Lawerenz[67], Hans Lehrach[591], Sarah Minner[596], Christoph Plass[284], **Guido Sauter**#[597], Thorsten Schlomm[113,598], Nikos Sidiropoulos[112],

Ronald Simon[597], **Holger Sültmann#**[81,599], Hans-Jörg Warnatz[591], Dieter Weichenhan[284], Joachim Weischenfeldt[8,112,113] and Marie-Laure Yaspo[591]

Tumor Specific Providers – India (Oral cancer)

Nidhan K Biswas[600], Luca Landoni[427], Arindam Maitra[600], **Partha P Majumder#**[600] and **Rajiv Sarin#**[601]

Tumor Specific Providers – Italy (Pancreatic cancer)

Davide Antonello[427], Stefano Barbi[435], Claudio Bassi[427], Samantha Bersani[430], Giada Bonizzato[431], Cinzia Cantù[431], Ivana Cataldo[430,431], Sara Cingarlini[37], Vincenzo Corbo[431,435], [436], M V Davi[436],Angelo P Dei Tos[602], Matteo Fassan[603], Sonia Grimaldi[431], Luca Landoni[427], Rita T Lawlor[431], Claudio Luchini[430], Andrea Mafficini[431], Giuseppe Malleo[427], Giovanni Marchegiani[427], Michele Milella[37], Marco Miotto[427], Salvatore Paiella[427], Antonio Pea[427], Paolo Pederzoli[427], Borislav C Rusev[431], Andrea Ruzzenente[427], Roberto Salvia[427], Maria Scardoni[430], **Aldo Scarpa#**[431], Elisabetta Sereni[427], Michele Simbolo[435], Nicola Sperandio[431], Giampaolo Tortora[37,38] and Caterina Vicentini[431]

Tumor Specific Providers – Japan (Biliary tract cancer)

Yasuhito Arai[33], Natsuko Hama[33], Nobuyoshi Hiraoka[604], Fumie Hosoda[33,605], Mamoru Kato[369], Hiromi Nakamura[33], Hidenori Ojima[606], Takuji Okusaka[607], **Tatsuhiro Shibata#**[33,34], Yasushi Totoki[33] and Tomoko Urushidate[34]

Tumor Specific Providers – Japan (Gastric cancer)

Hiroyuki Aburatani#[272], Yasuhito Arai[33], Masashi Fukayama[608], Natsuko Hama[33], Fumie Hosoda[33,605], Shumpei Ishikawa[609], Hitoshi Katai[610], Mamoru Kato[369], Hiroto Katoh[611], Daisuke Komura[609], Genta Nagae[272,283], Hiromi Nakamura[33], Hirofumi Rokutan[612], Mihoko Saito-Adachi[33], **Tatsuhiro Shibata#**[33,34], Akihiro Suzuki[272,613], Hirokazu Taniguchi[614], Kenji Tatsuno[272], Yasushi Totoki[33], Tetsuo Ushiku[608], Shinichi Yachida[33,615] and Shogo Yamamoto[272]

Tumor Specific Providers – Japan (Liver cancer)

Hiroyuki Aburatani[272], Hiroshi Aikata[616], Koji Arihiro[616], Shun-ichi Ariizumi[617], Keith A Boroevich[47,48], Kazuaki Chayama[616], Akihiro Fujimoto[48], Masashi Fujita[48], Mayuko Furuta[48], Kunihito Gotoh[618], Natsuko Hama[33], Takanori Hasegawa[86], Shinya Hayami[619], Shuto Hayashi[86], Satoshi Hirano[620], Seiya Imoto[85,86], Mamoru Kato[369], Yoshiiku Kawakami[616], Kazuhiro Maejima[48],

Satoru Miyano[86], Genta Nagae[272,283], **Hidewaki Nakagawa#**[48], Hiromi Nakamura[33], Toru Nakamura[620], Kaoru Nakano[48], Hideki Ohdan[616], Aya Sasaki-Oku[48], **Tatsuhiro Shibata#**[33,34], Yuichi Shiraishi[86], Hiroko Tanaka[86], Yasushi Totoki[33], Tatsuhiko Tsunoda[47,220,221,222], Masaki Ueno[619], Rui Yamaguchi[86], Masakazu Yamamoto[617] and Hiroki Yamaue[619]

Tumor Specific Providers – Singapore (Biliary tract cancer)

Su Pin Choo[621], Ioana Cutcutache[267,319], Narong Khuntikeo[427,622], John R McPherson[267,319], Choon Kiat Ong[623], Chawalit Pairojkul[253], Irinel Popescu[624], **Steven G Rozen#**[267,268,319], **Patrick Tan#**[254,266,267,268] and **Bin Tean Teh#**[266,267,268,269,270]

Tumor Specific Providers – South Korea (Blood cancer)

Keun Soo Ahn[625], Hyung-Lae Kim[27], Youngil Koh[307,308] and **Sung-Soo Yoon#**[308]

Tumor Specific Providers – Spain (Chronic Lymphocytic Leukemia)

Marta Aymerich[626], **Elias Campo#**[627,628], Josep Ll Gelpi[46,70], Ivo G Gut[133,134], Marta Gut[133,134], Armando Lopez-Guillermo[629], Carlos López-Otín[630], Xose S Puente[631], Romina Royo[46] and David Torrents[46,110]

Tumor Specific Providers – United Kingdom (Bone cancer)

Fernanda Amary[632], Daniel Baumhoer[633], Sam Behjati[1], Bodil Bjerkehagen[634], **Peter J Campbell#**[1,2], **Adrienne M Flanagan#**[635], PA Futreal[527], Ola Myklebost[636], Nischalan Pillay[637], Patrick Tarpey[638], Roberto Tirabosco[639] and Olga Zaikova[640]

Tumor Specific Providers – United Kingdom (Chronic myeloid disorders)

Jacqueline Boultwood[641], David T Bowen[1], Adam P Butler[1], **Peter J Campbell#**[1,2], Mario Cazzola[642], Carlo Gambacorti-Passerini[187], Anthony R Green[295], Eva Hellstrom-Lindberg[643], Luca Malcovati[642], Sancha Martin[1,372], Jyoti Nangalia[644], Elli Papaemmanuil[1] and Paresh Vyas[311,645]

Tumor Specific Providers – United Kingdom (Esophageal cancer)

Yeng Ang[646], Hugh Barr[647], Duncan Beardsmore[648], Matthew Eldridge[294], **Rebecca C Fitzgerald#**[325], James Gossage[649], Nicola Grehan[325], George B Hanna[650], Stephen J Hayes[651,652],

Ted R Hupp[653], David Khoo[654], Jesper Lagergren[643,655], Laurence B Lovat[251], Shona MacRae[398], Maria O'Donovan[325], J Robert O'Neill[656], Simon L Parsons[657], Shaun R Preston[658], Sonia Puig[659], Tom Roques[660], Grant Sanders[248], Sharmila Sothi[661], Simon Tavaré[294], Olga Tucker[662], Richard Turkington[663], Timothy J Underwood[664] and Ian Welch[665]

Tumor Specific Providers – United Kingdom (Prostate cancer)

Nicholas vVan As[666], Daniel M Berney[667], Johann S De Bono[408], G Steven Bova[331], Daniel S Brewer[406,407], Adam P Butler[1], Declan Cahill[666], Niedzica Camacho[408], **Colin S Cooper#**[407,408,409], Nening M Dennis[666], Tim Dudderidge[666,668], Sandra E Edwards[408], **Rosalind A Eeles#**[408,666], Cyril Fisher[666], Christopher S Foster[669,670], Mohammed Ghori[1], Pelvender Gill[645], Vincent J Gnanapragasam[386,671], Gunes Gundem[198], Freddie C Hamdy[672], Steve Hawkins[294], Steven Hazell[666], William Howat[386], William B Isaacs[291], Katalin Karaszi[645], Jonathan D Kay[251], Vincent Khoo[666], Zsofia Kote-Jarai[408], Barbara Kremeyer[1], Pardeep Kumar[666], Adam Lambert[645], Daniel A Leongamornlert[1,408], Naomi Livni[666], Yong-Jie Lu[667,673], Hayley J Luxton[251], Andy G Lynch[294,295,310], Luke Marsden[645], Charlie E Massie[294], Lucy Matthews[408], Erik Mayer[666,674], Ultan McDermott[1], Sue Merson[408], Thomas J Mitchell[1,295,386], David E Neal[294,386], Anthony Ng[675], David Nicol[666], Christopher Ogden[666], Edward W Rowe[666], Nimish C Shah[386], Jon W Teague[1], Sarah Thomas[666], Alan Thompson[666], Peter Van Loo[63,64], Clare Verrill[645,676], Tapio Visakorpi[331], Anne Y Warren[386,677], David C Wedge[1,357,358], Hayley C Whitaker[251], Jorge Zamora[1,286,287,288] and Hongwei Zhang[673]

Tumor Specific Providers – United States (TCGA)

Adam Abeshouse[198], Nishant Agrawal[71], Rehan Akbani[325,678], Hikmat Al-Ahmadie[198], Monique Albert[467], Kenneth Aldape[253,654,679], Adrian Ally[680], Yeng Ang[646], Elizabeth L Appelbaum[137,251], Joshua Armenia[681], Sylvia Asa[657,682], J Todd Auman[683], Matthew H Bailey[136,137], Miruna Balasundaram[680], Saianand Balu[248], Jill Barnholtz-Sloan[684,685], Hugh Barr[647], John Bartlett[466,467], Oliver F Bathe[686,687], Stephen B Baylin[6684,688], Duncan Beardsmore[648], Christopher Benz[689], Andrew Berchuck[690], Benjamin P Berman[273,274,275], Rameen Beroukhim[3,6,172], Mario Berrios[691], Darell Bigner[294,692], Michael Birrer[104], Tom Bodenheimer[248], Lori Boice[659], Moiz S Bootwalla[693], Marcus Bosenberg[694], Reanne Bowlby[680], Jeffrey Boyd[695], Russell R Broaddus[679], Malcolm Brock[696], Denise Brooks[680], Susan Bullman[3,172], Samantha J Caesar-Johnson[40], Thomas E Carey[697], Rebecca Carlsen[680], Robert Cerfolio[698], Vishal S Chandan[699], Hsiao-Wei Chen[646,681], Andrew D Cherniack[3,3,49,172], Jeremy Chien[700], Juok Cho[3], Eric Chuah[680], Carrie Cibulskis[3], Kristian Cibulskis[3], Leslie Cope[701], Matthew G Cordes[137,660], Kyle Covington[163], Erin Curley[702], Bogdan Czerniak[654,679], Ludmila Danilova[701], Ian J Davis[703], Timothy Defreitas[3], John A Demchok[40], Noreen Dhalla[680], Rajiv Dhir[704], Li Ding[136,137,144], HarshaVardhan Doddapaneni[163], Adel El-Naggar[654,679], Ina Felau[40], Martin L Ferguson[705], Gaetano Finocchiaro[706], Kwun M Fong[707], Scott Frazer[3], William Friedman[708], Catrina C Fronick[137,660], Lucinda A Fulton[137], Robert S Fulton[136,137,144], Stacey B Gabriel[3], Jianjiong Gao[681], Nils Gehlenborg[3,709], Jeffrey E Gershenwald[710,711], Gad Getz[3,4,5,6], Ronald Ghossein[525], Nasra H Giama[712], Richard A Gibbs[163],

Carmen Gomez[713], James Gossage[649], Ramaswamy Govindan[136], Nicola Grehan[325], George B Hanna[650], Stephen J Hayes[651,652], Apurva M Hegde[398,678], David I Heiman[3], Zachary Heins[198], Austin J Hepperla[248], Katherine A Hoadley[247,248], Andrea Holbrook[714], Robert A Holt[680], Alan P Hoyle[248], Ralph H Hruban[715], Jianhong Hu[163], Mei Huang[659], David Huntsman[716], Ted R Hupp[653], Jason Huse[198], **Carolyn M Hutter#**[21], Christine A Iacobuzio-Donahue[525], Michael Ittmann[717,718], Joy C Jayaseelan[163], Stuart R Jefferys[248], Corbin D Jones[719], Steven JM Jones[720], Hartmut Juhl[721], Koo Jeong Kang[722], Beth Karlan[723], Katayoon Kasaian[724], Electron Kebebew[725,726], David Khoo[654], Hark Kim[31], Jaegil Kim[3], Tari A King[535,536], Viktoriya Korchina[163], Ritika Kundra[646,681], Jesper Lagergren[643,655], Phillip H Lai[714], Peter W Laird[281], Eric Lander[3], Michael S Lawrence[3,47,104], Alexander J Lazar[370], Xuan Le[727], Darlene Lee[680], Douglas A Levine[198,728], Lora Lewis[163], Tim Ley[729], Haiyan Irene Li[680], Pei Lin[3], W M Linehan[730], Eric Minwei Liu[116,117,198], Fei Fei Liu[387], Laurence B Lovat[251], Yiling Lu[731], Lisa Lype[732], Yussanne Ma[680], Shona MacRae[398], Dennis T Maglinte[714], Elaine R Mardis[137,695,733], Jeffrey Marks[427,734], Marco A Marra[680], Thomas J Matthew[50], Michael Mayo[680], Karen McCune[735], Michael D McLellan[136,137,144], Samuel R Meier[3], Shaowu Meng[248], Matthew Meyerson[3,6,49,177,253], Piotr A Mieczkowski[247], Tom Mikkelsen[736], Christopher A Miller[137], Gordon B Mills[371,398,678], Richard A Moore[680], Carl Morrison[253,737], Lisle E Mose[248], Catherine D Moser[712], Andrew J Mungall[680], Karen Mungall[680], David Mutch[738], Donna M Muzny[163], Jerome Myers[739], Yulia Newton[50], Michael S Noble[3], Peter O'Donnell[740], Brian Patrick O'Neill[741], Angelica Ochoa[198], Akinyemi I Ojesina[255,256,257], Joong Won Park[31], Joel S Parker[742], Simon L Parsons[657], Harvey Pass[743], Alessandro Pastore[89], Chandra Sekhar Pedamallu[3,6,172], Nathan A Pennell[744], Charles M Perou[745], Gloria M Petersen[481], Nicholas Petrelli[746], Olga Potapova[747], Shaun R Preston[658], Sonia Puig[659], Janet S Rader[748], Suresh Ramalingam[749], W Kimryn Rathmell[750], Victor Reuter[253,525], Sheila M Reynolds[732], Matthew Ringel[751], Jeffrey Roach[752], Lewis R Roberts[712], A Gordon Robertson[680], Tom Roques[660], Mark A Rubin[129,193,211,212,213], Sara Sadeghi[680], Gordon Saksena[3], Charles Saller[753], Francisco Sanchez-Vega[646,681], Chris Sander[49,89,215,216], Grant Sanders[248], Dirk Schadendorf[79,754], Jacqueline E Schein[680], Heather K Schmidt[137], Nikolaus Schultz[681], Steven E Schumacher[3,217], Richard A Scolyer[425,457,462,463], Raja Seethala[755], Yasin Senbabaoglu[89], Troy Shelton[702], Yan Shi[248], Juliann Shih[3,172,177], Ilya Shmulevich[732], Craig Shriver[756], Sabina Signoretti[172,177,757], Janae V Simons[248], Samuel Singer[427,758], Payal Sipahimalani[680], Tara J Skelly[247], Karen Smith-McCune[735], Nicholas D Socci[89], Heidi J Sofia[21], Matthew G Soloway[742], Anil K Sood[759], Sharmila Sothi[661], Angela Tam[680], Donghui Tan[247], Roy Tarnuzzer[40], Nina Thiessen[680], R Houston Thompson[760], Leigh B Thorne[659], Ming Tsao[657,682], Olga Tucker[662], Richard Turkington[663], Christopher Umbricht[648,761], Timothy J Underwood[664], David J Van Den Berg[691], Erwin G Van Meir[762], Umadevi Veluvolu[247], Douglas Voet[3], Jiayin Wang[137,152,159], Linghua Wang[163], Zhining Wang[40], Paul Weinberger[763], John N Weinstein[398,399], Daniel J Weisenberger[714], Ian Welch[665], David A Wheeler[162,163], Dennis Wigle[764], Matthew D Wilkerson[247], Richard K Wilson[137,765], Boris Winterhoff[766], Maciej Wiznerowicz[767,768], Tina Wong[137,680], Winghing Wong[769], Liu Xi[163], Liming Yang[40], Christina Yau[294,689,690], Venkata D Yellapantula[165,166], **Jean C Zenklusen#**[40], Hailei Zhang[3], Hongxin Zhang[681] and Jiashan Zhang[40]

# Denotes **working group or project co-leader**

Author Affiliations

1. Wellcome Sanger Institute, Wellcome Genome Campus, Hinxton, Cambridge, CB10 1SA, UK.
2. Department of Haematology, University of Cambridge, Cambridge CB2 2XY, UK.
3. Broad Institute of MIT and Harvard, Cambridge, MA 02142, USA.
4. Center for Cancer Research, Massachusetts General Hospital, Boston, MA 02129, USA.
5. Department of Pathology, Massachusetts General Hospital, Boston, MA 02115, USA.
6. Harvard Medical School, Boston, MA 02115, USA.
7. European Molecular Biology Laboratory, European Bioinformatics Institute (EMBL-EBI), Wellcome Genome Campus, Hinxton, Cambridge, CB10 1SD, UK.
8. Genome Biology Unit, European Molecular Biology Laboratory (EMBL), Heidelberg 69117, Germany.
9. Computational Biology Program, Ontario Institute for Cancer Research, Toronto, ON M5G 0A3, Canada.
10. Department of Molecular Genetics, University of Toronto, Toronto, ON M5S 1A8, Canada.
11. Biomolecular Engineering Department, University of California, Santa Cruz, Santa Cruz, CA 95064, USA.
12. King Faisal Specialist Hospital and Research Centre, Al Maather, Riyadh 12713, Saudi Arabia.
13. DLR Project Management Agency, Bonn 53227, Germany.
14. Genome Canada, Ottawa, ON K2P 1P1, Canada.
15. Instituto Carlos Slim de la Salud, Mexico City, Mexico.
16. Federal Ministry of Education and Research, Berlin 10117, Germany.
17. Institut Gustave Roussy, Villejuif 94805, France.
18. Institut National du Cancer (INCA), Boulogne-Billancourt 92100, France.
19. The Wellcome Trust, London NW1 2BE, UK.
20. Prostate Cancer Canada, Toronto, ON M5C 1M1, Canada.
21. National Human Genome Research Institute, National Institutes of Health, Bethesda, MD 20892, USA.
22. Department of Biotechnology, Ministry of Science & Technology, Government of India, New Delhi, Delhi 110003, India.
23. Science Writer, Garrett Park, MD 20896, USA.
24. International Cancer Genome Consortium (ICGC)/ICGC Accelerating Research in Genomic Oncology (ARGO) Secretariat, Toronto, ON M5G 0A3, Canada.
25. Adaptive Oncology Initiative, Ontario Institute for Cancer Research, Toronto, ON M5G 0A3, Canada.
26. Cancer Research UK, London EC1V 4AD, UK.
27. Department of Biochemistry, College of Medicine, Ewha Womans University, Seoul 07895, South Korea.
28. Chinese Cancer Genome Consortium, Shenzhen 518083, China.
29. Laboratory of Molecular Oncology, Beijing,100142, China.
30. Peking University Cancer Hospital & Institute, Key Laboratory of Carcinogenesis and Translational Research (Ministry of Education), Peking University Cancer Hospital & Institute, Beijing 100142, China.
31. National Cancer Center, Tokyo 104-0045, Japan.
32. German Cancer Aid, Bonn 53113, Germany.
33. Division of Cancer Genomics, National Cancer Center Research Institute, Tokyo 104-0045,

Japan.
34. Laboratory of Molecular Medicine, Human Genome Center, The Institute of Medical Science, The University of Tokyo, Minato-ku, Tokyo 108-8639, Japan.
35. Japan Agency for Medical Research and Development, Chiyoda-ku, Tokyo 100-0004 Japan.
36. Japan Agency for Medical Research and Development, Chiyoda-ku, Tokyo 100-0004, Japan.
37. Medical Oncology, University and Hospital Trust of Verona, Verona 37134, Italy.
38. University of Verona, Verona 37129, Italy.
39. BGI-Shenzhen, Shenzhen 518083, China.
40. National Cancer Institute, National Institutes of Health, Bethesda, MD 20892, USA.
41. Centre for Law and Genetics, University of Tasmania, Sandy Bay Campus, Hobart, Tasmania 7001 Australia.
42. Centre of Genomics and Policy, McGill University and Génome Québec Innovation Centre, Montreal, QC H3A 1A4, Canada.
43. Heidelberg Academy of Sciences and Humanities, Heidelberg 69120, Germany.
44. CAPHRI Research School, Maastricht University, Maastricht, ER 6200MD, The Netherlands.
45. Genome Informatics Program, Ontario Institute for Cancer Research, Toronto, ON M5G 0A3, Canada.
46. Barcelona Supercomputing Center (BSC), Barcelona 08034, Spain.
47. Laboratory for Medical Science Mathematics, RIKEN Center for Integrative Medical Sciences, Yokohama, Kanagawa 230-0045, Japan.
48. RIKEN Center for Integrative Medical Sciences, Yokohama, Kanagawa 230-0045, Japan.
49. Dana-Farber Cancer Institute, Boston, MA 02215, USA.
50. University of California Santa Cruz, Santa Cruz, CA 95064, USA.
51. Oregon Health and Science University, Portland, OR 97239, USA.
52. Division of Theoretical Bioinformatics, German Cancer Research Center (DKFZ), Heidelberg 69120, Germany.
53. Heidelberg Center for Personalized Oncology (DKFZ-HIPO), German Cancer Research Center, Heidelberg 69120, Germany.
54. Institute of Pharmacy and Molecular Biotechnology and BioQuant, Heidelberg University, Heidelberg 69120, Germany.
55. University of California San Diego, San Diego, CA 92093, USA.
56. PDXen Biosystems Inc, Seoul 4900, South Korea.
57. Electronics and Telecommunications Research Institute, Daejoen 34129, South Korea.
58. Seven Bridges Genomics, Charlestown, MA 02129, USA.
59. Annai Systems, Inc, Carlsbad, CA 92013, USA.
60. Department of Biomedical Data Science, Stanford University School of Medicine, Stanford, CA 94305, USA.
61. Department of Genetics, Stanford University School of Medicine, Stanford, CA 94305, USA.
62. Departments of Genetics and Biomedical Data Science, Stanford University School of Medicine, Stanford, CA 94305, USA.
63. University of Leuven, Leuven B-3000, Belgium.
64. The Francis Crick Institute, London NW1 1AT, UK.
65. The Hospital for Sick Children, Toronto, ON M5G 0A4, Canada.
66. Heidelberg University, Heidelberg 69120, Germany.

67. New BIH Digital Health Center, Berlin Institute of Health (BIH) and Charité - Universitätsmedizin Berlin, Berlin 10117, Germany.
68. Department of Biochemistry and Molecular Medicine, University of Montreal, Montreal, QC H3C 3J7, Canada.
69. CIBIO/InBIO - Research Center in Biodiversity and Genetic Resources, Universidade do Porto, Vairão 4485-601, Portugal.
70. Department Biochemistry and Molecular Biomedicine, University of Barcelona, Barcelona 08028, Spain.
71. University of Chicago, Chicago, IL 60637, USA.
72. Division of Biomedical Informatics, Department of Medicine, & Moores Cancer Center, UC San Diego School of Medicine, San Diego, CA 92093, USA.
73. Children's Hospital of Philadelphia, Philadelphia, PA 19146, USA.
74. Massachusetts General Hospital Center for Cancer Research, Charlestown, MA 02129, USA.
75. University of Melbourne Centre for Cancer Research, University of Melbourne, Melbourne, VIC 3010, Australia.
76. Syntekabio Inc, Daejon 34025, South Korea.
77. AbbVie, North Chicago, IL 60064, USA.
78. Genomics Research Program, Ontario Institute for Cancer Research, Toronto, ON M5G 0A3, Canada.
79. German Cancer Consortium (DKTK), Heidelberg 69120, Germany.
80. Heidelberg Center for Personalized Oncology (DKFZ-HIPO), German Cancer Research Center (DKFZ), Heidelberg 69120, Germany.
81. National Center for Tumor Diseases (NCT) Heidelberg, Heidelberg 69120, Germany.
82. Department of Pediatric Immunology, Hematology and Oncology, University Hospital, Heidelberg 69120, Germany.
83. German Cancer Research Center (DKFZ), Heidelberg 69120, Germany.
84. Heidelberg Institute for Stem Cell Technology and Experimental Medicine (HI-STEM), Heidelberg 69120, Germany.
85. Institute of Medical Science, University of Tokyo, Tokyo 108-8639, Japan.
86. The Institute of Medical Science, The University of Tokyo, Tokyo 108-8639, Japan.
87. Seven Bridges, Charlestown, MA 02129, USA.
88. Genome Integration Data Center, Syntekabio, Inc, Daejon, 34025, South Korea.
89. Computational Biology Center, Memorial Sloan Kettering Cancer Center, New York, NY 10065, USA.
90. ETH Zurich, Department of Biology, Zürich 8093, Switzerland.
91. ETH Zurich, Department of Computer Science, Zurich 8092, Switzerland.
92. SIB Swiss Institute of Bioinformatics, Lausanne 1015, Switzerland.
93. University Hospital Zurich, Zurich, 8091, Switzerland.
94. Health Sciences Department of Biomedical Informatics, University of California San Diego, La Jolla, CA 92093, USA.
95. Department of Health Sciences and Technology, Sungkyunkwan University School of Medicine, Seoul 06351, South Korea.
96. Samsung Genome Institute, Seoul 06351, South Korea.
97. Functional and Structural Genomics, German Cancer Research Center (DKFZ), Heidelberg

69120, Germany.
98. Leidos Biomedical Research, Inc, McLean, VA 22102, USA.
99. Sage Bionetworks, Seattle WA 98109, USA.
100. Genome Informatics, Ontario Institute for Cancer Research, Toronto, ON M5G 2C4, Canada.
101. Department of Cell and Systems Biology, University of Toronto, Toronto, ON M5S 3G5, Canada.
102. Department of Radiation Oncology, University of California San Francisco, San Francisco, CA 94518, USA.
103. CSRA Incorporated, Fairfax, VA 22042, USA.
104. Massachusetts General Hospital, Boston, MA 02114, USA.
105. Department of Biology, ETH Zurich, Zürich 8093, Switzerland.
106. Department of Computer Science, ETH Zurich, Zurich 8092, Switzerland.
107. University Hospital Zurich, Zurich 8091, Switzerland.
108. Weill Cornell Medical College, New York, NY 10065, USA.
109. Bioinformatics and Omics Data Analytics, German Cancer Research Center (DKFZ), Heidelberg 69120, Germany.
110. Institució Catalana de Recerca i Estudis Avançats (ICREA), Barcelona 08010, Spain.
111. Department of Clinical and Molecular Medicine, Faculty of Medicine and Health Sciences, Norwegian University of Science and Technology, Trondheim 7030, Norway.
112. Finsen Laboratory and Biotech Research & Innovation Centre (BRIC), University of Copenhagen, Copenhagen 2200, Denmark.
113. Department of Urology, Charité Universitätsmedizin Berlin, Berlin 10117, Germany.
114. Department of Biological Oceanography, Leibniz Institute of Baltic Sea Research, Seestraße 15, Rostock 18119, Germany.
115. Ontario Institute for Cancer Research, Toronto, ON M5G 0A3, Canada.
116. Department of Physiology and Biophysics, Weill Cornell Medicine, New York, NY 10065, USA.
117. Institute for Computational Biomedicine, Weill Cornell Medicine, New York, NY 10021, USA.
118. Division of Applied Bioinformatics, German Cancer Research Center (DKFZ), Heidelberg 69120, Germany.
119. Department of Computer Science, Yale University, New Haven, CT 06520, USA.
120. Department of Molecular Biophysics and Biochemistry, Yale University, New Haven, CT 06520, USA.
121. Program in Computational Biology and Bioinformatics, Yale University, New Haven, CT 06520, USA.
122. Department of Internal Medicine, Stanford University, Stanford, CA 94305, USA.
123. Department of Molecular Medicine (MOMA), Aarhus University Hospital, Aarhus N 8200, Denmark.
124. Clinical Bioinformatics, Swiss Institute of Bioinformatics, Geneva 1202, Switzerland.
125. Institute for Pathology and Molecular Pathology, University Hospital Zurich, Zurich 8091, Switzerland.
126. Institute of Molecular Life Sciences, University of Zurich, Zurich 8057, Switzerland.

127. MIT Computer Science and Artificial Intelligence Laboratory, Massachusetts Institute of Technology, Cambridge, MA 02139, USA.
128. Englander Institute for Precision Medicine, Weill Cornell Medicine, New York, NY 10065, USA.
129. Meyer Cancer Center, Weill Cornell Medicine, New York, NY 10065, USA.
130. Bioinformatics Research Centre (BiRC), Aarhus University, Aarhus 8000, Denmark.
131. Department of Medical Biophysics, University of Toronto, Toronto, ON M5S 1A8, Canada.
132. Institute of Molecular Life Sciences and Swiss Institute of Bioinformatics, University of Zurich, Zurich 8057, Switzerland.
133. CNAG-CRG, Centre for Genomic Regulation (CRG), Barcelona Institute of Science and Technology (BIST), Barcelona 08028, Spain.
134. Universitat Pompeu Fabra (UPF), Barcelona 08003, Spain.
135. Office of Cancer Genomics, National Cancer Institute, US National Institutes of Health, Bethesda, MD 20892, USA.
136. Alvin J. Siteman Cancer Center, Washington University School of Medicine, St Louis, MO 63110, USA.
137. The McDonnell Genome Institute at Washington University, St Louis, MO 63108, USA.
138. Computer Network Information Center,Chinese Academy of Sciences, Beijing 100190, China.
139. Center for Digital Health, Berlin Institute of Health and Charitè - Universitätsmedizin Berlin, Berlin 10117, Germany.
140. Department of Pharmacology, University of Toronto, Toronto, ON M5S 1A8, Canada.
141. University of California Los Angeles, Los Angeles, CA 90095, USA.
142. University of Texas MD Anderson Cancer Center, Houston, TX 77030, USA.
143. Department of Genetics and Informatics Institute, University of Alabama at Birmingham, Birmingham, AL 35294, USA.
144. Department of Genetics, Department of Medicine, Washington University in St Louis, St Louis, MO 63110, USA.
145. Centre for Genomic Regulation (CRG), The Barcelona Institute of Science and Technology, Barcelona 08003, Spain.
146. Department of Bioinformatics and Computational Biology, The University of Texas MD Anderson Cancer Center, Houston, TX 77030, USA.
147. Beijing Genomics Institute, Shenzhen 518083, China.
148. Department of Urologic Sciences, University of British Columbia, Vancouver, BC V5Z 1M9, Canada.
149. Vancouver Prostate Centre, Vancouver, BC V6H 3Z6, Canada.
150. Division of Life Science and Applied Genomics Center, Hong Kong University of Science and Technology, Clear Water Bay, Hong Kong, China.
151. Geneplus-Shenzhen, Shenzhen 518122, China.
152. School of Computer Science and Technology, Xi'an Jiaotong University, Xi'an 710048, China.
153. Biobyte solutions GmbH, Heidelberg 69126, Germany.
154. Division of Oncology, Washington University School of Medicine, St Louis, MO 63110, USA.
155. Institute of Medical Genetics and Applied Genomics, University of Tübingen, Tübingen

72074, Germany.
156. Indiana University, Bloomington, IN 47405, USA.
157. Simon Fraser University, Burnaby, BC V5A 1S6, Canada.
158. Department of Computer Science, University of Toronto, Toronto, ON M5S 1A8, Canada.
159. School of Electronic and Information Engineering, Xi'an Jiaotong University, Xi'an 710048, China.
160. Department of Genetics, Washington University School of Medicine, St Louis, MO 63110, USA.
161. Department of Mathematics, Washington University in St Louis, St Louis, MO 63130, USA.
162. Department of Molecular and Human Genetics, Baylor College of Medicine, Houston, TX 77030, USA.
163. Human Genome Sequencing Center, Baylor College of Medicine, Houston, TX 77030, USA.
164. The First Affiliated Hospital, Xi'an Jiaotong University, Xi'an 710049, China.
165. Department of Epidemiology and Biostatistics, Memorial Sloan Kettering Cancer Center, New York, NY 10065, USA.
166. The McDonnell Genome Institute at Washington University, Department of Genetics, Department of Medicine, Siteman Cancer Center, Washington University in St Louis, St Louis, MO 63108, USA.
167. Department of Genomic Medicine, The University of Texas MD Anderson Cancer Center, Houston, TX 77030, USA.
168. The Jackson Laboratory for Genomic Medicine, Farmington, CT 06032, USA.
169. Quantitative & Computational Biosciences Graduate Program, Baylor College of Medicine, Houston, TX 77030, USA.
170. Dana-Farber/Boston Children’s Cancer and Blood Disorders Center, Boston, MA 02215, USA.
171. Department of Pediatrics, Harvard Medical School, Boston, MA 02115, USA.
172. Department of Medical Oncology, Dana-Farber Cancer Institute, Boston, MA 02115, USA.
173. Department of Mathematics, Aarhus University, Aarhus 8000, Denmark.
174. Technical University of Denmark, Lyngby 2800, Denmark.
175. University of Copenhagen, Copenhagen 2200, Denmark.
176. Department for BioMedical Research, University of Bern, Bern 3008, Switzerland.
177. Department of Medical Oncology, Inselspital, University Hospital and University of Bern, Bern 3010, Switzerland.
178. Graduate School for Cellular and Biomedical Sciences, University of Bern, Bern 3012, Switzerland.
179. Department of Genitourinary Medical Oncology - Research, Division of Cancer Medicine, The University of Texas MD Anderson Cancer Center, Houston, TX 77030, USA.
180. Department of Urology, Icahn Sschool of Medicine at Mount Sinai, New York, NY 10029, USA.
181. Faculty of Biosciences, Heidelberg University, Heidelberg 69120, Germany.
182. Korea Advanced Institute of Science and Technology, Daejeon 34141, South Korea.
183. Institute for Research in Biomedicine (IRB Barcelona), The Barcelona Institute of Science and Technology, Barcelona 8003, Spain.
184. Research Program on Biomedical Informatics, Universitat Pompeu Fabra, Barcelona 08002,

Spain.
185. Science for Life Laboratory, Department of Cell and Molecular Biology, Uppsala University, Uppsala SE-75124, Sweden.
186. Queensland Centre for Medical Genomics, Institute for Molecular Bioscience, The University of Queensland, St Lucia, QLD 4072, Australia.
187. University of Milano Bicocca, Monza 20052, Italy.
188. Peter MacCallum Cancer Centre, Melbourne, VIC 3000, Australia.
189. Sir Peter MacCallum Department of Oncology, The University of Melbourne, Melbourne, VIC 3052, Australia.
190. Center for Precision Health, School of Biomedical Informatics, University of Texas Health Science Center, Houston, TX 77030, USA.
191. The Donnelly Centre, University of Toronto, Toronto, ON M5S 3E1, Canada.
192. Health Data Science Unit, University Clinics, Heidelberg 69120, Germany.
193. Department for Biomedical Research, University of Bern, Bern 3008, Switzerland.
194. Research Core Center, National Cancer Centre Korea, Goyang-si 410-769, South Korea.
195. Institute of Computer Science, Polish Academy of Sciences, Warsawa 01-248, Poland.
196. ETH Zurich, Department of Biology, Wolfgang-Pauli-Strasse 27, 8093 Zürich, Switzerland.
197. Harvard University, Cambridge, MA 02138, USA.
198. Memorial Sloan Kettering Cancer Center, New York, NY 10065, USA.
199. Department of Molecular Biophysics and Biochemistry, New Haven, CT 06520, USA.
200. Program in Computational Biology and Bioinformatics, New Haven, CT 06520, USA.
201. Yale University, New Haven, CT 06520, USA.
202. Department of Information Technology, Ghent University, Ghent B-9000, Belgium.
203. Department of Plant Biotechnology and Bioinformatics, Ghent University, Ghent B-9000, Belgium.
204. Yale School of Medicine, Yale University, New Haven, CT 06520, USA.
205. Institute for Research in Biomedicine (IRB Barcelona), Barcelona 08028, Spain.
206. Division of Hematology-Oncology, Samsung Medical Center, Sungkyunkwan University School of Medicine, Seoul, 06351, South Korea.
207. Samsung Advanced Institute for Health Sciences and Technology, Sungkyunkwan University School of Medicine, Seoul 06351, South Korea.
208. Cheonan Industry-Academic Collaboration Foundation, Sangmyung University, Cheonan 31066, South Korea.
209. Spanish National Cancer Research Centre, Madrid 28029, Spain.
210. Department of Computer Science, Princeton University, Princeton, NJ 08540, USA.
211. Bern Center for Precision Medicine, University Hospital of Bern, University of Bern, Bern 3008, Switzerland.
212. Englander Institute for Precision Medicine, Weill Cornell Medicine and NewYork Presbyterian Hospital, New York, NY 10021, USA.
213. Pathology and Laboratory, Weill Cornell Medical College, New York, NY 10021, USA.
214. Vall d'Hebron Institute of Oncology: VHIO, Barcelona 08035, Spain.
215. cBio Center, Dana-Farber Cancer Institute, Harvard Medical School, Boston, MA 02115, USA.
216. Department of Cell Biology, Harvard Medical School, Boston, MA 02115, USA.

217. Department of Cancer Biology, Dana-Farber Cancer Institute, Boston, MA 02215, USA.
218. Peter MacCallum Cancer Centre and University of Melbourne, Melbourne, VIC 3000, Australia.
219. cBio Center, Dana-Farber Cancer Institute, Boston, MA 02215, USA.
220. CREST, Japan Science and Technology Agency, Tokyo 113-0033, Japan.
221. Department of Medical Science Mathematics, Medical Research Institute, Tokyo Medical and Dental University, Bunkyo-ku, Tokyo 113-8510, Japan.
222. Laboratory for Medical Science Mathematics, Department of Biological Sciences, Graduate School of Science, The University of Tokyo, Bunkyo-ku, Tokyo 113-0033, Japan.
223. Science for Life Laboratory, Department of Oncology-Pathology, Karolinska Institutet, Stockholm 17121, Sweden.
224. Department of Gene Technology, Tallinn University of Technology, Tallinn 12616, Estonia.
225. Genetics & Genome Biology Program, SickKids Research Institute, The Hospital for Sick Children, Toronto, ON M5G 1X8, Canada.
226. Department of Information Technology, Ghent University, Interuniversitair Micro-Electronica Centrum (IMEC), Ghent B-9000, Belgium.
227. Science for Life Laboratory, Department of Immunology, Genetics and Pathology, Uppsala University, Uppsala SE-75108, Sweden.
228. Oregon Health & Sciences University, Portland, OR 97239, USA.
229. Department of Medicine and Therapeutics, The Chinese University of Hong Kong, Shatin, NT, Hong Kong, China SAR.
230. The University of Texas Health Science Center at Houston, Houston, TX 77030, USA.
231. Department of Biomedical Informatics, College of Medicine, The Ohio State University, Columbus, OH 43210, USA.
232. The Ohio State University Comprehensive Cancer Center (OSUCCC – James), Columbus, OH 43210, USA.
233. School of Biomedical Informatics, The University of Texas Health Science Center at Houston, Houston, TX 77030, USA.
234. Department of Biochemistry and Molecular Genetics, Feinberg School of Medicine, Northwestern University, Chicago, IL 60637, USA.
235. University of Glasgow, CRUK Beatson Institute for Cancer Research, Bearsden, Glasgow G61 1BD, UK.
236. Centre for Molecular Science Informatics, Department of Chemistry, University of Cambridge, Cambridge CB2 1EW, UK.
237. Department of Biomedical Informatics, Harvard Medical School, Boston, MA 02115, USA.
238. Ludwig Center, Harvard Medical School, Boston, MA 02115, USA.
239. UC Santa Cruz Genomics Institute, University of California Santa Cruz, Santa Cruz, CA 95064, USA.
240. Massachusetts General Hospital, Physics Division, Optimization and Systems Biology Lab, Boston, MA 02114, USA.
241. Department of Medicine, Baylor College of Medicine, Houston, TX 77030, USA.
242. Computational and Systems Biology, Genome Institute of Singapore, Singapore 138672, Singapore.
243. School of Computing, National University of Singapore, Singapore 117417, Singapore.

244. The Azrieli Faculty of Medicine, Bar-Ilan University, Safed 13195, Israel.
245. National Cancer Centre Singapore, Singapore 169610, Singapore.
246. Peking University, Beijing 100871, China.
247. Department of Genetics, University of North Carolina at Chapel Hill, Chapel Hill, NC 27599, USA.
248. Lineberger Comprehensive Cancer Center, University of North Carolina at Chapel Hill, Chapel Hill, NC 27599, USA.
249. China National GeneBank-Shenzhen, Shenzhen 518083, China.
250. Berlin Institute for Medical Systems Biology, Max Delbrück Center for Molecular Medicine, Berlin 13125, Germany.
251. University College London, London WC1E 6BT, UK.
252. School of Life Sciences, Peking University, Beijing 100180, China.
253. Department of Pathology, The University of Melbourne, Melbourne, VIC 3052, Australia.
254. Genome Institute of Singapore, Singapore 138672, Singapore.
255. Department of Epidemiology, University of Alabama at Birmingham, Birmingham, AL 35294, USA.
256. HudsonAlpha Institute for Biotechnology, Huntsville, AL 35806, USA.
257. O'Neal Comprehensive Cancer Center, University of Alabama at Birmingham, Birmingham, AL 35294, USA.
258. Department of Biosciences and Nutrition, Karolinska Institutet, Stockholm 14183, Sweden.
259. Ludwig Center at Harvard, Boston, MA 02115, USA.
260. German Cancer Consortium (DKTK), Partner site Berlin.
261. Human Genetics, University of Kiel, Kiel 24118, Germany.
262. Institute of Human Genetics, Ulm University and Ulm University Medical Center, Ulm 89081, Germany.
263. Computational & Systems Biology Program, Memorial Sloan Kettering Cancer Center, New York, NY 10065, USA.
264. Korea University, Seoul 02481, South Korea.
265. Division of Computational Genomics and Systems Genetics, German Cancer Research Center (DKFZ), Heidelberg 69120, Germany.
266. Cancer Science Institute of Singapore, National University of Singapore, Singapore 169609, Singapore.
267. Programme in Cancer & Stem Cell Biology, Duke-NUS Medical School, Singapore 169857, Singapore.
268. SingHealth, Duke-NUS Institute of Precision Medicine, National Heart Centre Singapore, Singapore 169609, Singapore.
269. Institute of Molecular and Cell Biology, Singapore 169609, Singapore.
270. Laboratory of Cancer Epigenome, Division of Medical Science, National Cancer Centre Singapore, Singapore 169610, Singapore.
271. BIOPIC, ICG and College of Life Sciences, Peking University, Beijing 100871, China.
272. Genome Science Division, Research Center for Advanced Science and Technology, The University of Tokyo, Tokyo 153-8904, Japan.
273. Center for Bioinformatics and Functional Genomics, Cedars-Sinai Medical Center, Los Angeles, CA 90048, USA.

274. Department of Biomedical Sciences, Cedars-Sinai Medical Center, Los Angeles, CA 90048, USA.
275. The Hebrew University Faculty of Medicine, Jerusalem 91120, Israel.
276. Bioinformatics Group, Department of Computer Science, University of Leipzig, Leipzig 04109, Germany.
277. Interdisciplinary Center for Bioinformatics, University of Leipzig, Leipzig 04109, Germany.
278. German Cancer Consortium (DKTK), German Cancer Research Center (DKFZ), Heidelberg 69120, Germany.
279. Computational Biology, Leibniz Institute on Aging - Fritz Lipmann Institute (FLI), Jena 07745, Germany.
280. Transcriptome Bioinformatics, LIFE Research Center for Civilization Diseases, University of Leipzig, Leipzig 04109, Germany.
281. Center for Epigenetics, Van Andel Research Institute, Grand Rapids, MI 49503, USA.
282. Institut d'Investigacions Biomèdiques August Pi i Sunyer (IDIBAPS), Barcelona 08036, Spain.
283. Research Center for Advanced Science and Technology, The University of Tokyo, Minato-ku, Tokyo 108-8639, Japan.
284. Cancer Epigenomics, German Cancer Research Center (DKFZ), Heidelberg 69120, Germany.
285. Van Andel Research Institute, Grand Rapids, MI 49503, USA.
286. Department of Zoology, Genetics and Physical Anthropology, Universidade de Santiago de Compostela, Santiago de Compostela 15706, Spain.
287. Centre for Research in Molecular Medicine and Chronic Diseases (CIMUS), Universidade de Santiago de Compostela, Santiago de Compostela 15706, Spain.
288. The Biomedical Research Centre (CINBIO), Universidade de Vigo, Vigo 36310, Spain.
289. Transmissible Cancer Group, Department of Veterinary Medicine, University of Cambridge, Cambridge CB3 0ES, UK.
290. Sir Peter MacCallum Department of Oncology, University of Melbourne, Melbourne, VIC 3052, Australia.
291. Johns Hopkins School of Medicine, Baltimore, MD 21205, USA.
292. Foundation Medicine, Inc, Cambridge, MA 02141, USA.
293. University of Ottawa Faculty of Medicine, Department of Biochemistry, Microbiology and Immunology, Ottawa, ON K1H 8M5, Canada.
294. Cancer Research UK Cambridge Institute, University of Cambridge, Cambridge CB2 0RE, UK.
295. University of Cambridge, Cambridge CB2 1TN, UK.
296. Quantitative Genomics Laboratories (qGenomics), Barcelona 08950, Spain.
297. Genome Integrity and Structural Biology Laboratory, National Institute of Environmental Health Sciences (NIEHS), Durham, NC 27709, USA.
298. Brandeis University, Waltham, MA 02254, USA.
299. New York Genome Center, New York, NY 10013, USA.
300. Weill Cornell Medicine, New York, NY 10065, USA.
301. Hopp Children's Cancer Center (KiTZ), Heidelberg 69120, Germany.
302. Pediatric Glioma Research Group, German Cancer Research Center (DKFZ), Heidelberg 69120, Germany.
303. Skolkovo Institute of Science and Technology, Moscow 121205, Russia.

304. A.A.Kharkevich Institute of Information Transmission Problems, Moscow 127051, Russia.
305. Dmitry Rogachev National Research Center of Pediatric Hematology, Oncology and Immunology, Moscow, 117997, Russia.
306. Integrative Bioinformatics Support Group, National Institute of Environmental Health Sciences (NIEHS), Durham, NC 27709, USA.
307. Center For Medical Innovation, Seoul National University Hospital, Seoul 03080, South Korea.
308. Department of Internal Medicine, Seoul National University Hospital, Seoul 03080, South Korea.
309. Division of Genetics and Genomics, Boston Children's Hospital and Harvard Medical School, Boston, MA 02115, USA.
310. School of Medicine/School of Mathematics and Statistics, University of St Andrews, St Andrews, Fife KY16 9SS, UK.
311. Department of Genetics and Computational Biology, QIMR Berghofer Medical Research Institute, Brisbane 4006, Australia.
312. Institute for Molecular Bioscience, University of Queensland, St Lucia, Brisbane, QLD 4072, Australia.
313. School of Molecular Biosciences and Center for Reproductive Biology, Washington State University, Pullman, WA 99164, USA.
314. Cancer Research Institute, Beth Israel Deaconess Medical Center, Boston, MA 02215, USA.
315. Ben May Department for Cancer Research, Department of Human Genetics, The University of Chicago, Chicago, IL 60637, USA.
316. Tri-institutional PhD program of computational biology and medicine, Weill Cornell Medicine, New York, NY 10065, USA.
317. Department of Cellular and Molecular Medicine and Department of Bioengineering and Moores Cancer Center, University of California San Diego, La Jolla, California 92093, USA.
318. Department of Cellular and Molecular Medicine and Department of Bioengineering and Moores Cancer Center, University of California, San Diego, La Jolla, California 92093, USA.
319. Centre for Computational Biology, Duke-NUS Medical School, Singapore 169857, Singapore.
320. Department of Computer Science, University of Helsinki, Helsinki 00014, Finland.
321. Institute of Biotechnology, University of Helsinki, Helsinki 00014, Finland.
322. Organismal and Evolutionary Biology Research Programme, University of Helsinki, Helsinki 00014, Finland.
323. Programme in Cancer & Stem Cell Biology, Centre for Computational Biology, Duke-NUS Medical School, Singapore 169857, Singapore.
324. Academic Department of Medical Genetics, University of Cambridge, Addenbrooke's Hospital, Cambridge CB2 0QQ, UK.
325. MRC Cancer Unit, University of Cambridge, Cambridge CB2 0XZ, UK.
326. The University of Cambridge School of Clinical Medicine, Cambridge CB2 0SP, UK.
327. National Centre for Biological Sciences, Tata Institute of Fundamental Research, Bangalore 560065, India.
328. Department of Applied Mathematics and Theoretical Physics, Centre for Mathematical Sciences, University of Cambridge, Cambridge CB3 0WA, UK.

329. Department of Statistics, Columbia University, New York, NY 10027, USA.
330. Duke-NUS Medical School, Singapore 169857, Singapore.
331. Faculty of Medicine and Health Technology, Tampere University and Tays Cancer Center, Tampere University Hospital, Tampere FI-33014, Finland.
332. Bakar Computational Health Sciences Institute and Department of Pediatrics, University of California, San Francisco, CA 94158-2549, USA.
333. Division of Cancer Epidemiology and Genetics, National Cancer Institute, National Institutes of Health, Bethesda, MD 20892, USA.
334. Department of Biostatistics, Bloomberg School of Public Health, Johns Hopkins University, Baltimore, MD 21287, USA.
335. Department of Oncology, School of Medicine, Johns Hopkins University, Baltimore, MD 21230, USA.
336. Integrated Graduate Program in Physical and Engineering Biology, Yale University, New Haven, CT 06520, USA.
337. Department of Computational Biology, University of Lausanne, Lausanne 1015, Switzerland.
338. Department of Genetic Medicine and Development, University of Geneva Medical School, Geneva CH1211, Switzerland.
339. Swiss Institute of Bioinformatics, University of Geneva, Geneva CH1211, Switzerland.
340. Institute of Medical Genetics and Applied Genomics, University of Tübingen, Tübingen 72076, Germany.
341. Independent Consultant, Wellesley 02481, USA.
342. Centre for Cancer Genetic Epidemiology, Department of Oncology, University of Cambridge, Cambridge CB1 8RN, UK.
343. Centre for Cancer Genetic Epidemiology, Department of Public Health and Primary Care, University of Cambridge, Cambridge CB1 8RN, UK.
344. CIBER Epidemiología y Salud Pública (CIBERESP), Madrid 28029, Spain.
345. Research Group on Statistics, Econometrics and Health (GRECS), UdG, Barcelona 8041, Spain.
346. Oxford Nanopore Technologies, New York, NY 10013, USA.
347. Department of Medical Genetics, College of Medicine, Hallym University, Chunchoen 24252, South Korea.
348. Institute of Evolutionary Biology (UPF-CSIC), Department of Experimental and Health Sciences, Universitat Pompeu Fabra, Barcelona 08003, Spain.
349. Icahn School of Medicine at Mount Sinai, New York, NY 10029, USA.
350. Laboratory of Translational Genomics, Division of Cancer Epidemiology and Genetics, National Cancer Institute, National Institutes of Health, Bethesda, MD 20892, USA.
351. Institut Català de Paleontologia Miquel Crusafont, Universitat Autònoma de Barcelona, Barcelona 08193, Spain.
352. Applications Department, Oxford Nanopore Technologies, Oxford OX4 4DQ, UK.
353. Institut de Recerca Sant Joan de Déu; Institut de Biomedicina de la Universitat de Barcelona (IBUB) & Department of Genetics, Microbiology & Statistics, Faculty of Biology, University of Barcelona, Barcelona 08028, Spain.
354. Institute for Research in Biomedicine (IRB Barcelona), The Barcelona Institute of Science

and Technology, Barcelona 08028, Spain.
355. Department of Ophthalmology and Ocular Genomics Institute, Massachusetts Eye and Ear, Harvard Medical School, Boston, MA 02114, USA.
356. Department of Medical and Clinical Genetics, Genome-Scale Biology Research Program, University of Helsinki, Helsinki 00100, Finland.
357. Big Data Institute, Li Ka Shing Centre, University of Oxford, Oxford OX3 7LF, UK.
358. Oxford NIHR Biomedical Research Centre, University of Oxford, Oxford OX4 2PG, UK.
359. School of Electronic Information and Communications, Huazhong University of Science and Technology, Wuhan, Hubei 430074, China.
360. Bioinformatics Unit, Spanish National Cancer Research Centre (CNIO), Madrid 28029, Spain.
361. Vector Institute, Toronto, ON M5G 0A3, Canada.
362. South Western Sydney Clinical School, Faculty of Medicine, University of NSW, Liverpool, NSW 2170, Australia.
363. The Kinghorn Cancer Centre, Cancer Division, Garvan Institute of Medical Research, University of NSW, Sydney, NSW 2010, Australia.
364. West of Scotland Pancreatic Unit, Glasgow Royal Infirmary, Glasgow G31 2ER, UK.
365. Wolfson Wohl Cancer Research Centre, Institute of Cancer Sciences, University of Glasgow, Bearsden, Glasgow G61 1QH, UK.
366. University of Melbourne Centre for Cancer Research, The University of Melbourne, Melbourne, VIC 3052, Australia.
367. MRC Human Genetics Unit, MRC IGMM, University of Edinburgh, Edinburgh EH4 2XU, UK.
368. Bioinformatics Group, Division of Molecular Biology, Department of Biology, Faculty of Science, University of Zagreb, Zagreb 10000, Croatia.
369. Department of Bioinformatics, Research Institute, National Cancer Center Japan, Tokyo 104-0045, Japan.
370. Departments of Pathology, Genomic Medicine, and Translational Molecular Pathology, The University of Texas MD Anderson Cancer Center, Houston, TX 77030, USA.
371. Oregon Health & Science University, Portland, OR 97239, USA.
372. University of Glasgow, Glasgow G61 1BD, UK.
373. MRC-University of Glasgow Centre for Virus Research, Glasgow G61 1QH, UK.
374. Wolfson Wohl Cancer Research Centre, Institute of Cancer Sciences, University of Glasgow, Bearsden G61 1QH, United Kingdom.
375. School of Computing Science, University of Glasgow, Glasgow G12 8RZ, UK.
376. Molecular and Medical Genetics, Oregon Health and Science University, Portland, OR 97201, USA.
377. Department of Surgery, University of Melbourne, Parkville VIC 3010, Australia.
378. The Murdoch Children's Research Institute, Royal Children's Hospital, Parkville, VIC 3052, Australia.
379. Walter + Eliza Hall Institute, Parkville, VIC 3052, Australia.
380. University of Cologne, Cologne 50931, Germany.
381. The Edward S. Rogers Sr. Department of Electrical and Computer Engineering, University of Toronto, Toronto, ON M5S 3G4, Canada.
382. University of Ljubljana, Ljubljana 1000, Slovenia.

383. Research Institute, NorthShore University HealthSystem, Evanston, IL 60201, USA.
384. Department of Public Health Sciences, The University of Chicago, Chicago IL 60637.
385. Department of Statistics, University of California Santa Cruz, Santa Cruz, CA 95064, USA.
386. Cambridge University Hospitals NHS Foundation Trust, Cambridge CB2 0QQ, UK.
387. University of Toronto, Toronto, ON M5G 2M9, Canada.
388. Department of Computer Science, Carleton College, Northfield, MN 55057, USA.
389. Molecular and Medical Genetics, Oregon Health & Science University, Portland, OR 97239, USA.
390. Center for Psychiatric Genetics, NorthShore University HealthSystem, Evanston, IL 60201, USA.
391. Molecular and Medical Genetics, Knight Cancer Institute, Oregon Health & Science University, Portland, OR 97219, USA.
392. Argmix Consulting, North Vancouver BC V7M 2J5, Canada.
393. Department of Computer Science, University of Toronto, Toronto, ON M5S 2E4, Canada.
394. Department of Biostatistics, The University of Texas MD Anderson Cancer Center, Houston, TX 77030, USA.
395. Department of Biostatistics, University of North Carolina at Chapel Hill, Chapel Hill, NC 27599, USA.
396. The University of Texas MD Anderson Cancer Center, Houston, TX 77030, USA.
397. Howard Hughes Medical Institute, University of California Santa Cruz, Santa Cruz, CA 95065, USA.
398. Cancer Unit, MRC University of Cambridge, Cambridge CB2 0XZ, UK.
399. Department of Bioinformatics and Computational Biology and Department of Systems Biology, The University of Texas MD Anderson Cancer Center, Houston, TX 77030, USA.
400. Department of Bioinformatics and Computational Biology, Department of Systems Biology, The University of Texas MD Anderson Cancer Center, Houston, TX 77030, USA.
401. Department of Health Sciences, Faculty of Medical Sciences, Kyushu University, Fukuoka 812-8582, Japan.
402. Baylor College of Medicine, Houston, TX 77030, USA.
403. Department of Applied Mathematics and Statistics, Johns Hopkins University, Baltimore, MD 21218, USA.
404. Heinrich Pette Institute, Leibniz Institute for Experimental Virology, Hamburg 20251, Germany.
405. University Medical Center Hamburg-Eppendorf, Bioinformatics Core, Hamburg 20246, Germany.
406. Earlham Institute, Norwich NR4 7UZ, UK.
407. Norwich Medical School, University of East Anglia, Norwich NR4 7TJ, UK.
408. The Institute of Cancer Research, London SW7 3RP, UK.
409. University of East Anglia, Norwich NR4 7TJ, UK.
410. German Center for Infection Research (DZIF), Partner Site Hamburg-Borstel-Lübeck-Riems, Hamburg, Germany.
411. Division of Molecular Genetics, German Cancer Research Center (DKFZ), Heidelberg 69120, Germany.
412. Peter MacCallum Cancer Centre, Melbourne, Victoria 3000, Australia.

413. Sir Peter MacCallum Department of Oncology, The University of Melbourne, Melbourne VIC 3000, Australia.
414. QIMR Berghofer Medical Research Institute, Brisbane, QLD 4006, Australia.
415. Victorian Institute of Forensic Medicine, Southbank, Victoria 3006, Australia.
416. University of Pennsylvania, Philadelphia, PA 19104, USA.
417. Peter MacCallum Cancer Centre, Melbourne VIC 3000, Australia.
418. Centre for Cancer Research, The Westmead Institute for Medical Research, Sydney 2145, Australia.
419. Department of Gynaecological Oncology, Westmead Hospital, Sydney 2145, Australia.
420. Genetics and Molecular Pathology, SA Pathology, Adelaide, SA 5000, Australia.
421. Centre for Cancer Research, The Westmead Institute for Medical Research, The University of Sydney, Sydney, NSW 2145, Australia.
422. Department of Gynaecological Oncology, Westmead Hospital, Sydney, NSW 2006, Australia.
423. Garvan Institute of Medical Research, Darlinghurst, NSW 2010 Australia.
424. Centre for Cancer Research, The Westmead Institute for Medical Research, and Department of Gynaecological Oncology, Westmead Hospital, Sydney, NSW 2145, Australia.
425. The University of Sydney, Sydney, NSW 2006, Australia.
426. The Westmead Institute for Medical Research. The University of Sydney. The Department of Gynaecological Oncology, Westmead Hospital, Westmead, NSW 2145, Australia.
427. Department of Surgery, Pancreas Institute, University and Hospital Trust of Verona, Verona 37134, Italy.
428. Department of Surgery, Princess Alexandra Hospital, Woolloongabba QLD 4102, Australia.
429. Surgical Oncology Group, Diamantina Institute, The University of Queensland, Woolloongabba, Brisbane, QLD 4102, Australia.
430. Department of Diagnostics and Public Health, University and Hospital Trust of Verona, Verona 37134, Italy.
431. ARC-Net Centre for Applied Research on Cancer, University and Hospital Trust of Verona, Verona 37134, Italy.
432. Illawarra Shoalhaven Local Health District L3 Illawarra Cancer Care Centre, Wollongong Hospital, Wollongong NSW 2500, Australia.
433. University of Sydney, Sydney NSW 2006, Australia.
434. School of Biological Sciences, The University of Auckland, Auckland 1010, New Zealand.
435. Department of Pathology and Diagnostics, University and Hospital Trust of Verona, Verona 37134, Italy.
436. Department of Medicine, Section of Endocrinology, University and Hospital Trust of Verona, Verona 37134, Italy.
437. Department of Pathology, Queen Elizabeth University Hospital, Glasgow G51 4TF, UK.
438. Wolfson Wohl Cancer Research Centre, Bearsden, Glasgow G61 1QH, UK.
439. University of Sydney, Sydney, NSW 2006, Australia.
440. Department of Medical Oncology, Beatson West of Scotland Cancer Centre, Glasgow G12 0YN, UK.
441. Academic Unit of Surgery, School of Medicine, College of Medical, Veterinary and Life Sciences, University of Glasgow, Glasgow Royal Infirmary, Glasgow G4 OSF, UK.

442. Tissue Pathology and Diagnostic Oncology, Royal Prince Alfred Hospital, Camperdown, NSW 2050, Australia.
443. Discipline of Surgery, Western Sydney University, Penrith NSW 2751, Australia.
444. Institute of Cancer Sciences, College of Medical Veterinary and Life Sciences, University of Glasgow, Glasgow G12 8QQ, UK.
445. The Kinghorn Cancer Centre, Cancer Division, Garvan Institute of Medical Research, University of NSW, Sydney, NSW 2109, Australia.
446. School of Environmental and Life Sciences, Faculty of Science, The University of Newcastle, Ourimbah, NSW 2258, Australia.
447. School of Surgery M507, University of Western Australia, Nedlands 6009, Australia.
448. Applied Tumor Genomics Research Program, Research Programs Unit, University of Helsinki, Helsinki 00290, Finland.
449. Olivia Newton-John Cancer Research Institute, La Trobe University, Heidelberg, Victoria 3084, Australia.
450. Melanoma Institute Australia, The University of Sydney, Wollstonecraft NSW 2065, Australia.
451. Children's Hospital at Westmead, The University of Sydney, Westmead, NSW 2145, Australia.
452. Melanoma Institute Australia, The University of Sydney, Sydney 2065, Australia.
453. Australian Institute of Tropical Health and Medicine, James Cook University, Douglas QLD 4814, Australia.
454. Bioplatforms Australia, North Ryde, NSW 2109, Australia.
455. Melanoma Institute Australia, Macquarie University, Wollstonecraft NSW, 2109, Australia.
456. Children's Medical Research Institute, Westmead, NSW 2145 Australia.
457. Melanoma Institute Australia, The University of Sydney, Wollstonecraft 2065, NSW, Australia.
458. Westmead Institute for Medical Research, University of Sydney, Westmead, NSW 2145 Australia.
459. Melanoma Institute Australia, The University of Sydney, Wollstonecraft, NSW 2065, Australia.
460. Centre for Cancer Research, The Westmead Millennium Institute for Medical Research, University of Sydney, Westmead Hospital, Westmead NSW 2145, Australia.
461. Centre for Cancer Research, Westmead Institute for Medical Research, Westmead, NSW 2145, Australia.
462. Discipline of Pathology, Sydney Medical School, The University of Sydney, Sydney 2065, Australia.
463. Royal Prince Alfred Hospital, Sydney, NSW 2050, Australia.
464. Bioplatforms Australia, North Ryde, NSW 2109 Australia.
465. School of Mathematics and Statistics, The University of Sydney, Sydney, NSW 2006 Australia.
466. Diagnostic Development, Ontario Institute for Cancer Research, Toronto, ON M5G 0A3, Canada.
467. Ontario Tumour Bank, Ontario Institute for Cancer Research, Toronto, ON M5G 0A3, Canada.

468. PanCuRx Translational Research Initiative, Ontario Institute for Cancer Research, Toronto, ON M5G 0A3, Canada.
469. BioSpecimen Sciences Program, University Health Network, Toronto, ON M5G 2C4, Canada, Toronto, ON M5G 2C4, Canada.
470. Hepatobiliary/Pancreatic Surgical Oncology Program, University Health Network, Toronto, ON M5G 2C4, Canada.
471. Hepatobiliary/pancreatic Surgical Oncology Program, University Health Network, Toronto, ON M5G 2C4, Canada.
472. Lunenfeld-Tanenbaum Research Institute, Mount Sinai Hospital, Toronto, ON M5G 1X5, Canada.
473. Genomics, Ontario Institute for Cancer Research, Toronto, ON M5G 0A3, Canada.
474. Division of Medical Oncology, Princess Margaret Cancer Centre, Toronto, ON M5G 2M9, Canada.
475. Lunenfeld-Tanenbaum Research Institute, Toronto, ON M5G 1X5, Canada.
476. University of Nebraska Medical Centre, Omaha, NE 68198, USA.
477. BioSpecimen Sciences Program, University Health Network, Toronto, ON M5G 2C4, Canada.
478. Transformative Pathology, Ontario Institute for Cancer Research, Toronto, ON M5G 0A3, Canada.
479. Department of Biochemistry and Molecular Medicine, University California at Davis, Sacramento, CA 95817 USA.
480. University Health Network, Princess Margaret Cancer Centre, Toronto, ON M5G 1L7, Canada.
481. Department of Health Sciences Research, Mayo Clinic, Rochester, MN 55905, USA.
482. Department of Pathology, Human Oncology and Pathogenesis Program, Memorial Sloan Kettering Cancer Center, New York, NY 10053, USA.
483. Department of Laboratory Medicine and Pathobiology, University of Toronto, Toronto, ON M5S 1A8, Canada.
484. BioSpecimen Sciences, Laboratory Medicine (Toronto), Medical Biophysics, PanCuRX, Toronto, ON M5S 1A8, Canada.
485. University of Nebraska Medical Center, Omaha, NE 68198-6880, USA.
486. Department of Medical Biophysics, University of Toronto, Toronto, ON M5G 1L7, Canada.
487. Human Longevity Inc, San Diego, CA 92121, USA.
488. CRUK Manchester Institute and Centre, Manchester M204GJ, UK.
489. Department of Radiation Oncology, University of Toronto, Toronto, ON M5S 1A8, Canada.
490. Manchester Cancer Research Centre, Cancer Division, FBMH, University of Manchester, Manchester M204GJ, UK.
491. Radiation Medicine Program, Princess Margaret Cancer Centre, Toronto, ON M5G 2M9, Canada.
492. Department of Surgical Oncology, Princess Margaret Cancer Centre, Toronto, ON M5G 2M9, Canada.
493. Genome Informatics Program, Ontario Institute for Cancer Research, Toronto, ON M5G 2C4, Canada.
494. STTARR Innovation Facility, Princess Margaret Cancer Centre, Toronto, ON M5G 1L7,

Canada.
495. Department of Pathology, Toronto General Hospital, Toronto, ON M5G 2C4, Canada.
496. Hefei University of Technology, Anhui 230009, China.
497. State key Laboratory of Cancer Biology, and Xijing Hospital of Digestive Diseases, Fourth Military Medical University, Shaanxi 710032, China.
498. Fourth Military Medical University, Shaanxi 710032, China.
499. Peking University Cancer Hospital & Institute, Beijing 100142, China.
500. Department of Surgery, Ruijin Hospital, Shanghai Jiaotong University School of Medicine, Shanghai, 200025, China.
501. Leeds Institute of Medical Research, University of Leeds, St James's University Hospital, Leeds LS9 7TF, UK.
502. Canadian Center for Computional Genomics, McGill University, Montreal, QC H3A 0G1, Canada.
503. Department of Human Genetics, McGill University, Montreal, QC H3A 1B1, Canada.
504. International Agency for Research on Cancer, Lyon 69008, France.
505. McGill University and Genome Quebec Innovation Centre, Montreal, QC H3A 0G1, Canada.
506. Centre National de Génotypage, CEA - Institute de Génomique, Evry 91000, France.
507. Leeds Institute of Medical Research @ St James's, University of Leeds, St James's University Hospital, Leeds LS9 7TF, UK.
508. Institute of Mathematics and Computer Science, University of Latvia, Riga LV1459, Latvia.
509. Department of Oncology, Gil Medical Center, Gachon University, Incheon, South Korea.
510. Department of Molecular Oncology, BC Cancer Agency, Vancouver, BC V5Z 1L3, Canada.
511. Los Alamos National Laboratory, Los Alamos, NM 87545, USA.
512. Department of Genetics, Institute for Cancer Research, Oslo University Hospital, The Norwegian Radium Hospital, Oslo O310, Norway.
513. Lund University, Lund 223 62, Sweden.
514. Translational Research Lab, Centre Léon Bérard, Lyon 69373, France.
515. Radboud University, Department of Molecular Biology, Faculty of Science, Nijmegen Centre for Molecular Life Sciences, Nijmegen 6500 HB, The Netherlands.
516. Department of Pathology, Brigham and Women's Hospital, Harvard Medical School, Boston, MA 02115, USA.
517. Department Experimental Therapy, The Netherlands Cancer Institute, Amsterdam 1066 CX, The Netherlands.
518. Cancer Research UK Cambridge Institute, University of Cambridge, Li Ka Shing Centre, Cambridge CB2 0RE, UK.
519. Department of Oncology, University of Cambridge, Cambridge CB2 1TN, UK.
520. Breast Cancer Translational Research Laboratory JC Heuson, Institut Jules Bordet, Brussels 1000, Belgium.
521. Translational Cancer Research Unit, Center for Oncological Research, Faculty of Medicine and Health Sciences, University of Antwerp, Antwerp 2000, Belgium.
522. Department of Gynecology & Obstetrics, Department of Clinical Sciences, Skåne University Hospital, Lund University, Lund SE-221 85, Sweden.
523. Icelandic Cancer Registry, Icelandic Cancer Society, Reykjavik 125, Iceland.
524. Translational Cancer Research Unit, GZA Hospitals St.-Augustinus, Antwerp 2000, Belgium.

525. Department of Pathology, Memorial Sloan Kettering Cancer Center, New York, NY 10065, USA.
526. Department of Medical Oncology, Josephine Nefkens Institute and Cancer Genomics Centre, Erasmus Medical Center, Rotterdam 3015CN, The Netherlands.
527. National Genotyping Center, Institute of Biomedical Sciences, Academia Sinica, Taipei 115, Taiwan.
528. Department of Pathology, Oslo University Hospital Ulleval, Oslo 0450, Norway.
529. Faculty of Medicine and Institute of Clinical Medicine, University of Oslo, Oslo NO-0316, Norway.
530. Department of Pathology, Skåne University Hospital, Lund University, Lund SE-221 85, Sweden.
531. Department of Pathology, Academic Medical Center, Amsterdam 1105 AZ, The Netherlands.
532. Department of Pathology, College of Medicine, Hanyang University, Seoul 133-791, South Korea.
533. Department of Pathology, Asan Medical Center, College of Medicine, Ulsan University, Songpa-gu, Seoul 05505, South Korea.
534. The Netherlands Cancer Institute, Amsterdam 1066 CX, The Netherlands.
535. Department of Surgery, Memorial Sloan-Kettering Cancer Center, New York, NY 10065, USA.
536. Department of Surgery, Brigham and Women’s Hospital/Dana Farber Cancer Institute, Boston, MA 02115, USA.
537. Department of Clinical Science, University of Bergen, Bergen 5020, Norway.
538. Morgan Welch Inflammatory Breast Cancer Research Program and Clinic, The University of Texas MD Anderson Cancer Center, Houston, TX 77030, USA.
539. The University of Queensland Centre for Clinical Research, The Royal Brisbane & Women's Hospital, Herston, QLD 4029, Australia.
540. Department of Pathology, Jules Bordet Institute, Brussels 1000, Belgium.
541. Institute for Bioengineering and Biopharmaceutical Research (IBBR), Hanyang University, Seoul, South Korea.
542. University of Oslo, Oslo 0316, Norway.
543. Institut Bergonié, Bordeaux 33076, France.
544. Department of Research Oncology, Guy’s Hospital, King’s Health Partners AHSC, King’s College London School of Medicine, London SE1 9RT, UK.
545. University Hospital of Minjoz, INSERM UMR 1098, Besançon 25000, France.
546. Cambridge Breast Unit, Addenbrooke’s Hospital, Cambridge University Hospital NHS Foundation Trust and NIHR Cambridge Biomedical Research Centre, Cambridge CB2 2QQ, UK.
547. East of Scotland Breast Service, Ninewells Hospital, Aberdeen AB25 2XF, UK.
548. Oncologie Sénologie, ICM Institut Régional du Cancer, Montpellier 34298, France.
549. Los Alamos National Laboratory, Los Alamos, NM 87545, USA.
550. Department of Radiation Oncology, Radboud University Medical Centre, Nijmegen 6525 GA, The Netherlands.
551. University of Iceland, Reykjavik 101, Iceland.
552. Dundee Cancer Centre, Ninewells Hospital, Dundee DD2 1SY, UK.

553. Institut Curie, INSERM Unit 830, Paris 75248, France.
554. Department of Laboratory Medicine, Radboud University Nijmegen Medical Centre, Nijmegen GA 6525, The Netherlands.
555. Department of General Surgery, Singapore General Hospital, Outram Rd, Singapore 169608, Singapore.
556. Universite Lyon, INCa-Synergie, Centre Léon Bérard, Lyon 69008, France.
557. Giovanni Paolo II / I.R.C.C.S. Cancer Institute, Bari BA 70124, Italy.
558. Department of Biopathology, Centre Léon Bérard, Lyon 69008, France.
559. Université Claude Bernard Lyon 1, Villeurbanne 69100, France.
560. NCCS-VARI Translational Research Laboratory, National Cancer Centre Singapore, Singapore 169610, Singapore.
561. Department of Pathology, Erasmus Medical Center Rotterdam, Rotterdam 3015 GD, The Netherlands.
562. Division of Molecular Carcinogenesis, The Netherlands Cancer Institute, Amsterdam 1066 CX, The Netherlands.
563. Division of Molecular Carcinogenesis, The Netherlands Cancer Institute, Amsterdam, The Netherlands.
564. Institute of Human Genetics, Christian-Albrechts-University, Kiel 24118, Germany.
565. Institute of Human Genetics, Ulm University and Ulm University Medical Center of Ulm, Ulm 89081, Germany.
566. Institute of Human Genetics, University of Ulm and University Hospital of Ulm, Ulm 89081, Germany.
567. Hematopathology Section, Institute of Pathology, Christian-Albrechts-University, Kiel 24118, Germany.
568. Department of Human Genetics, Hannover Medical School, Hannover 30625, Germany.
569. Department of Pediatric Oncology, Hematology and Clinical Immunology, Heinrich-Heine-University, Düsseldorf 40225, Germany.
570. Department of Internal Medicine/Hematology, Friedrich-Ebert-Hospital, Neumünster 24534, Germany.
571. University Hospital Muenster - Pediatric Hematology and Oncology, Muenster 24534, Germany.
572. Department of Pediatrics, University Hospital Schleswig-Holstein, Kiel 24105, Germany.
573. Department of Medicine II, University of Würzburg, Würzburg, Germany.
574. Senckenberg Institute of Pathology, University of Frankfurt Medical School, Frankfurt 60596, Germany.
575. Institute of Pathology, Charité – University Medicine Berlin, Berlin 10117, Germany.
576. Department for Internal Medicine II, University Hospital Schleswig-Holstein, Kiel 24105, Germany.
577. Institute for Medical Informatics Statistics and Epidemiology, University of Leipzig, Leipzig 04109, Germany.
578. Department of Hematology and Oncology, Georg-Augusts-University of Göttingen, Göttingen 37073, Germany.
579. Institute of Cell Biology (Cancer Research), University of Duisburg-Essen, Essen D-45147, Germany.

580. MVZ Department of Oncology, PraxisClinic am Johannisplatz, Leipzig 04109, Germany.
581. Institute of Pathology, Ulm University and University Hospital of Ulm, Ulm 89081, Germany.
582. Department of Pathology, Robert-Bosch-Hospital, Stuttgart, Germany, Stuttgart 70376, Germany.
583. University Hospital Giessen, Pediatric Hematology and Oncology, Giessen 35392, Germany.
584. Institute of Clinical Molecular Biology, Christian-Albrechts-University, Kiel 24118, Germany.
585. Institute of Pathology, University of Wuerzburg, Wuerzburg 97070, Germany.
586. Department of General Internal Medicine, University Kiel, Kiel 24118, Germany.
587. Clinic for Hematology and Oncology, St.-Antonius-Hospital, Eschweiler D-52249, Germany.
588. Department for Internal Medicine III, University of Ulm and University Hospital of Ulm, Ulm 89081, Germany.
589. Neuroblastoma Genomics, German Cancer Research Center (DKFZ), Heidelberg 69120, Germany.
590. University of Düsseldorf, Düsseldorf 40225, Germany.
591. Department of Vertebrate Genomics/Otto Warburg Laboratory Gene Regulation and Systems Biology of Cancer, Max Planck Institute for Molecular Genetics, Berlin 14195, Germany.
592. St. Jude Children's Research Hospital, Memphis, TN 38105-3678, USA.
593. Heidelberg University Hospital, Heidelberg 69120, Germany.
594. Genomics and Proteomics Core Facility High Throughput Sequencing Unit, German Cancer Research Center (DKFZ), Heidelberg 69120, Germany.
595. Epigenomics and Cancer Risk Factors, German Cancer Research Center (DKFZ), Heidelberg 69120, Germany.
596. University Medical Center Hamburg-Eppendorf, Hamburg 20251, Germany.
597. Institute of Pathology, University Medical Center Hamburg-Eppendorf, Hamburg 20251, Germany.
598. Martini-Clinic, Prostate Cancer Center, University Medical Center Hamburg-Eppendorf, Hamburg 20095, Germany.
599. Division of Cancer Genome Research, German Cancer Research Center (DKFZ), Heidelberg 69120, Germany.
600. National Institute of Biomedical Genomics, Kalyani 741235, West Bengal, India.
601. Advanced Centre for Treatment Research & Education in Cancer, Tata Memorial Centre, Navi Mumbai, Maharashtra 410210, India.
602. Department of Pathology, General Hospital of Treviso, Department of Medicine, University of Padua, Italy, Treviso 31100, Italy.
603. Department of Medicine (DIMED), Surgical Pathology Unit, University of Padua, Padua 35121, Italy.
604. Division of Pathology and Clinical Laboratories, Department of Hepatobiliary and Pancreatic Oncology, Hepatobiliary and Pancreatic Surgery Division, National Cancer Center Hospital, Chuo-ku, Tokyo, 104-0045, Japan.
605. Division of Cancer Genomics, Department of Bioinformatics, National Cancer Center, Tokyo 104-0045, Japan.
606. Department of Pathology, Keio University School of Medicine, Tokyo 160-8582, Japan.
607. Department of Hepatobiliary and Pancreatic Oncology, National Cancer Center Hospital

(NCCH), Tokyo, 104-0045 Japan.
608. Department of Pathology, Graduate School of Medicine, The University of Tokyo, Bunkyo-ku, Tokyo 113-0033, Japan.
609. Preventive Medicine, Graduate School of Medicine, The University of Tokyo, Tokyo 113-0033, Japan.
610. Gastric Surgery Division, Division of Pathology and Clinical Laboratories, National Cancer Center Hospital, Tokyo 104-0045, Japan.
611. Department of Preventive Medicine, Graduate School of Medicine, The University of Tokyo, Tokyo 113-0033, Japan.
612. Division of Cancer Genomics, Department of Bioinformatics, National Cancer Center Research Institute, National Cancer Center, Tokyo 104-0045, Japan.
613. Department of Gastroenterology and Hepatology, Yokohama City University Graduate School of Medicine, Kanagawa 236-0004, Japan.
614. Laboratory of Molecular Medicine, Human Genome Center, The Institute of Medical Science, University of Tokyo, Shirokane-dai, Minato-ku, Tokyo 108-8639, Japan.
615. Department of Cancer Genome Informatics, Graduate School of Medicine, Osaka University, Osaka 565-0871, Japan.
616. Hiroshima University, Hiroshima 734-8553, Japan.
617. Tokyo Women's Medical University, Tokyo 162-8666, Japan.
618. Osaka International Cancer Center, Osaka 541-8567, Japan.
619. Wakayama Medical University, Wakayama 641-8509, Japan.
620. Hokkaido University, Sapporo 060-8648, Japan.
621. Division of Medical Oncology, National Cancer Centre, Singapore 169610, Singapore.
622. Cholangiocarcinoma Screening and Care Program and Liver Fluke and Cholangiocarcinoma Research Centre, Faculty of Medicine, Khon Kaen University, Khon Kaen, Thailand.
623. Lymphoma Genomic Translational Research Laboratory, National Cancer Centre, Singapore 169610, Singapore.
624. Center of Digestive Diseases and Liver Transplantation, Fundeni Clinical Institute, Bucharest 022328, Romania.
625. Division of Hepatobiliary and Pancreatic Surgery, Department of Surgery, School of Medicine, Keimyung University Dongsan Medical Center, Daegu 41931, South Korea.
626. Pathology, Hospital Clinic, Institut d'Investigacions Biomèdiques August Pi i Sunyer (IDIBAPS), University of Barcelona, Barcelona 8034, Spain.
627. Anatomia Patológica, Hospital Clinic, Institut d'Investigacions Biomèdiques August Pi i Sunyer (IDIBAPS), University of Barcelona, Barcelona 8036, Spain.
628. Spanish Ministry of Science and Innovation, Madrid 28046, Spain.
629. Hematology, Hospital Clinic, Institut d'Investigacions Biomèdiques August Pi i Sunyer (IDIBAPS), University of Barcelona, Barcelona 8034, Spain.
630. Departamento de Bioquımicay Biologıa Molecular, Facultad de Medicina, Instituto Universitario de Oncologıa-IUOPA, Oviedo 33006, Spain.
631. Departamento de Bioquímica y Biología Molecular, Instituto Universitario de Oncología (IUOPA), Universidad de Oviedo, Oviedo 33006, Spain.
632. Royal National Orthopaedic Hospital - Bolsover, London W1W 5AQ, UK.
633. Department of Pathology, Oslo University Hospital, Oslo O310, Norway.

634. Department of Pathology, Oslo University Hospital, Norway and University of Oslo, Norway, Oslo O310, Norway.
635. Department of Pathology (Research), University College London Cancer Institute, London WC1E 6BT, UK.
636. Department for Clinical Science, University of Bergen, Bergen 5020, Norway.
637. Research Department of Pathology, University College London Cancer Institute, 72 Huntley Street, London, WC1E 6BT.
638. East Anglian Medical Genetics Service, Cambridge University Hospitals NHS Foundation Trust, Cambridge CB2 0QQ, UK.
639. Royal National Orthopaedic Hospital - Stanmore, Stanmore, Middlesex HA7 4LP, UK.
640. Division of Orthopaedic Surgery, Oslo University Hospital, Oslo 0379, Norway.
641. Radcliffe Department of Medicine, University of Oxford, Oxford OX3 9DU, UK.
642. University of Pavia, Pavia 27100, Italy.
643. Karolinska Institute, Stockholm SE-171 76, Sweden.
644. Wellcome Sanger Institute, Wellcome Genome Campus, Hinxton, Cambridge, CB10 1SD, UK.
645. University of Oxford, Oxford OX3 9DU, UK.
646. Salford Royal NHS Foundation Trust, Salford M6 8HD, UK.
647. Gloucester Royal Hospital, Gloucester, GL1 3NL, UK.
648. Royal Stoke University Hospital, Stoke-on-Trent ST4 6QG, UK.
649. St Thomas's Hospital, London SE1 7EH, UK.
650. Imperial College NHS Trust, Imperial College, London W2 INY, UK.
651. Department of Histopathology, Salford Royal NHS Foundation Trust, Salford M6 8HD, UK.
652. Faculty of Biology, Medicine and Health, University of Manchester, Salford M6 8HD, UK.
653. Edinburgh Royal Infirmary, Edinburgh EH16 4SA, UK.
654. Barking Havering and Redbridge University Hospitals NHS Trust, Romford, RM7 0AG, UK.
655. King's College London and Guy's and St Thomas' NHS Foundation Trust, London SE1 7EH, UK.
656. Cambridge Oesophagogastric Centre, Cambridge University Hospitals NHS Foundation Trust, Cambridge, CB2 0QQ.
657. Nottingham University Hospitals NHS Trust, Nottingham NG7 2UH, UK.
658. St Luke's Cancer Centre, Royal Surrey County Hospital NHS Foundation Trust, Guildford GU2 7XX, UK.
659. University of North Carolina at Chapel Hill, Chapel Hill, NC 27599, USA.
660. Norfolk and Norwich University Hospital NHS Trust, Norwich NR4 7UY, UK.
661. University Hospitals Coventry and Warwickshire NHS Trust, Coventry CV2 2DX, UK.
662. University Hospitals Birmingham NHS Foundation Trust, Birmingham B15 2GW, UK.
663. Centre for Cancer Research and Cell Biology, Queen's University, Belfast BT9 7AB, UK.
664. School of Cancer Sciences, Faculty of Medicine, University of Southampton, Southampton SO17 1BJ, University Hospital Southampton NHS Foundation Trust, Southampton, SO16 6YD, UK.
665. Wythenshawe Hospital, Manchester M23 9LT, UK.
666. Royal Marsden NHS Foundation Trust, London and Sutton SW3 6JJ, UK.
667. Barts Cancer Institute, Barts and the London School of Medicine and Dentistry, Queen

Mary University of London, London EC1M 6BQ, UK.
668. University Hospital Southampton NHS Foundation Trust, Southampton SO16 6YD, UK.
669. HCA Laboratories, London W1G 8AQ, UK.
670. University of Liverpool, Liverpool L69 3BX, UK.
671. Academic Urology Group, Department of Surgery, University of Cambridge, Cambridge CB2 0QQ, UK.
672. University of Oxford, Oxford, OX3 9DU, UK.
673. Second Military Medical University, Shanghai 200433, China.
674. Department of Surgery and Cancer, Imperial College, London W2 INY, UK.
675. The Chinese University of Hong Kong, Shatin, Hong Kong, China.
676. Nuffield Department of Surgical Sciences, John Radcliffe Hospital, University of Oxford, Headington, Oxford OX3 9DU, UK.
677. Department of Histopathology, Cambridge University Hospitals NHS Foundation Trust, Cambridge CB2 0QQ, UK.
678. Department of Bioinformatics and Computational Biology / Department of Systems Biology, The University of Texas MD Anderson Cancer Center, Houston, TX 77030, USA.
679. Department of Pathology, The University of Texas MD Anderson Cancer Center, Houston, TX 77030, USA.
680. Canada's Michael Smith Genome Sciences Center, BC Cancer Agency, Vancouver, BC V5Z 4S6, Canada.
681. Center for Molecular Oncology, Memorial Sloan Kettering Cancer Center, New York, NY 10065, USA.
682. University Health Network, Toronto, ON M5G 2C4, Canada.
683. Department of Pathology and Laboratory Medicine, School of Medicine, University of North Carolina at Chapel Hill, Chapel Hill, NC 27599, USA.
684. Department of Population and Quantitative Health Sciences, Case Western Reserve University School of Medicine, Cleveland, OH 44016, USA.
685. Research Health Analytics and Informatics, University Hospitals Cleveland Medical Center, Cleveland, OH 44106, USA.
686. Arnie Charbonneau Cancer Institute, University of Calgary, Calgary, AB T2N 4N2, Canada.
687. Departments of Surgery and Oncology, University of Calgary, Calgary, AB T2N 4N2, Canada.
688. Sidney Kimmel Cancer Center, The Johns Hopkins Medical Institutions, Baltimore, MD 21230, USA.
689. Buck Institute for Research on Aging, Novato, CA 94945, USA.
690. Duke University Medical Center, Durham, NC 27710, USA.
691. Norris Comprehensive Cancer Center, University of Southern California, Los Angeles, CA 90033, USA.
692. The Preston Robert Tisch Brain Tumor Center, Duke University Medical Center, Durham, NC 27710, USA.
693. University of Southern California, USC/Norris Comprehensive Cancer Center, Los Angeles, CA 90033, USA.
694. Departments of Dermatology and Pathology, Yale University, New Haven, CT 06510, USA.
695. Fox Chase Cancer Center, Philadelphia, PA 19111, USA.
696. Johns Hopkins University, Baltimore, MD 21287, USA.

697. University of Michigan Comprehensive Cancer Center, Ann Arbor, MI 48109, USA.
698. University of Alabama at Birmingham, Birmingham, AL 35294, USA.
699. Division of Anatomic Pathology, Mayo Clinic, Rochester, MN 55905, USA.
700. Division of Experimental Pathology, Mayo Clinic, Rochester, MN 55905 USA.
701. The Sidney Kimmel Comprehensive Cancer Center at Johns Hopkins University, Baltimore, MD 21287, USA.
702. International Genomics Consortium, Phoenix, AZ 85004, USA.
703. Departments of Pediatrics and Genetics, University of North Carolina at Chapel Hill, Chapel Hill, NC 27599, USA.
704. Department of Pathology, UPMC Shadyside, Pittsburgh, PA 15232, USA.
705. Center for Cancer Genomics, National Cancer Institute, National Institutes of Health, Bethesda, MD 20892, USA.
706. Istituto Neurologico Besta, Department of Neuro-Oncology, Milano 20133, Italy.
707. University of Queensland Thoracic Research Centre, The Prince Charles Hospital, Brisbane, QLD 4032, Australia.
708. Department of Neurosurgery, University of Florida, Gainesville, FL 32610, USA.
709. Center for Biomedical Informatics, Harvard Medical School, Boston, MA 02115, USA.
710. Department of Cancer Biology, The University of Texas MD Anderson Cancer Center, Houston, TX 77030, USA.
711. Department of Surgical Oncology, The University of Texas MD Anderson Cancer Center, Houston, TX 77030, USA.
712. Division of Gastroenterology and Hepatology, Mayo Clinic, Rochester, MN 55905, USA.
713. University of Miami, Sylvester Comprehensive Cancer Center, Miami, FL 33136, USA.
714. University of Southern California, Norris Comprehensive Cancer Center, Los Angeles, CA 90033, USA.
715. The Sol Goldman Pancreatic Cancer Research Center, Department of Pathology, Johns Hopkins Hospital, Baltimore, MD 21287, USA.
716. Centre for Translational and Applied Genomics, British Columbia Cancer Agency, Vancouver, BC V5Z 1L3, Canada.
717. Department of Pathology & Immunology, Baylor College of Medicine, Houston, TX 770230, USA.
718. Michael E. DeBakey Veterans Affairs Medical Center, Houston, TX 770230, USA.
719. Carolina Center for Genome Sciences, University of North Carolina at Chapel Hill, Chapel Hill, NC 27599, USA.
720. Canada’s Michael Smith Genome Sciences Centre, BC Cancer, Vancouver, BC V5Z 4S6, Canada.
721. Indivumed GmbH, Hamburg 20251, Germany.
722. Division of Hepatobiliary and Pancreatic Surgery, Department of Surgery, School of Medicine, Keimyung University Dong-san Medical Center, Daegu 41931, South Korea.
723. Women's Cancer Program at the Samuel Oschin Comprehensive Cancer Institute, Cedars-Sinai Medical Center, Los Angeles, CA 90048, USA.
724. Canada’s Michael Smith Genome Sciences Centre, BC Cancer Agency, Vancouver, BC V5Z 4S6, Canada.
725. Department of Surgery, The George Washington University, School of Medicine and Health

Science, Washington, DC 20052, USA.
726. Endocrine Oncology Branch, Center for Cancer Research, National Cancer Institute, National Institutes of Health, Bethesda, MD 20892, USA.
727. ILSbio, LLC Biobank, Chestertown, MD 21620, USA.
728. Gynecologic Oncology, NYU Laura and Isaac Perlmutter Cancer Center, New York University, New York, NY 10016, USA.
729. Division of Oncology, Stem Cell Biology Section, Washington University School of Medicine, St. Louis, MO 63110, USA.
730. Urologic Oncology Branch, Center for Cancer Research, National Cancer Institute, National Institutes of Health, Bethesda, MD 20892, USA.
731. Department of Systems Biology, University of Texas MD Anderson Cancer Center, Houston, TX 77030, USA.
732. Institute for Systems Biology, Seattle, WA 98109, USA.
733. Institute for Genomic Medicine, Nationwide Children's Hospital, Columbus, OH 43215, USA.
734. Department of Surgery, Duke University, Durham, NC 27710, USA.
735. Department of Obstetrics, Gynecology and Reproductive Services, University of California San Francisco, San Francisco, CA 94143, USA.
736. Departments of Neurology and Neurosurgery, Henry Ford Hospital, Detroit, MI 48202, USA.
737. Department of Pathology, Roswell Park Cancer Institute, Buffalo, NY 14263, USA.
738. Department of Obstetrics and Gynecology, Division of Gynecologic Oncology, Washington University School of Medicine, St. Louis, MO 3110, USA.
739. Penrose St. Francis Health Services, Colorado Springs, CO 80907, USA.
740. The University of Chicago, Chicago, IL 60637, USA.
741. Department of Neurology, Mayo Clinic, Rochester, MN 55905, USA.
742. Department of Genetics and Lineberger Comprehensive Cancer Center, University of North Carolina at Chapel Hill, Chapel Hill, NC 27599, USA.
743. NYU Langone Medical Center, New York, NY 10016, USA.
744. Department of Hematology and Medical Oncology, Cleveland Clinic, Cleveland, OH 44195, USA.
745. Department of Genetics, Department of Pathology and Laboratory Medicine, School of Medicine, University of North Carolina at Chapel Hill, Chapel Hill, NC 27599, USA.
746. Helen F. Graham Cancer Center at Christiana Care Health Systems, Newark, DE 19713, USA.
747. Cureline, Inc, South San Francisco, CA 94080, USA.
748. Department of Obstetrics and Gynecology, Medical College of Wisconsin, Milwaukee, WI 53226, USA.
749. Emory University, Atlanta, GA 30322, USA.
750. Vanderbilt University, Vanderbilt Ingram Cancer Center, Nashville, TN 37232, USA.
751. Ohio State University College of Medicine and Arthur G. James Comprehensive Cancer Center, Columbus, OH 43210, USA.
752. Research Computing Center, University of North Carolina at Chapel Hill, Chapel Hill, NC 27599, USA.

753. Analytical Biological Services, Inc, Wilmington, DE 19801, USA.
754. Department of Dermatology, University Hospital Essen, Westdeutsches Tumorzentrum & German Cancer Consortium, Essen 45122, Germany.
755. University of Pittsburgh, Pittsburgh, PA 15213, USA.
756. Murtha Cancer Center, Walter Reed National Military Medical Center, Bethesda, MD 20889, USA.
757. Brigham and Women's Hospital, Harvard Medical School, Boston, MA 02115, USA.
758. Department of Surgery, Memorial Sloan Kettering Cancer Center, New York, NY 10065, USA.
759. Department of Gynecologic Oncology & Reproductive Medicine, and Center for RNA Interference and Non-Coding RNA, the University of Texas MD Anderson Cancer Center, Houston, TX 77030, USA.
760. Department of Urology, Mayo Clinic, Rochester, MN 55905, USA.
761. Johns Hopkins Medical Institutions, Baltimore, MD 21205, USA.
762. Departments of Neurosurgery and Hematology and Medical Oncology, Winship Cancer Institute and School of Medicine, Emory University, Atlanta, GA 30322, USA.
763. Georgia Regents University Cancer Center, Augusta, GA 30912, USA.
764. Thoracic Oncology Laboratory, Mayo Clinic, Rochester, MN 55905, USA.
765. Institute for Genomic Medicine, Nationwide Children's Hospital, Columbus, OH 43205, USA.
766. Department of Obstetrics & Gynecology, Division of Gynecologic Oncology, Mayo Clinic, Rochester, MN 55905, USA.
767. International Institute for Molecular Oncology, Poznań 60-203, Poland.
768. Poznan University of Medical Sciences, Poznań 61-701, Poland.
769. Edison Family Center for Genome Sciences and Systems Biology, Washington University, St. Louis, MO 63110, USA.
770. Department of Pediatric Oncology and Hematology, University of Cologne, Cologne, Germany

Supplementary Tables: (supplied separately)

Supplementary Table 1: Genomic element type summary statistics

Supplementary Table 2: Driver discovery method description

Supplementary Table 3: Driver discovery methods included in integration

Supplementary Table 4: Summary and annotation of protein-coding driver candidates

Supplementary Table 5: Summary and annotation of non-coding driver candidates

Supplementary Table 6: Summary of additional evidence for non-coding driver candidates

Supplementary Table 7: List of cancer genes used in this study

Supplementary Table 8: Impact of covariates on obs/exp ratio

Supplementary Table 9: Significant bins in genome-wide analysis

Supplementary Table 10: Expression correlations for driver candidates

Supplementary Table 11: Significant and near significant hits from restricted hypothesis testing

Supplementary Table 12: Significantly amplified and deleted regions called by GISTIC

Supplementary Table 13: Data sources and covariates used in the SRB model

Supplementary Table 14: Significant recurrent breakpoints (SRBs)

Supplementary Table 15: Significant recurrent juxtapositions (SRJs)

Supplementary Table 16: Top ten SCNAs and SNVs by tissue type

Supplementary Table 17: SRBs in 500 kb windows

Supplementary Table 18: Number of indels and SNVs in different regions of 18 genes enriched in 2-5 bp indels

Supplementary Table 19: LOH ratios in aggregates of cancer and passenger genes

Supplementary Table 20: Links to data files
